# COVID-19 Vaccination and Incidence of Pediatric SARS-CoV-2 Infection and Hospitalization

**DOI:** 10.1001/jamanetworkopen.2024.7822

**Published:** 2024-04-23

**Authors:** Jennifer R. Head, Philip A. Collender, Tomás M. León, Lauren A. White, Sohil R. Sud, Simon K. Camponuri, Vivian Lee, Joseph A. Lewnard, Justin V. Remais

**Affiliations:** 1 Department of Epidemiology, University of Michigan, Ann Arbor; 2 Insitute for Global Change Biology, University of Michigan, Ann Arbor; 3 Division of Environmental Health Sciences, University of California, Berkeley; 4 California Department of Public Health, Richmond; 5 College of Letters and Sciences, University of California, Berkeley; 6 Division of Epidemiology, University of California, Berkeley; 7 Center for Computational Biology, University of California, Berkeley

## Abstract

**Importance:**

A SARS-CoV-2 vaccine was approved for adolescents aged 12 to 15 years on May 10, 2021, with approval for younger age groups following thereafter. The population level impact of the pediatric COVID-19 vaccination program has not yet been established.

**Objective:**

To identify whether California's pediatric COVID-19 immunization program was associated with changes in pediatric COVID-19 incidence and hospitalizations.

**Design, Setting, and Participants:**

A case series on COVID-19 vaccination including children aged 6 months to 15 years was conducted in California. Data were obtained on COVID-19 cases in California between April 1, 2020, and February 27, 2023.

**Exposure:**

Postvaccination evaluation periods spanned 141 days (June 10 to October 29, 2021) for adolescents aged 12 to 15 years, 199 days (November 29, 2021, to June 17, 2022) for children aged 5 to 11 years, and 225 days (July 17, 2022, to February 27, 2023) for those aged 6 to 59 months. During these periods, statewide vaccine coverage reached 53.5% among adolescents aged 12 to 15 years, 34.8% among children aged 5 to 11 years, and 7.9% among those aged 6 to 59 months.

**Main Outcomes and Measures:**

Age-stepped implementation of COVID-19 vaccination was used to compare observed county-level incidence and hospitalization rates during periods when each age group became vaccine eligible to counterfactual rates predicted from observations among other age groups. COVID-19 case and hospitalization data were obtained from the California reportable disease surveillance system.

**Results:**

Between April 1, 2020, and February 27, 2023, a total of 3 913 063 pediatric COVID-19 cases and 12 740 hospitalizations were reported in California. Reductions of 146 210 cases (95% prediction interval [PI], 136 056-158 948) were estimated among adolescents aged 12 to 15 years, corresponding to a 37.1% (35.5%-39.1%) reduction from counterfactual predictions. Reductions of 230 134 (200 170-265 149) cases were estimated among children aged 5 to 11 years, corresponding to a 23.7% (20.6%-27.3%) reduction from counterfactual predictions. No evidence of reductions in COVID-19 cases statewide were found among children aged 6 to 59 months (estimated averted cases, −259; 95% PI, −1938 to 1019), although low transmission during the evaluation period may have limited the ability to do so. An estimated 168 hospitalizations (95% PI, 42-324) were averted among children aged 6 to 59 months, corresponding to a 24.4% (95% PI, 6.1%-47.1%) reduction. In meta-analyses, county-level vaccination coverage was associated with averted cases for all age groups. Despite low vaccination coverage, pediatric COVID-19 immunization in California averted 376 085 (95% PI, 348 355-417 328) reported cases and 273 (95% PI, 77-605) hospitalizations among children aged 6 months to 15 years over approximately 4 to 7 months following vaccination availability.

**Conclusions and Relevance:**

The findings of this case series analysis of 3 913 063 cases suggest reduced pediatric SARS-CoV-2 transmission following immunization. These results support the use of COVID-19 vaccines to reduce COVID-19 incidence and hospitalization in pediatric populations.

## Key Points

**Question:**

Was implementation of the pediatric COVID-19 immunization program of California associated with reductions in the reported pediatric COVID-19 incidence and hospitalizations?

**Finding:**

In this case series including 3.9 million children, pediatric vaccination was estimated to avert 146 210 cases of COVID-19 among adolescents aged 12 to 15 years during a 141-day postvaccine evaluation period and 230 134 cases among children aged 5 to 11 years during a 199-day postvaccine evaluation period. In addition, an estimated 168 hospitalizations were averted among children aged 6 to 59 months during a 225-day evaluation period.

**Meaning:**

The findings of this study suggest that vaccination against SARS-CoV-2 was associated with significant reductions in COVID-19 incidence and hospitalizations among children in California.

## Teaser

This case series examines the cases and hospitalizations of children due to COVID-19 in California following eligibility by age group for SARS-CoV-2 vaccination.

## Introduction

**Figure 1.**
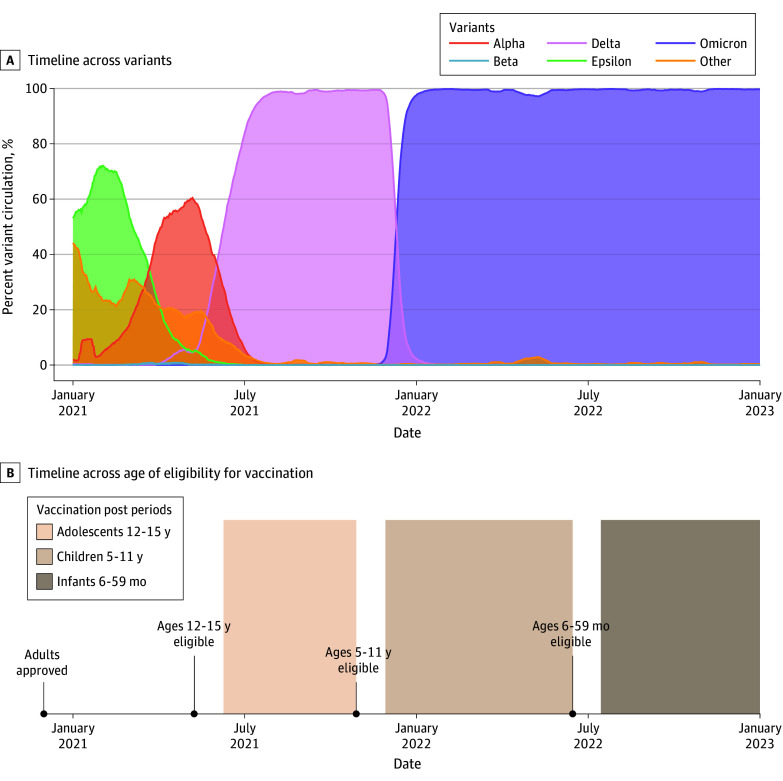
Timeline of Major Circulating Variants and Vaccine Eligibility by Age Group Timelines of major circulating variants (A) and eligibility, with the age groups examined shown as shaded rectangles (B).

Vaccination is among the most important interventions to reduce the public health impact of infectious diseases.^1^ SARS-CoV-2 mRNA vaccines, including mRNA1273 (Moderna) and BNT162b2 (Pfizer BioNTech), were approved for adult use in December 2020.^2^ On May 10, 2021, the first mRNA COVID-19 vaccine was approved for use in adolescents aged 12 to 15 years. Vaccines were subsequently approved for children aged 5 to 11 years on October 29, 2021, and for children aged 6 to 59 months on June 17, 2022 (Figure 1).^2^

COVID-19 vaccines are safe for children.^3^ However, concerns over vaccine-related adverse events, lower vaccine effectiveness against illness in children, and perceptions of a milder disease course in children have resulted in high rates of parental vaccine hesitancy^4,5,6^ and resistance to pediatric vaccine mandates.^7^ While California has among the highest rates of vaccination in the US,^8^ pediatric vaccination coverage lags that of adults substantially, with only 8.2% of children younger than 5 years and 37.8% of children aged 5 to 11 years fully vaccinated as of May 2023.^8^ Severe manifestations of COVID-19 are rare among children, but can occur.^9^ Understanding the population-level impact of COVID-19 vaccinations in SARS-CoV-2 infections and hospitalizations in pediatric populations would aid in public health decision-making on pediatric vaccine and booster policy and provide pediatric-specific information on vaccine outcomes that could be applied to future SARS-CoV-2 variants.

Herein, we analyze data on 3 913 063 pediatric cases of COVID-19 and 12 740 hospitalizations from California. Using the phased introduction of the vaccine to individuals aged 12 to 15 years, 5 to 11 years, and 6 to 59 months, we estimated statewide and county-specific outcomes associated with vaccination on pediatric incidence and hospitalizations in California.

## Methods

### Epidemiologic Data

We obtained deidentified information on all pediatric COVID-19 cases reported in California between April 1, 2020, and February 27, 2023, from the California COVID-19 Reporting System, along with the patient’s county of residence, age, and hospitalization status. Each case was confirmed using a nucleic acid amplification test. Because the research constitutes a public health surveillance activity, the study did not constitute human research and does not require institutional review board review or exemption according to the Common Rule (45 CFR §46). We followed the reporting guideline for case series studies.

Daily cases were aggregated by county and age groups based on dates of vaccination eligibility: 0 to 5 months (vaccine ineligible), 6 to 59 months, 5 to 11 years, 12 to 15 years, and older than 16 years (nonpediatric). To remove variation due to differential health care seeking by day of week, we calculated 7-day moving averages of case counts per county and age group. Due to small counts for pediatric hospitalizations, we aggregated hospitalizations by week and age group within 5 California-designated regions (eFigure 1 in Supplement 1). Descriptions of other covariate data are covered in the eMethods in Supplement 1).

### Statistical Analysis

#### Training and Prediction Periods

For each age group of interest (6-59 months, 5-11 years, and 12-15 years), we split data into age-eligible and age-ineligible periods. The prevaccine eligibility period encompassed data up to the date of vaccine eligibility. The evaluation period for the outcome associated with the vaccine lasted from 1 month following the date of vaccine eligibility (to allow time to complete 2 doses) until the date that the next age group became eligible or until the time of analysis (February 27, 2023) (Figure 1). Ending the evaluation period at the vaccine eligibility date of the next eligible age group permitted that age group to be selected as a control time series in our predictive models.

#### Candidate Model Generation and Selection

We developed a set of candidate predictive generalized linear models, which are described in greater detail in the eMethods in Supplement 1. Predictors eligible for selection within candidate models included (1) log-incidence series for other age groups (ie, <6 months, 6-59 months, 5-11 years, 12-15 years, and ≥16 years, omitting the group being modeled) included as either covariates or as an offset term for any 1 age group; (2) an indicator of vaccine age eligibility for other age groups; (3) an indicator for in-person school being in session; (4) interactions between school and vaccine introduction indicators and time series for other age groups, aiming to account for differences in constant proportionality during school periods or when 1 age group became vaccinated; and (5) seasonal controls. Eligible predictors are summarized in eTable 1 in Supplement 1. Quasi-Poisson distributions were fit for the outcome to account for overdispersion. Models were developed separately for each California county.

Candidate models for hospitalizations included similar eligible predictors, with 2 main differences: log weekly case incidence series for other age groups was lagged by 2 weeks in accordance with the expected lag between infection and hospitalization^10^ and unlagged weekly hospitalizations across other age groups were included as possible predictors. Models were developed separately for each of the 5 regions.

We used a time series with a 1-year gap cross-validation approach (eMethods and eFigure 2 in Supplement 1) to select the best predictive model for each age group and geographic area (county or region) within the prevaccine period.^11,12,13^ For each area-age group combination, we selected the model with the lowest out-of-sample mean square error across holdout folds. For this model, we also calculated the coefficient of determination, *r^2^*, a goodness-of-fit metric. The selected predictors varied by area and age group. Selected models for each area-age group combination are included in eTable 2 (for cases) and eTable 3 (for hospitalizations) in Supplement 1.

#### Calculation of Vaccine Outcomes and Association Between Averted Cases and Vaccination Coverage

Selected models were fit to prevaccine eligibility data for their age group and geographic area and then used to predict counterfactual incidence or hospitalization in the postvaccine period or the expected case or hospitalization counts had vaccination not occurred. For inference, we computed 95% prediction intervals (PIs) around the counterfactual predictions, using a sandwich estimator to account for overdispersion when computing SEs (eMethods in Supplement 1).^14^ Prediction intervals, which are wider than CIs, capture the uncertainty around each future predicted value. Statewide estimates were obtained by summing predictions across geographies (eMethods in Supplement 1).

We estimated the absolute and relative differences between predicted counterfactual values and observed values for each county or region during the postvaccine evaluation period. To understand the association between vaccination coverage and averted cases, we fit regression models relating the reduction in cases within each age and county to county-level vaccination coverage within the same age group, using a fixed-effects meta-analysis with weights equal to the inverse estimated SE of the estimates per county. We used segmented regression models (eMethods in Supplement 1) to examine whether there were coverages below which reductions in cases could not be identified or above which diminishing returns on vaccination were observed.^15^

To examine whether postvaccine predictions from a different, but well predictive model, yielded similar estimates of vaccination outcomes, we repeated model selection using the mean absolute error instead of the mean square error in our cross-validation algorithm. We conducted jackknife analyses to examine whether postvaccine predictions from any one county were driving observed effects, dropping each county in turn from the overall pool of counties and recalculating the primary analytic end point of cases averted.

All analyses were conducted in R, version 3.6.0 (R Foundation for Statistical Computing).^16^

## Results

### Descriptive Results

Between April 1, 2020, and February 27, 2023, a total of 3 913 063 COVID-19 cases were reported in California among individuals aged 18 years or younger. Of these, 47 174 cases (1.2%) were among children younger than 6 months, 517 447 (13.2%) in children aged 6 to 59 months, 1 590 806 (40.7%) in children aged 5 to 11 years, and 1 511 690 (38.6%) in adolescents aged 12 to 15 years. A total of 12 740 hospitalizations were reported: 1443 (11.3%) were among children younger than 6 months, 3428 (26.9%) in children aged 6 to 59 months, 2536 (19.9%) in children aged 5 to 11 years, and 3921 (30.8%) in adolescents aged 12 to 15 years.

### Vaccine-Attributable Averted Cases and Hospitalizations by Pediatric Age Group

As shown in eFigure 3 in Supplement 1, *r*^2^ values for models fit to daily case data were 0.92 (IQR, 0.79-0.96) for children aged 6 to 59 months, 0.89 (IQR, 0.78-0.95) for children aged 5 to 11 years, and 0.79 (IQR, 0.62-0.90) for adolescents aged 12 to 15 years. eFigure 4 in Supplement 1 shows the model fit for hospitalizations. More details on model fit are included in the eResults in Supplement 1.

#### Adolescents Aged 12 to 15 Years

**Figure 2.**
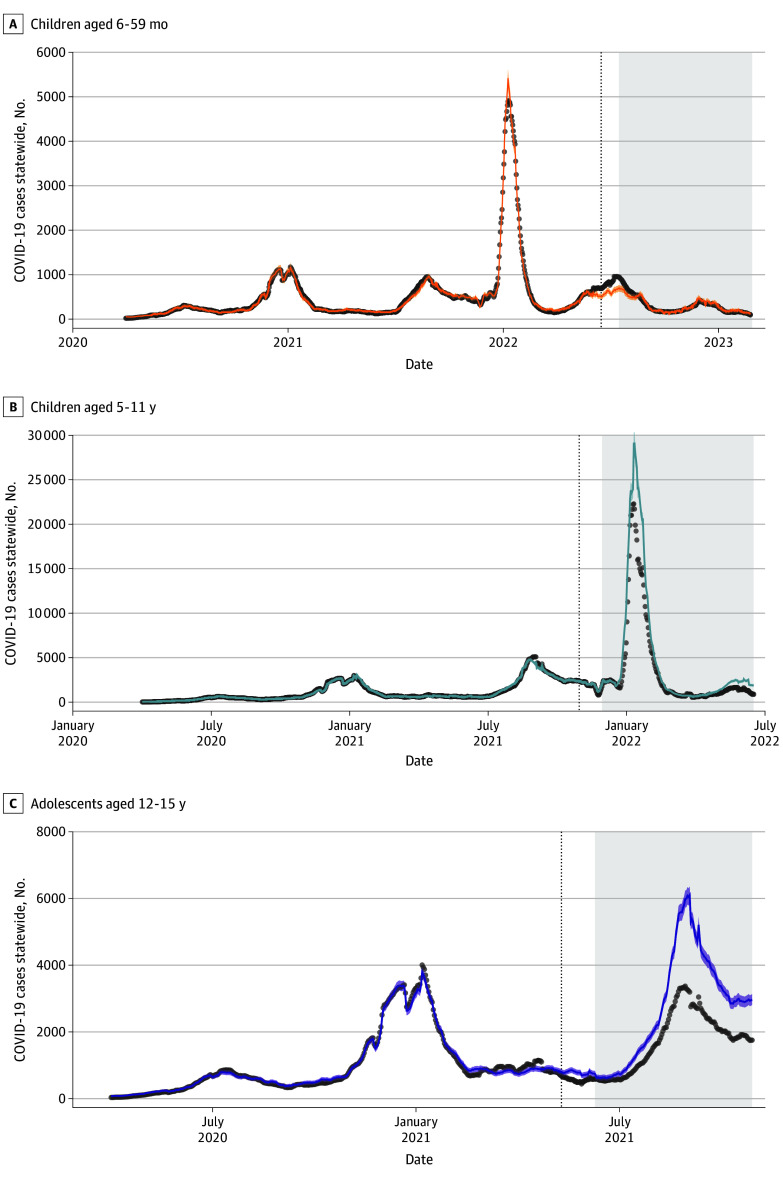
Comparison of Observed and Counterfactual Model Predictions for COVID-19 Cases by Age Statewide Comparisons shown for ages 6 to 59 months (A), 5 to 11 years (B), and 12 to 15 years (C). Gray dots represent daily observed COVID-19 cases, smoothed using a 7-day rolling average. Solid lines indicate the model predictions and shaded regions indicate the 95% prediction interval. Vertical lines indicate the date the age group became eligible for vaccination. The postvaccine evaluation period is shown in the shaded region. Additional plots from all counties are included in eFigures 9-11 in Supplement 1.

Individuals aged 12 to 15 years were eligible to be vaccinated against SARS-CoV-2 as of May 10, 2021. By October 29, 2021, when the next age group became eligible, 53.5% of this population had completed the 2-dose primary series of the vaccine, corresponding to 1 712 686 individuals. County-level vaccination rates ranged from 11.5% to 85.7%.^8^ During the 141 days spanning June 10 to October 29, 2021, 247 700 COVID-19 cases were observed among individuals aged 12 to 15 years. We estimated that 394 506 (95% PI, 392 545-396 467) cases of COVID-19 would have occurred absent vaccination, corresponding to 146 210 (95% PI, 136 056-158 948) cases averted statewide or 37.1% (95% PI, 34.5%-40.3%) of expected cases (Table, Figure 2C). Incidence plots from all counties are included as eFigures 9-14 in Supplement 1.

**Table.** Statewide Estimates of Averted COVID-19 Cases and Hospitalizations in California Due to Vaccination Among Children Aged 6 Months to 15 Years^a^

Age group (vaccine eligibility date)	Postvaccine period (length, d)	Children vaccinated, No. (%)	Observed, No.		Expected, No. (95% PI)		Averted, No. (95% PI)		% Averted (95% PI)	
			Cases	Hospitalizations	Cases	Hospitalizations	Cases	Hospitalizations	Cases	Hospitalizations
12-15 y (May 10, 2021)	June 10, 2021, to October 29, 2021 (144)	1 712 868 (53.5)	248 296	688	394 506 (392 545 to 396 467)	747 (623 to 932)	146 210 (136 056 to 158 948)	59 (−65 to 244)	37.1 (34.5 to 40.3)	7.9 (−8.7 to 32.7)
5-11 y (October 29, 2021)	November 19, 2021, to June 17, 2022 (199)	1 219 432 (34.8)	739 830	729	969 964 (940 000 to 1 000 497)	775 (650 to 950)	230 134 (200 170 to 265 149)	46 (−79 to 221)	23.7 (20.6 to 27.3)	5.8 (−10.2 to 28.6)
6-59 mo (June 17, 2022)	July 17, 2022, to February 27, 2023 (225)	177 087 (7.9)	67 287	520	67 027 (66 321 to 67 733)	688 (561 to 844)	−259 (−1938 to 1019)	168 (42 to 324)	−0.4 (−2.9 to 1.5)	24.4 (6.1 to 47.1)

Abbreviation: PI, prediction interval.

^a^ The expected number of cases and hospitalizations for each age group is calculated according to the counterfactual predictions.

**Figure 3.**
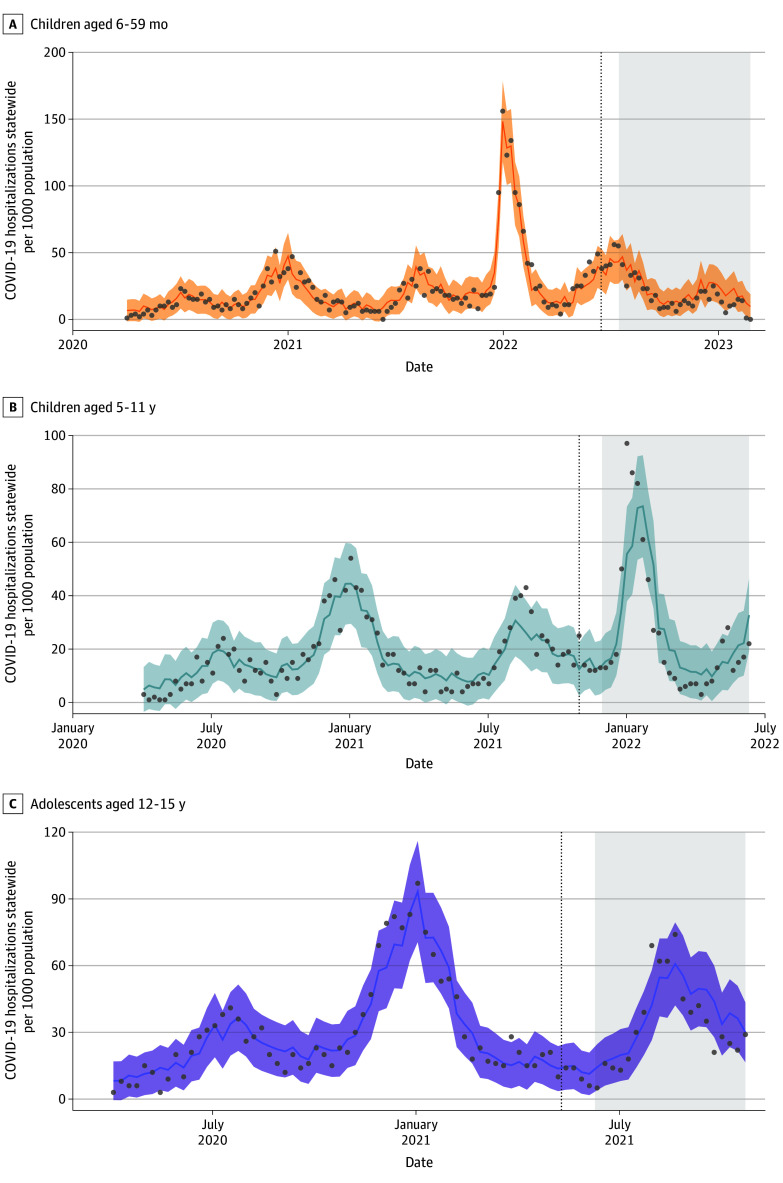
Comparison of Observed and Counterfactual Model Predictions for Statewide COVID-19 Hospitalizations Among Individuals by Age Comparisons shown for ages 6 to 59 months (A), 5 to 11 years (B), and 12 to 15 years (C). Gray dots represent weekly observed COVID-19 hospitalizations. Solid colored lines indicate the model predictions and shaded regions indicate the 95% prediction interval. Vertical lines indicate the date the age group became eligible for vaccination. The post period is shown in the shaded region. Plots from all regions are included in eFigures 12-14 in Supplement 1.

During this same 141-day period, 688 hospitalizations were observed among adolescents. We estimated that 59 (95% PI, −65 to 244) hospitalizations were averted or a reduction of 7.9% (95% PI, −8.7% to 32.7%) from expectation (Table, Figure 3C). Hospitalization plots from all regions are included as eFigures 12-14 in Supplement 1.

#### Children Aged 5 to 11 Years

Children aged 5 to 11 years were eligible for vaccination on October 29, 2021. By June 17, 2022, 1 219 432 individuals (34.8% of this population) had completed a primary series of the vaccine, with a range of 10.0% to 74.7% by county.^8^ During the 199-day period following November 29, 2021, we estimated that 230 134 (95% PI, 200 170-265 149) cases were averted due to the vaccine corresponding to a reduction of 23.7% (95% PI, 20.6%-27.3%) from counterfactual expectations (Table, Figure 2B). During this same period, we estimated that 46 (95% PI, −79 to 221) hospitalizations were averted, corresponding to 5.8% (95% PI, −10.2% to 28.6%) of expected hospitalizations (Table, Figure 3B).

#### Children Aged 6 to 59 Months

Children aged 6 to 59 months were eligible for vaccination on June 17, 2022. By February 27, 2023, 177 087 (7.9%) individuals had received both doses of the primary series, with a range of 0.7% to 38.5% across counties.^8^ In the 225 days following July 17, 2022, we did not detect any significant changes in cases from counterfactual expectations in the postvaccine period (estimated averted cases: −259; 95% PI, −1938 – 1019) (Table). The postvaccine evaluation period for this age group did not include a surge in COVID-19 cases as it did for the other age groups (Figure 2A). However, we estimated that 168 (95% PI, 42-324) hospitalizations were averted following vaccination, or a reduction of 24.4% (95% PI, 6.1%-47.1%) from counterfactual expectations (Table and Figure 3A). Summing across all age groups, we estimated that pediatric vaccination was associated with reductions of 376 085 (95% PI, 348 355-417 328) reported cases and 273 (95% PI, 77-605) hospitalizations among children aged 6 months to 15 years during the 4 to 7 months following vaccine availability. This represents a reduction of 26.3% of the number of cases and 12.4% of the hospitalizations that would have been seen in this population absent the vaccine.

#### Sensitivity Analyses

As indicated in the eResults and eFigures 5 and 6 in Supplement 1, results for individuals aged 5 to 15 years were not sensitive to the inclusion of any single county, although results for children aged 6 to 59 months were sensitive to the inclusion of Los Angeles (eFigure 7 in Supplement 1). Estimated cases (eTable 4 in Supplement 1) and hospitalizations (eTable 5 in Supplement 1) were consistent when model selection was done using mean absolute error as the loss function for children aged 5 to 15 years. Estimated averted cases in children aged 6 to 59 months were slightly lower using mean absolute error, but hospitalization results were consistent (eResults in Supplement 1). Estimates of cases averted (eTable 6 in Supplement 1) and hospitalizations averted (eTable 7 in Supplement 1) made using the mean absolute error as the loss function for each county or region are available, along with plots of observed and counterfactual case and hospitalization series for all geographic areas (eFigures 9-14 in Supplement 1).

### Association Between Averted Cases and Vaccination

**Figure 4.**
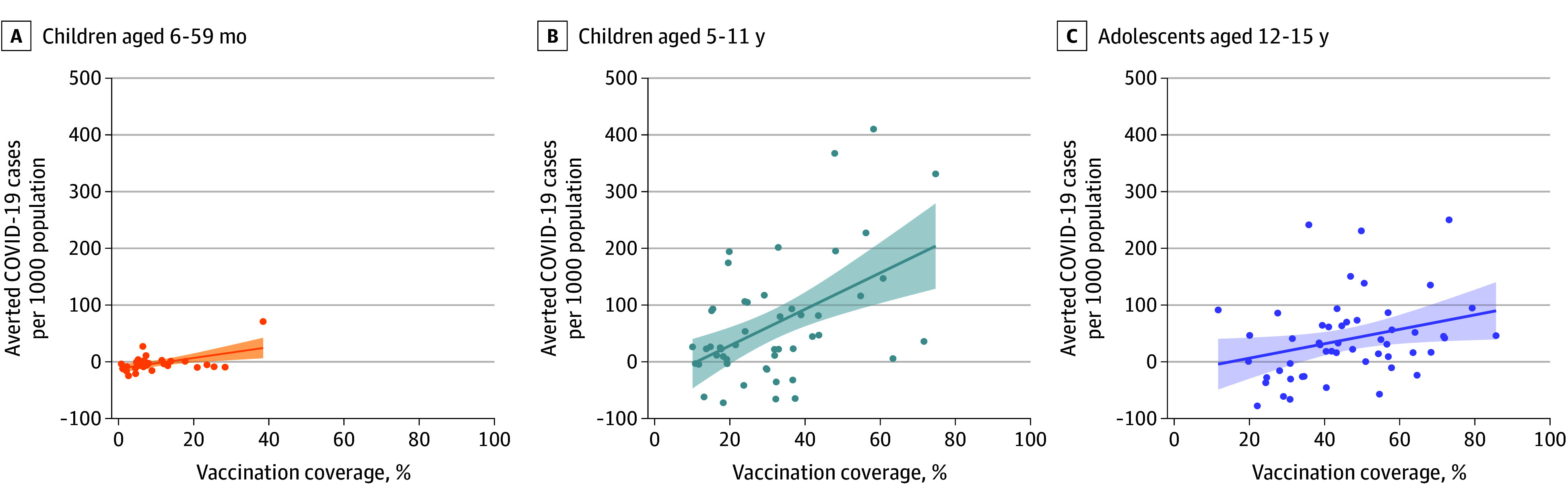
Pediatric COVID-19 Cases Averted by Pediatric County-Level Vaccine Coverage Association between estimated number of COVID-19 cases averted per 1000 population per county and the county-level of vaccination coverage for age groups 6 to 59 months (A), 5 to 11 years (B), and 12 to 15 years (C). Colored lines indicate line of best fit, and shaded regions represent the 95% CI.

County-level vaccination coverage explained 26% of variation of cases averted for children aged 6 to 59 months, 28% for children aged 5 and 11 years, and 12% for adolescents aged 12 to 15 years (Figure 4). On average, every increase of 10 vaccinations per 1000 children corresponded to a reduction of 0.9 (95% CI, 0.3-1.4) cases per 1000 children for individuals aged 6 to 59 months, 3.5 (95% CI, 1.9-5.1) cases per 1000 children for those aged 5 and 11 years, and 2.0 (95% CI, 0.6-3.4) cases per 1000 children for adolescents aged 12 to 15 years. Linear model fits had lower Akaike information criterion and bayesian information criterion values than segmented regression model fits for all age groups. Across all age groups, pediatric vaccination rates in California were generally highest among Bay Area counties (eFigure 8 in Supplement 1), which also ranked highest for averted cases due to vaccination (eResults in Supplement 1).

## Discussion

We provide evidence that California’s pediatric COVID-19 immunization program averted 376 085 (95% PI, 348 355-417 328) reported cases and 273 (95% PI, 77-605) hospitalizations among children aged 6 months to 15 years during the 4 to 7 months following vaccine availability. This represents a reduction of 26.3% of the number of cases that would have been seen in this population absent the vaccine. Prior work has similarly reported a high impact of widespread administration of mRNA vaccines in adult populations. In California, COVID-19 vaccines were estimated to avert more than 1.5 million cases, 72 000 hospitalizations, and 19 000 deaths statewide during the first 10 months of vaccination (through October 16, 2021).^17^ In the US, each 10% increase in vaccination coverage among individuals aged 18 years or older at the county level was associated with an 8% reduction in mortality and a 7% reduction in incidence.^18^ Similarly, a study in Israel estimated that nearly 650 000 cases of COVID-19 were averted in the first 2 months following vaccination introduction,^19^

Earlier studies have estimated vaccine effectiveness in pediatric populations by comparing incidence rates among vaccinated children with those in unvaccinated children using test-negative designs,^20,21,22^ or retrospective^23,24^ or prospective cohort studies.^25^ Our counterfactual case series approach, which has been used in other studies to estimate the population-level impact of interventions with a clearly specified rollout time,^26,27^ enables calculation of vaccine program impact at the population level, without information on individual vaccine status.

The cumulative effect of vaccination at the population level may be meaningful even if individual vaccine effectiveness is low. While influenza vaccine effectiveness was estimated at 29% in 2017-2018,^28^ it was estimated that widespread vaccination averted more than 3.1 million cases of influenza in the US.^29^ Nevertheless, overall impact depends on vaccine coverage. We identified positive associations between county-level vaccination coverage and averted cases in each age group, whereby each 10 additional vaccinations per 1000 children corresponded to an average reduction of 0.9 to 3.5 cases per 1000 children. Segmented regression models associating vaccine coverage with averted cases did not identify break points, suggesting that over the range of vaccination coverages examined (0%-85%), we saw neither diminishing returns on increased coverage owing to the acquisition of sufficient population-level immunity nor a threshold below which vaccination has limited public health impact. This is consistent with the persistence of SARS-CoV-2 circulation in populations with high vaccination coverage and resulting value of direct protection.

Results for individuals aged 6 to 59 months differed from those of older age groups in that we found a significant reduction in hospitalizations, but not cases, following vaccination. One explanation for this discrepancy could be that postvaccine evaluation period for children aged 6 to 59 months did not include a surge in COVID-19 cases as it did for the other age groups (Figure 2), potentially making it difficult to detect statistically significant reductions from the counterfactual. However, vaccine effectiveness of early mRNA vaccines was lower against Omicron variants compared with Alpha and Delta variants,^30,31^ and the Omicron variant dominated during the postvaccine period for children aged 6 months to 11 years (Figure 1). The detection of significant reductions in hospitalization in this age group, but not others, may be due, in part, to the fact that COVID-19 mortality disproportionately affects very young children compared with older children.^32^ For older age groups, we also estimated reductions in hospitalizations, although the 95% PI spans 0. However, we note that 95% CIs are narrower than PIs and may not have encompassed the null.

### Limitations

This study has limitations. Case data represented individuals who sought testing, which may be differential across unvaccinated and vaccinated groups, geographies, and time. Access to at-home testing likely resulted in further case underascertainment. If individuals were, on average, less likely to seek care for mild illness following vaccination, our analysis could have overestimated the absolute effect of the vaccine on cases averted. Overestimation of the relative effect of the vaccine may have resulted if vaccine recipients were disproportionately represented in the surveillance record both before and after vaccine eligibility compared with never-vaccinated individuals being more connected to care. Data on hospitalizations are less likely to be subjected to biases from differential case ascertainment. We estimated significant reductions in hospitalizations following vaccine introduction compared with counterfactual predictions.

Several considerations could lead to underestimates of the association between vaccination and child long-term health. First, asymptomatic cases are less likely to be reported, yet remain an important outcome, as post–COVID-19 condition symptoms may present after asymptomatic infections.^33,34,35^ Second, we were unable to estimate indirect outcomes associated with the vaccine in other age groups or control for social contacts. If children increased social contacts following receipt of the vaccine, as has been shown elsewhere,^36^ they may be challenged more frequently with SARS-CoV-2. Third, we assessed the outcomes of the vaccine over a short postvaccination period, limiting our ability to examine vaccine responses under waning immunity.

Two important limitations relate to model functional form. First, attributing differences between the observed and the predicted counterfactual cases to the vaccine assumes that the associations between incidence in the age group being modeled and incidence in the age groups selected as model predictors would, absent the vaccine, remain constant over the pre-to-post vaccine periods. This would not occur if one age group developed increased immunity or if different variants had differential age-disease associations. This is especially salient for the 5- to 11-year age group, as the models were primarily trained on data from the period when the Delta variant predominated, yet the Omicron variant, which is less reliant on angiotensin-converting enzyme 2 binding for entry^37^ and disproportionately influenced children younger than 5 years, prevailed in the evaluation period. Accordingly, the effect of vaccination may have been overestimated for this age group in counties where the incidence in children younger than 5 years was selected as a predictor (eTable 2 in the Supplement).

Second, there is potential for unstable predictions in the evaluation period if the predictive model was faced with values of selected predictors that fell outside the range of data used to fit the model. Our time series with a gap cross-validation approach guards against both of these limitations by prioritizing selection of generalized linear models that do well predicting values in periods that follow the training period, and in periods where the predictors may fall outside the range of what they were during the training period.^11,12,13,38^ Moreover, generalized linear models selected using different loss functions resulted in similar model predictions during the postevaluation period, suggesting that results are robust to differences in the nature of the association between incidence in the modeled age group and incidence in the predictor age groups.

## Conclusions

In this case series analysis of 3 913 063 pediatric cases, we provide evidence suggesting that programmatic vaccination against SARS-CoV-2 was associated with significant reductions in COVID-19 incidence among children in California in the 4 to 7 months following vaccine eligibility. At the county level, we found associations of higher vaccine coverage with greater reductions in pediatric cases. Our results support the use of COVID-19 vaccines to reduce COVID-19 incidence and hospitalization in pediatric populations.

## Notes

Supplement 1. **eMethods.** Detailed Methods **eResults.** Detailed Results
eReferences
**eTable 1.** Predictors Eligible for Selection in Candidate Models for Each Age Group and Outcome Combination **eTable 2.** Best Predictive Models Selected for Each County-Age Group Combination Using Incidence as Outcome **eTable 3.** Best Predictive Models Selected for Each Region-Age Group Combination Using Hospitalization as Outcome **eTable 4.** Statewide Estimates of Averted COVID-19 Cases Due to Vaccination Among Children Aged 6 Months to 15 Years of Age Using Mean Absolute Error as the Loss Function **eTable 5.** Statewide Estimates of Averted COVID-19 Hospitalizations Due to Vaccination Among Children Aged 6 Months to 15 Years of Age Using Mean Absolute Error as the Loss Function **eTable 6.** Estimated Number of COVID-19 Cases Averted by County and Age Group During the Post-Vaccination Period **eTable 7.** Estimated Number of COVID-19 Hospitalizations Averted by Region and Age Group During the Post-Vaccination Period **eFigure 1.** Map of the Five Regions Used for Estimating the Effect of Pediatric SARS-CoV-2 Vaccines on Childhood Hospitalizations for COVID-19 **eFigure 2.** Schematic of the Time Series With Gap Cross-Validation Algorithm Used to Select the Best Predictive Algorithm During the Pre-Period **eFigure 3.** Model Performance of Selected Generalized Linear Model Predicting Cases During the Pre-Vaccine Period for Each County-Age Group Combination **eFigure 4.** Model Performance of Selected Generalized Linear Model Predicting Hospitalizations During the Pre-Vaccine Period for Each Region-Age Group Combination **eFigure 5.** Jackknife Analysis for Children 12-15 Years **eFigure 6.** Jackknife Analysis for Children 5-11 Years **eFigure 7.** Jackknife Analysis for Children 6-59 Months **eFigure 8.** Maps of Pediatric COVID-19 Cases Averted and County-Level Pediatric Vaccination Coverage in California. **eFigure 9.** County-Specific Model Predictions for COVID-19 Cases Among Children Aged 12-15 Years **eFigure 10.** County-Specific Model Predictions for COVID-19 Cases Among Children Aged 5-11 Years **eFigure 11.** County-Specific Model Predictions for COVID-19 Cases Among Children Aged 6-59 Months **eFigure 12.** Region-Specific Model Predictions for COVID-19 Hospitalizations Among Children Aged 12-15 Years **eFigure 13.** Region-Specific Model Predictions for COVID-19 Hospitalizations Among Children Aged 5-11 Years **eFigure 14.** Region-Specific Model Predictions for COVID-19 Hospitalizations Among Children Aged 6-59 Months

Supplement 2.
Data Sharing Statement

## References

[1] Ehreth J. The global value of vaccination. Vaccine. 2003;21(7-8):596-600. doi:10.1016/S0264-410X(02)00623-0 12531324

[2] HHS.gov. US Department of Health and Human Services. COVID-19 vaccines. Accessed April 5, 2023. https://www.hhs.gov/coronavirus/covid-19-vaccines/index.html

[3] Watanabe A, Kani R, Iwagami M, Takagi H, Yasuhara J, Kuno T. Assessment of efficacy and safety of mRNA COVID-19 vaccines in children aged 5 to 11 years: a systematic review and meta-analysis. JAMA Pediatr. 2023;177(4):384-394. doi:10.1001/jamapediatrics.2022.6243 36689319 PMC9871947

[4] Ruiz JB, Bell RA. Parental COVID-19 vaccine hesitancy in the United States. Public Health Rep. 2022;137(6):1162-1169. doi:10.1177/00333549221114346 35915993 PMC9574308

[5] Gray A, Fisher CB. Determinants of COVID-19 vaccine uptake in adolescents 12-17 years old: examining pediatric vaccine hesitancy among racially diverse parents in the United States. Front Public Health. 2022;10:844310. doi:10.3389/fpubh.2022.844310 35392471 PMC8980347

[6] Fisher CB, Bragard E, Jaber R, Gray A. COVID-19 vaccine hesitancy among parents of children under five years in the United States. Vaccines (Basel). 2022;10(8):1313. doi:10.3390/vaccines10081313 36016200 PMC9413913

[7] Lambert D. California ends plans for kids’ COVID vaccine mandate. EdSource. February 1, 2023. Accessed April 5, 2023. https://edsource.org/2023/california-ends-plans-for-kids-covid-vaccine-mandate

[8] California Department of Public Health. Vaccination status by age and race and ethnicity. Accessed April 5, 2023. https://covid19.ca.gov/vaccination-progress-data/#age-ethnicity

[9] Guimarães D, Pissarra R, Reis-Melo A, Guimarães H. Multisystem inflammatory syndrome in children (MISC): a systematic review. Int J Clin Pract. 2021;75(11):e14450. doi:10.1111/ijcp.14450 34105843

[10] Jin R. The lag between daily reported Covid-19 cases and deaths and its relationship to age. J Public Health Res. 2021;10(3):2049. doi:10.4081/jphr.2021.2049 33709641 PMC8431868

[11] Bergmeir C, Benítez JM. On the use of cross-validation for time series predictor evaluation. Inf Sci. 2012;191:192-213. doi:10.1016/j.ins.2011.12.028

[12] Arlot S, Celisse A. A survey of cross-validation procedures for model selection. Statist Surv. 2010;4:40-179. doi:10.1214/09-SS054

[13] Bergmeir C, Hyndman RJ, Koo B. A note on the validity of cross-validation for evaluating autoregressive time series prediction. Comput Stat Data Anal. 2018;120:70-83. doi:10.1016/j.csda.2017.11.003

[14] Liu W. Prediction intervals for Poisson regression. Accessed May 10, 2023. https://statcompute.wordpress.com/2015/12/20/prediction-intervals-for-poisson-regression/

[15] Muggeo VM. Estimating regression models with unknown break-points. Stat Med. 2003;22(19):3055-3071. doi:10.1002/sim.1545 12973787

[16] R: A language and environment for statistical computing. R Foundation for Statistical Computing; 2015. http://www.R-project.org/

[17] Tan ST, Park HJ, Rodríguez-Barraquer I, . COVID-19 vaccination and estimated public health impact in California. JAMA Netw Open. 2022;5(4):e228526-e228526. doi:10.1001/jamanetworkopen.2022.8526 35452106 PMC9034409

[18] Suthar AB, Wang J, Seffren V, Wiegand RE, Griffing S, Zell E. Public health impact of covid-19 vaccines in the US: observational study. BMJ. 2022;377:e069317. doi:10.1136/bmj-2021-069317 35477670 PMC9044401

[19] Somekh I, KhudaBukhsh WR, Root ED, . Quantifying the population-level effect of the COVID-19 mass vaccination campaign in Israel: a modeling study. Open Forum Infect Dis. 2022;9(5):ofac087. doi:10.1093/ofid/ofac087 35493128 PMC9043004

[20] Khan FL, Nguyen JL, Singh TG, . Estimated BNT162b2 vaccine effectiveness against infection with Delta and Omicron variants among US children 5 to 11 years of age. JAMA Netw Open. 2022;5(12):e2246915-e2246915. doi:10.1001/jamanetworkopen.2022.46915 36515946 PMC9856252

[21] Fleming-Dutra KE, Britton A, Shang N, . Association of prior BNT162b2 COVID-19 vaccination with symptomatic SARS-CoV-2 infection in children and adolescents during Omicron predominance. JAMA. 2022;327(22):2210-2219. doi:10.1001/jama.2022.7493 35560036 PMC9107063

[22] Klein NP, Stockwell MS, Demarco M, . Effectiveness of COVID-19 Pfizer-BioNTech BNT162b2 mRNA vaccination in preventing COVID-19-associated emergency department and urgent care encounters and hospitalizations among nonimmunocompromised children and adolescents aged 5-17 years—VISION Network, 10 states, April 2021-January 2022. MMWR Morb Mortal Wkly Rep. 2022;71(9):352-358. doi:10.15585/mmwr.mm7109e3 35239634 PMC8893336

[23] Sacco C, Del Manso M, Mateo-Urdiales A, ; Italian National COVID-19 Integrated Surveillance System and the Italian COVID-19 vaccines registry. Effectiveness of BNT162b2 vaccine against SARS-CoV-2 infection and severe COVID-19 in children aged 5-11 years in Italy: a retrospective analysis of January-April, 2022. Lancet. 2022;400(10346):97-103. doi:10.1016/S0140-6736(22)01185-0 35780801 PMC9246475

[24] Cohen-Stavi CJ, Magen O, Barda N, . BNT162b2 vaccine effectiveness against Omicron in children 5 to 11 years of age. N Engl J Med. 2022;387(3):227-236. doi:10.1056/NEJMoa2205011 35767475 PMC9258754

[25] Fowlkes AL, Yoon SK, Lutrick K, . Effectiveness of 2-dose BNT162b2 (Pfizer BioNTech) mRNA vaccine in preventing SARS-CoV-2 infection among children aged 5-11 years and adolescents aged 12-15 years—PROTECT cohort, July 2021-February 2022. MMWR Morb Mortal Wkly Rep. 2022;71(11):422-428. doi:10.15585/mmwr.mm7111e1 35298453 PMC8942308

[26] Head JR, Collender PA, Lewnard JA, . Early evidence of inactivated enterovirus 71 vaccine impact against hand, foot, and mouth disease in a major center of ongoing transmission in China, 2011-2018: a longitudinal surveillance study. Clin Infect Dis. 2020;71(12):3088-3095. doi:10.1093/cid/ciz1188 31879754 PMC7819528

[27] Baker JM, Tate JE, Steiner CA, Haber MJ, Parashar UD, Lopman BA. Longer-term direct and indirect effects of infant rotavirus vaccination across all ages in the US; 2000-2013: analysis of a large hospital discharge dataset. Clin Infect Dis. 2018:ciy580-ciy580. 30020438 10.1093/cid/ciy580 PMC7182126

[28] Centers for Disease Control and Prevention. Past seasons’ vaccine effectiveness estimates: influenza (flu). Accessed April 4, 2023. https://www.cdc.gov/flu/vaccines-work/past-seasons-estimates.html

[29] Centers for Disease Control and Prevention (CDC). 2018-2019 estimated flu illnesses, medical visits, hospitalizations, and deaths prevented by vaccination. Accessed April 4, 2023. https://archive.cdc.gov/#/details?url=https://www.cdc.gov/flu/about/burden-averted/2018-2019.htm

[30] Willett BJ, Grove J, MacLean OA, ; PITCH Consortium; COVID-19 Genomics UK (COG-UK) Consortium. SARS-CoV-2 Omicron is an immune escape variant with an altered cell entry pathway. Nat Microbiol. 2022;7(8):1161-1179. doi:10.1038/s41564-022-01143-7 35798890 PMC9352574

[31] Buchan SA, Chung H, Brown KA, . Estimated effectiveness of COVID-19 vaccines against Omicron or Delta symptomatic infection and severe outcomes. JAMA Netw Open. 2022;5(9):e2232760-e2232760. doi:10.1001/jamanetworkopen.2022.32760 36136332 PMC9500552

[32] Havers F. Epidemiology of COVID-19 in children aged 5-11 years. October 26, 2021. Accessed June 20, 2023. https://www.fda.gov/media/153508/download

[33] Buonsenso D, Munblit D, De Rose C, . Preliminary evidence on long COVID in children. Acta Paediatr. 2021;110(7):2208-2211. doi:10.1111/apa.15870 33835507 PMC8251440

[34] Wise J. Long covid: One in seven children may still have symptoms 15 weeks after infection, data show. BMJ. 2021;374(2157):n2157. doi:10.1136/bmj.n2157 34470745

[35] Stephenson T, Pinto Pereira SM, Shafran R, ; CLoCk Consortium. Physical and mental health 3 months after SARS-CoV-2 infection (long covid) among adolescents in England (CLoCk): a national matched cohort study. Lancet Child Adolesc Health. 2022;6(4):230-239. doi:10.1016/S2352-4642(22)00022-0 35143770 PMC8820961

[36] Andrejko KL, Head JR, Lewnard JA, Remais JV. Longitudinal social contacts among school-aged children during the COVID-19 pandemic: the Bay Area Contacts among Kids (BACK) study. BMC Infect Dis. 2022;22(1):242. doi:10.1186/s12879-022-07218-4 35272626 PMC8907906

[37] Lewnard JA, Hong VX, Patel MM, Kahn R, Lipsitch M, Tartof SY. Clinical outcomes associated with SARS-CoV-2 Omicron (B.1.1.529) variant and BA.1/BA.1.1 or BA.2 subvariant infection in Southern California. Nat Med. 2022;28(9):1933-1943. doi:10.1038/s41591-022-01887-z 35675841 PMC10208005

[38] Cerqueira V, Torgo L, Mozetič I. Evaluating time series forecasting models: an empirical study on performance estimation methods. Mach Learn. 2020;109(11):1997-2028. doi:10.1007/s10994-020-05910-7

