# Supplementary material for: COVID-19 Vaccination and Incidence of Pediatric SARS-CoV-2 Infection and Hospitalization
Source: JAMA Netw Open. 2024 Apr 23;7(4):e247822. doi: 10.1001/jamanetworkopen.2024.7822 (PMC11040406; doi:10.1001/jamanetworkopen.2024.7822)
Supplement: Supplement 1. — eMethods. Detailed Methods eResults. Detailed Results eReferences eTable 1. Predictors Eligible for Selection in Candidate Models for Each Age Group and Outcome Combination eTable 2. Best Predictive Models Selected for Each County-Age Group Combination Using Incidence as Outcome eTable 3. Best Predictive Models Selected for Each Region-Age Group Combination Using Hospitalization as Outcome eTable 4. Statewide Estimates of Averted COVID-19 Cases Due to Vaccination Among Children Aged 6 Months to 15 Years of Age Using Mean Absolute Error as the Loss Function eTable 5. Statewide Estimates of Averted COVID-19 Hospitalizations Due to Vaccination Among Children Aged 6 Months to 15 Years of Age Using Mean Absolute Error as the Loss Function eTable 6. Estimated Number of COVID-19 Cases Averted by County and Age Group During the Post-Vaccination Period eTable 7. Estimated Number of COVID-19 Hospitalizations Averted by Region and Age Group During the Post-Vaccination Period eFigure 1. Map of the Five Regions Used for Estimating the Effect of Pediatric SARS-CoV-2 Vaccines on Childhood Hospitalizations for COVID-19 eFigure 2. Schematic of the Time Series With Gap Cross-Validation Algorithm Used to Select the Best Predictive Algorithm During the Pre-Period eFigure 3. Model Performance of Selected Generalized Linear Model Predicting Cases During the Pre-Vaccine Period for Each County-Age Group Combination eFigure 4. Model Performance of Selected Generalized Linear Model Predicting Hospitalizations During the Pre-Vaccine Period for Each Region-Age Group Combination eFigure 5. Jackknife Analysis for Children 12-15 Years eFigure 6. Jackknife Analysis for Children 5-11 Years eFigure 7. Jackknife Analysis for Children 6-59 Months eFigure 8. Maps of Pediatric COVID-19 Cases Averted and County-Level Pediatric Vaccination Coverage in California. eFigure 9. County-Specific Model Predictions for COVID-19 Cases Among Children Aged 12-15 Years eFigure 10. County-Specific Model Predictions fo [file jamanetwopen-e247822-s001.pdf]

Supplementary Online Content

Head JR, Collender PA, León TM, et al. COVID-19 vaccination and incidence of pediatric SARS-CoV-2 infection and hospitalization. *JAMA Netw Open*. 2024;7(4):e247822. doi:10.1001/jamanetworkopen.2024.7822

eMethods. Detailed Methods

eResults. Detailed Results

eReferences

eTable 1. Predictors Eligible for Selection in Candidate Models for Each Age Group and Outcome Combination

eTable 2. Best Predictive Models Selected for Each County-Age Group Combination Using Incidence as Outcome

eTable 3. Best Predictive Models Selected for Each Region-Age Group Combination Using Hospitalization as Outcome

eTable 4. Statewide Estimates of Averted COVID-19 Cases Due to Vaccination Among Children Aged 6 Months to 15 Years of Age Using Mean Absolute Error as the Loss Function

eTable 5. Statewide Estimates of Averted COVID-19 Hospitalizations Due to Vaccination Among Children Aged 6 Months to 15 Years of Age Using Mean Absolute Error as the Loss Function

eTable 6. Estimated Number of COVID-19 Cases Averted by County and Age Group During the Post-Vaccination Period

eTable 7. Estimated Number of COVID-19 Hospitalizations Averted by Region and Age Group During the Post-Vaccination Period

eFigure 1. Map of the Five Regions Used for Estimating the Effect of Pediatric SARS-CoV-2 Vaccines on Childhood Hospitalizations for COVID-19

eFigure 2. Schematic of the Time Series With Gap Cross-Validation Algorithm Used to Select the Best Predictive Algorithm During the Pre-Period

eFigure 3. Model Performance of Selected Generalized Linear Model Predicting Cases During the Pre-Vaccine Period for Each County-Age Group Combination

eFigure 4. Model Performance of Selected Generalized Linear Model Predicting Hospitalizations During the Pre-Vaccine Period for Each Region-Age Group Combination

eFigure 5. Jackknife Analysis for Children 12-15 Years

eFigure 6. Jackknife Analysis for Children 5-11 Years

eFigure 7. Jackknife Analysis for Children 6-59 Months

eFigure 8. Maps of Pediatric COVID-19 Cases Averted and County-Level Pediatric Vaccination Coverage in California.

eFigure 9. County-Specific Model Predictions for COVID-19 Cases Among Children Aged 12-15 Years

eFigure 10. County-Specific Model Predictions for COVID-19 Cases Among Children Aged 5-11 Years

eFigure 11. County-Specific Model Predictions for COVID-19 Cases Among Children Aged 6-59 Months

eFigure 12. Region-Specific Model Predictions for COVID-19 Hospitalizations Among Children Aged 12-15 Years

eFigure 13. Region-Specific Model Predictions for COVID-19 Hospitalizations Among Children Aged 5-11 Years

eFigure 14. Region-Specific Model Predictions for COVID-19 Hospitalizations Among Children Aged 6-59 Months

This supplementary material has been provided by the authors to give readers additional information about their work.

© 2024 Head JR et al. *JAMA Network Open.*

eMethods. Detailed Methods

Epidemiologic and Covariate Data

To determine the number of cases among individuals aged 16 years and older, we first obtained information on the daily number of all COVID-19 cases reported by county from USAFacts (1). We then subtracted the number of cases of individuals younger than 16 years from the total case count to arrive at the daily case count of COVID-19 cases among people aged 16 years and older.

Information on when vaccines were approved for select age groups was obtained from (2) (Figure 1 lower panel). We obtained information on the number of vaccines offered per county and age group from California Open data portal (3). The data portal draws information from the California Immunization Registry (CAIR) (4). San Diego and some of the central California counties have their own registries which are then merged with CAIR. This dataset contains daily county-level information on the number of individuals per vaccine age group that had received at least one vaccine, the number of individuals per age group that received a full vaccine series, and the population in each age group. We calculated the proportion of individuals vaccinated by dividing the total counts per age group by the population of that age group. Some counties did not vaccine information and were dropped from analysis. We obtained information on vaccination for 31 counties in the 6-59 month age group, 52 counties in the 5-11 year old age group, and 56 counties in the 12-15 year old age group.

© 2024 Head JR et al. *JAMA Network Open.*

Information on when schools were in session vs. not was estimated based on academic calendars from four major school districts (5-8), which tended to lie in the middle range of the span of possible opening and closing dates. In California, schools are required to open no later than August 31, with the majority beginning in August, and let out for the summer no later than June 15. Our use of the weekly rolling average additionally helps average over weekly effects of school attendance.

Candidate model generation

We developed a set of candidate predictive quasi-Poisson generalized linear models (GLMs). Candidate models were constructed by first identifying the potential predictors eligible for inclusion in models. These predictors are summarized in eTable 1 in the Supplement. We then generated candidate models that included all possible combinations of those predictors. Not including a specific predictor was also included among the potential combinations. In this way, candidate models ranged from being very parsimonious (e.g., intercept-only model) to very complex (all possible predictors included).

Predictors eligible for selection included: 1) log-incidence series for other age groups, included as either covariates or as an offset term for any one age group other than the one being modeled (see below); 2) a binary indicator of vaccine age-eligibility for other age groups; 3) a binary indicator for in-person school being in session; 4) interactions between school and vaccine introduction indicator variables and time series for other age groups, aiming to account for differences in constant proportionality during school periods or when one age group

© 2024 Head JR et al. *JAMA Network Open.*

became vaccinated; 5) seasonal controls. While seasonality of SARS-CoV-2 is yet to be resolved (9), we included seasonal controls as potential predictors to account for observed winter and summer surges in incidence or potential co-occurrence with other seasonal respiratory infections (10). Seasonality was controlled for using harmonic terms with a period of 12 months. Models defined 7-day rolling average case counts within each county and age group as the outcome variable.

We used two frameworks to account for possible differences in the nature of the relationship between the log incidence series being modeled and the log incidence series being used as predictors. In one framework, log incidence of other age groups were used as model predictors, and in the other, a log incidence of a single age group was used as a model offset. Models where the logged time series of non-vaccine age groups are selected as predictors assumes that the times series of interest is related to other time series exponentially, as shown:

$$\log\big(E(Y_{a=A,it}|\boldsymbol{\beta}, Y_{a\neq A,it})\big) = \beta_0 + \beta_1 \log\big(Y_{a\neq A,it}\big)$$

$$E(Y_{a=A,it}|\boldsymbol{\beta}, Y_{a\neq A,it}) = \exp\big(\beta_0 + \beta_1\, log\big(Y_{a\neq A,it}\big)\big) = \exp(\beta_0) \times \exp\big(\beta_1\, log\big(Y_{a\neq A,it}\big)\big)$$

$$= \exp(\beta_0) \times \exp\big(log\big(Y_{a\neq A,it}\big)\big)^{\beta_1} = \exp(\beta_0) \times {Y_{a\neq A,it}}^{\beta_1}$$

$$E(Y_{a=A,it}|\boldsymbol{\beta}, Y_{a\neq A,it}) = \mathbf{exp}(\boldsymbol{\beta_0}) \times {\boldsymbol{Y_{a\neq A,it}}}^{\boldsymbol{\beta_1}}$$

Where:

$Y_{a=A,it}$ is incidence of COVID-19 in age group (a) of interest ($a = A$), county i, and time t,

$Y_{a\neq A,it}$ is incidence of COVID-19 in another age group ($a \neq A$), county i, and time t

β_0 is the model intercept

β_1 is a coefficient on the predictor of interest

Models where a single logged time series of a non-vaccine age group is selected as an offset assumes that the time series of interest is related to other time series by a simple multiplier, according to the equation below:

$$\log\left(E(Y_{a=A,it}|\boldsymbol{\beta}, Y_{a\neq A,it})\right) = \beta_0 + \text{offset}\left(\log\left(Y_{a\neq A,it}\right)\right)$$

$$E(Y_{a=A,it}|\boldsymbol{\beta}, Y_{a\neq A,it}) = \exp\left(\beta_0 + log\left(Y_{a\neq A,it}\right)\right) = \exp(\beta_0) \times \exp\left(log\left(Y_{a\neq A,it}\right)\right)$$
$$= \exp(\beta_0) \times Y_{a\neq A,it}$$

$$E(Y_{a=A,it}|\boldsymbol{\beta}, Y_{a\neq A,it}) = \mathbf{exp}(\boldsymbol{\beta_0}) \times \boldsymbol{Y_{a\neq A,it}}$$

Candidate models for hospitalizations included all possible combinations of terms for: 1) seasonal controls (harmonic terms); 2) an indicator for in-person school being in session; 3) indicator variables for vaccine introduction within any age group that became eligible before the group being modeled; 4) log weekly case incidence series for other age groups, lagged by two weeks in accordance with the expected lag between infection and hospitalization (11); 5) unlagged weekly hospitalizations across other age groups; 6) interactions between school and vaccine introduction indicator variables and time series for other age groups. Models were

© 2024 Head JR et al. *JAMA Network Open.*

developed separately for each of California Department of Public Health's five designated regions (shown in eFigure 1) using weekly hospitalizations per age group as the outcome.

Model selection and fitting

We used a time series cross validation approach to select the best predictive model for each age group and geographical area (county or region) within the pre-vaccine period (as depicted in eFigure 2) (12-14). For this approach, in the first fold, the training period was the first month of data followed by a one-month gap and a two-month test period. Each successive fold added one additional month to the training period and shifted the one-month gap and two-month hold-out one month later. The one-month gap between the training and the test periods helps increase independence between data used to train and test the models and mirrors the gap we imposed between pre- and post-vaccine periods (15).

Calculation of vaccine impact and relationship between averted cases and vaccination coverage

We used selected models fit to pre-vaccine data to predict counterfactual incidence and hospitalization in the post-vaccine period, reflecting the expected case or hospitalization count had vaccination not occurred. For inference, we computed 95% prediction intervals (PI) around the pre-vaccine fitted values and post-vaccine counterfactual predictions using a two-step bootstrap simulation process that allows us to accommodate uncertainty related to prediction errors (16). To compute the prediction intervals, we first drew 1,000 MonteCarlo simulations for each time point from a quasiPoisson distribution using the predint package in R (17). This package allows generation of random samples from a Poisson distribution centered at a mean,

© 2024 Head JR et al. *JAMA Network Open.*

lambda, but with inclusion of a dispersion factor to allow for the variance to be greater than lambda (e.g., overdispersion). This is akin to using the sandwich package in R to obtain standard errors that account for overdispersion. Random samples were centered at the model-based prediction. We then refitted the models using the newly drawn samples and generated predictions from these new fitted models. We drew a second round of 1,000 MonteCarlo simulations with lambda equal to the predicted values from these models. We computed the 2.5th and the 97.5th percentile of all simulated samples to arrive at the 95% prediction interval for each time point. When the dispersion factor from the best predictive model was less than one, we drew samples from a Poisson distribution rather than the quasiPoisson distribution. Statewide estimates were obtained by summing predictions across county, and the 95% PI for the statewide estimate was calculated by summing all sampled values and computing the 2.5th and 97.5th percentile.

We estimated the vaccine impact as the difference between predicted and observed values during the post-vaccine evaluation period. For each county or region, we calculated both the absolute number of cases and hospitalizations averted, respectively, as well as the percent reduction from expected burden, over the full evaluation period. To understand the relationship between vaccination coverage and averted cases, we fit regression models relating the reduction in cases within each age and per county to county-level vaccination coverage within the same age group at the end of the post-vaccine evaluation period, using a fixed effects meta-analysis with weights equal to the inverse estimated standard error of the estimates per county. We also used segmented regression models to examine whether break

© 2024 Head JR et al. *JAMA Network Open.*

points occurred in the relationship between vaccination coverage and averted cases per capita. Segmented regression models are piecewise linear models, where the independent variable (vaccination coverage) is partitioned into intervals, with separate lines fit to each segment (18). The algorithm used applies a maximum likelihood approach to iteratively identify best fitting break points, or absence of break points (18). By allowing for the linear relationship between coverage and averted cases to change across the observed range of vaccination coverages, we were able to ask if there were coverages below which reductions in cases could not be identified (i.e., below some critical threshold for meaningful effect) or above which diminishing returns on vaccination were observed (i.e., above a threshold needed to achieve sufficient population-level immunity).

© 2024 Head JR et al. *JAMA Network Open.*

eResults. Detailed Results

Fit of selected models

Median r^2 values for models fit to daily case data were 0.92 for children 6-59 months (interquartile range (IQR): 0.79 – 0.96), 0.89 for children 5-11 years (IQR: 0.78 – 0.95), and 0.79 for adolescents 12-15 years (IQR: 0.62 – 0.90) (eFigure 3; see eFigure 4 for hospitalizations). Models with the lowest r^2 values were from sparsely populated counties (eFigure 3). Roughly half (43%, 45%, and 55% for ages 6-59 months, 5-11 years, and 12-15 years, respectively) of the best predictive models for daily cases included an offset of a single age group time series (see eTable 2 for selected models).

Results using mean absolute error (MAE) as the loss function

Children 12-15 years

Statewide estimates for averted cases and hospitalizations in children aged 12-15 years were similar when model selection was done using MAE as the loss function. Estimates are included in eTable 5 for cases and eTable 6 for hospitalizations.

Children 5-11 years

Statewide estimates were lower when model selection was done using MAE (estimated averted cases: 175,548, 95% CI: 146,862 – 937,506), but prediction intervals overlapped and estimated averted cases remained significant (eTable 5). Statewide hospitalizations were similar when model selection was done using MAE as the loss function. Estimates are included in eTable 5 for cases and eTable 6 for hospitalizations.

© 2024 Head JR et al. *JAMA Network Open.*

Children 6-59 months

Statewide estimates for cases were lower when model selection was done using MAE (eTable 5), but estimates for hospitalizations were similar between the two loss functions (eTable 6).

Jack-knife analyses

In our jack-knife analyses, results for individuals aged 5-11 years and 12-15 years were not sensitive to the inclusion of any single county. This is depicted in eFigure 5 for ages 5-11 years and eFigure for ages 12-15 years. For both age groups, the removal of Contra Costa or Santa Clara County (two counties from the Bay Area with high vaccination coverage) resulted in somewhat smaller estimated reductions following vaccinations, although the confidence intervals overlapped with statewide estimates. Results for children aged 6-59 months were sensitive to the inclusion of Los Angeles, as depicted in eFigure 7. Los Angeles represents the largest county in the state accounting for 23.9% of residents as well as 20.7% of cases and 39.2% of hospitalizations analyzed for this age group. Beyond being the largest county, Los Angeles also maintains separate COVID-19 data systems that get merged with the rest of the state.

© 2024 Head JR et al. *JAMA Network Open.*

eReferences

1. USAFACTS. Coronavirus Locations: COVID-19 Map by County and State. 2021 [Available from: https://usafacts.org/visualizations/coronavirus-covid-19-spread-map/.
2. U.S. Department of Health and Human Services. COVID-19 Vaccines Washington DC: HHS.gov; 2022 [Available from: https://www.hhs.gov/coronavirus/covid-19-vaccines/index.html.
3. CalHHS. COVID-19 Vaccine Progress Dashboard Data. Sacramento2023.
4. California Department of Public Health. CALIFORNIA IMMUNIZATION REGISTRY (CAIR) Sacramento, CA: CDPH; 2023 [Available from: https://www.cdph.ca.gov/Programs/CID/DCDC/CAIR/Pages/CAIR-updates.aspx.
5. Los Angeles Unified School District. School Calendars Los Angeles: LAUSD; 2023 [Available from: https://achieve.lausd.net/Page/2#calendar78377/20230510/month.
6. District SFUS. SFUSD Calendars San Francisco: SFUSD; 2023, [Available from: https://www.sfusd.edu/calendars.
7. Oakland Unified School District. District Calendar 2023 [Available from: https://www.ousd.org/about-us/districtcalendar.
8. San Diego Unified School District. Academic Calendars 2023 [Available from: https://www.sandiegounified.org/academics/academic_calendars.
9. Murray CJL, Piot P. The Potential Future of the COVID-19 Pandemic: Will SARS-CoV-2 Become a Recurrent Seasonal Infection? Jama. 2021;325(13):1249-50.
10. Adams K, Tastad KJ, Huang S, Ujamaa D, Kniss K, Cummings C, et al. Prevalence of SARS-CoV-2 and Influenza Coinfection and Clinical Characteristics Among Children and Adolescents Aged< 18 Years Who Were Hospitalized or Died with Influenza—United States, 2021–22 Influenza Season. Morbidity and Mortality Weekly Report. 2022;71(50):1589.
11. Jin R. The lag between daily reported Covid-19 cases and deaths and its relationship to age. J Public Health Res. 2021;10(3).
12. Bergmeir C, Benítez JM. On the use of cross-validation for time series predictor evaluation. Information Sciences. 2012;191:192-213.
13. Arlot S, Celisse A. A survey of cross-validation procedures for model selection. 2010.
14. Bergmeir C, Hyndman RJ, Koo B. A note on the validity of cross-validation for evaluating autoregressive time series prediction. Computational Statistics & Data Analysis. 2018;120:70-83.
15. Cerqueira V, Torgo L, Mozetič I. Evaluating time series forecasting models: an empirical study on performance estimation methods. Machine Learning. 2020;109(11):1997-2028.
16. Liu W. Prediction Intervals for Poisson Regression 2016 [Available from: https://statcompute.wordpress.com/2015/12/20/prediction-intervals-for-poisson-regression/.
17. Menssen M. preint: Prediction Intervals. 2023.
18. Muggeo VM. Estimating regression models with unknown break-points. Statistics in medicine. 2003;22(19):3055-71.

eTable 1. Predictors eligible for selection in candidate models for each age group and outcome combination. Blue shaded boxes indicate that the predictor was eligible to be included as a main effect only, without interaction with other terms. Orange colored boxes indicate the predictor was eligible to be included within an interaction term between one or more relevant indicator variables (e.g., the indicator for in school and the indicator for beings past the age-eligibility for vaccination of children over 5 years could interact with the time series of children aged 5-11 years.) Any eligible offset term was not used in conjunction with another offset term (e.g., if incidence in children aged 6-59 months was chosen as an included offset term, incidence in no other age group could be included as a predictor in the models)

	**Ages 6-59 months**		**Ages 5-11 years**		**Ages 12-15 years**	
**Variable description (variable name, used in eTable 2 or eTable 3)**	**Cases**	**Hospital-izations**	**Cases**	**Hospital-izations**	**Cases**	**Hospital-izations**
Harmonic terms on month (sin_month, cos_month)						
Indicator variable for in school (inschool)						
Indicator variable for date past approval of vaccine for individuals 16 years or older (postvacc16)						
Indicator variable for date past approval of vaccine for individuals 12 years or older (postvacc12)						
Indicator variable for date past approval of vaccine for individuals 5 years or older (postvacc5)						
Logged daily case time series of adults (logcases_adult)						
Logged daily case time series of children 12-15 years (logcases_12_15)						
Logged daily case time series of children 5-11 years (logcases_5_11)						
Logged daily case time series of children 6-59 months (logcases_6_59m)						
Logged daily case time series of children under 6 months (logcases_less6m)						
Offset of logged daily case time series of adults						
Offset of logged daily case time series of children 12-15 years						
Offset of logged daily case time series of children 5-11 years						
Offset of logged daily case time series of children 6-59 months						
Offset of logged daily case time series of children under 6 months						
Logged daily case time series of adults, lagged by 2 weeks (loglag2cases_adults)						
Logged daily case time series of children 12-15 years, lagged by 2 weeks (loglag2cases_12_15)						
Logged daily case time series of children 5-11 years, lagged by 2 weeks (loglag2cases_5_11)						
Logged daily case time series of children 6-59 months, lagged by 2 weeks (loglag2cases_5_69m)						
Logged daily case time series of children under 6 months, lagged by 2 weeks (loglag2cases_less6m)						
Logged weekly hospitalization time series of adults (loghosp_adults)						
Logged weekly hospitalization time series of children 12-15 years (loghosp_12_15)						
Logged weekly hospitalization time series of children 5-11 years (loghosp_5_11)						
Logged weekly hospitalization time series of children 6-59 months (loghosp_6_59m)						
Logged weekly hospitalization time series of children under 6 months (loghosp_less6m)						

© 2024 Head JR et al. *JAMA Network Open.*

eTable 2. Best predictive models selected for each county-age group combination using incidence as outcome. Description for variable names are given in eTable 1.

County	Age	Selected Model According to Mean Square Error	Selected Model According to Mean Absolute Error
Alameda	6-59m	Y ~ postvacc16+logcases_5_11+logcases_less6m	Y ~ logcases_12_15
Alpine	6-59m	Y ~ postvacc5+logcases_adult+logcases_5_11+logcases_12_15	Y ~ inschool+postvacc5+logcases_adult+postvacc16+logcases_12_15+logcases_adult:postvacc16
Amador	6-59m	Y ~ offset(logcases_5_11)	Y ~ inschool+postvacc16+logcases_5_11+logcases_12_15*postvacc12+logcases_less6m
Butte	6-59m	Y ~ inschool+postvacc5+postvacc12+postvacc16+logcases_adult+logcases_5_11	Y ~ postvacc5+postvacc16+logcases_adult+logcases_5_11+logcases_12_15
Calaveras	6-59m	Y ~ logcases_adult+logcases_12_15	Y ~ postvacc12+logcases_adult+logcases_12_15
Colusa	6-59m	Y ~ postvacc12+offset(logcases_adult)	Y ~ logcases_adult+logcases_5_11
Contra Costa	6-59m	Y ~ postvacc5+logcases_adult+logcases_5_11+logcases_less6m	Y ~ inschool+postvacc5+logcases_adult+logcases_5_11+logcases_less6m
Del Norte	6-59m	Y ~ offset(logcases_adult)	Y ~ postvacc5+logcases_adult*postvacc16+logcases_5_11+logcases_12_15
El Dorado	6-59m	Y ~ logcases_adult+logcases_5_11+logcases_12_15*postvacc16+logcases_less6m	Y ~ logcases_adult+logcases_12_15*postvacc12
Fresno	6-59m	Y ~ inschool+postvacc5+postvacc12+offset(logcases_5_11)	Y ~ sin_month + cos_month+postvacc5+logcases_adult+logcases_5_11+logcases_12_15*postvacc12+logcases_less6m
Glenn	6-59m	Y ~ inschool+postvacc16+offset(logcases_12_15)	Y ~ postvacc12+offset(logcases_adult)
Humboldt	6-59m	Y ~ sin_month + cos_month+inschool+logcases_adult+logcases_5_11+logcases_12_15	Y ~ inschool+logcases_adult*postvacc16+logcases_5_11*postvacc5
Imperial	6-59m	Y ~ postvacc12+logcases_adult+logcases_12_15	Y ~ inschool+postvacc5+postvacc12+postvacc16+logcases_adult+logcases_5_11+logcases_12_15
Inyo	6-59m	Y ~ logcases_adult+logcases_5_11+logcases_12_15	Y ~ inschool+logcases_adult*postvacc16+logcases_5_11*postvacc5+logcases_12_15*postvacc16+logcases_less6m
Kern	6-59m	Y ~ sin_month + cos_month+postvacc16+offset(logcases_5_11)	Y ~ sin_month + cos_month+logcases_12_15
Kings	6-59m	Y ~ inschool+offset(logcases_12_15)	Y ~ inschool+postvacc12+offset(logcases_12_15)
Lake	6-59m	Y ~ inschool+offset(logcases_5_11)	Y ~ postvacc5+logcases_12_15+logcases_less6m
Lassen	6-59m	Y ~ postvacc16+logcases_5_11	Y ~ sin_month + cos_month+logcases_5_11*postvacc5+logcases_less6m
Los Angeles	6-59m	Y ~ inschool+logcases_less6m	Y ~ postvacc5+logcases_adult+logcases_5_11+logcases_12_15+logcases_less6m
Madera	6-59m	Y ~ inschool+postvacc5+postvacc12+offset(logcases_12_15)	Y ~ inschool+postvacc5+offset(logcases_12_15)
Marin	6-59m	Y ~ logcases_adult+logcases_less6m	Y ~ logcases_5_11
Mariposa	6-59m	Y ~ offset(logcases_adult)	Y ~ postvacc5+offset(logcases_adult)
Mendocino	6-59m	Y ~ inschool+logcases_adult+logcases_12_15*postvacc12	Y ~ inschool+logcases_adult+logcases_12_15+logcases_less6m
Merced	6-59m	Y ~ inschool+postvacc16+offset(logcases_5_11)	Y ~ inschool+postvacc12+postvacc16+offset(logcases_adult)
Modoc	6-59m	Y ~ postvacc5+postvacc16+offset(logcases_12_15)	Y ~ postvacc5+logcases_5_11+logcases_12_15*postvacc16+logcases_less6m
Mono	6-59m	Y ~ offset(logcases_adult)	Y ~ inschool+postvacc5+postvacc16+logcases_adult+logcases_12_15

© 2024 Head JR et al. *JAMA Network Open.*

County	Age	Selected Model According to Mean Square Error	Selected Model According to Mean Absolute Error
Monterey	6-59m	Y ~ sin_month + cos_month+inschool+postvacc5+logcases_adult+logcases_12_15+logcases_less6m	Y ~ inschool+postvacc5+postvacc16+logcases_12_15+logcases_less6m
Napa	6-59m	Y ~ inschool+postvacc5+logcases_adult+logcases_5_11+logcases_12_15*postvacc12	Y ~ inschool+postvacc5+logcases_adult*postvacc16+logcases_5_11+logcases_12_15
Nevada	6-59m	Y ~ offset(logcases_5_11)	Y ~ offset(logcases_12_15)
Orange	6-59m	Y ~ logcases_adult+logcases_5_11+logcases_12_15	Y ~ postvacc16+offset(logcases_5_11)
Placer	6-59m	Y ~ logcases_adult+logcases_5_11+logcases_12_15*postvacc16	Y ~ postvacc16+logcases_adult+logcases_5_11+logcases_12_15*postvacc12
Plumas	6-59m	Y ~ postvacc5+offset(logcases_adult)	Y ~ offset(logcases_adult)
Riverside	6-59m	Y ~ inschool+postvacc16+logcases_5_11+logcases_less6m	Y ~ logcases_adult+logcases_5_11+logcases_less6m
Sacramento	6-59m	Y ~ postvacc12+logcases_adult+logcases_5_11+logcases_12_15*postvacc16	Y ~ logcases_adult+logcases_5_11*postvacc5+logcases_12_15*postvacc16
San Benito	6-59m	Y ~ inschool+offset(logcases_adult)	Y ~ postvacc16+logcases_adult+logcases_less6m
San Bernardino	6-59m	Y ~ offset(logcases_12_15)	Y ~ postvacc12+logcases_adult+logcases_5_11+logcases_less6m
San Diego	6-59m	Y ~ postvacc16+offset(logcases_5_11)	Y ~ inschool+postvacc5+postvacc12+offset(logcases_5_11)
San Francisco	6-59m	Y ~ logcases_adult+logcases_5_11+logcases_12_15	Y ~ logcases_adult+logcases_5_11+logcases_less6m
San Joaquin	6-59m	Y ~ postvacc5+postvacc16+logcases_adult+logcases_12_15	Y ~ postvacc5+postvacc16+logcases_adult+logcases_less6m
San Luis Obispo	6-59m	Y ~ logcases_adult*postvacc16+logcases_12_15*postvacc16	Y ~ postvacc5+logcases_adult+logcases_5_11+logcases_12_15*postvacc12
San Mateo	6-59m	Y ~ inschool+logcases_adult+logcases_5_11+logcases_12_15+logcases_less6m	Y ~ inschool+postvacc5+postvacc16+logcases_adult+logcases_5_11+logcases_less6m
Santa Barbara	6-59m	Y ~ postvacc12+postvacc16+logcases_12_15+logcases_less6m	Y ~ inschool+logcases_5_11+logcases_less6m
Santa Clara	6-59m	Y ~ postvacc16+logcases_5_11+logcases_less6m	Y ~ inschool+postvacc5+postvacc12+postvacc16+logcases_5_11+logcases_less6m
Santa Cruz	6-59m	Y ~ postvacc16+logcases_adult+logcases_5_11+logcases_12_15	Y ~ inschool+logcases_adult+logcases_5_11*postvacc5+logcases_12_15
Shasta	6-59m	Y ~ postvacc5+postvacc12+logcases_adult+logcases_5_11	Y ~ postvacc5+postvacc12+logcases_adult+logcases_5_11+logcases_12_15+logcases_less6m
Sierra	6-59m	Y ~ postvacc12+postvacc16+offset(logcases_adult)	Y ~ logcases_5_11+logcases_12_15
Siskiyou	6-59m	Y ~ inschool+postvacc5+offset(logcases_5_11)	Y ~ inschool+logcases_5_11*postvacc5
Solano	6-59m	Y ~ postvacc12+logcases_adult+logcases_12_15+logcases_less6m	Y ~ logcases_less6m
Sonoma	6-59m	Y ~ logcases_5_11+logcases_12_15+logcases_less6m	Y ~ inschool+postvacc5+offset(logcases_12_15)
Stanislaus	6-59m	Y ~ postvacc12+logcases_adult+logcases_12_15*postvacc16	Y ~ postvacc5+postvacc16+offset(logcases_adult)
Sutter	6-59m	Y ~ inschool+logcases_adult+logcases_5_11+logcases_less6m	Y ~ inschool+logcases_adult*postvacc16+logcases_12_15*postvacc16+logcases_less6m
Tehama	6-59m	Y ~ offset(logcases_5_11)	Y ~ postvacc12+logcases_adult*postvacc16+logcases_12_15*postvacc16
Trinity	6-59m	Y ~ inschool+postvacc5+offset(logcases_adult)	Y ~ logcases_5_11
Tulare	6-59m	Y ~ postvacc5+logcases_12_15	Y ~ postvacc5+logcases_adult*postvacc16+logcases_12_15*postvacc16+logcases_less6m
Tuolumne	6-59m	Y ~ offset(logcases_5_11)	Y ~ inschool+logcases_adult+logcases_5_11+logcases_12_15*postvacc16+logcases_less6m

© 2024 Head JR et al. *JAMA Network Open.*

County	Age	Selected Model According to Mean Square Error	Selected Model According to Mean Absolute Error
Ventura	6-59m	Y ~ offset(logcases_5_11)	Y ~ offset(logcases_12_15)
Yolo	6-59m	Y ~ sin_month + cos_month+inschool+logcases_adult+logcases_12_15+logcases_less6m	Y ~ sin_month + cos_month+postvacc16+logcases_adult+logcases_5_11
Yuba	6-59m	Y ~ sin_month + cos_month+offset(logcases_5_11)	Y ~ inschool+logcases_5_11*postvacc5+logcases_12_15+logcases_less6m
Alameda	5-11y	Y ~ postvacc12+logcases_6_59m+logcases_12_15+logcases_less6m	Y ~ inschool+postvacc16+logcases_12_15*postvacc12
Alpine	5-11y	Y ~ postvacc12+offset(logcases_adult)	Y ~ inschool+postvacc16+offset(logcases_less6m)
Amador	5-11y	Y ~ inschool+postvacc16+offset(logcases_adult)	Y ~ offset(logcases_12_15)
Butte	5-11y	Y ~ logcases_adult*postvacc16+logcases_6_59m+logcases_12_15	Y ~ logcases_12_15*postvacc12+logcases_less6m
Calaveras	5-11y	Y ~ logcases_12_15+logcases_less6m	Y ~ inschool+postvacc12+postvacc16+logcases_12_15
Colusa	5-11y	Y ~ inschool+postvacc12+logcases_adult+logcases_6_59m	Y ~ postvacc16+logcases_12_15+logcases_less6m
Contra Costa	5-11y	Y ~ inschool+postvacc12+logcases_6_59m	Y ~ sin_month + cos_month+inschool+postvacc12+offset(logcases_12_15)
Del Norte	5-11y	Y ~ inschool+logcases_adult+logcases_6_59m+logcases_12_15*postvacc12 + logcases_12_15*postvacc16+logcases_less6m	Y ~ inschool+postvacc12+postvacc16+logcases_6_59m+logcases_less6m
El Dorado	5-11y	Y ~ sin_month + cos_month+inschool+postvacc12+logcases_adult+logcases_6_59m	Y ~ sin_month + cos_month+inschool+postvacc12+offset(logcases_12_15)
Fresno	5-11y	Y ~ inschool+postvacc16+logcases_6_59m	Y ~ inschool+postvacc16+logcases_6_59m
Glenn	5-11y	Y ~ inschool+postvacc12+postvacc16+offset(logcases_adult)	Y ~ inschool+postvacc12+offset(logcases_adult)
Humboldt	5-11y	Y ~ inschool+logcases_6_59m+logcases_12_15*postvacc12	Y ~ inschool+logcases_6_59m+logcases_12_15*postvacc12
Imperial	5-11y	Y ~ sin_month + cos_month+inschool+postvacc12+offset(logcases_adult)	Y ~ postvacc12+logcases_6_59m+logcases_12_15
Inyo	5-11y	Y ~ inschool+postvacc16+logcases_adult	Y ~ inschool+postvacc16+logcases_adult+logcases_less6m
Kern	5-11y	Y ~ inschool+postvacc12+offset(logcases_12_15)	Y ~ sin_month + cos_month+inschool+logcases_adult+logcases_12_15+logcases_less6m
Kings	5-11y	Y ~ inschool+postvacc12+postvacc16+logcases_adult+logcases_12_15	Y ~ inschool+logcases_adult+logcases_6_59m+logcases_12_15*postvacc12+logcases_less6m
Lake	5-11y	Y ~ inschool+postvacc16+offset(logcases_adult)	Y ~ inschool+postvacc12+logcases_adult+logcases_12_15*postvacc16
Lassen	5-11y	Y ~ postvacc12+postvacc16+logcases_6_59m*inschool+logcases_12_15	Y ~ logcases_12_15
Los Angeles	5-11y	Y ~ inschool+logcases_adult*postvacc16+logcases_6_59m+logcases_less6m	Y ~ postvacc12+logcases_6_59m*inschool+logcases_12_15*postvacc16
Madera	5-11y	Y ~ inschool+postvacc16+offset(logcases_12_15)	Y ~ inschool+postvacc16+offset(logcases_12_15)
Marin	5-11y	Y ~ logcases_6_59m+logcases_12_15	Y ~ logcases_6_59m+logcases_12_15*postvacc16
Mariposa	5-11y	Y ~ logcases_adult+logcases_12_15	Y ~ logcases_adult*postvacc16+logcases_6_59m+logcases_12_15*postvacc16+logcases_less6m
Mendocino	5-11y	Y ~ inschool+postvacc12+offset(logcases_12_15)	Y ~ inschool+postvacc12+offset(logcases_12_15)
Merced	5-11y	Y ~ inschool+postvacc12+offset(logcases_adult)	Y ~ inschool+postvacc16+logcases_6_59m
Modoc	5-11y	Y ~ logcases_adult+logcases_6_59m	Y ~ inschool+postvacc12+logcases_adult+logcases_6_59m+logcases_less6m
Mono	5-11y	Y ~ offset(logcases_12_15)	Y ~ inschool+offset(logcases_12_15)

© 2024 Head JR et al. *JAMA Network Open.*

County	Age	Selected Model According to Mean Square Error	Selected Model According to Mean Absolute Error
Monterey	5-11y	Y ~ inschool+postvacc16+logcases_6_59m+logcases_12_15+logcases_less6m	Y ~ postvacc12+offset(logcases_12_15)
Napa	5-11y	Y ~ inschool+postvacc12+postvacc16+logcases_6_59m+logcases_12_15	Y ~ inschool+postvacc12+postvacc16+logcases_6_59m+logcases_12_15
Nevada	5-11y	Y ~ inschool+postvacc16+offset(logcases_6_59m)	Y ~ sin_month + cos_month+inschool+logcases_adult+logcases_12_15*postvacc16+logcases_less6m
Orange	5-11y	Y ~ inschool+postvacc12+postvacc16+logcases_6_59m+logcases_12_15	Y ~ inschool+postvacc12+postvacc16+logcases_adult+logcases_6_59m+logcases_12_15+logcases_less6m
Placer	5-11y	Y ~ inschool+logcases_adult+logcases_6_59m+logcases_12_15*postvacc12+logcases_less6m	Y ~ inschool+postvacc16+offset(logcases_12_15)
Plumas	5-11y	Y ~ offset(logcases_adult)	Y ~ logcases_6_59m*inschool+logcases_12_15*postvacc16+logcases_less6m
Riverside	5-11y	Y ~ logcases_adult*postvacc16+logcases_6_59m*inschool+logcases_12_15+logcases_less6m	Y ~ inschool+postvacc12+offset(logcases_12_15)
Sacramento	5-11y	Y ~ sin_month + cos_month+postvacc12+postvacc16+offset(logcases_12_15)	Y ~ sin_month + cos_month+inschool+postvacc12+postvacc16+offset(logcases_12_15)
San Benito	5-11y	Y ~ inschool+postvacc12+postvacc16+offset(logcases_adult)	Y ~ inschool+postvacc16+offset(logcases_adult)
San Bernardino	5-11y	Y ~ inschool+postvacc12+offset(logcases_12_15)	Y ~ inschool+postvacc12+offset(logcases_12_15)
San Diego	5-11y	Y ~ inschool+postvacc12+logcases_12_15	Y ~ inschool+postvacc12+postvacc16+logcases_6_59m+logcases_12_15+logcases_less6m
San Francisco	5-11y	Y ~ inschool+postvacc12+postvacc16+offset(logcases_6_59m)	Y ~ inschool+postvacc12+logcases_adult+logcases_6_59m+logcases_12_15+logcases_less6m
San Joaquin	5-11y	Y ~ inschool+postvacc12+logcases_6_59m+logcases_less6m	Y ~ inschool+postvacc16+offset(logcases_adult)
San Luis Obispo	5-11y	Y ~ sin_month + cos_month+inschool+postvacc12+postvacc16+offset(logcases_12_15)	Y ~ inschool+postvacc12+logcases_6_59m+logcases_12_15
San Mateo	5-11y	Y ~ postvacc12+logcases_adult+logcases_6_59m+logcases_12_15	Y ~ sin_month + cos_month+inschool+logcases_adult+logcases_6_59m+logcases_12_15+logcases_less6m
Santa Barbara	5-11y	Y ~ inschool+postvacc16+logcases_adult+logcases_12_15+logcases_less6m	Y ~ logcases_adult*postvacc16+logcases_6_59m+logcases_12_15*postvacc12+logcases_less6m
Santa Clara	5-11y	Y ~ inschool+postvacc12+postvacc16+logcases_6_59m+logcases_less6m	Y ~ sin_month + cos_month+inschool+postvacc12+offset(logcases_12_15)
Santa Cruz	5-11y	Y ~ postvacc16+offset(logcases_12_15)	Y ~ inschool+postvacc16+offset(logcases_12_15)
Shasta	5-11y	Y ~ sin_month + cos_month+inschool+postvacc12+postvacc16+logcases_adult+logcases_6_59m+logcases_12_15+logcases_less6m	Y ~ logcases_6_59m+logcases_less6m

© 2024 Head JR et al. *JAMA Network Open.*

County	Age	Selected Model According to Mean Square Error	Selected Model According to Mean Absolute Error
Sierra	5-11y	Y ~ postvacc12+offset(logcases_adult)	Y ~ logcases_adult+postvacc16+logcases_12_15+postvacc12+logcases_adult:postvacc16+logcases_12_15:postvacc12+postvacc16:logcases_12_15
Siskiyou	5-11y	Y ~ logcases_6_59m+logcases_12_15+logcases_less6m	Y ~ logcases_6_59m+logcases_12_15+logcases_less6m
Solano	5-11y	Y ~ postvacc12+logcases_6_59m*inschool+logcases_12_15	Y ~ inschool+postvacc12+postvacc16+logcases_adult+logcases_12_15
Sonoma	5-11y	Y ~ inschool+postvacc12+offset(logcases_12_15)	Y ~ inschool+postvacc12+postvacc16+offset(logcases_12_15)
Stanislaus	5-11y	Y ~ sin_month + cos_month+inschool+postvacc12+offset(logcases_12_15)	Y ~ logcases_6_59m+logcases_12_15*postvacc12
Sutter	5-11y	Y ~ inschool+postvacc16+logcases_6_59m+logcases_12_15+logcases_less6m	Y ~ inschool+postvacc16+offset(logcases_12_15)
Tehama	5-11y	Y ~ postvacc12+logcases_6_59m	Y ~ postvacc12+logcases_6_59m*inschool
Trinity	5-11y	Y ~ offset(logcases_adult)	Y ~ logcases_6_59m
Tulare	5-11y	Y ~ inschool+offset(logcases_12_15)	Y ~ inschool+offset(logcases_12_15)
Tuolumne	5-11y	Y ~ offset(logcases_12_15)	Y ~ sin_month + cos_month+inschool+postvacc16+logcases_12_15+logcases_less6m
Ventura	5-11y	Y ~ inschool+postvacc12+offset(logcases_12_15)	Y ~ inschool+offset(logcases_12_15)
Yolo	5-11y	Y ~ postvacc16+offset(logcases_12_15)	Y ~ postvacc16+logcases_12_15
Yuba	5-11y	Y ~ inschool+postvacc16+logcases_6_59m+logcases_12_15	Y ~ inschool+logcases_6_59m+logcases_12_15+logcases_less6m
Alameda	12-15y	Y ~ logcases_adult+logcases_5_11	Y ~ logcases_6_59m*inschool+logcases_5_11
Alpine	12-15y	Y ~ offset(logcases_adult)	Y ~ inschool+postvaccadult+offset(logcases_5_11)
Amador	12-15y	Y ~ logcases_5_11+logcases_less6m	Y ~ logcases_6_59m+logcases_5_11
Butte	12-15y	Y ~ inschool+offset(logcases_5_11)	Y ~ logcases_5_11+logcases_less6m
Calaveras	12-15y	Y ~ inschool+logcases_adult	Y ~ inschool+logcases_5_11
Colusa	12-15y	Y ~ logcases_adult+logcases_5_11*inschool	Y ~ logcases_adult+logcases_5_11
Contra Costa	12-15y	Y ~ inschool+offset(logcases_5_11)	Y ~ inschool+offset(logcases_5_11)
Del Norte	12-15y	Y ~ inschool+offset(logcases_adult)	Y ~ logcases_5_11*inschool+logcases_less6m
El Dorado	12-15y	Y ~ logcases_adult+logcases_5_11*inschool	Y ~ logcases_6_59m+logcases_5_11+logcases_less6m
Fresno	12-15y	Y ~ logcases_6_59m+logcases_5_11	Y ~ inschool+logcases_6_59m+logcases_5_11+logcases_less6m
Glenn	12-15y	Y ~ inschool+offset(logcases_adult)	Y ~ inschool+offset(logcases_adult)
Humboldt	12-15y	Y ~ inschool+offset(logcases_5_11)	Y ~ logcases_adult+logcases_5_11*inschool+logcases_less6m
Imperial	12-15y	Y ~ logcases_6_59m+logcases_5_11*inschool+logcases_less6m	Y ~ logcases_adult+logcases_less6m
Inyo	12-15y	Y ~ offset(logcases_adult)	Y ~ logcases_6_59m+logcases_5_11
Kern	12-15y	Y ~ inschool+logcases_5_11	Y ~ postvaccadult+offset(logcases_5_11)
Kings	12-15y	Y ~ offset(logcases_5_11)	Y ~ logcases_adult+logcases_6_59m*inschool+logcases_5_11*inschool
Lake	12-15y	Y ~ postvaccadult+offset(logcases_adult)	Y ~ logcases_6_59m+logcases_5_11+logcases_less6m
Lassen	12-15y	Y ~ logcases_5_11	Y ~ offset(logcases_5_11)
Los Angeles	12-15y	Y ~ postvaccadult+offset(logcases_5_11)	Y ~ offset(logcases_5_11)
Madera	12-15y	Y ~ logcases_6_59m+logcases_5_11*inschool	Y ~ inschool+logcases_adult+logcases_6_59m+logcases_5_11+logcases_less6m
Marin	12-15y	Y ~ postvaccadult+offset(logcases_6_59m)	Y ~ logcases_6_59m*inschool+logcases_5_11*inschool
Mariposa	12-15y	Y ~ inschool+offset(logcases_5_11)	Y ~ logcases_6_59m*inschool+logcases_5_11
Mendocino	12-15y	Y ~ offset(logcases_5_11)	Y ~ logcases_6_59m+logcases_5_11
Merced	12-15y	Y ~ logcases_6_59m*inschool+logcases_5_11*inschool	Y ~ logcases_5_11
Modoc	12-15y	Y ~ inschool+logcases_adult	Y ~ logcases_adult+logcases_6_59m+logcases_less6m
Mono	12-15y	Y ~ logcases_5_11+logcases_less6m	Y ~ logcases_5_11*inschool+logcases_less6m

© 2024 Head JR et al. *JAMA Network Open.*

County	Age	Selected Model According to Mean Square Error	Selected Model According to Mean Absolute Error
Monterey	12-15y	Y ~ offset(logcases_5_11)	Y ~ postvaccadult+offset(logcases_5_11)
Napa	12-15y	Y ~ logcases_adult+logcases_6_59m*inschool+logcases_5_11*inschool	Y ~ inschool+logcases_adult+logcases_6_59m+logcases_5_11
Nevada	12-15y	Y ~ inschool+offset(logcases_5_11)	Y ~ inschool+offset(logcases_5_11)
Orange	12-15y	Y ~ postvaccadult+offset(logcases_5_11)	Y ~ offset(logcases_5_11)
Placer	12-15y	Y ~ inschool+offset(logcases_5_11)	Y ~ inschool+logcases_adult+logcases_5_11
Plumas	12-15y	Y ~ postvaccadult+offset(logcases_adult)	Y ~ logcases_6_59m+logcases_less6m
Riverside	12-15y	Y ~ logcases_adult+logcases_5_11	Y ~ inschool+logcases_5_11
Sacramento	12-15y	Y ~ inschool+logcases_5_11+logcases_less6m	Y ~ inschool+logcases_5_11+logcases_less6m
San Benito	12-15y	Y ~ postvaccadult+offset(logcases_adult)	Y ~ postvaccadult+offset(logcases_adult)
San Bernardino	12-15y	Y ~ logcases_5_11+logcases_less6m	Y ~ logcases_5_11+logcases_less6m
San Diego	12-15y	Y ~ inschool+offset(logcases_5_11)	Y ~ inschool+offset(logcases_5_11)
San Francisco	12-15y	Y ~ inschool+postvaccadult+offset(logcases_6_59m)	Y ~ inschool+logcases_5_11
San Joaquin	12-15y	Y ~ offset(logcases_5_11)	Y ~ inschool+postvaccadult+offset(logcases_adult)
San Luis Obispo	12-15y	Y ~ logcases_5_11+logcases_less6m	Y ~ logcases_adult+logcases_6_59m*inschool+logcases_5_11*inschool+logcases_less6m
San Mateo	12-15y	Y ~ logcases_6_59m*inschool	Y ~ inschool+postvaccadult+offset(logcases_5_11)
Santa Barbara	12-15y	Y ~ logcases_adult+logcases_6_59m+logcases_5_11	Y ~ logcases_5_11
Santa Clara	12-15y	Y ~ inschool+postvaccadult+offset(logcases_5_11)	Y ~ logcases_6_59m*inschool+logcases_5_11*inschool
Santa Cruz	12-15y	Y ~ logcases_adult+logcases_5_11*inschool	Y ~ inschool+logcases_adult+logcases_5_11
Shasta	12-15y	Y ~ logcases_adult+logcases_5_11*inschool	Y ~ logcases_adult+logcases_5_11*inschool+logcases_less6m
Sierra	12-15y	Y ~ offset(logcases_adult)	Y ~ inschool+logcases_5_11
Siskiyou	12-15y	Y ~ inschool+postvaccadult+offset(logcases_adult)	Y ~ inschool+postvaccadult+offset(logcases_adult)
Solano	12-15y	Y ~ postvaccadult+offset(logcases_5_11)	Y ~ postvaccadult+offset(logcases_5_11)
Sonoma	12-15y	Y ~ postvaccadult+offset(logcases_5_11)	Y ~ inschool+logcases_adult+logcases_5_11+logcases_less6m
Stanislaus	12-15y	Y ~ postvaccadult+offset(logcases_5_11)	Y ~ logcases_adult+logcases_5_11*inschool+logcases_less6m
Sutter	12-15y	Y ~ logcases_adult+logcases_less6m	Y ~ inschool+logcases_adult+logcases_5_11+logcases_less6m
Tehama	12-15y	Y ~ inschool	Y ~ inschool+offset(logcases_5_11)
Trinity	12-15y	Y ~ offset(logcases_adult)	Y ~ logcases_adult+logcases_6_59m*inschool
Tulare	12-15y	Y ~ inschool+postvaccadult+offset(logcases_5_11)	Y ~ inschool+logcases_adult+logcases_5_11
Tuolumne	12-15y	Y ~ logcases_5_11*inschool	Y ~ inschool+logcases_6_59m+logcases_5_11
Ventura	12-15y	Y ~ inschool+postvaccadult+offset(logcases_5_11)	Y ~ inschool+offset(logcases_5_11)
Yolo	12-15y	Y ~ postvaccadult+offset(logcases_5_11)	Y ~ inschool+postvaccadult+offset(logcases_5_11)
Yuba	12-15y	Y ~ logcases_adult+logcases_6_59m*inschool	Y ~ logcases_adult+logcases_6_59m*inschool

© 2024 Head JR et al. *JAMA Network Open.*

eTable 3. Best predictive models selected for each region-age group combination using hospitalization as outcome. Description for variable names are given in eTable 1.

Region	Age	Selected Model According to Mean Square Error	Selected Model According to Mean Absolute Error
Bay Area	6-59m	Y ~ inschool+postvacc5+loglag2cases_12_15+loglag2cases_5_11+loglag2cases_less6m+loghosp_12_15*postvacc16+loghosp_5_11+loghosp_less6m	Y ~ inschool+postvacc12+postvacc16+loglag2cases_12_15+loglag2cases_5_11*postvacc5+loglag2cases_less6m+loghosp_12_15
Central	6-59m	Y ~ sin_month + cos_month+inschool+postvacc12+postvacc16+loghosp_12_15+loghosp_less6m	Y ~ postvacc5+loglag2cases_less6m+loghosp_5_11+loghosp_less6m
Northern	6-59m	Y ~ loglag2cases_less6m+loghosp_12_15	Y ~ postvacc5+loghosp_12_15*postvacc12+loghosp_less6m
Southeast	6-59m	Y ~ postvacc16+loghosp_5_11+loghosp_less6m	Y ~ postvacc16+loghosp_12_15+loghosp_5_11+loghosp_less6m
Southwest	6-59m	Y ~ postvacc5+postvacc12+postvacc16+loghosp_12_15+loghosp_less6m	Y ~ inschool+postvacc5+postvacc12+loghosp_12_15+loghosp_less6m
Bay Area	5-11y	Y ~ inschool+postvacc12+loglag2cases_12_15*postvacc16+loglag2cases_6_59m	Y ~ inschool+loglag2cases_adult+loglag2cases_12_15*postvacc12+loglag2cases_6_59m
Central	5-11y	Y ~ loghosp_less6m	Y ~ loghosp_6_59m+loghosp_less6m
Northern	5-11y	Y ~ postvacc12+postvacc16+loglag2cases_adult+loghosp_12_15+loghosp_6_59m+loghosp_less6m	Y ~ loglag2cases_12_15*postvacc12+loghosp_12_15*postvacc16+loghosp_6_59m+loghosp_less6m
Southeast	5-11y	Y ~ postvacc12+postvacc16+loglag2cases_adult+loghosp_12_15+loghosp_6_59m	Y ~ inschool+postvacc12+loghosp_12_15+loghosp_6_59m
Southwest	5-11y	Y ~ loghosp_12_15+loghosp_6_59m	Y ~ postvacc12+loglag2cases_adult*postvacc16+loglag2cases_12_15*postvacc16+loglag2cases_less6m+loghosp_12_15+loghosp_6_59m+loghosp_less6m
Bay Area	12-15y	Y ~ inschool+loglag2cases_6_59m	Y ~ inschool+postvacc16+loglag2cases_5_11+loghosp_6_59m
Central	12-15y	Y ~ inschool+postvacc16+loghosp_5_11+loghosp_6_59m	Y ~ inschool+loghosp_5_11
Northern	12-15y	Y ~ loglag2cases_less6m+loghosp_5_11	Y ~ inschool+postvacc16+loglag2cases_5_11+loglag2cases_less6m+loghosp_5_11
Southeast	12-15y	Y ~ postvacc16+loglag2cases_5_11+loghosp_5_11+loghosp_less6m	Y ~ inschool+loghosp_5_11+loghosp_6_59m+loghosp_less6m
Southwest	12-15y	Y ~ loglag2cases_adult+loglag2cases_6_59m+loglag2cases_less6m+loghosp_less6m	Y ~ loglag2cases_adult+loghosp_5_11

© 2024 Head JR et al. *JAMA Network Open.*

eTable 4. Statewide estimates of averted COVID-19 cases due to vaccination among children aged 6 months to 15 years of age using mean absolute error as the loss function. PI = prediction interval

**Age group (vaccine eligibility date)**	**Post vaccine period (length, days)**	**Children vaccinated No. (%)**	**Observed cases**	**Expected cases** No. (95% PI)	**Averted cases** No. (95% PI)	**Percent averted (%)** No. (95% PI)
12-15 years (May 10, 2021)	June 10, 2021 – Oct 29, 2021 (144 d)	1,712,868 (53.5%)	248,296	403,009 (394,008 – 421,011)	**154,713** (145,712 – 172,715)	**38.3** (36.2, 42.9)
5-11 years (Oct 29, 2021)	Nov 19, 2021 – June 17, 2022 (199 d)	1,219,432 (34.8%)	739,830	911,378 (886,692 – 937,506)	**171,548** (146,862 – 937,506)	**18.8** (16.1 – 21.7)
6-59 months (June 17, 2022)	July 17, 2022 – Feb 27, 2023 (226 d)	177,087 (7.9%)	67,287	52,518 (50,975 – 53,865)	**-14,768** (-16,312 – -13,422)	**-28.1** (-31.1 – -25.6)

© 2024 Head JR et al. *JAMA Network Open.*

eTable 5. Statewide estimates of averted COVID-19 hospitalizations due to vaccination among children aged 6 months to 15 years of age using mean absolute error as the loss function. PI = prediction interval

**Age group (vaccine eligibility date)**	**Post vaccine period (length, days)**	**Children vaccinated No. (%)**	**Observed hospitalizations**	**Expected hospitalizations** No. (95% PI)	**Averted hospitalizations** No. (95% PI)	**Percent averted (%)** No. (95% PI)
12-15 years (May 10, 2021)	June 10, 2021 – Oct 29, 2021 (144 d)	1,712,868 (53.5%)	688	771 (633 – 963)	**83** (-55 – 275)	**10.8** (-7.1 – 35.7)
5-11 years (Oct 29, 2021)	Nov 19, 2021 – June 17, 2022 (199 d)	1,219,432 (34.8%)	729	724 (588 – 931)	**-5** (-141 – 202)	**-0.7** (-19.5 – 27.9)
6-59 months (June 17, 2022)	July 17, 2022 – Feb 27, 2023 (226 d)	177,087 (7.9%)	520	687 (558 – 868)	**166** (39 – 348)	**24.2** (5.6 – 50.7)

© 2024 Head JR et al. *JAMA Network Open.*

eTable 6. Estimated number of COVID-19 cases averted by county and age group during the post-vaccination period. Numbers in parenthesis indicate 95% prediction intervals (PI). Estimates using two different loss functions are shown per age group: mean square error (MSE) and mean absolute error (MAE).

County	**Children 6-59 months** Cases averted (95% Prediction Interval)		**Children 5-11 years** Cases averted (95% Prediction Interval)		**Children 12-15 years** Cases averted (95% Prediction Interval)	
	**MSE**	**MAE**	**MSE**	**MAE**	**MSE**	**MAE**
Alameda	-460 (-707, -217)	-1851 (-2021, -1682)	750 (-2327, 4145)	13690 (10891, 16773)	2024 (1156, 2986)	1430 (-282, 3311)
Alpine	0 (0, 0)	0 (0, 0)	-1 (-2, 4)	-1 (-2, 2)	-1 (-4, 3)	-1 (-4, 17760448)
Amador	-8 (-17, 2)	-10 (-21, 4)	54 (-44, 143)	-414 (-467, -358)	490 (125, 1123)	883 (298, 1985)
Butte	-295 (-350, -234)	-297 (-352, -236)	428 (192, 686)	1127 (795, 1499)	688 (534, 865)	1400 (1008, 1949)
Calaveras	-7 (-15, 5)	1 (-12, 15)	-11 (-76, 56)	28 (-36, 93)	-389 (-439, -325)	467 (62, 1219)
Colusa	-11 (-24, 2)	-26 (-35, -16)	175 (89, 262)	228 (124, 344)	-69 (-106, -21)	-59 (-95, -11)
Contra Costa	53 (-101, 202)	46 (-97, 199)	37484 (33416, 41934)	5634 (2334, 9411)	22312 (21227, 23436)	22312 (21227, 23436)
Del Norte	-13 (-27, -1)	-24 (-38, -9)	197 (51, 406)	249 (75, 514)	164 (88, 248)	1216 (438, 3334)
El Dorado	-5 (-36, 38)	0 (-30, 37)	-161 (-482, 197)	-52 (-401, 337)	276 (-54, 759)	2168 (1818, 2589)
Fresno	-590 (-670, -515)	-133 (-297, 47)	5976 (5270, 6769)	5976 (5270, 6769)	6034 (4910, 7159)	6058 (4943, 7148)
Glenn	-55 (-73, -36)	-61 (-79, -43)	26 (-33, 80)	26 (-33, 80)	-69 (-106, -26)	-69 (-106, -26)
Humboldt	-17 (-75, 55)	50 (-25, 140)	-701 (-863, -540)	-701 (-863, -540)	331 (126, 537)	3507 (1469, 6754)
Imperial	-59 (-118, 12)	-77 (-140, -1)	791 (5, 1576)	7005 (5791, 8348)	806 (102, 1917)	-1275 (-1329, -1222)
Inyo	-14 (-22, -5)	-4 (-18, 12)	154 (85, 229)	125 (40, 230)	-62 (-83, -36)	423 (212, 743)
Kern	-782 (-860, -715)	-658 (-746, -580)	-331 (-690, 42)	-2418 (-3193, -1729)	2915 (2397, 3417)	93 (-357, 497)
Kings	-45 (-71, -10)	-42 (-70, -10)	416 (116, 710)	313 (-117, 736)	-271 (-395, -142)	-1093 (-1311, -849)
Lake	-28 (-46, -9)	-45 (-63, -25)	-328 (-411, -236)	-178 (-433, 226)	-14 (-93, 94)	-30 (-108, 79)
Lassen	-1 (-10, 9)	13 (-4, 37)	240 (36, 845)	105 (12, 249)	152 (-39, 510)	-146 (-169, -120)
Los Angeles	14567 (13874, 15295)	-1497 (-2100, -808)	69007 (45275, 98793)	76025 (56661, 97774)	7042 (6126, 7979)	11145 (10590, 11711)
Madera	-112 (-146, -75)	-114 (-145, -74)	-654 (-876, -419)	-654 (-876, -419)	266 (-440, 1275)	1042 (238, 2084)
Marin	646 (572, 726)	-3 (-48, 46)	5935 (4758, 7461)	-1123 (-1912, 5)	1892 (1571, 2231)	586 (454, 739)
Mariposa	12 (2, 23)	14 (2, 26)	92 (18, 167)	96 (21, 200)	-138 (-175, -94)	1040 (273, 2821)
Mendocino	-2 (-30, 27)	-8 (-33, 18)	770 (602, 925)	770 (602, 925)	974 (792, 1171)	2542 (1554, 3988)
Merced	-317 (-347, -286)	69 (17, 130)	5276 (4424, 6176)	1414 (921, 2042)	1859 (-199, 4826)	9508 (6956, 12643)
Modoc	12 (2, 24)	4 (-4, 14)	-8 (-19, 4)	1 (-16, 21)	0 (-15, 18)	-15 (-25, -2)
Mono	1 (-9, 10)	27 (8, 48)	-64 (-81, -47)	-57 (-77, -39)	-61 (-150, 95)	-114 (-219, 92)
Monterey	-595 (-765, -396)	-871 (-1005, -717)	4153 (2121, 6207)	5244 (4296, 6297)	3447 (3049, 3964)	2922 (2198, 3634)

© 2024 Head JR et al. *JAMA Network Open.*

County	Children 6-59 months<br>Cases averted (95% Prediction Interval)		Children 5-11 years<br>Cases averted (95% Prediction Interval)		Children 12-15 years<br>Cases averted (95% Prediction Interval)	
	**MSE**	**MAE**	**MSE**	**MAE**	**MSE**	**MAE**
Napa	-22 (-52, 17)	-22 (-53, 13)	3758 (2744, 5027)	3758 (2744, 5027)	-249 (-352, -114)	71 (-77, 285)
Nevada	43 (21, 64)	46 (25, 70)	125 (-11, 276)	-799 (-996, -576)	2 (-100, 106)	2 (-100, 106)
Orange	-1590 (-1741, -1446)	-2380 (-2513, -2243)	6055 (4674, 7621)	7627 (5764, 9812)	9855 (8896, 11044)	9768 (9108, 10534)
Placer	-117 (-163, -63)	-116 (-162, -61)	1245 (704, 1868)	180 (-18, 389)	1563 (1384, 1771)	1113 (796, 1503)
Plumas	15 (3, 26)	13 (3, 24)	-123 (-166, -75)	-83 (-154, -5)	-73 (-156, 28)	-261 (-291, -227)
Riverside	-1309 (-1470, -1136)	-1071 (-1253, -904)	6739 (4018, 9901)	-111 (-901, 590)	3466 (2861, 4112)	4799 (4449, 5177)
Sacramento	-650 (-773, -526)	-713 (-837, -592)	-4385 (-5904, -2687)	-6646 (-8122, -4988)	29381 (25455, 33637)	29381 (25455, 33637)
San Benito	-22 (-48, 1)	27 (-5, 57)	412 (260, 573)	441 (286, 588)	81 (27, 145)	81 (27, 145)
San Bernardino	-2335 (-2433, -2237)	-754 (-952, -550)	983 (217, 1610)	983 (217, 1610)	6108 (5365, 7004)	6108 (5365, 7004)
San Diego	-3009 (-3120, -2899)	-2924 (-3039, -2807)	7429 (5487, 9523)	3314 (60, 6714)	3907 (3628, 4202)	3907 (3628, 4202)
San Francisco	-342 (-419, -259)	-154 (-264, -47)	6496 (6019, 6969)	8199 (6944, 9887)	1807 (1438, 2174)	2425 (2096, 2785)
San Joaquin	-249 (-362, -138)	539 (418, 665)	8968 (7363, 10964)	7492 (6635, 8502)	5040 (4597, 5574)	797 (361, 1260)
San Luis Obispo	-1 (-48, 57)	-10 (-52, 43)	-646 (-1149, -68)	1600 (1023, 2231)	1820 (1356, 2340)	3000 (1493, 5380)
San Mateo	-366 (-464, -269)	-362 (-466, -261)	9295 (7100, 11455)	4510 (1797, 7770)	2304 (1674, 3073)	10010 (8736, 11374)
Santa Barbara	103 (7, 195)	102 (29, 182)	-470 (-1270, 489)	3037 (706, 6167)	4979 (3688, 6507)	3732 (2921, 4579)
Santa Clara	-994 (-1263, -692)	-1056 (-1321, -723)	38215 (31818, 45201)	13779 (9375, 18650)	21070 (18772, 23607)	3982 (7, 9973)
Santa Cruz	15 (-37, 78)	-49 (-96, 13)	1013 (632, 1353)	1084 (705, 1442)	-232 (-495, 88)	160 (-115, 455)
Shasta	-109 (-147, -71)	-135 (-169, -91)	-45 (-281, 225)	-194 (-315, -59)	-485 (-833, -38)	-230 (-698, 426)
Sierra	5 (1, 11)	2 (0, 5)	-1 (-10, 10)	-4 (-11, 7)	-12 (-16, -5)	-13 (-16, 62)
Siskiyou	14 (-1, 28)	18 (-2, 39)	-9 (-101, 100)	-9 (-101, 100)	-84 (-183, 41)	-84 (-183, 41)
Solano	34 (-64, 130)	621 (533, 708)	7418 (6150, 9096)	3335 (2661, 4118)	2704 (2393, 3038)	2704 (2393, 3038)
Sonoma	-141 (-205, -71)	-352 (-385, -316)	6694 (6234, 7242)	6686 (6165, 7189)	1792 (1537, 2057)	1458 (1147, 1827)
Stanislaus	-325 (-416, -242)	-64 (-156, 43)	-4061 (-4841, -3238)	-673 (-1205, -43)	3098 (2579, 3608)	2029 (-36, 4614)
Sutter	-37 (-65, -6)	-28 (-62, 11)	1742 (1006, 2514)	-271 (-450, -116)	-1877 (-1924, -1821)	-1735 (-1936, -1404)
Tehama	-118 (-135, -101)	-64 (-96, -28)	145 (13, 295)	182 (38, 355)	-412 (-553, -248)	2619 (2150, 3118)
Trinity	0 (-12, 11)	1 (-9, 12)	74 (43, 106)	8 (-14, 29)	37 (6, 71)	268 (94, 673)
Tulare	-129 (-218, -42)	115 (-3, 229)	1245 (890, 1575)	1245 (890, 1575)	-1289 (-1660, -931)	339 (-100, 777)
Tuolumne	-22 (-35, -9)	-3 (-23, 22)	-396 (-454, -341)	-306 (-467, -114)	-778 (-818, -699)	567 (194, 1081)
Ventura	-505 (-555, -461)	-520 (-566, -473)	810 (421, 1198)	-1460 (-1796, -1118)	2126 (1753, 2578)	1939 (1668, 2201)
Yolo	30 (-10, 74)	19 (-25, 65)	1656 (1436, 1851)	1929 (1677, 2208)	440 (290, 586)	316 (158, 490)
Yuba	NA	NA	92 (-156, 375)	262 (-125, 729)	-477 (-517, -431)	-477 (-517, -431)

© 2024 Head JR et al. *JAMA Network Open.*

eTable 7. Estimated number of COVID-19 hospitalizations averted by region and age group during the post-vaccination period. Numbers in parenthesis indicate 95% prediction intervals. Estimates using two different loss functions are shown per age group: mean square error (MSE) and mean absolute error (MAE). Counties included in each of the five regions are shown in eFigure 1.

**Region**	**Children 6-59 months** Cases averted (95% Prediction Interval)		**Children 5-11 years** Cases averted (95% Prediction Interval)		**Children 12-15 years** Cases averted (95% Prediction Interval)	
	**MSE**	**MAE**	**MSE**	**MSE**	**MAE**	**MSE**
Bay Area	59 (16, 123)	85 (29, 191)	-14 (-48, 31)	-4 (-45, 54)	85 (49, 130)	125 (44, 233)
Central	42 (10, 98)	40 (4, 91)	13 (-27, 68)	63 (0, 162)	-19 (-70, 72)	-26 (-65, 16)
Northern	33 (13, 58)	23 (4, 46)	8 (-15, 43)	-2 (-20, 25)	-6 (-29, 34)	25 (-24, 154)
Southeast	5 (-22, 36)	-3 (-27, 27)	71 (-15, 179)	-40 (-100, 34)	-13 (-87, 76)	-42 (-87, 1)
Southwest	29 (-38, 104)	23 (-45, 105)	-32 (-90, 38)	-22 (-95, 115)	13 (-28, 64)	1 (-32, 39)

© 2024 Head JR et al. *JAMA Network Open.*

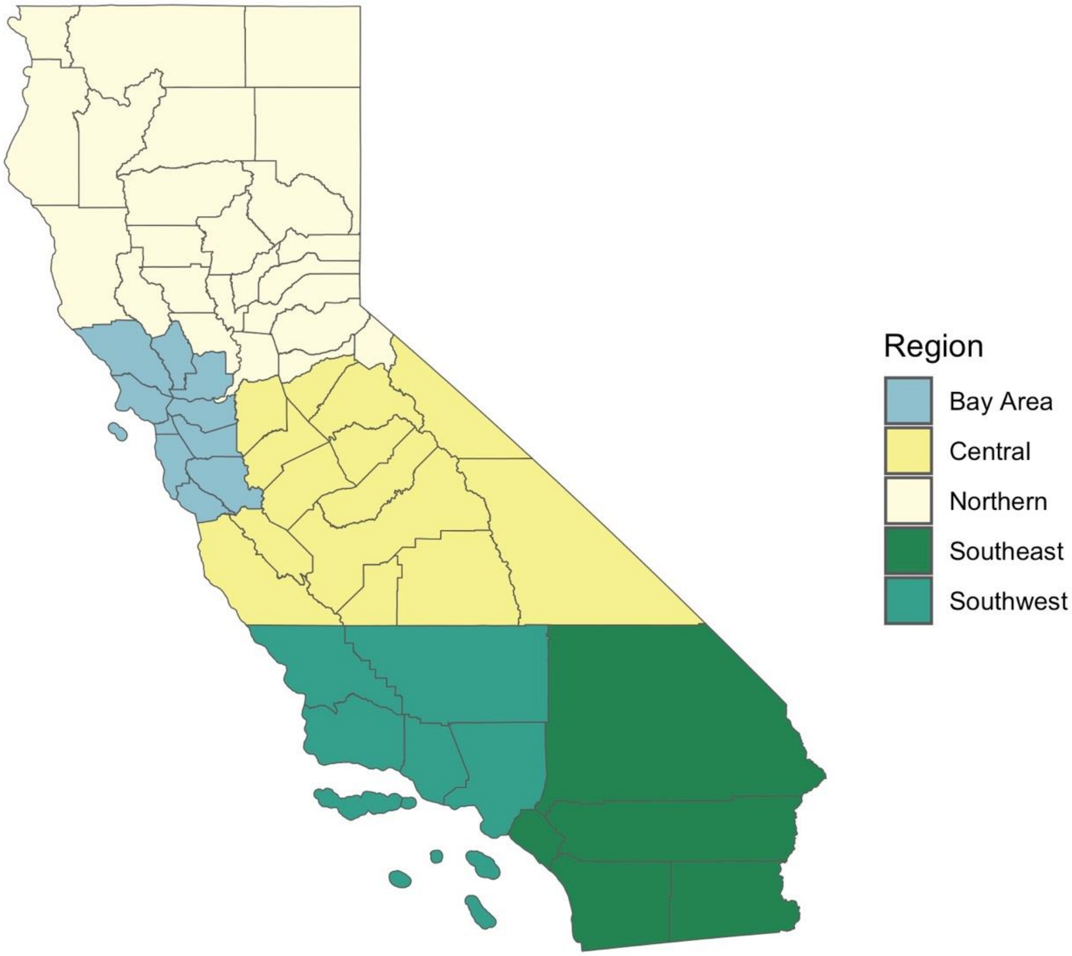

eFigure 1. Map of the five regions used for estimating the effect of pediatric SARS-CoV-2 vaccines on childhood hospitalizations for COVID-19. Regions are designated by the California Department of Public Health.

© 2024 Head JR et al. *JAMA Network Open.*

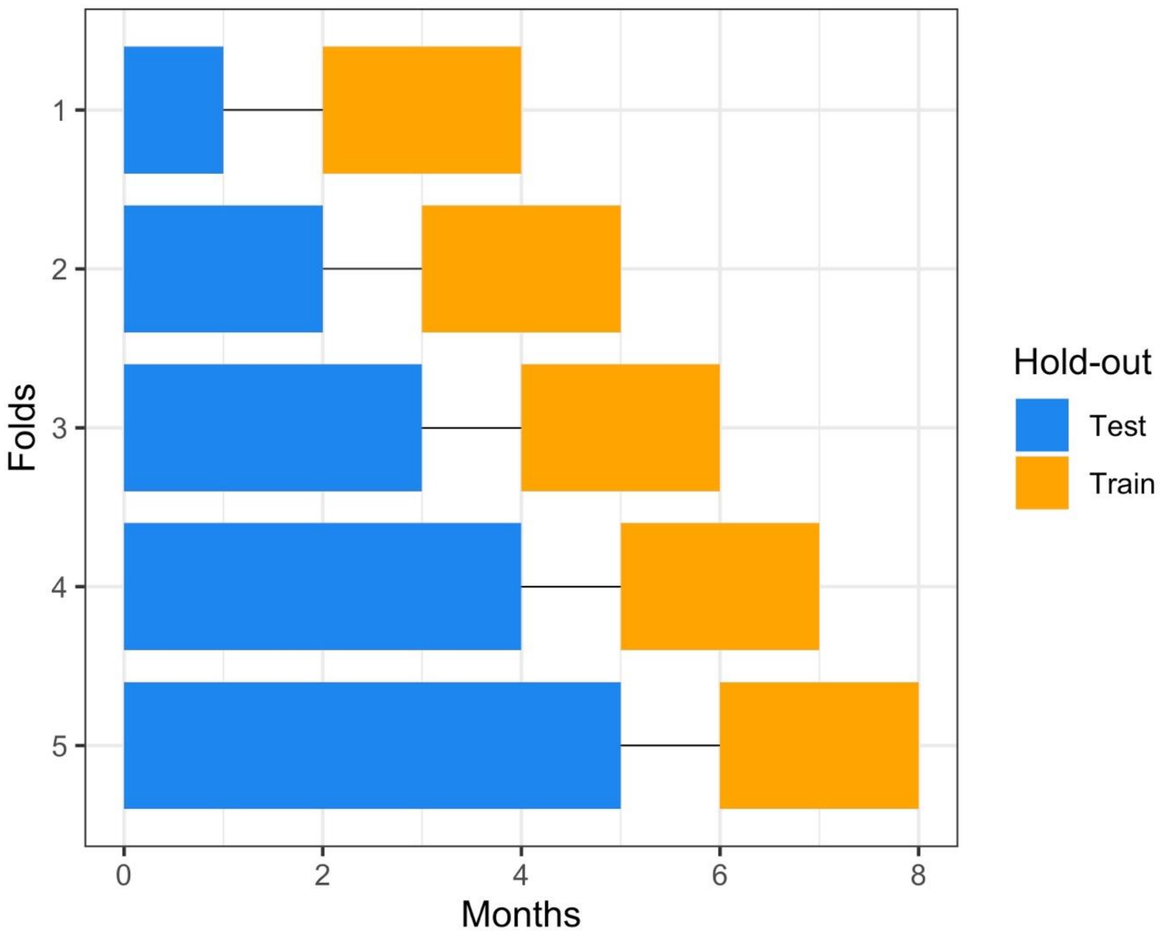

eFigure 2. Schematic of the time series with gap cross-validation algorithm used to select the best predictive algorithm during the pre-period.

© 2024 Head JR et al. *JAMA Network Open.*

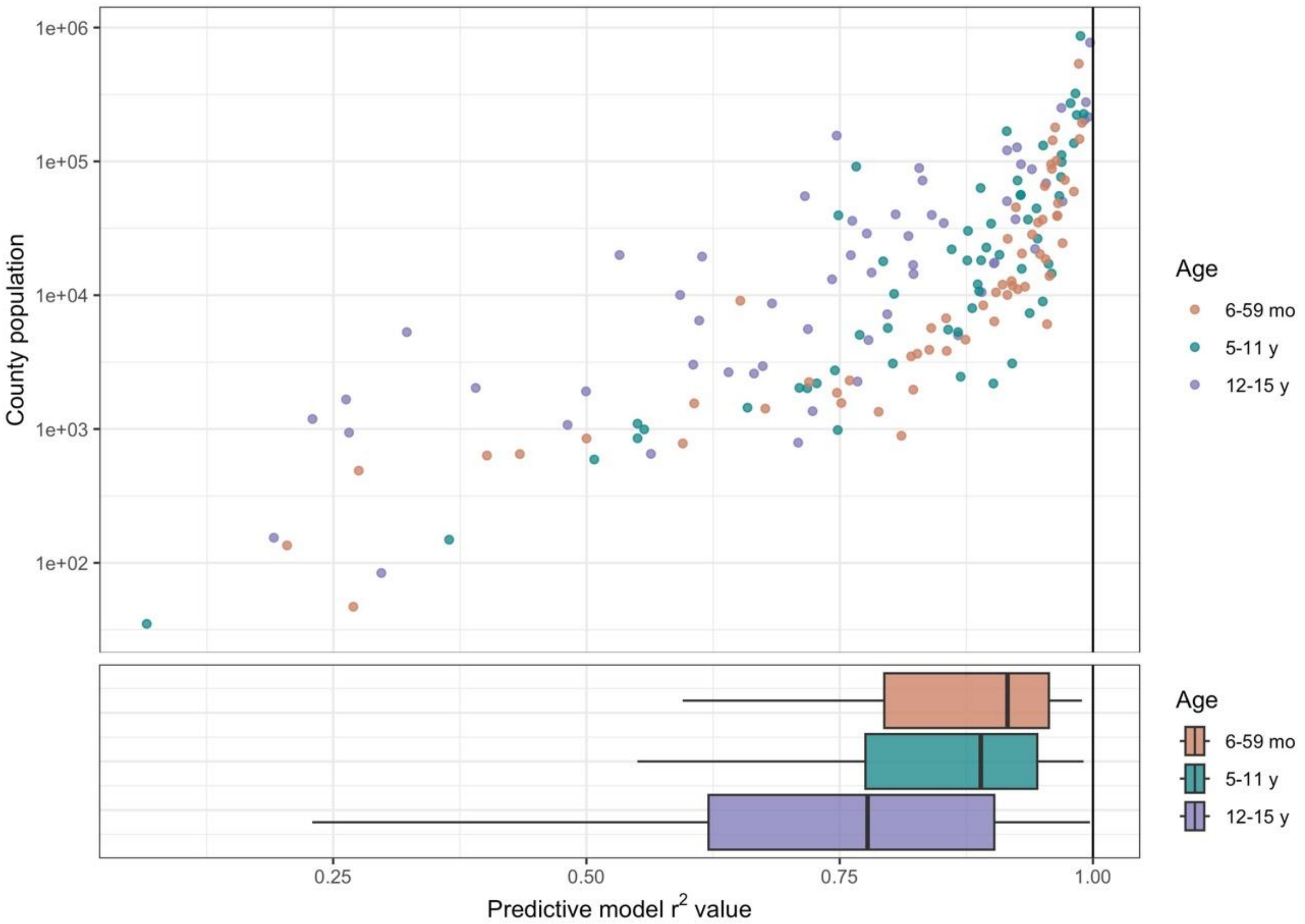

eFigure 3. Model performance of selected generalized linear model predicting cases during the pre-vaccine period for each county-age group combination. Top panel: R^2 values of the predictive models fit to pre-vaccine case data for each age group/county combination, plotted against the county population for the age group. Bottom panel: boxplot of the R^2 values from California's 58 counties. The black vertical line within the colored box is the median, the colored box spans the interquartile range, and horizontal lines denote the 95% CI.

© 2024 Head JR et al. *JAMA Network Open.*

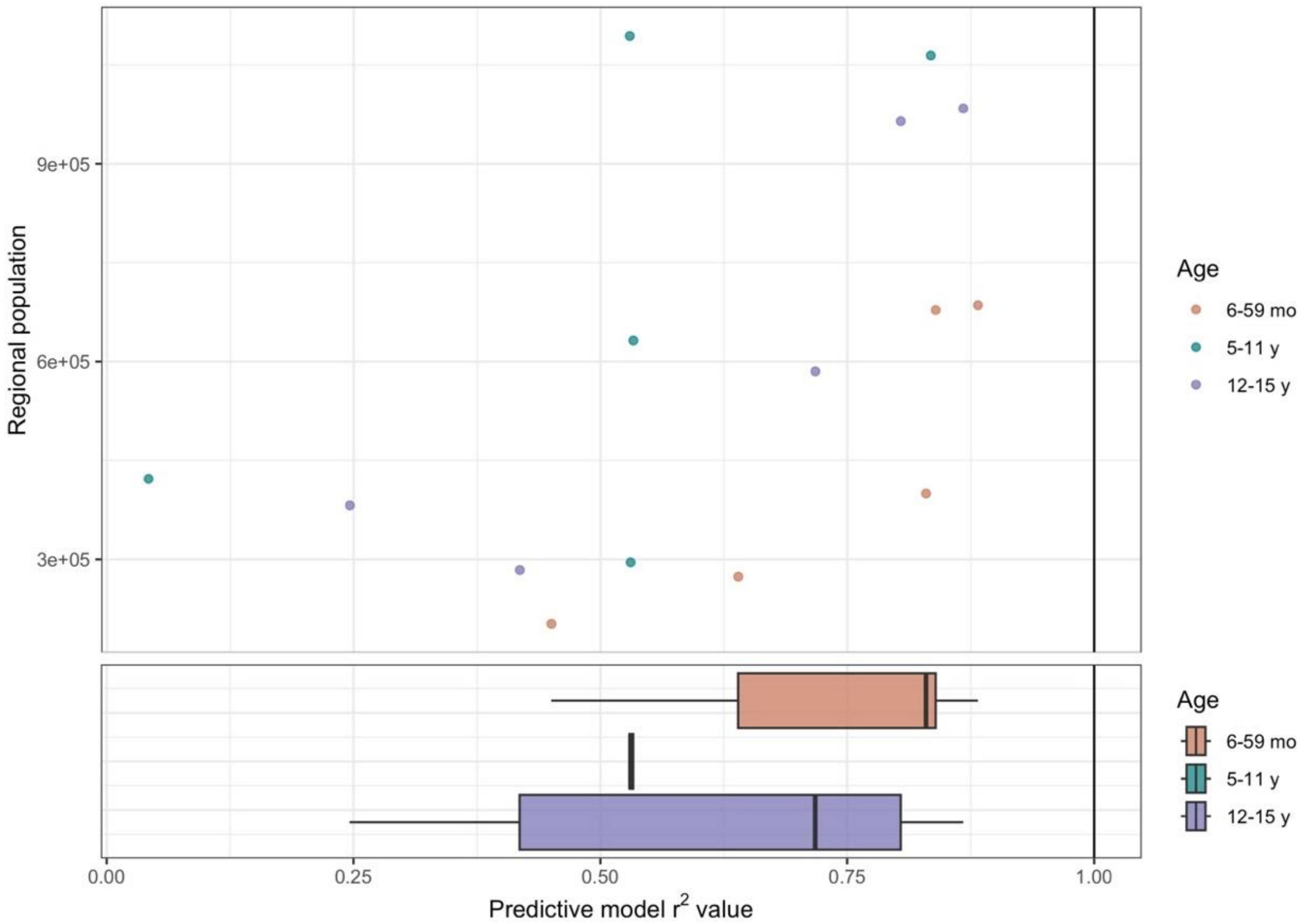

eFigure 4. Model performance of selected generalized linear model predicting hospitalizations during the pre-vaccine period for each region-age group combination. Top panel: R^2 values of the predictive models fit to pre-vaccine case data for each age group/region combination, plotted against the region population for the age group. Bottom panel: boxplot of the R^2 values from California's 5 regions. The black vertical line within the colored box is the median, the colored box spans the interquartile range, and horizontal lines denote the 95% CI.

© 2024 Head JR et al. *JAMA Network Open.*

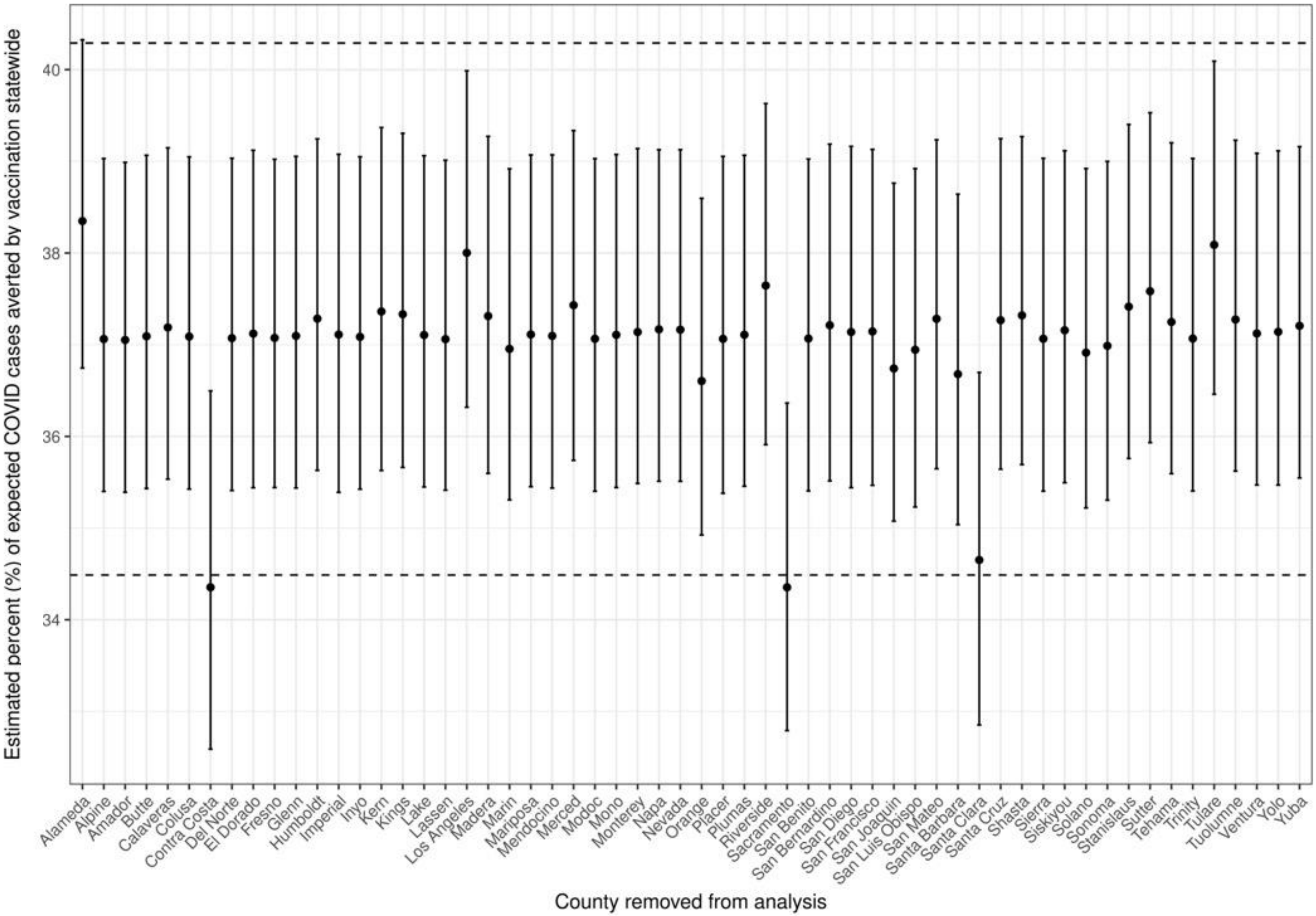

eFigure 5. Jackknife analysis for children 12-15 years. Points indicate the estimated percentage of cases among children 12-15 years old averted due to vaccination if the county on the x-axis had been dropped from the analysis. Vertical lines indicate 95% prediction intervals. Horizontal dashed lines indicate the statewide estimated percentage of cases among children 12-15 years old averted due to vaccination, with no counties excluded.

© 2024 Head JR et al. *JAMA Network Open.*

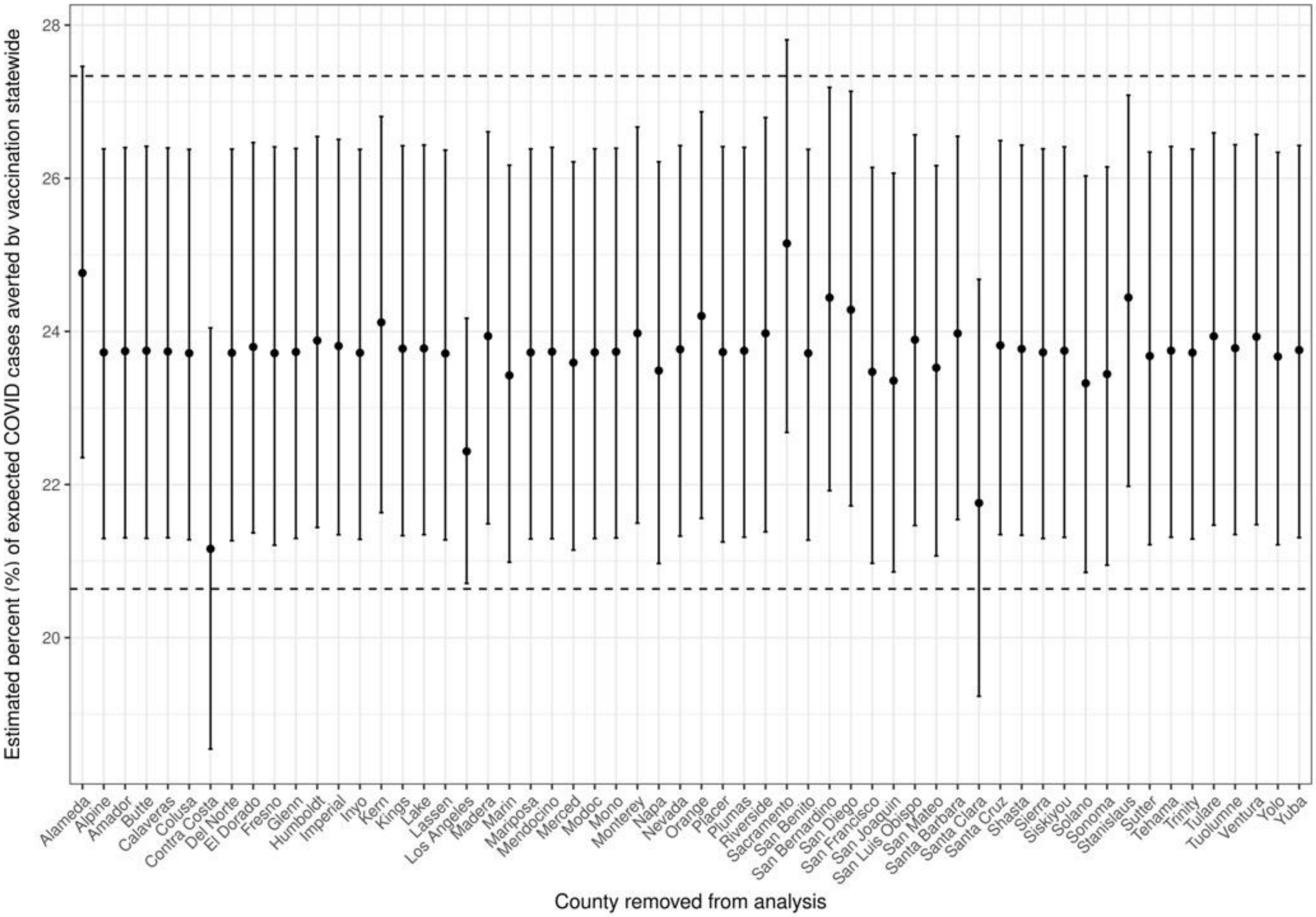

eFigure 6. Jackknife analysis for children 5-11 years. Points indicate the estimated percentage of cases among children 5-11 years old averted due to vaccination if the county on the x-axis had been dropped from the analysis. Vertical lines indicate 95% prediction intervals. Horizontal dashed lines indicate the statewide estimated percentage of cases among children 5-11 years old averted due to vaccination, with no counties excluded.

© 2024 Head JR et al. *JAMA Network Open.*

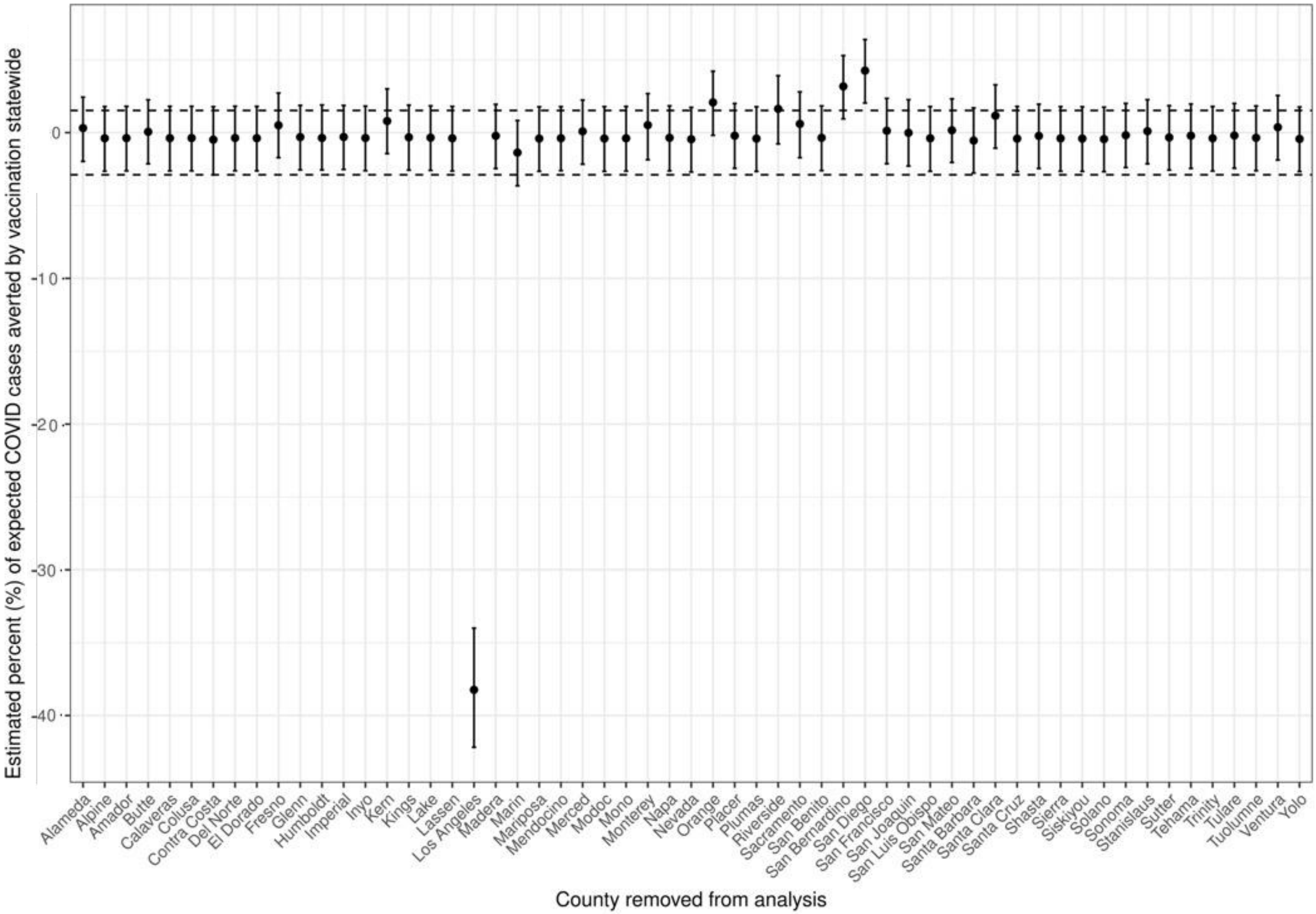

eFigure 7. Jackknife analysis for children 6-59 months. Points indicate the estimated percentage of cases among children 6-59 months old averted due to vaccination if the county on the x-axis had been dropped from the analysis. Vertical lines indicate 95% prediction intervals. Horizontal dashed lines indicate the statewide estimated percentage of cases among children 6-59 months old averted due to vaccination, with no counties excluded.

© 2024 Head JR et al. *JAMA Network Open.*

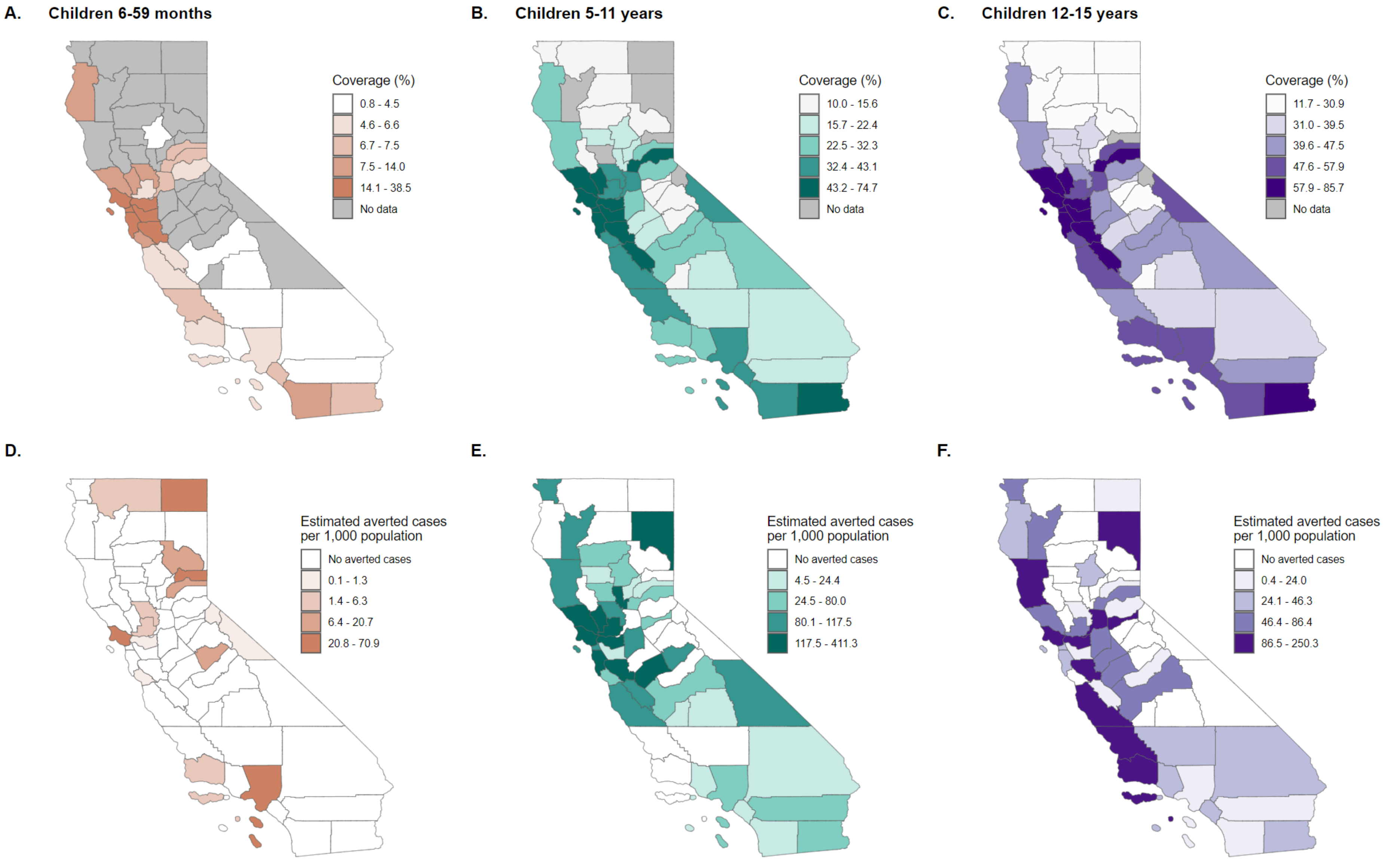

eFigure 8. Maps of pediatric COVID-19 cases averted and county-level pediatric vaccination coverage in California. Top row: vaccination coverage per county and age group achieved by the end of each age group's post-vaccination evaluation period (A) February 27, 2023 for children aged 5-69 months; B) June 19, 2022 for children aged 5-11 years; C) October 29, 2021 for adolescents aged 12-15 years). "No data" indicates that vaccination data was unavailable at the end of the age group's post-vaccination period. Bottom row: estimated number of cases averted per 1,000 population over the post-vaccine evaluation period of children aged D) 5-69 months, E) 5-11 years, and F) 12-15 years. Binning in maps D-I was done using quantiles.

© 2024 Head JR et al. *JAMA Network Open.*

eFigure 9. County-specific model predictions for COVID-19 cases among children aged 12-15 years. Black dots represent weekly hospitalization count, lines are model predictions, and shaded colored region is 95% prediction interval. Vertical lines are located at the time of vaccine eligibility.

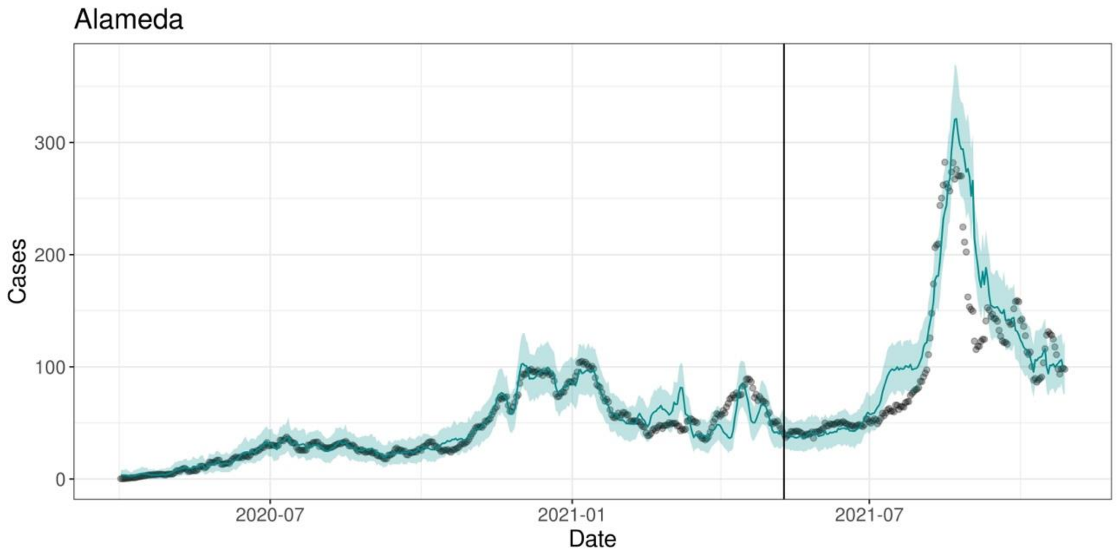

Model predictions for COVID-19 cases among children aged 12-15 years in Alameda County.

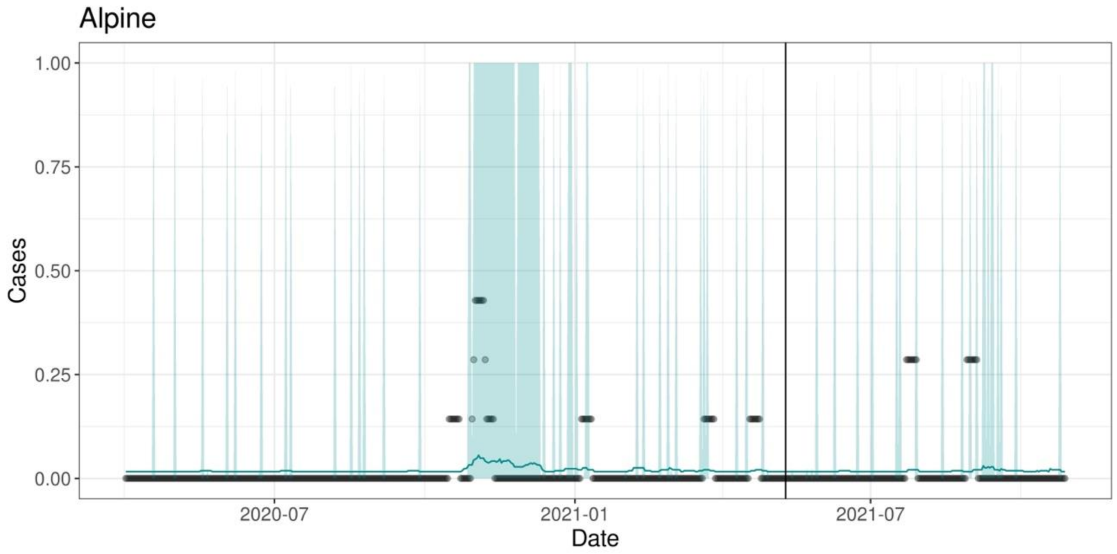

Model predictions for COVID-19 cases among children aged 12-15 years in Alpine County.

© 2024 Head JR et al. *JAMA Network Open.*

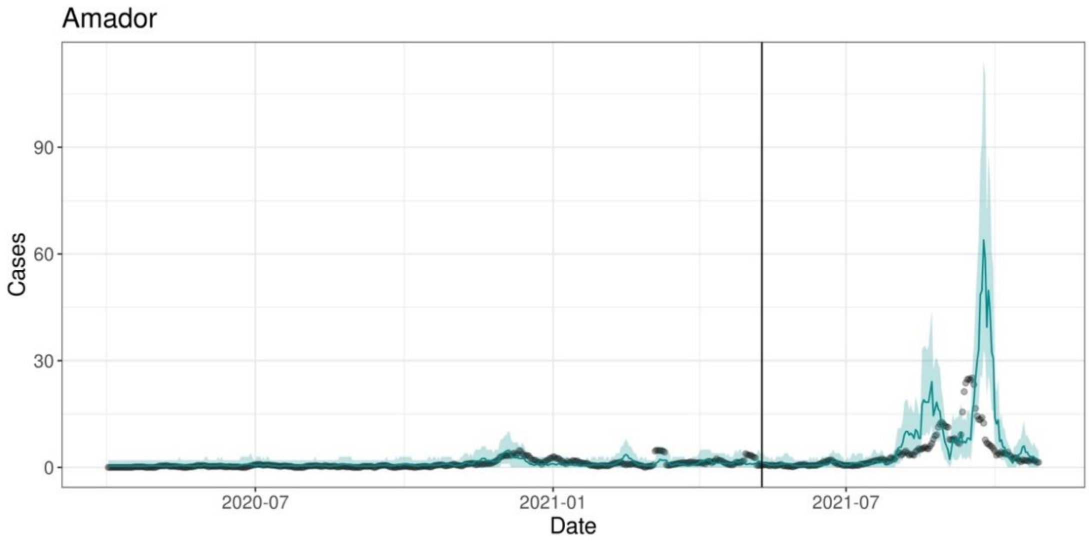

Model predictions for COVID-19 cases among children aged 12-15 years in Amador County.

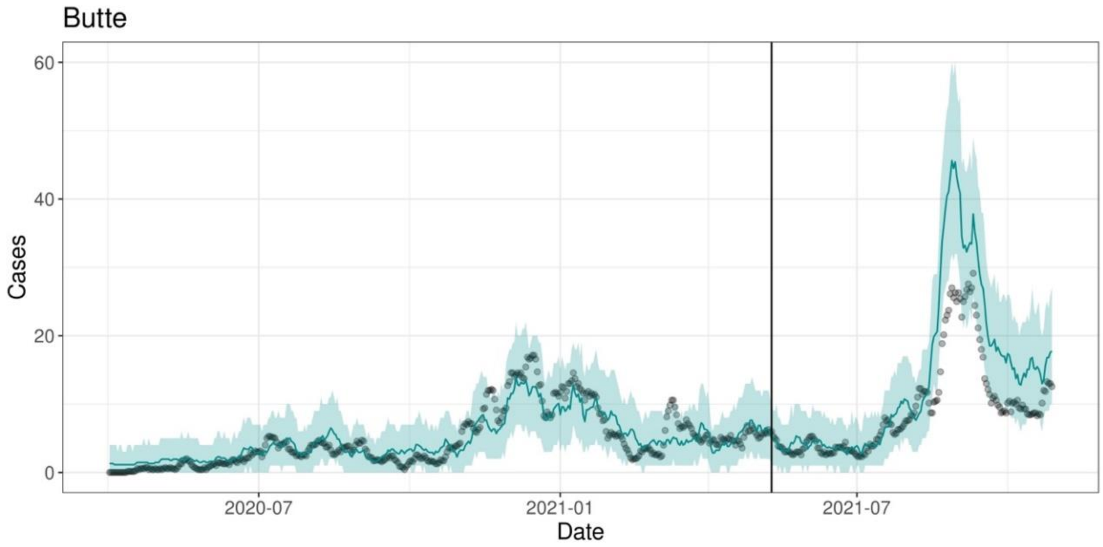

Model predictions for COVID-19 cases among children aged 12-15 years in Butte County.

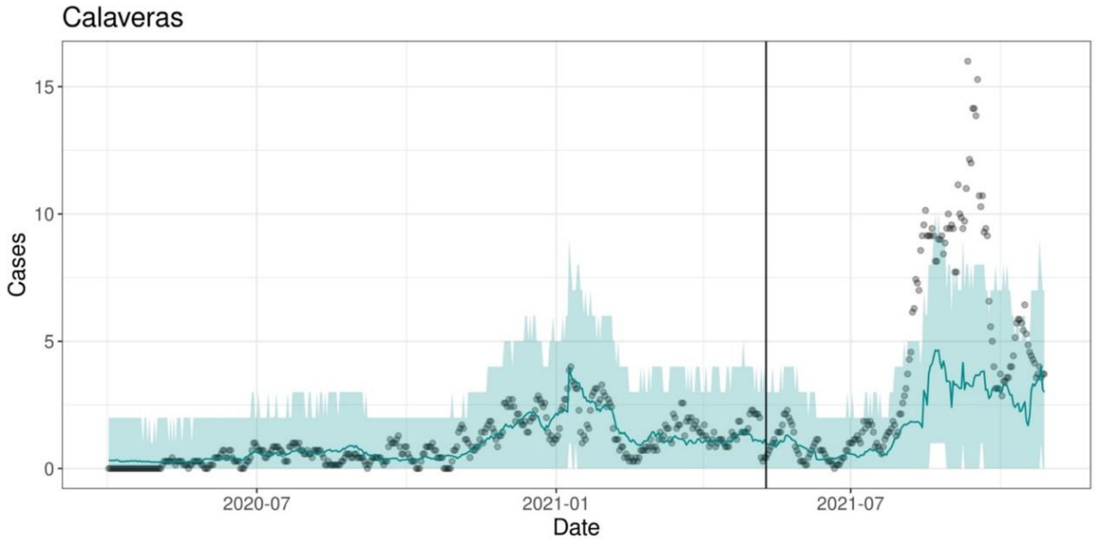

Model predictions for COVID-19 cases among children aged 12-15 years in Calaveras County.

© 2024 Head JR et al. *JAMA Network Open.*

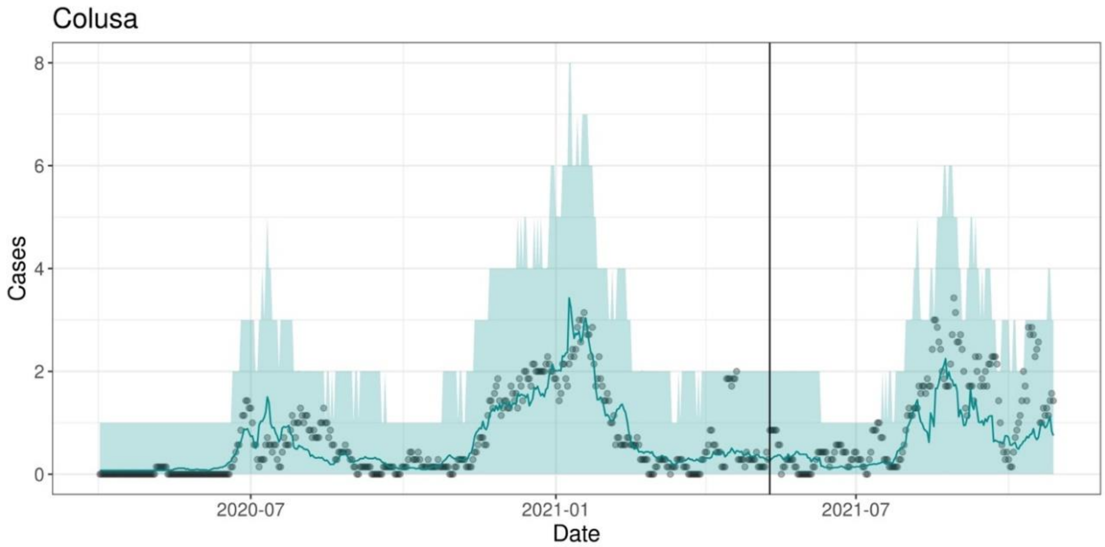

Model predictions for COVID-19 cases among children aged 12-15 years in Colusa County.

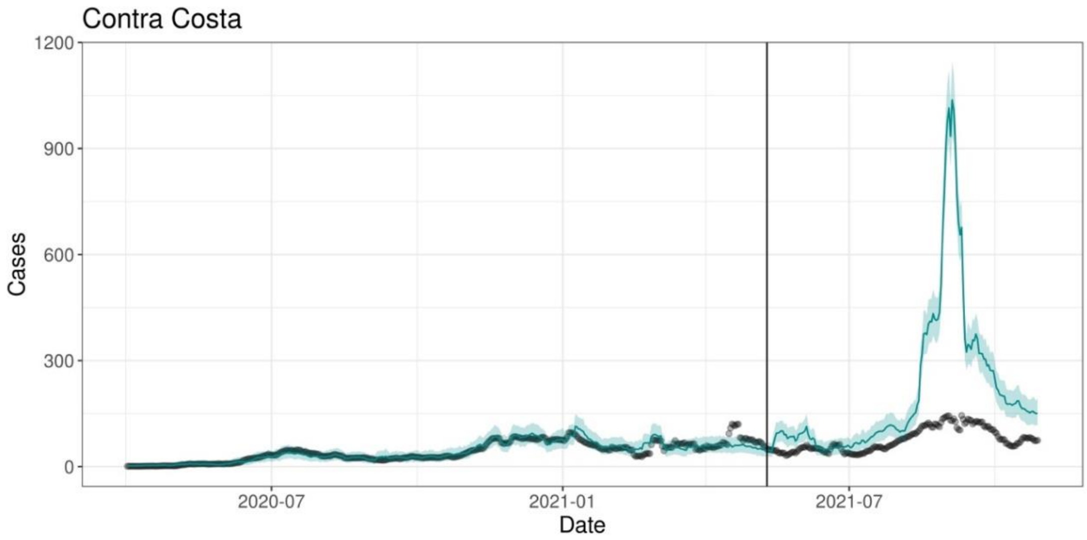

Model predictions for COVID-19 cases among children aged 12-15 years in Contra Costa County.

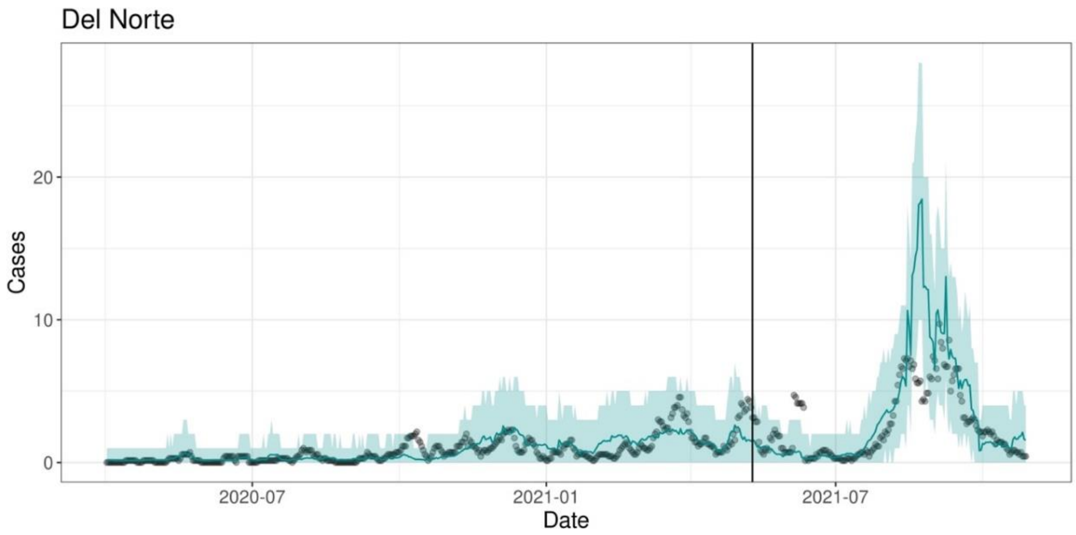

eFigure 16. Model predictions for COVID-19 cases among children aged 12-15 years in Del Norte County.

© 2024 Head JR et al. *JAMA Network Open.*

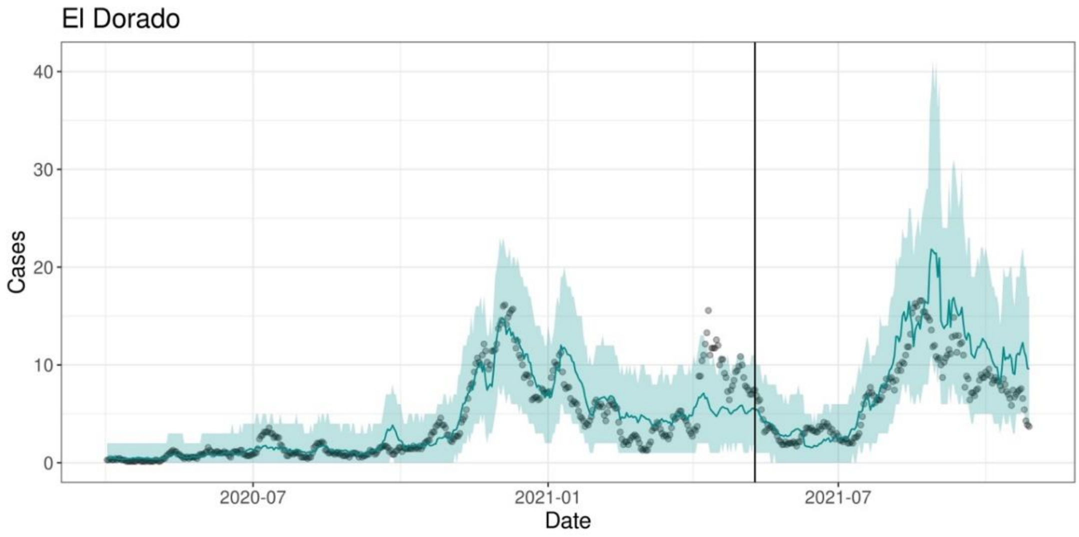

eFigure 17. Model predictions for COVID-19 cases among children aged 12-15 years in El Dorado County.

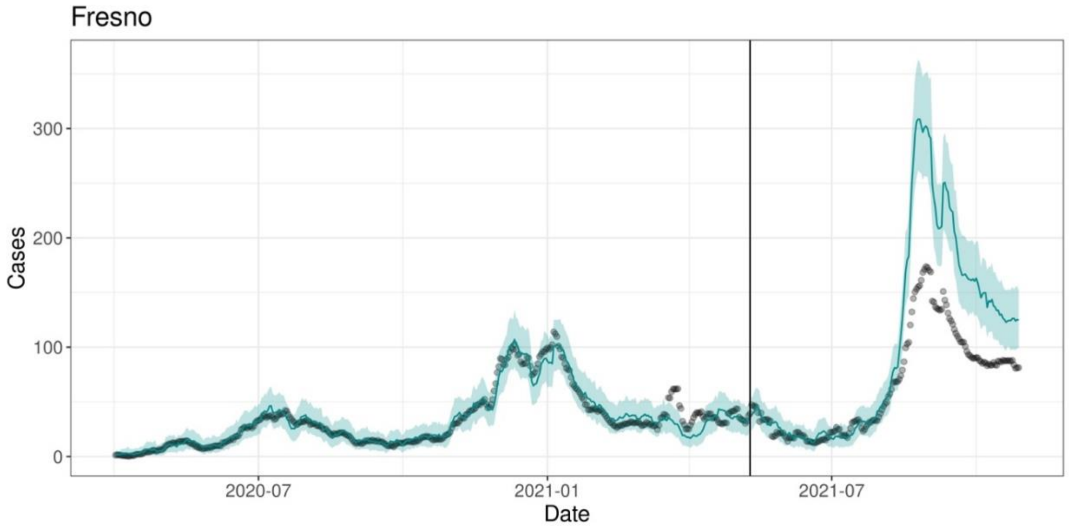

Model predictions for COVID-19 cases among children aged 12-15 years in Fresno County.

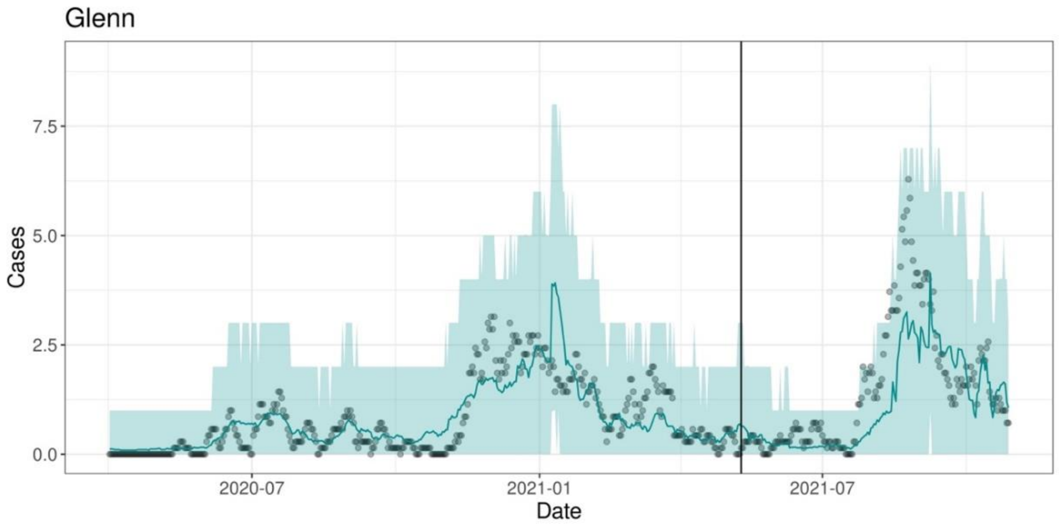

Model predictions for COVID-19 cases among children aged 12-15 years in Glenn County.

© 2024 Head JR et al. *JAMA Network Open.*

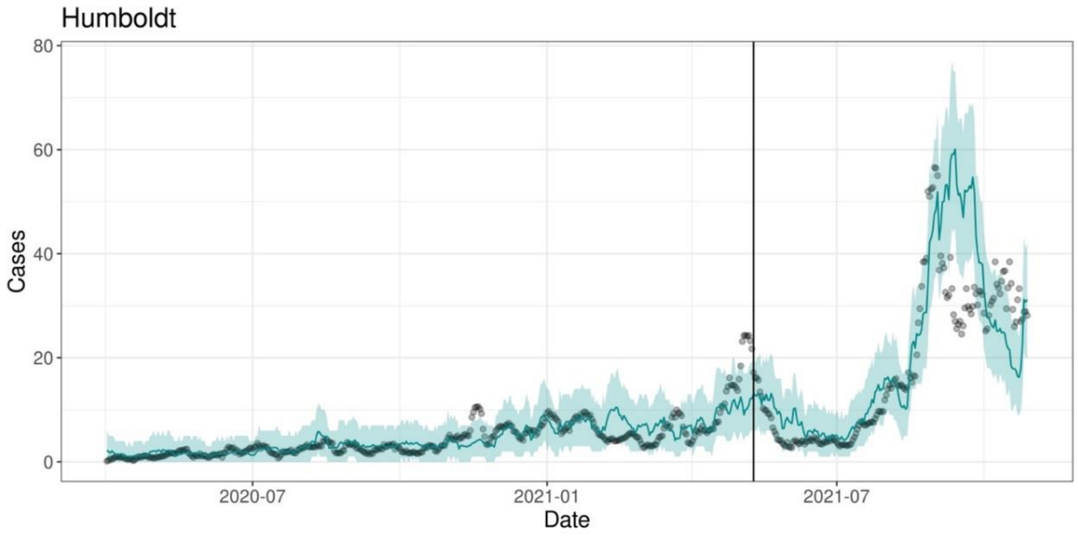

Model predictions for COVID-19 cases among children aged 12-15 years in Humboldt County.

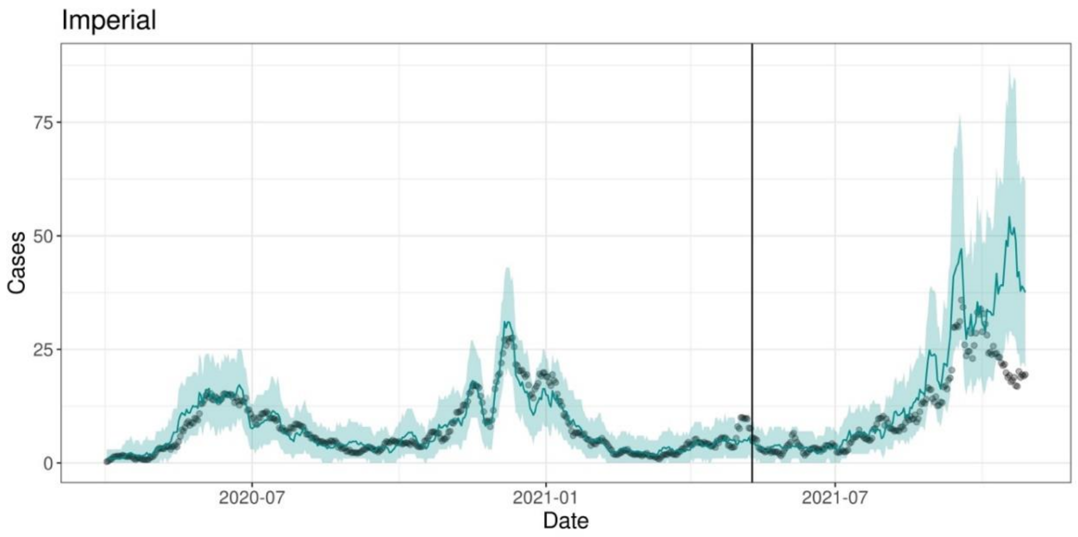

Model predictions for COVID-19 cases among children aged 12-15 years in Imperial County.

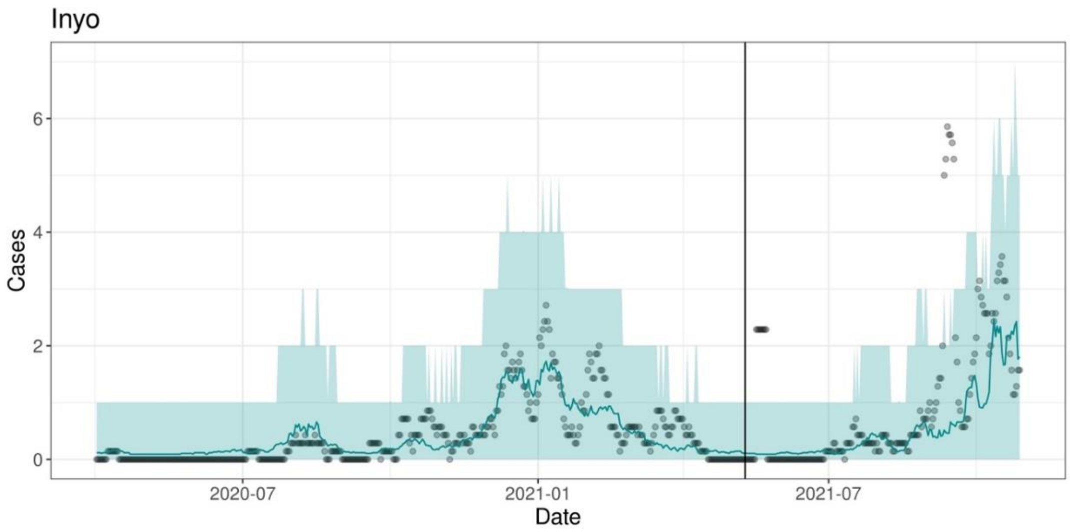

Model predictions for COVID-19 cases among children aged 12-15 years in Inyo County.

© 2024 Head JR et al. *JAMA Network Open.*

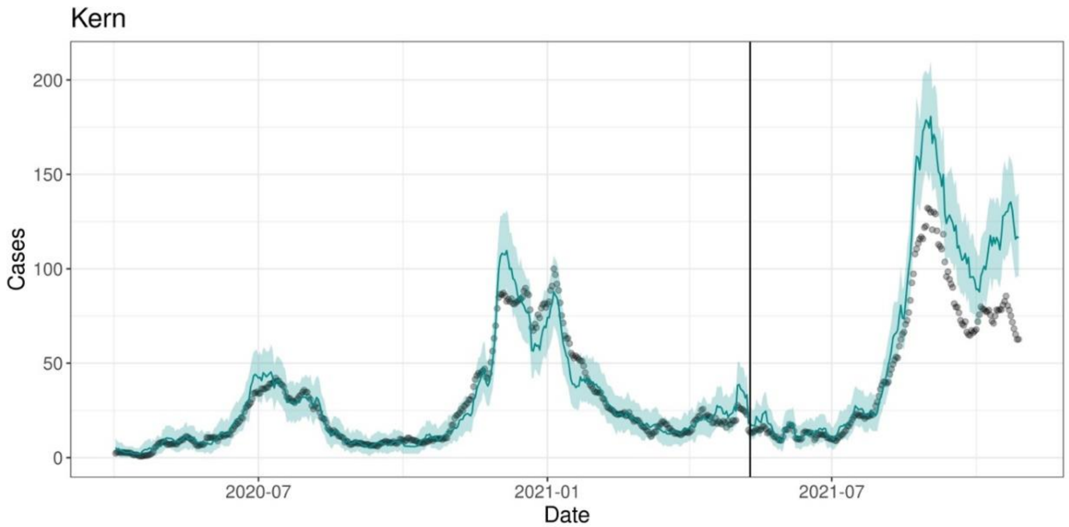

Model predictions for COVID-19 cases among children aged 12-15 years in Kern County.

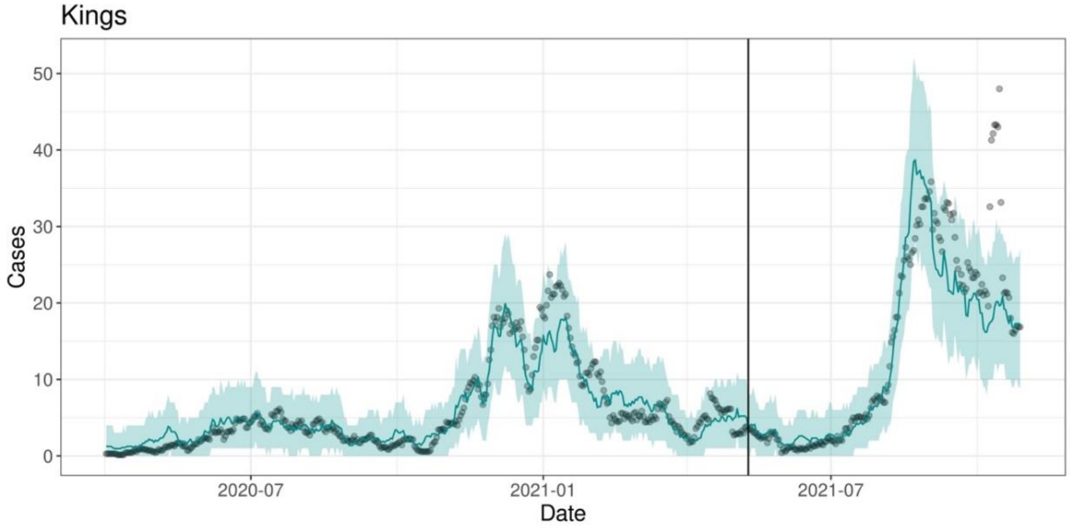

Model predictions for COVID-19 cases among children aged 12-15 years in Kings County.

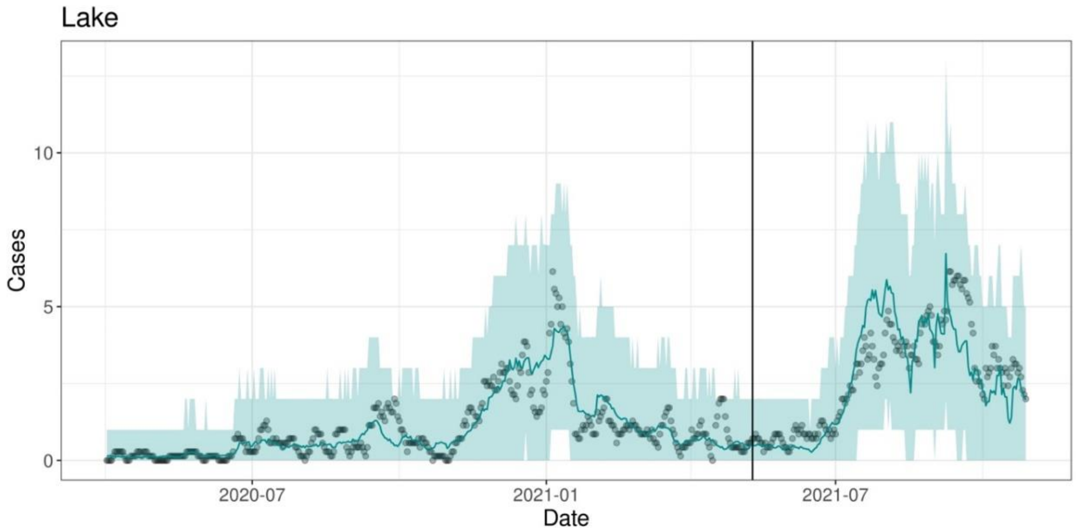

Model predictions for COVID-19 cases among children aged 12-15 years in Lake County.

© 2024 Head JR et al. *JAMA Network Open.*

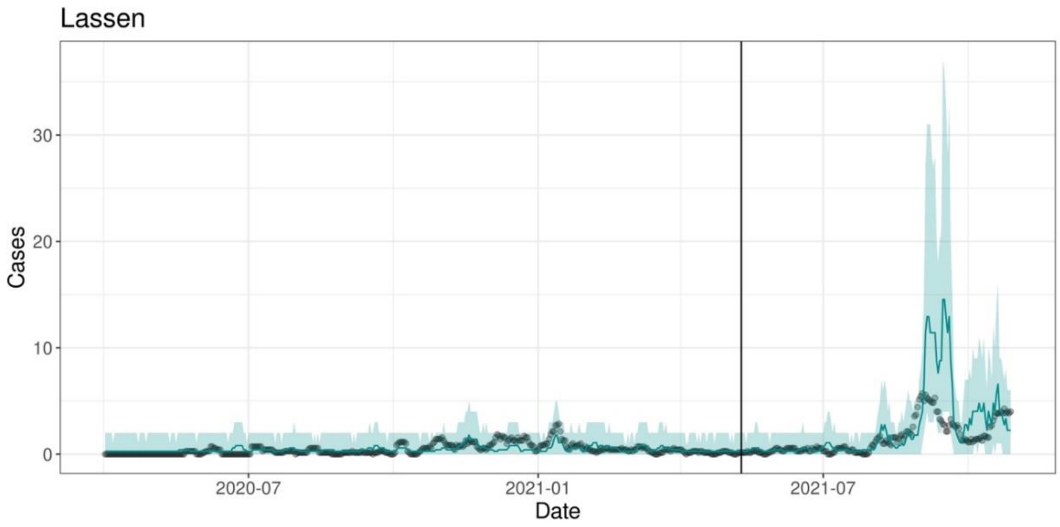

Model predictions for COVID-19 cases among children aged 12-15 years in Lassen County.

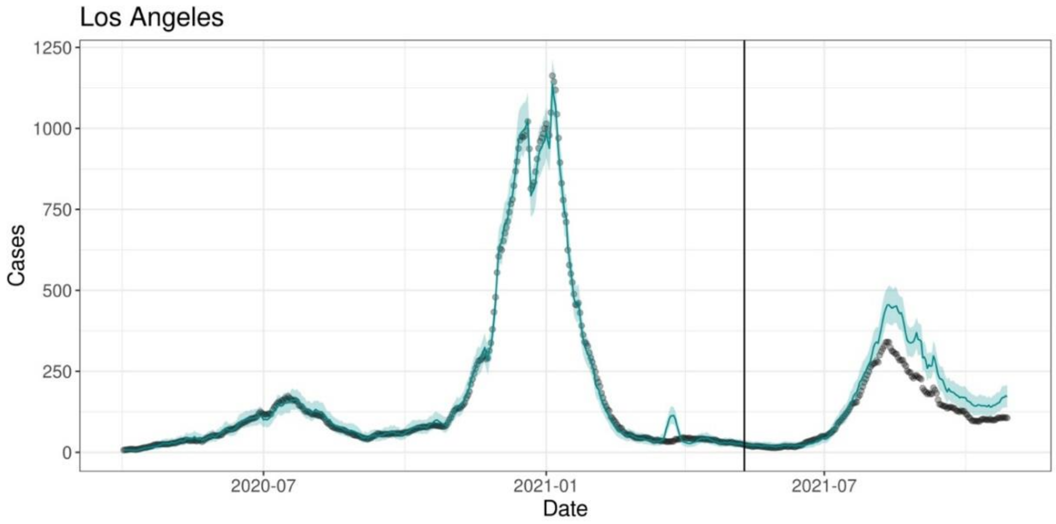

Model predictions for COVID-19 cases among children aged 12-15 years in Los Angeles County.

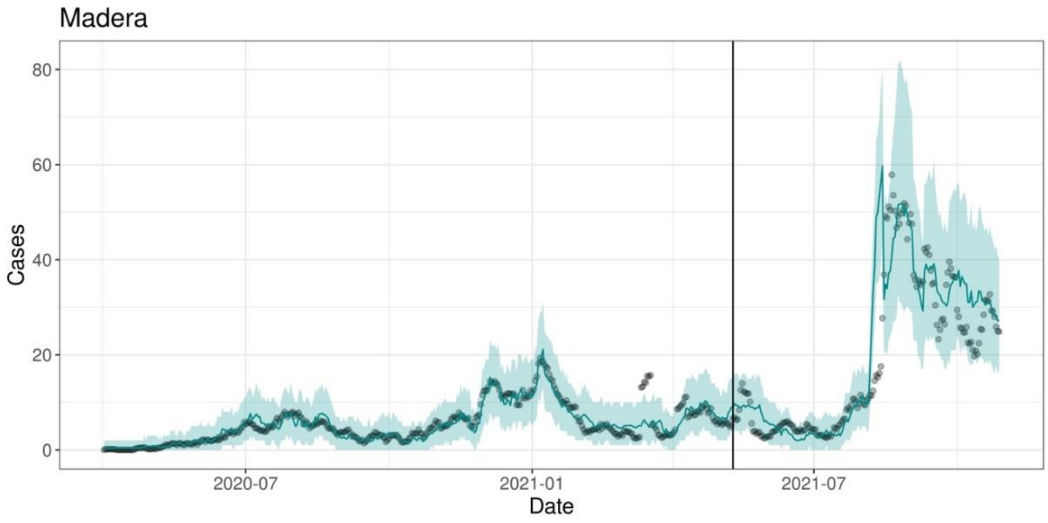

Model predictions for COVID-19 cases among children aged 12-15 years in Madera County.

© 2024 Head JR et al. *JAMA Network Open.*

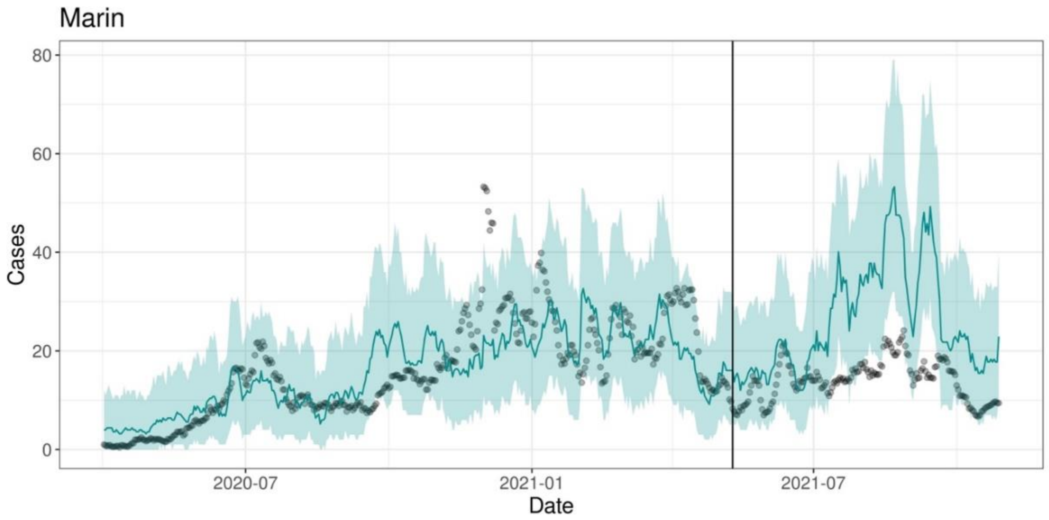

Model predictions for COVID-19 cases among children aged 12-15 years in Marin County.

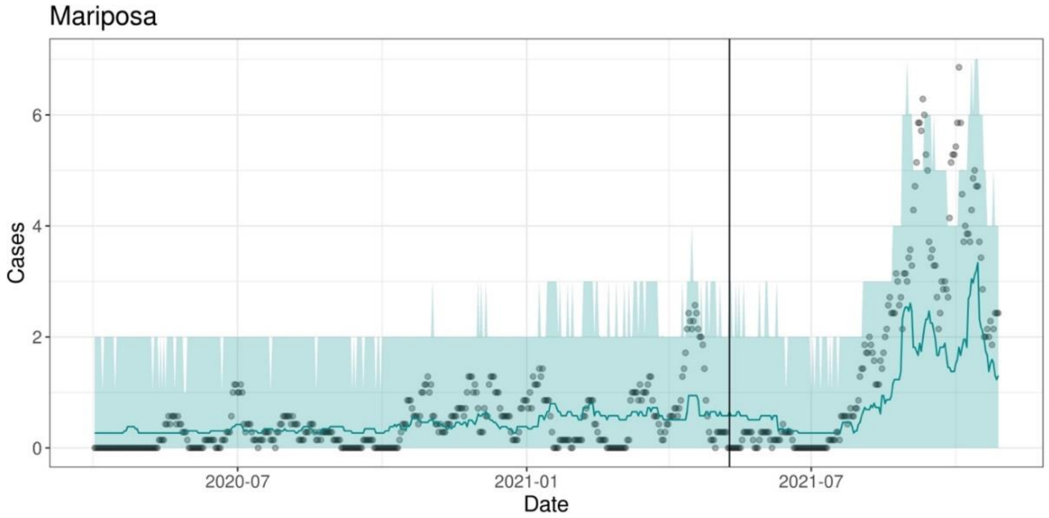

Model predictions for COVID-19 cases among children aged 12-15 years in Mariposa County.

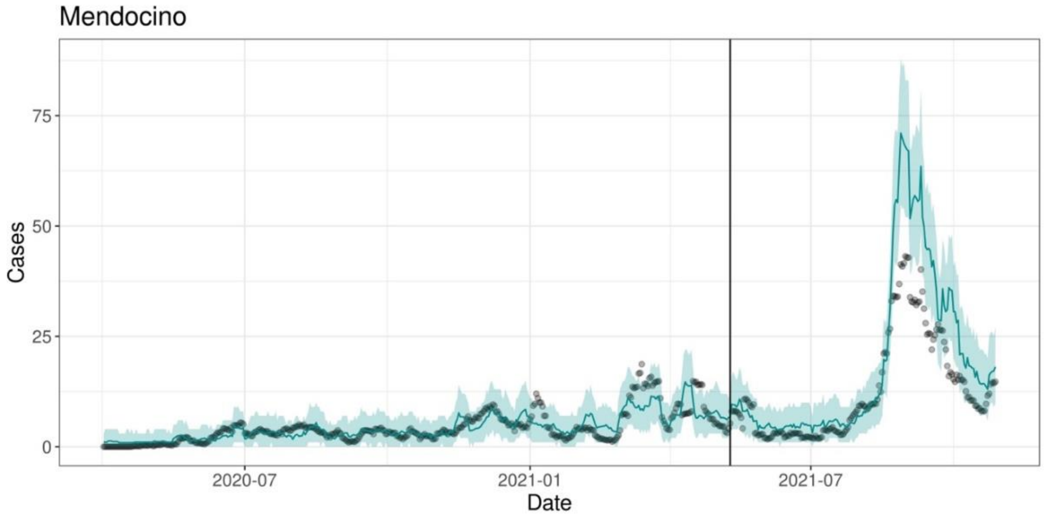

Model predictions for COVID-19 cases among children aged 12-15 years in Mendocino County.

© 2024 Head JR et al. *JAMA Network Open.*

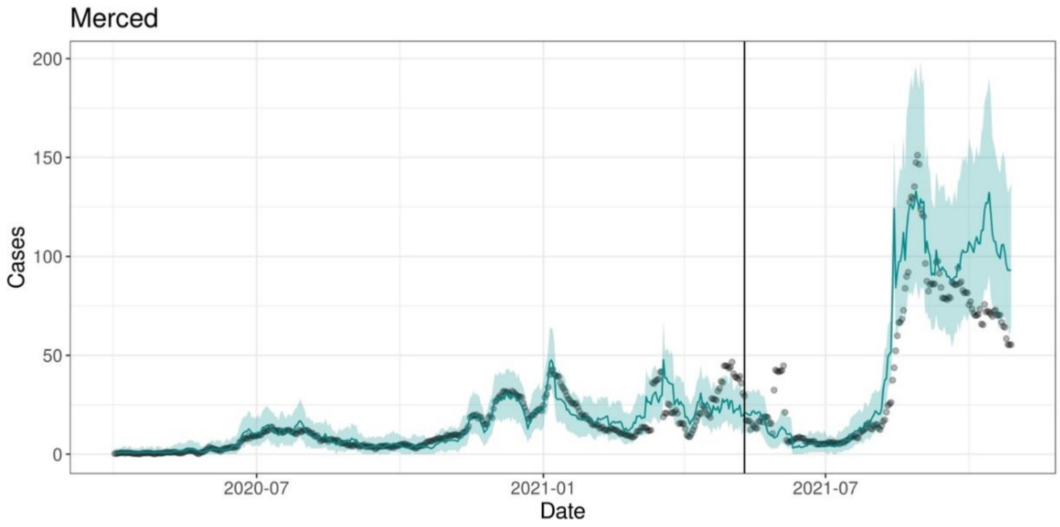

Model predictions for COVID-19 cases among children aged 12-15 years in Merced County.

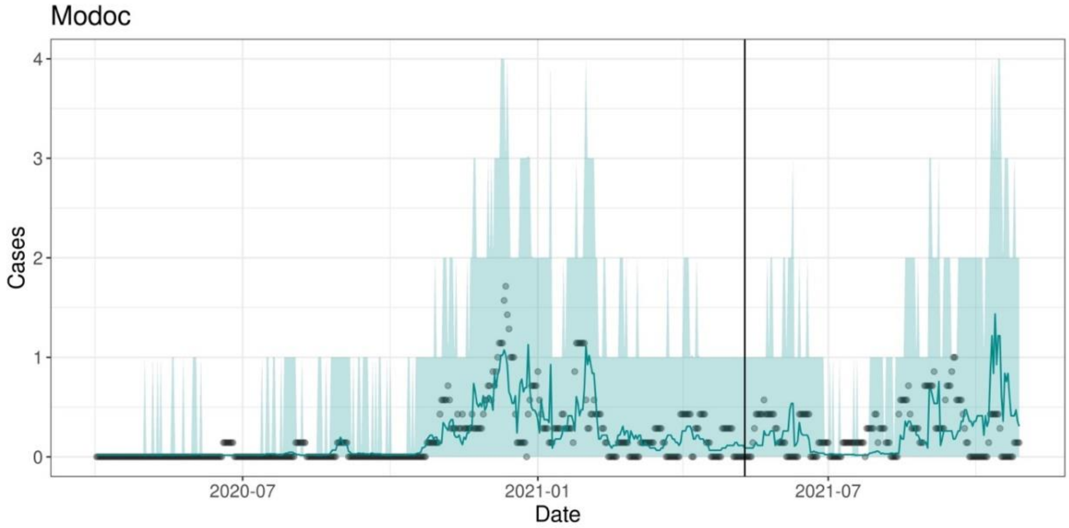

eFigure 33. Model predictions for COVID-19 cases among children aged 12-15 years in Modoc County.

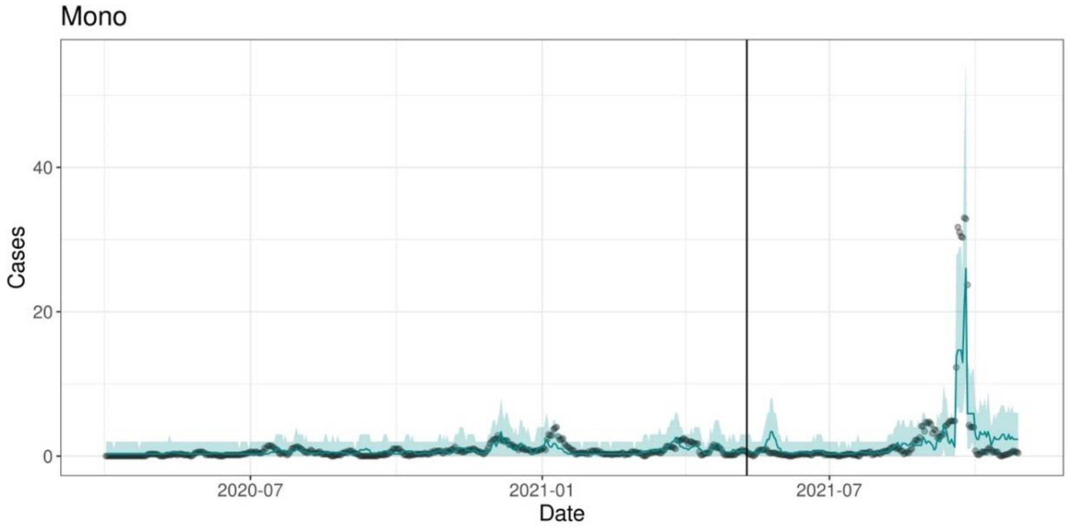

Model predictions for COVID-19 cases among children aged 12-15 years in Mono County.

© 2024 Head JR et al. *JAMA Network Open.*

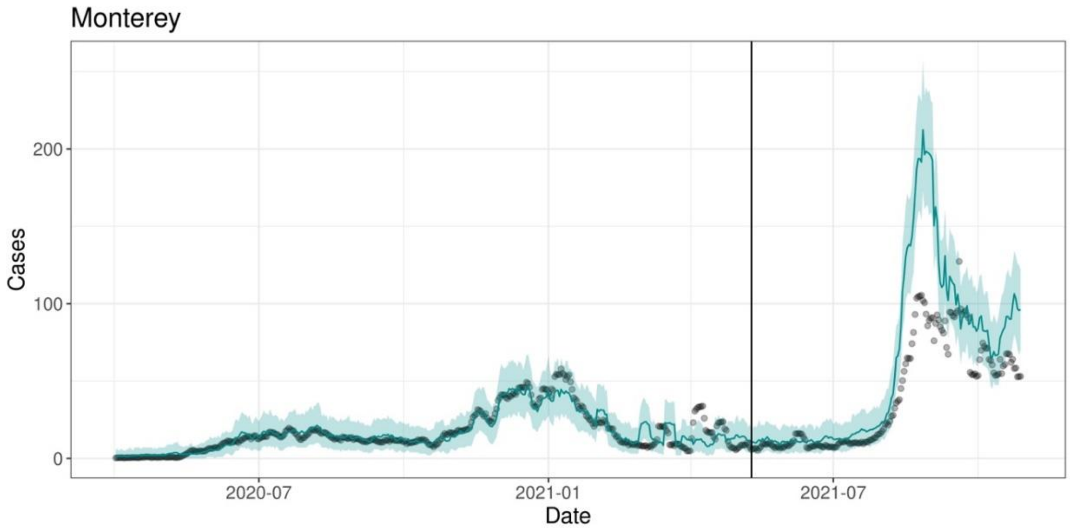

Model predictions for COVID-19 cases among children aged 12-15 years in Monterey County.

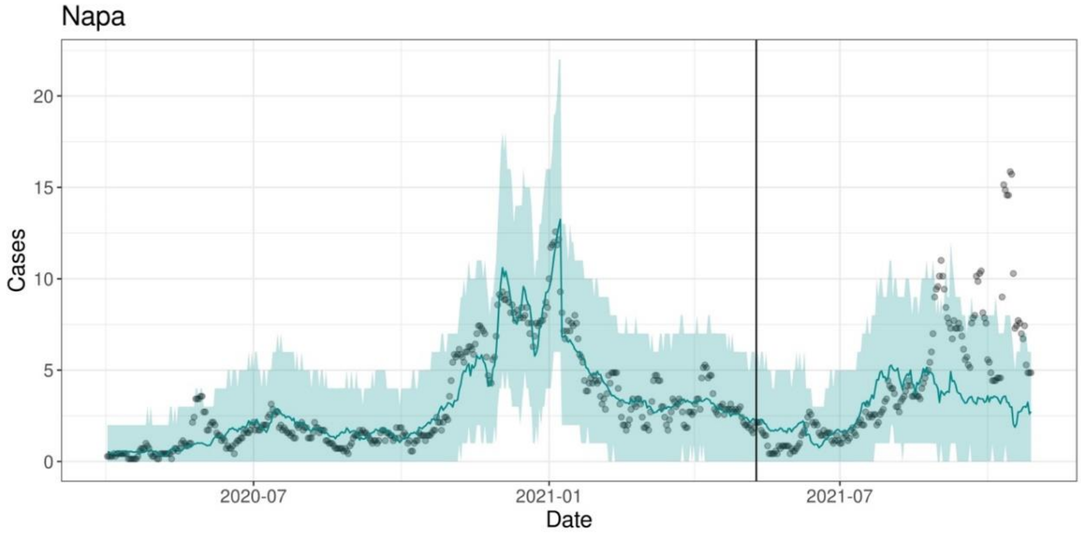

Model predictions for COVID-19 cases among children aged 12-15 years in Napa County.

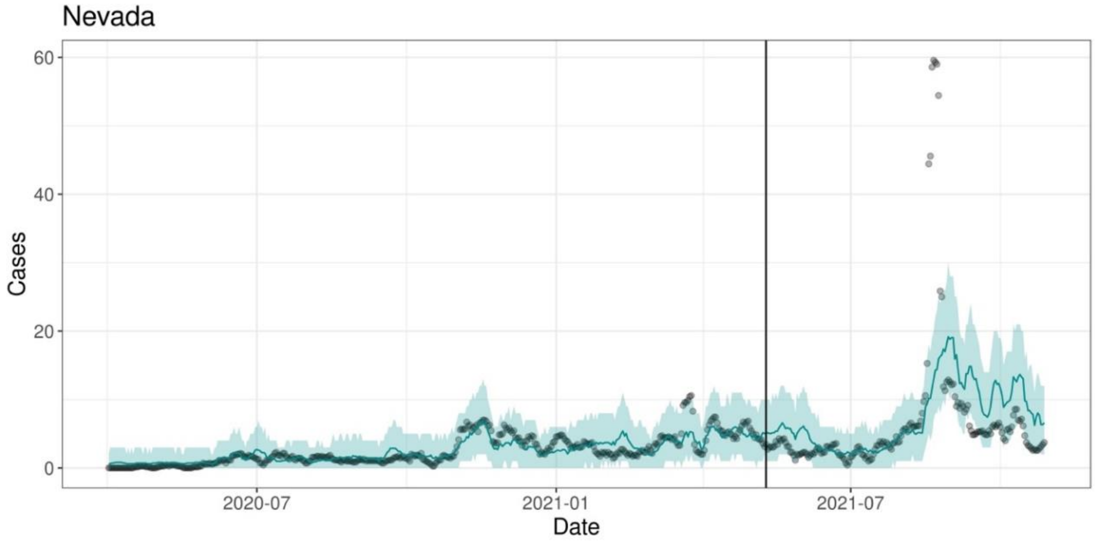

Model predictions for COVID-19 cases among children aged 12-15 years in Nevada County.

© 2024 Head JR et al. *JAMA Network Open.*

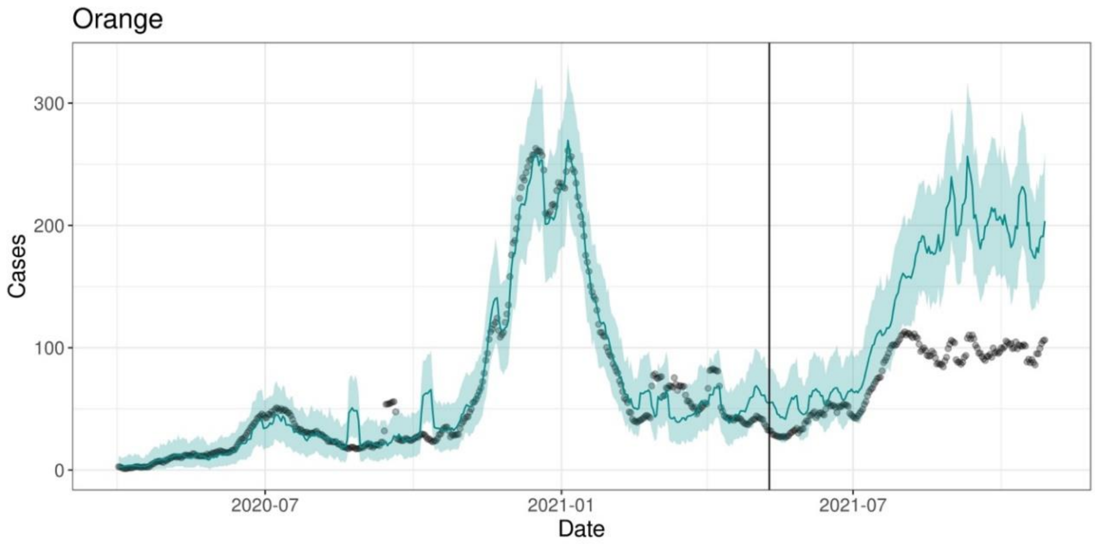

Model predictions for COVID-19 cases among children aged 12-15 years in Orange County.

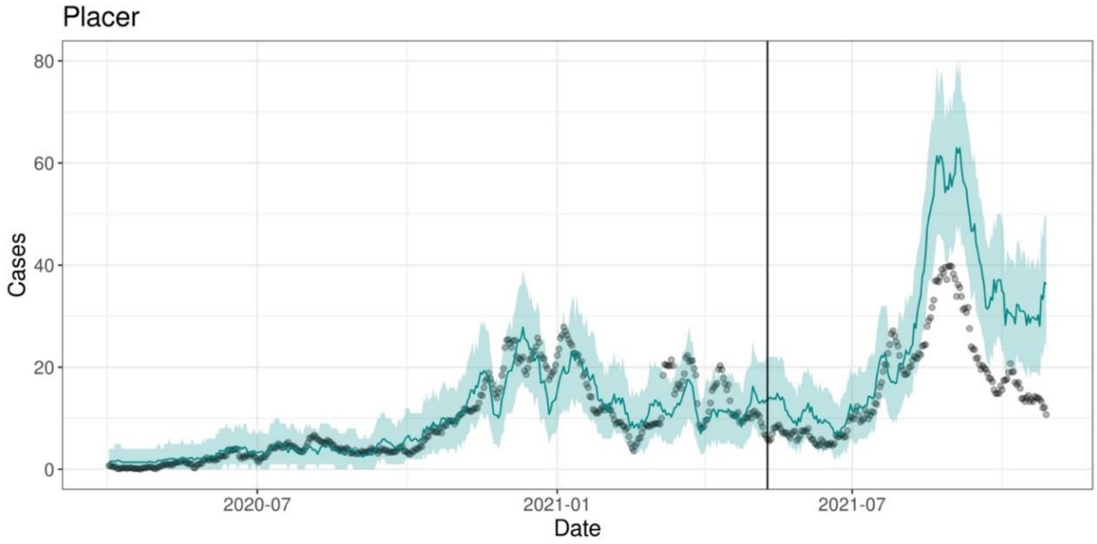

Model predictions for COVID-19 cases among children aged 12-15 years in Placer County.

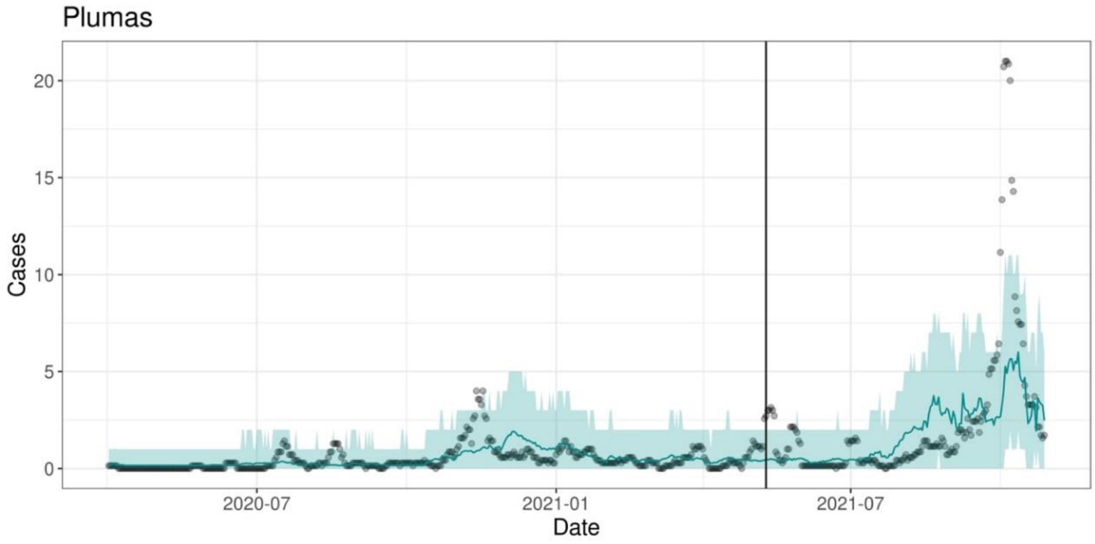

Model predictions for COVID-19 cases among children aged 12-15 years in Plumas County.

© 2024 Head JR et al. *JAMA Network Open.*

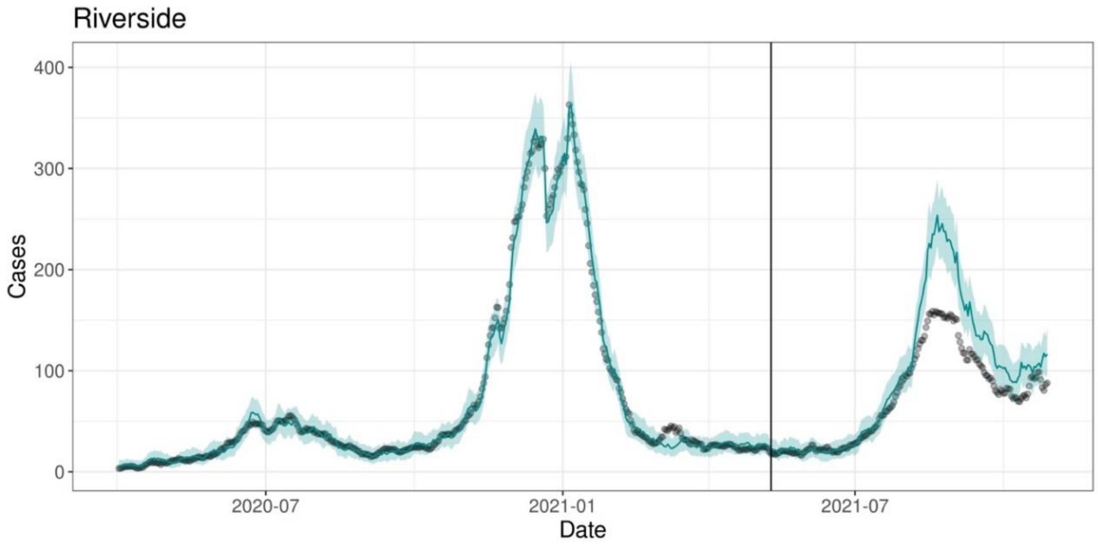

Model predictions for COVID-19 cases among children aged 12-15 years in Riverside County.

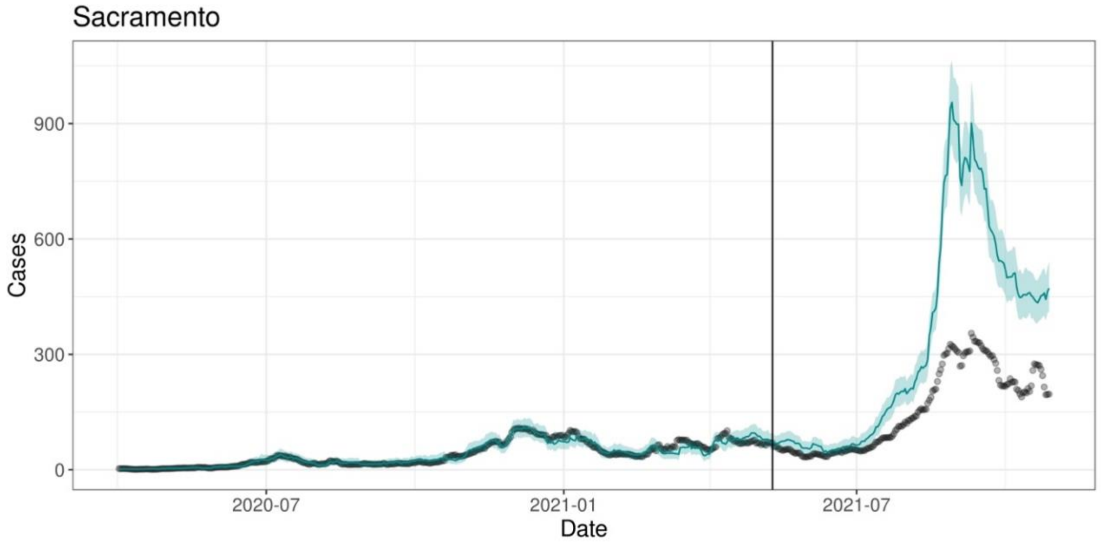

Model predictions for COVID-19 cases among children aged 12-15 years in Sacramento County.

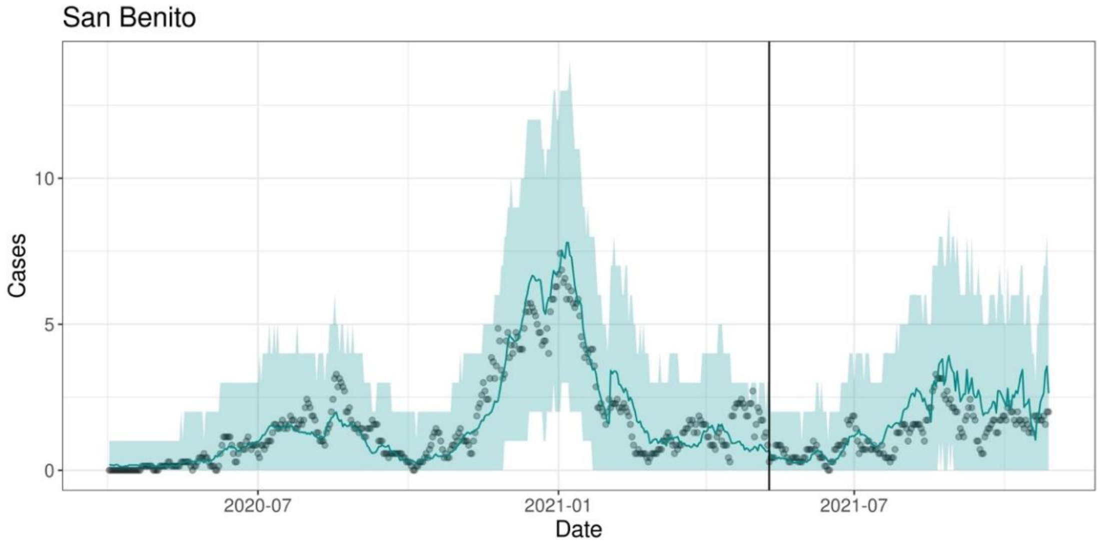

Model predictions for COVID-19 cases among children aged 12-15 years in San Benito County.

© 2024 Head JR et al. *JAMA Network Open.*

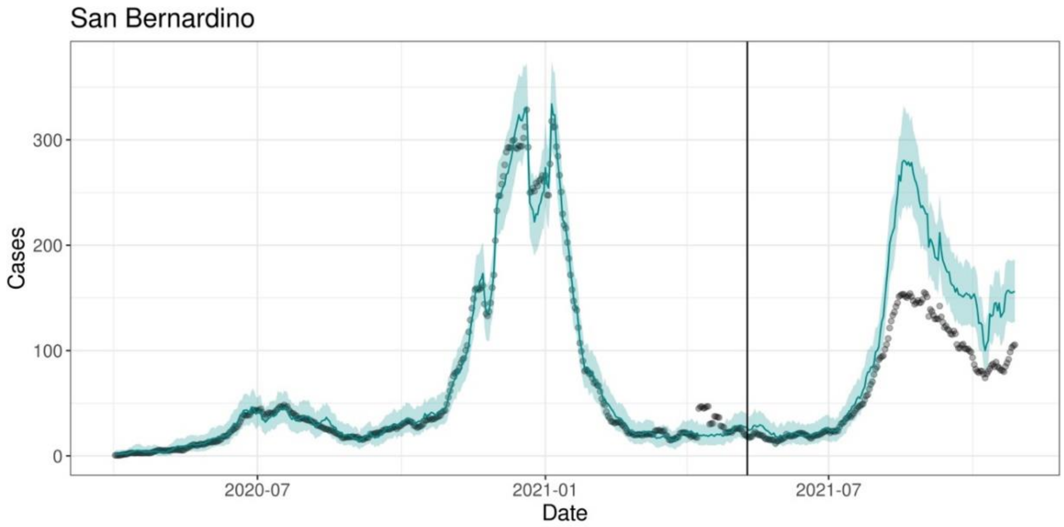

Model predictions for COVID-19 cases among children aged 12-15 years in San Bernadino County.

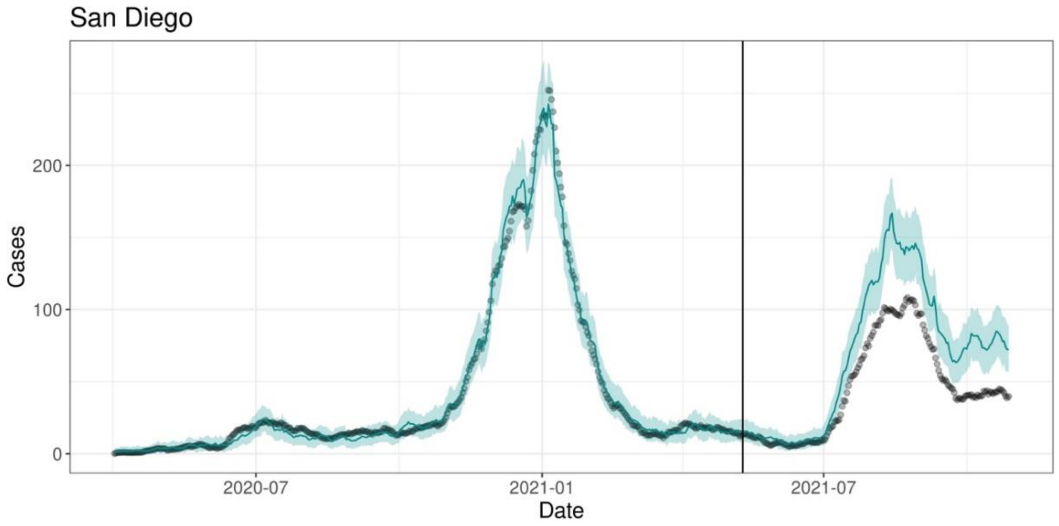

Model predictions for COVID-19 cases among children aged 12-15 years in San Diego County.

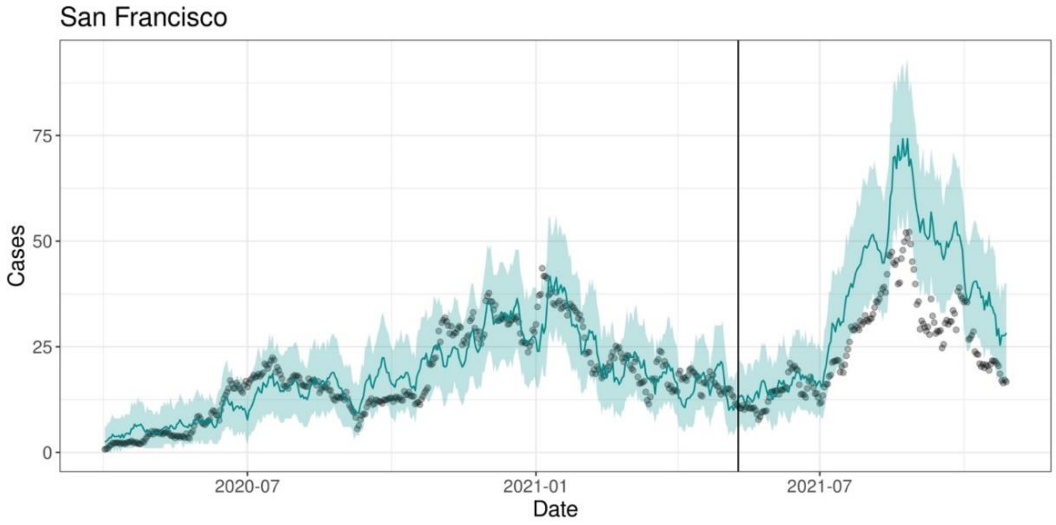

Model predictions for COVID-19 cases among children aged 12-15 years in San Francisco County.

© 2024 Head JR et al. *JAMA Network Open.*

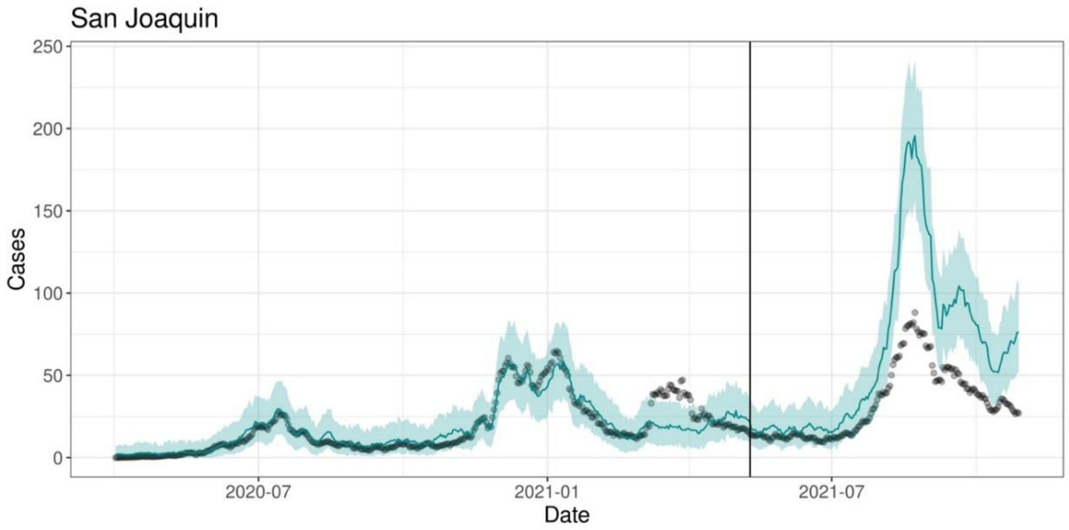

Model predictions for COVID-19 cases among children aged 12-15 years in San Joaquin County.

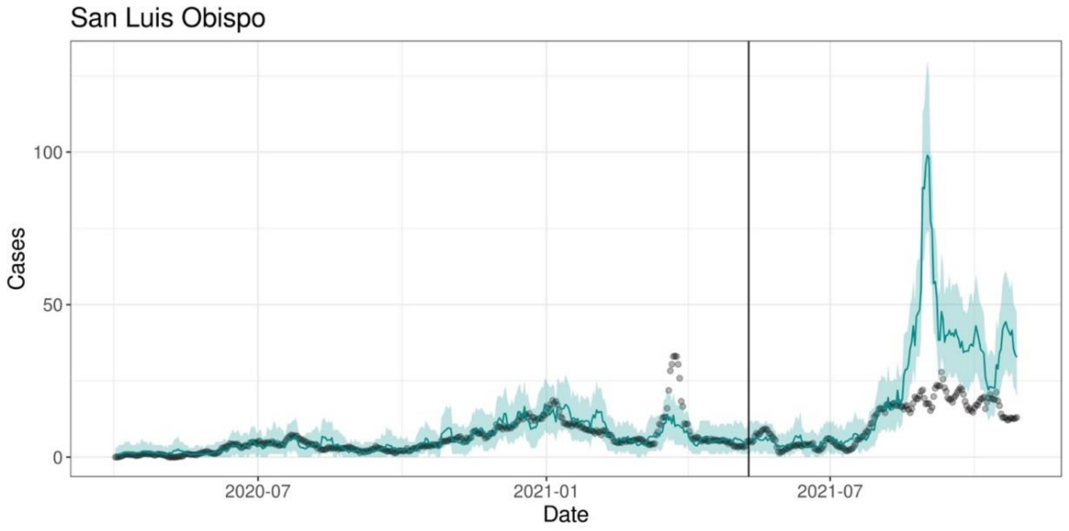

Model predictions for COVID-19 cases among children aged 12-15 years in San Luis Obispo County.

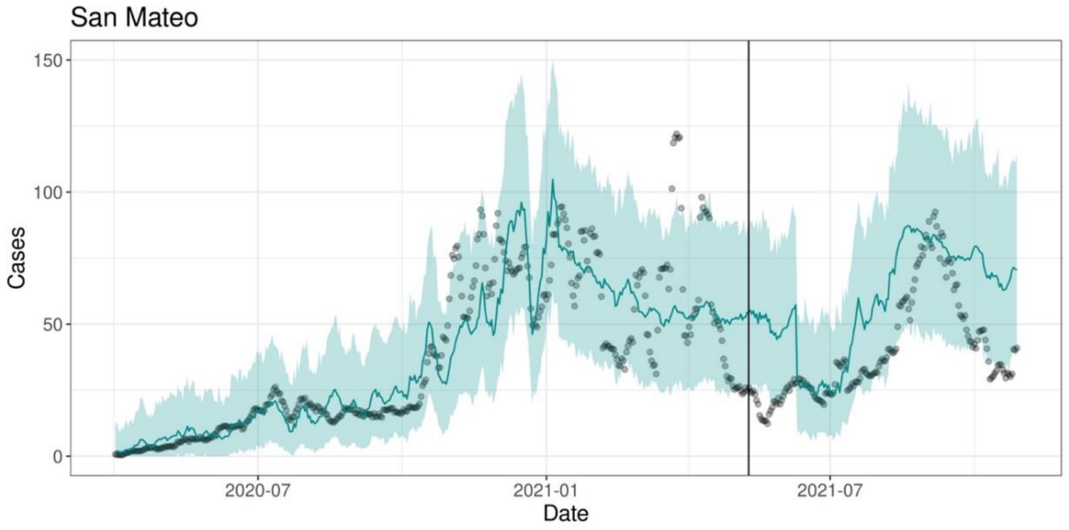

Model predictions for COVID-19 cases among children aged 12-15 years in San Mateo County.

© 2024 Head JR et al. *JAMA Network Open.*

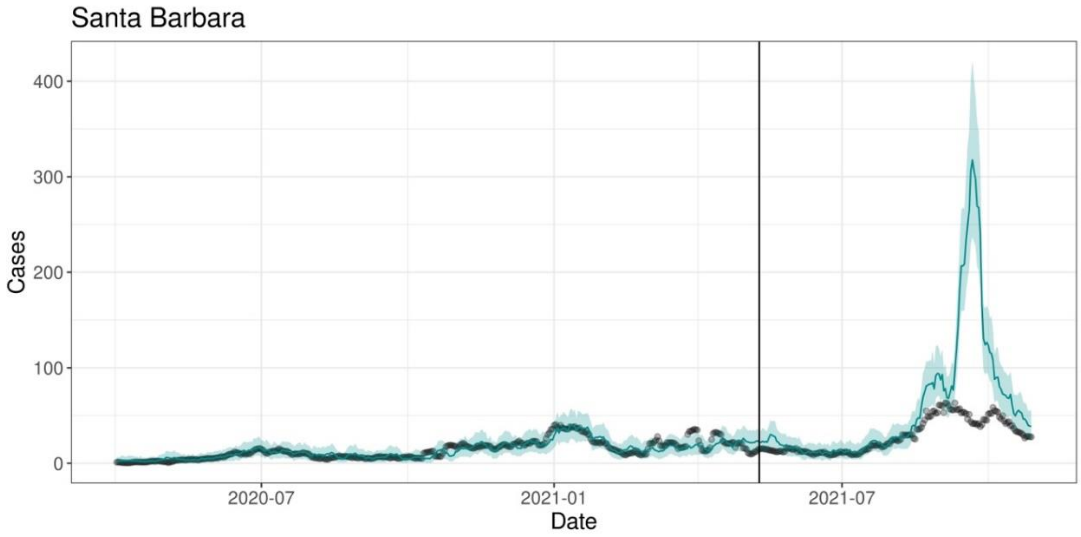

Model predictions for COVID-19 cases among children aged 12-15 years in Santa Barbara County.

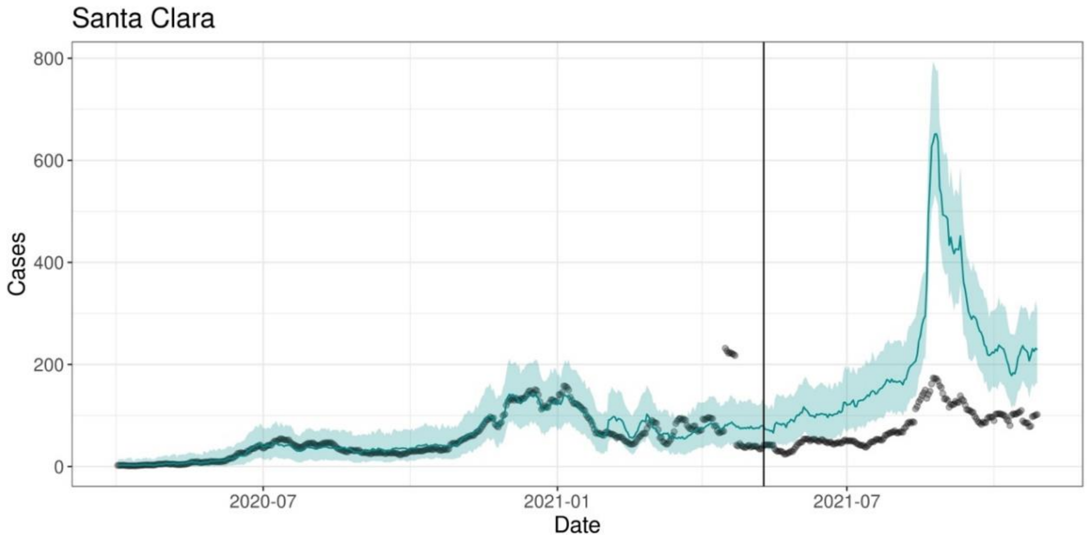

Model predictions for COVID-19 cases among children aged 12-15 years in Santa Clara County.

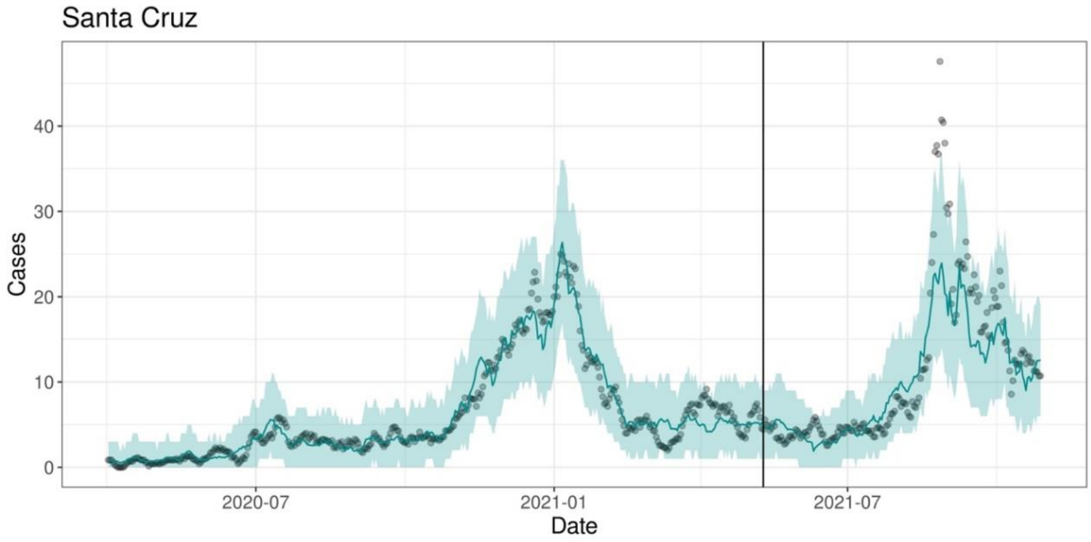

Model predictions for COVID-19 cases among children aged 12-15 years in Santa Cruz County.

© 2024 Head JR et al. *JAMA Network Open.*

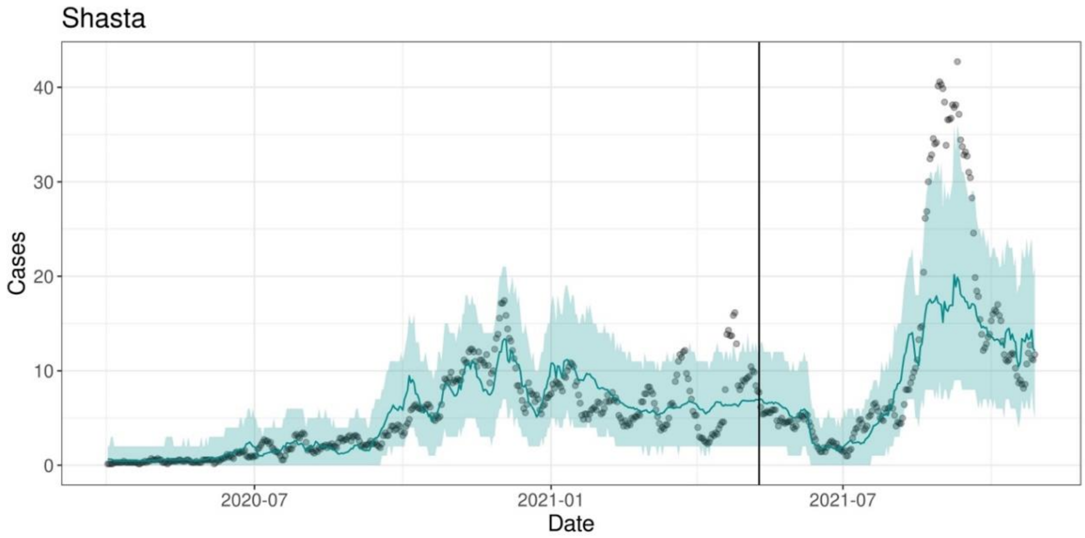

Model predictions for COVID-19 cases among children aged 12-15 years in Shasta County.

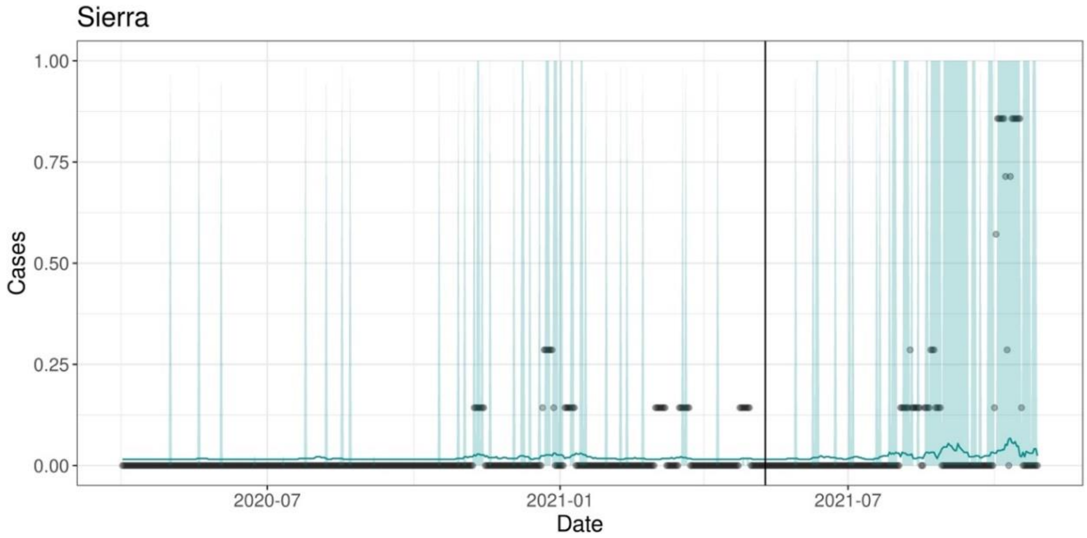

Model predictions for COVID-19 cases among children aged 12-15 years in Sierra County.

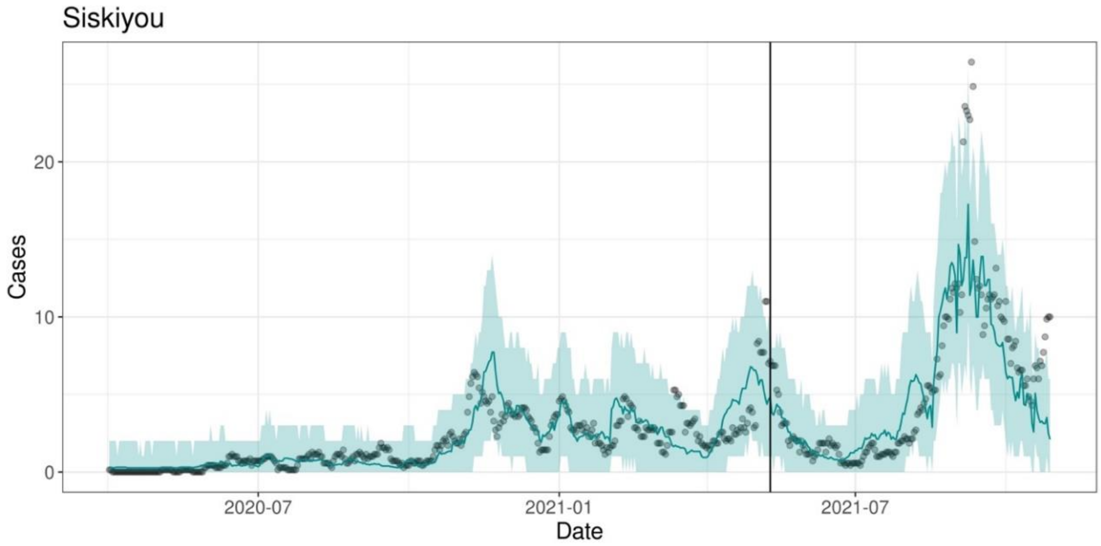

Model predictions for COVID-19 cases among children aged 12-15 years in Siskiyou County.

© 2024 Head JR et al. *JAMA Network Open.*

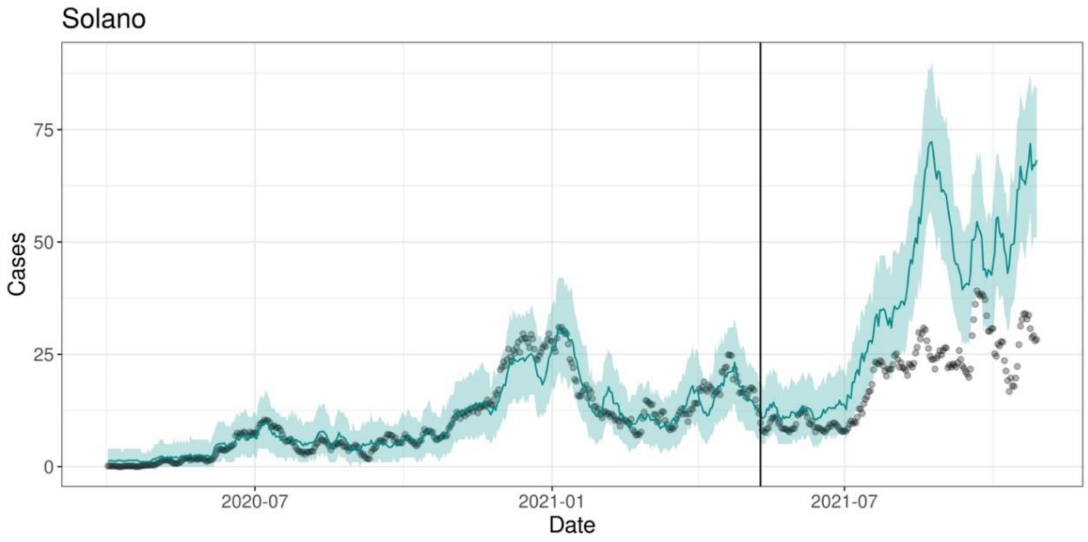

Model predictions for COVID-19 cases among children aged 12-15 years in Solano County.

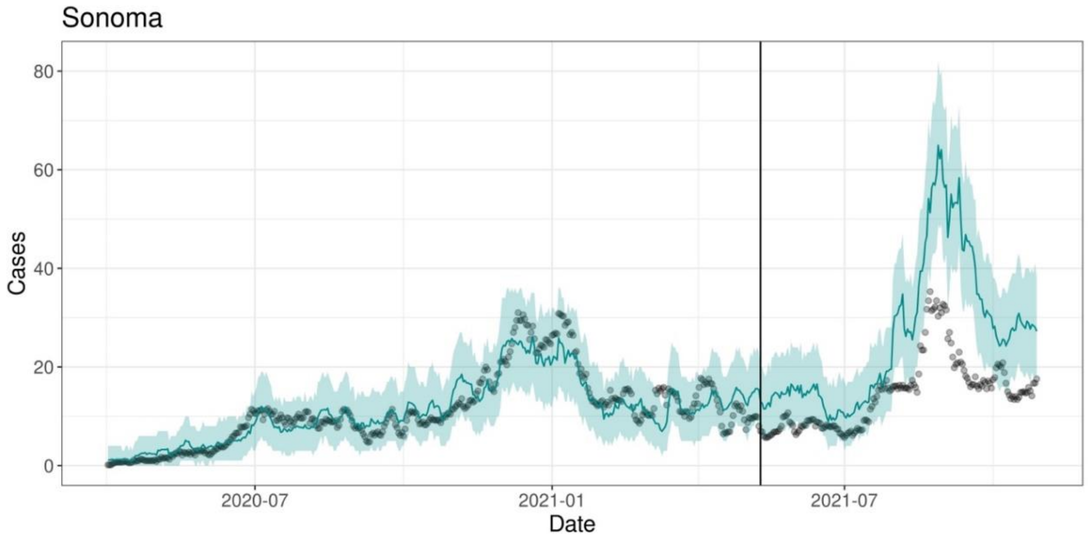

Model predictions for COVID-19 cases among children aged 12-15 years in Sonoma County.

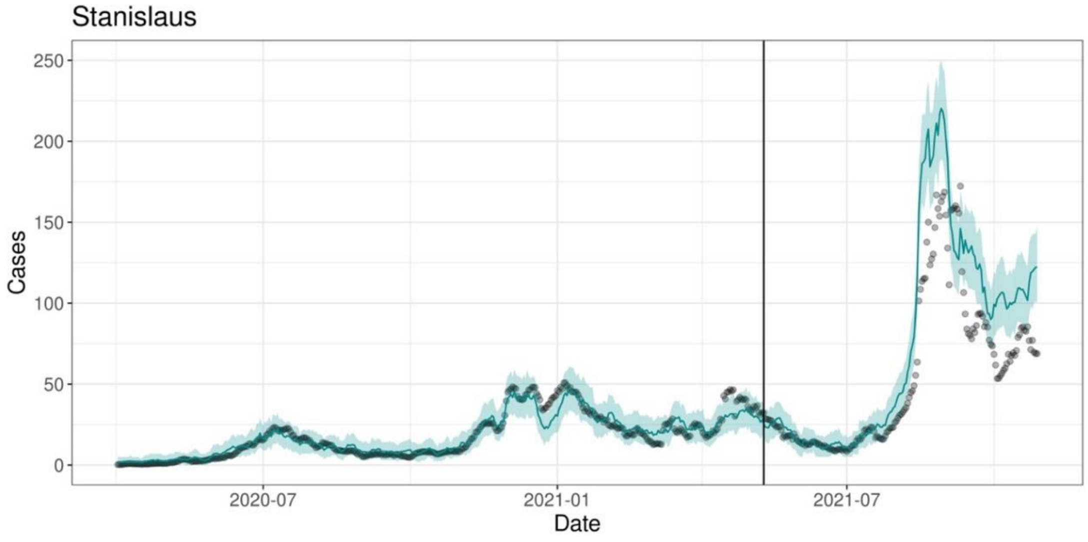

Model predictions for COVID-19 cases among children aged 12-15 years in Stanislaus County.

© 2024 Head JR et al. *JAMA Network Open.*

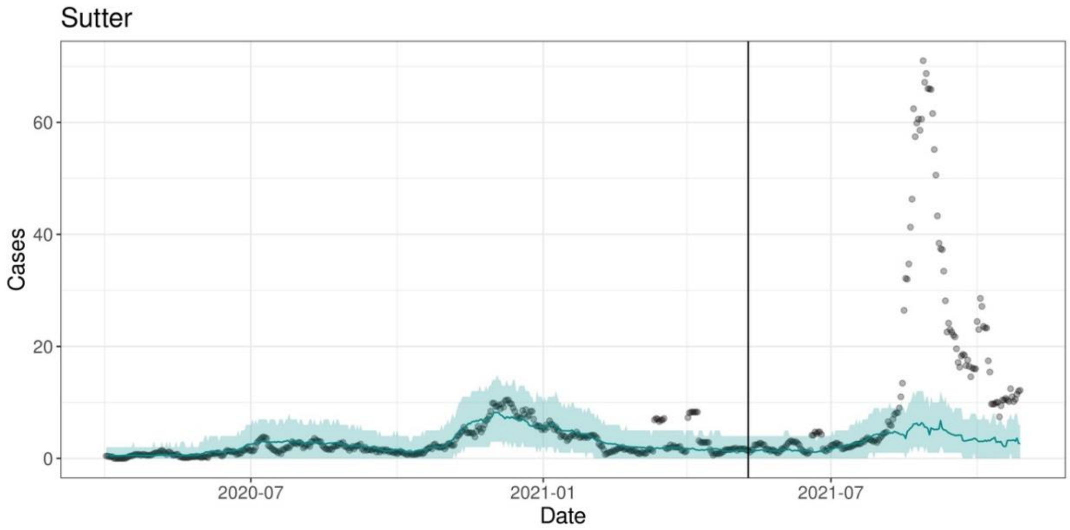

Model predictions for COVID-19 cases among children aged 12-15 years in Sutter County.

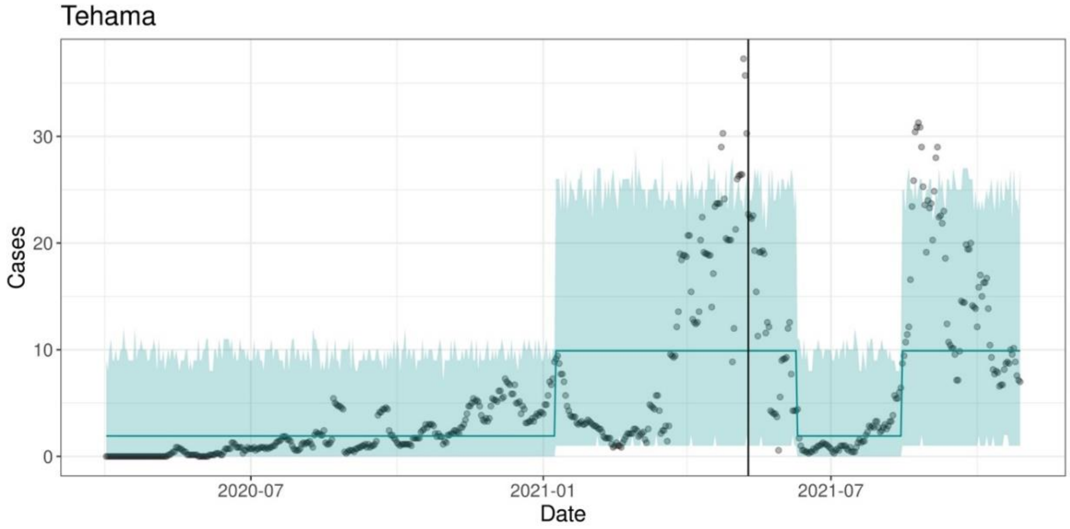

Model predictions for COVID-19 cases among children aged 12-15 years in Tehama County.

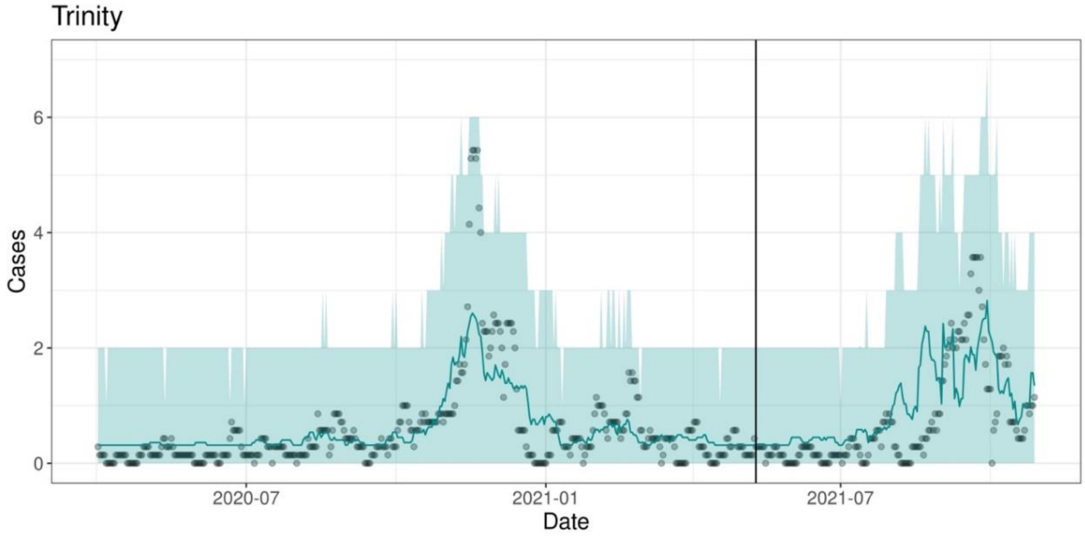

Model predictions for COVID-19 cases among children aged 12-15 years in Trinity County.

© 2024 Head JR et al. *JAMA Network Open.*

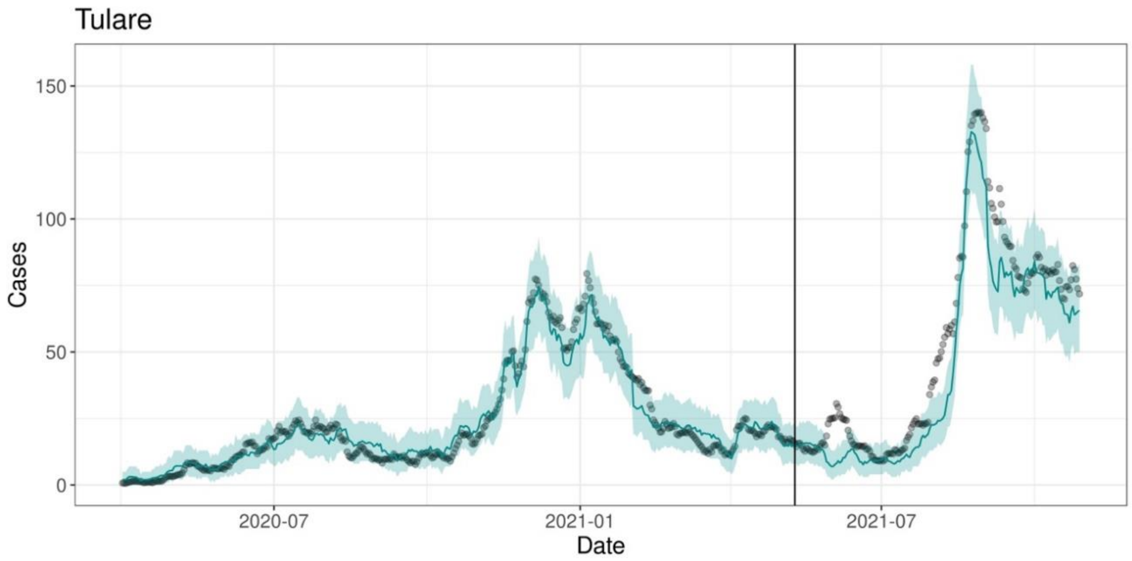

Model predictions for COVID-19 cases among children aged 12-15 years in Tulare County.

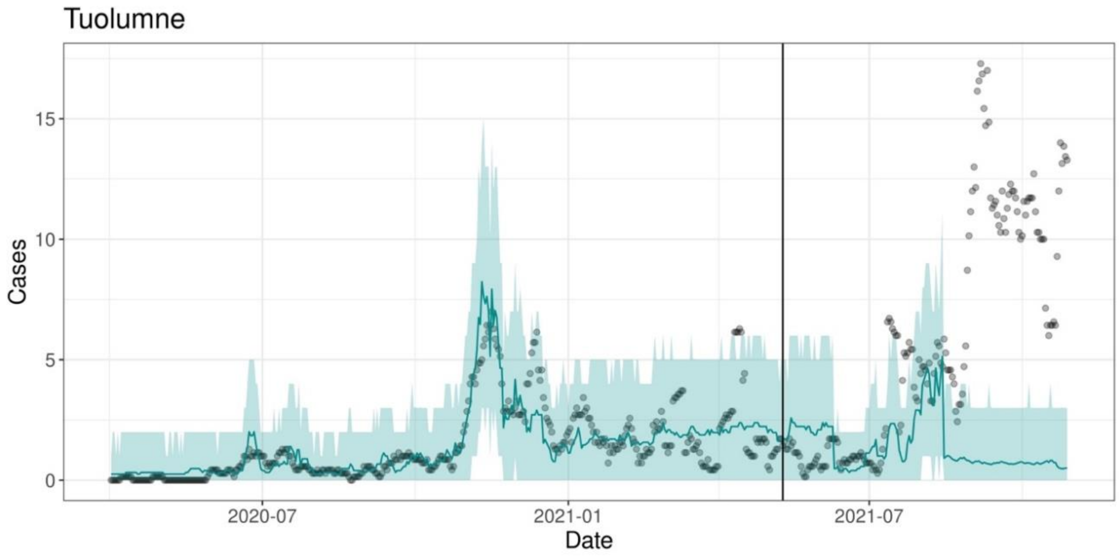

Model predictions for COVID-19 cases among children aged 12-15 years in Tuolumne County.

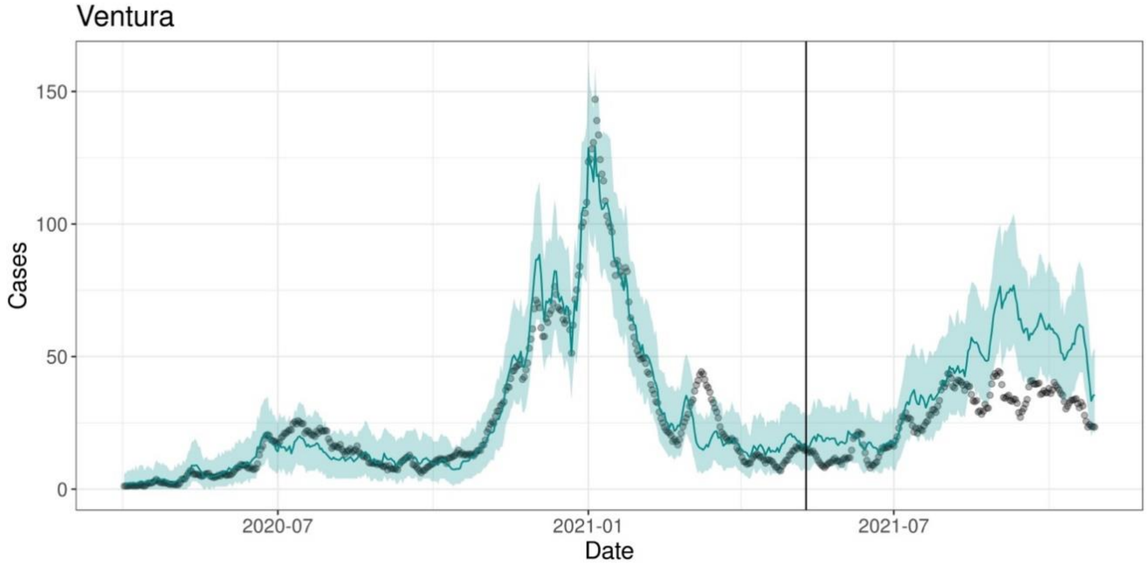

© 2024 Head JR et al. *JAMA Network Open.*

Model predictions for COVID-19 cases among children aged 12-15 years in Ventura County.

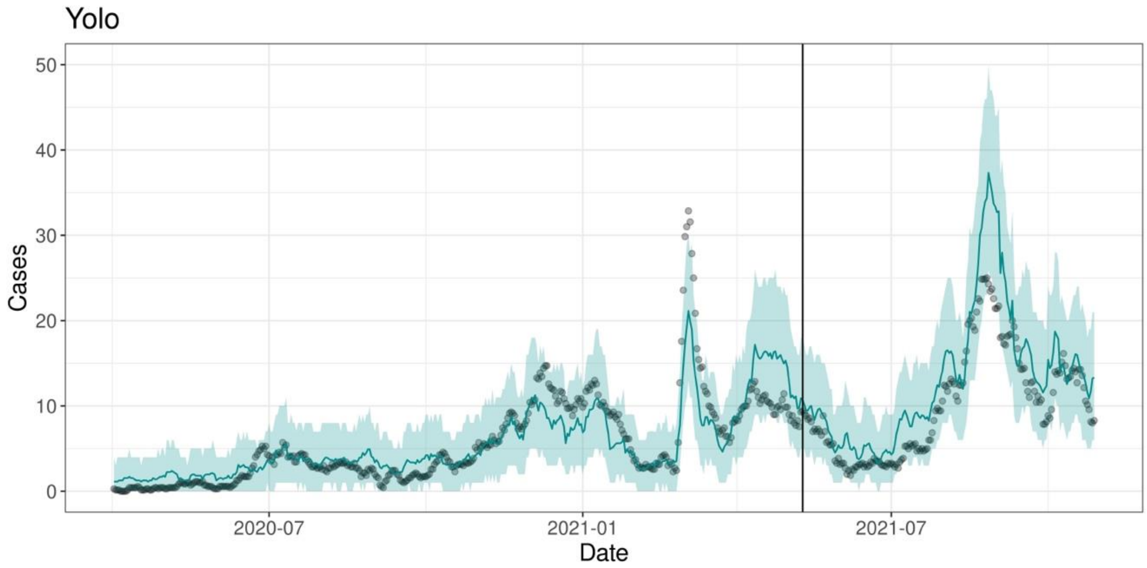

Model predictions for COVID-19 cases among children aged 12-15 years in Yolo County.

© 2024 Head JR et al. *JAMA Network Open.*

eFigure 10. Model predictions for COVID-19 cases among children aged 5-11 years. Black dots represent weekly hospitalization count, lines are model predictions, and shaded colored region is 95% prediction interval. Vertical lines are located at the time of vaccine eligibility.

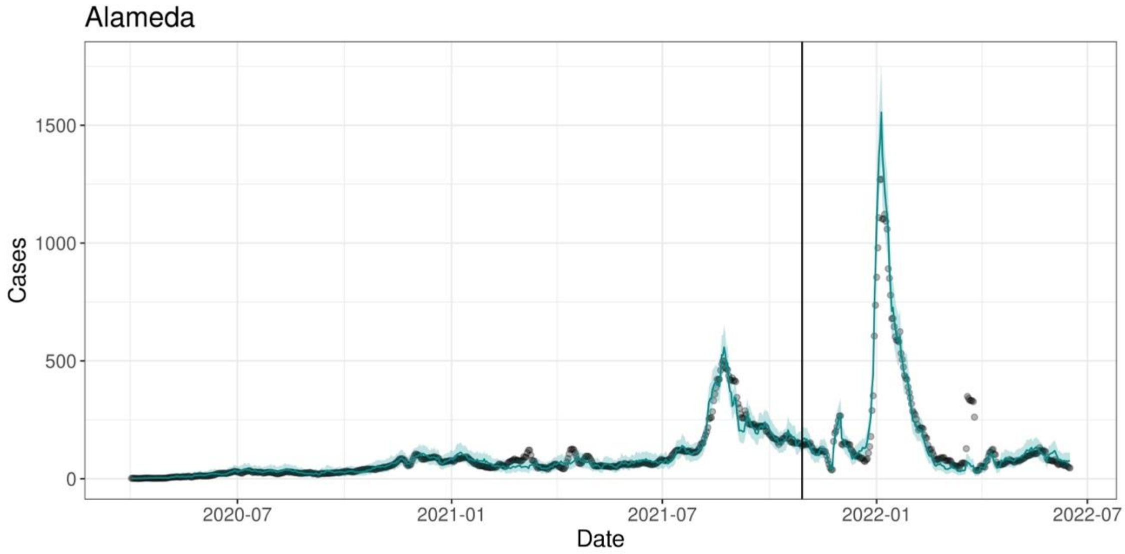

Model predictions for COVID-19 cases among children aged 5-11 years in Alameda County.

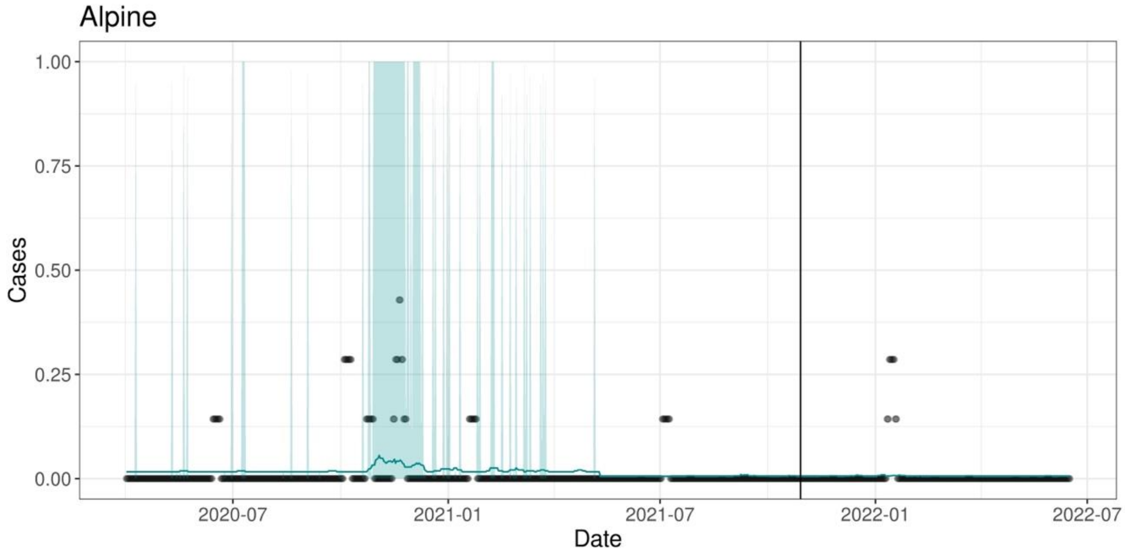

Model predictions for COVID-19 cases among children aged 5-11 years in Alpine County.

© 2024 Head JR et al. *JAMA Network Open.*

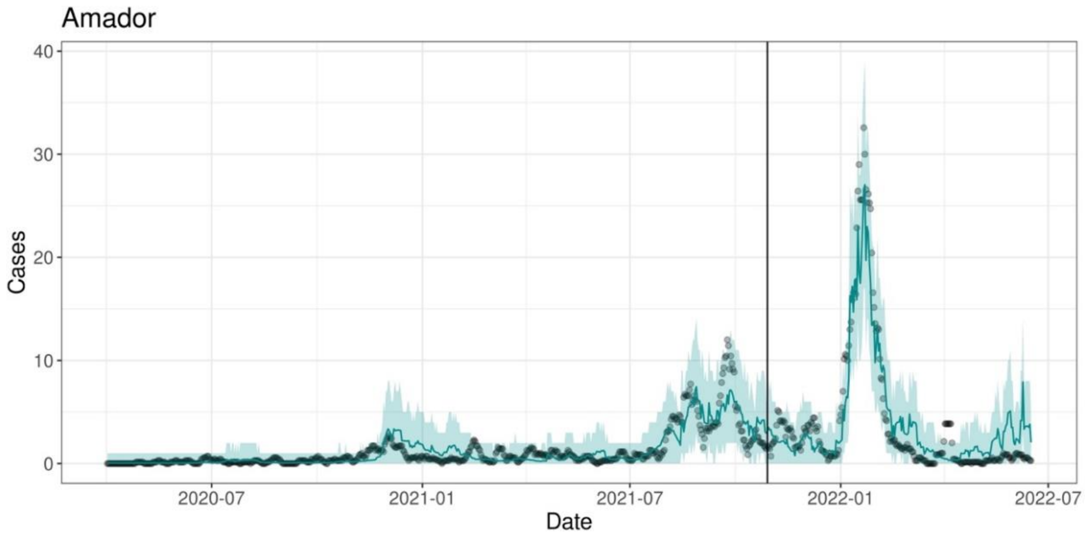

Model predictions for COVID-19 cases among children aged 5-11 years in Amador County.

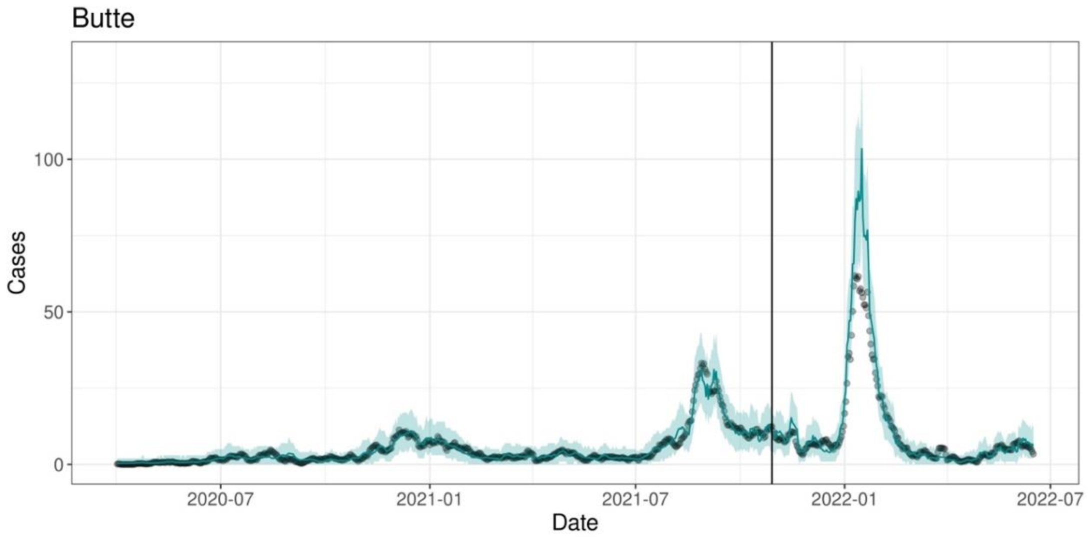

Model predictions for COVID-19 cases among children aged 5-11 years in Butte County.

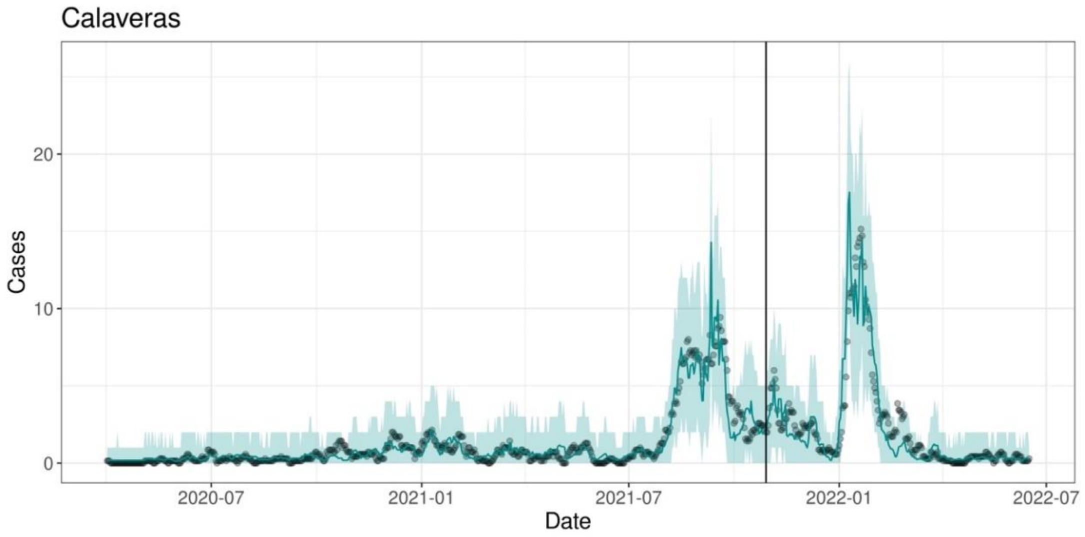

Model predictions for COVID-19 cases among children aged 5-11 years in Calaveras County.

© 2024 Head JR et al. *JAMA Network Open.*

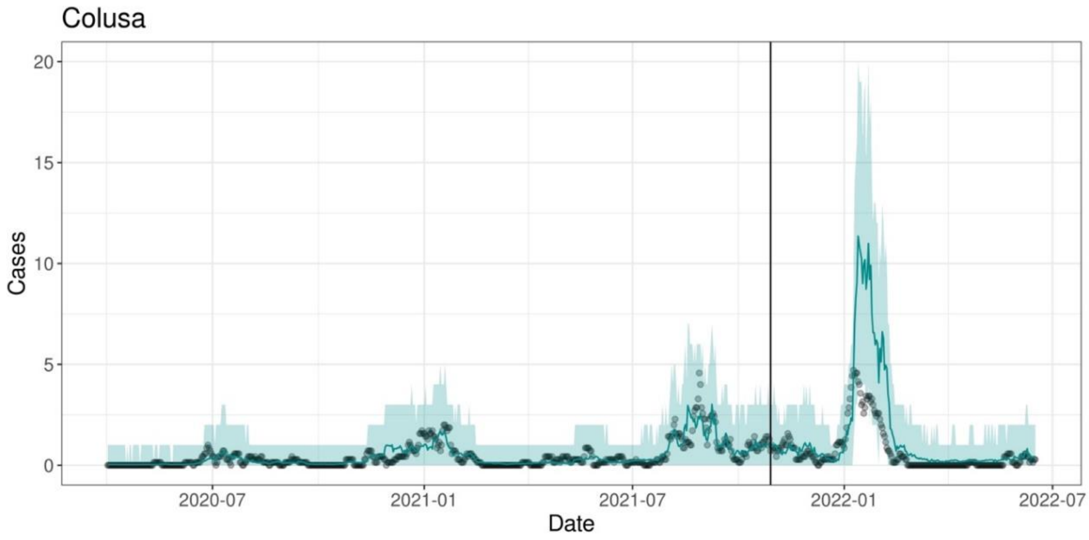

Model predictions for COVID-19 cases among children aged 5-11 years in Colusa County.

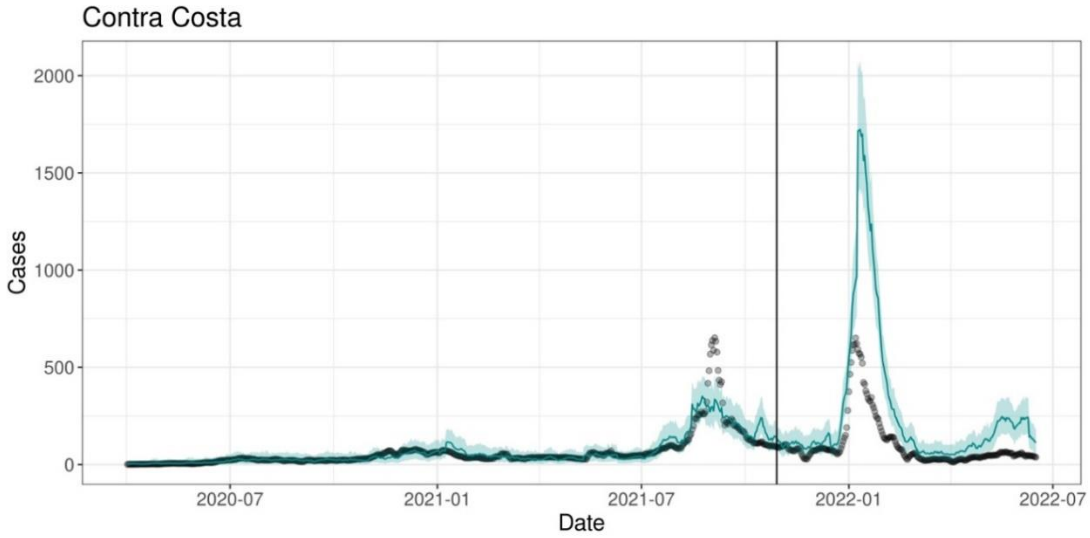

Model predictions for COVID-19 cases among children aged 5-11 years in Contra Costa County.

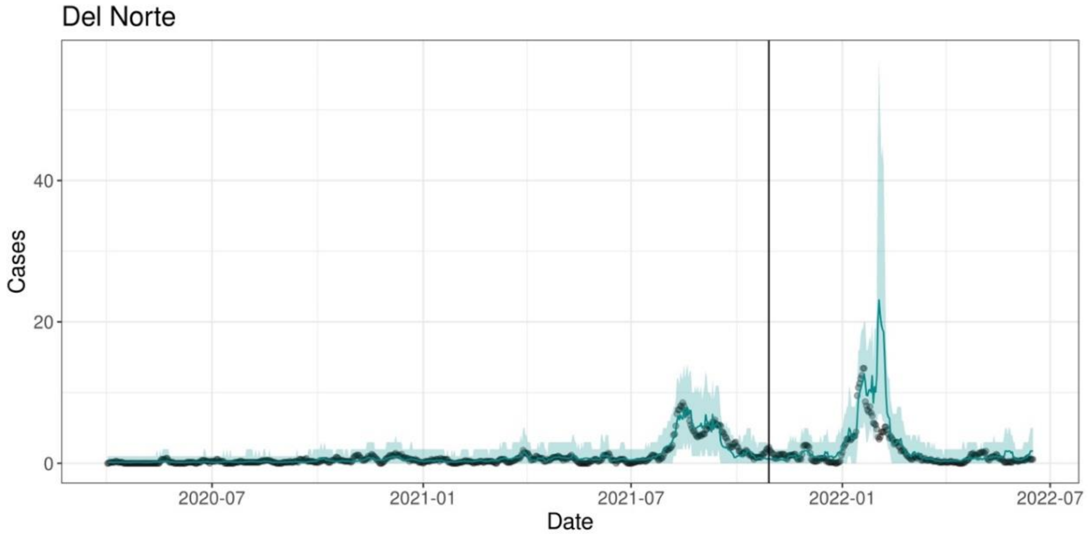

Model predictions for COVID-19 cases among children aged 5-11 years in Del Norte County.

© 2024 Head JR et al. *JAMA Network Open.*

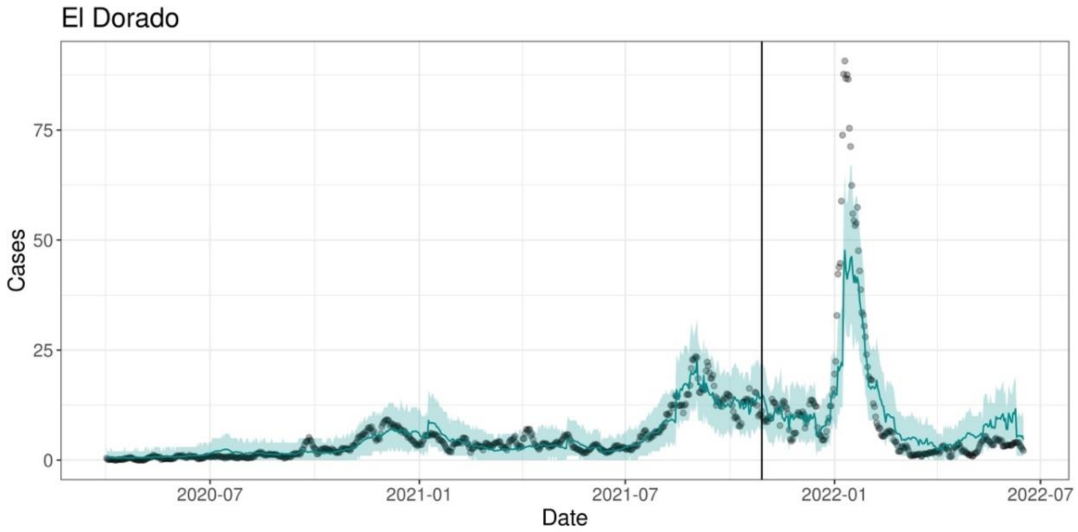

Model predictions for COVID-19 cases among children aged 5-11 years in El Dorado County.

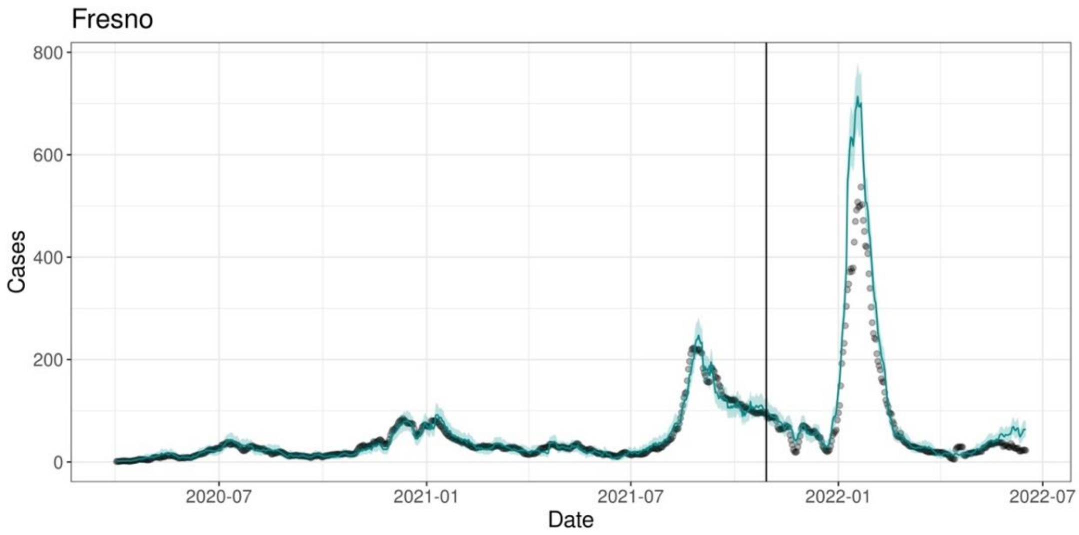

Model predictions for COVID-19 cases among children aged 5-11 years in Fresno County.

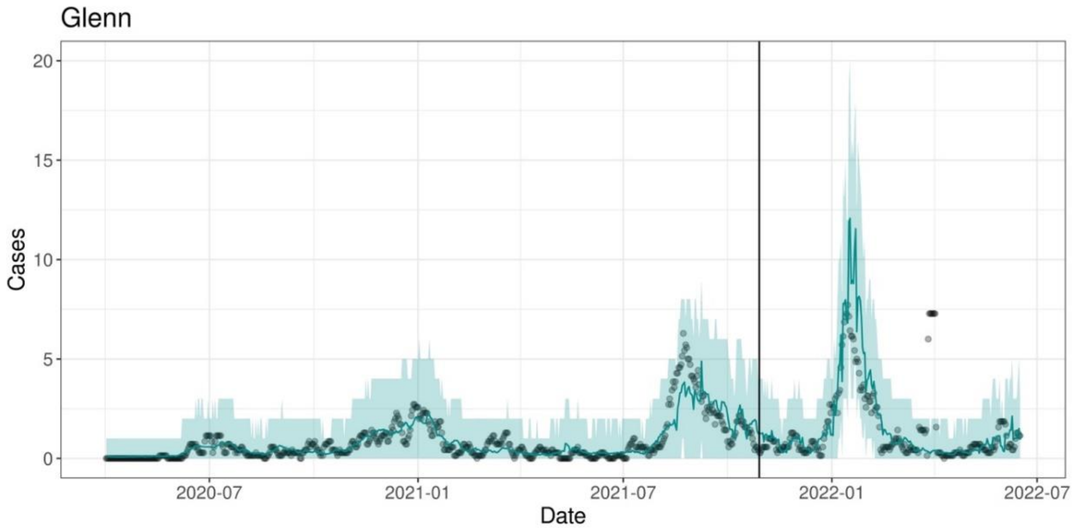

Model predictions for COVID-19 cases among children aged 5-11 years in Glenn County.

© 2024 Head JR et al. *JAMA Network Open.*

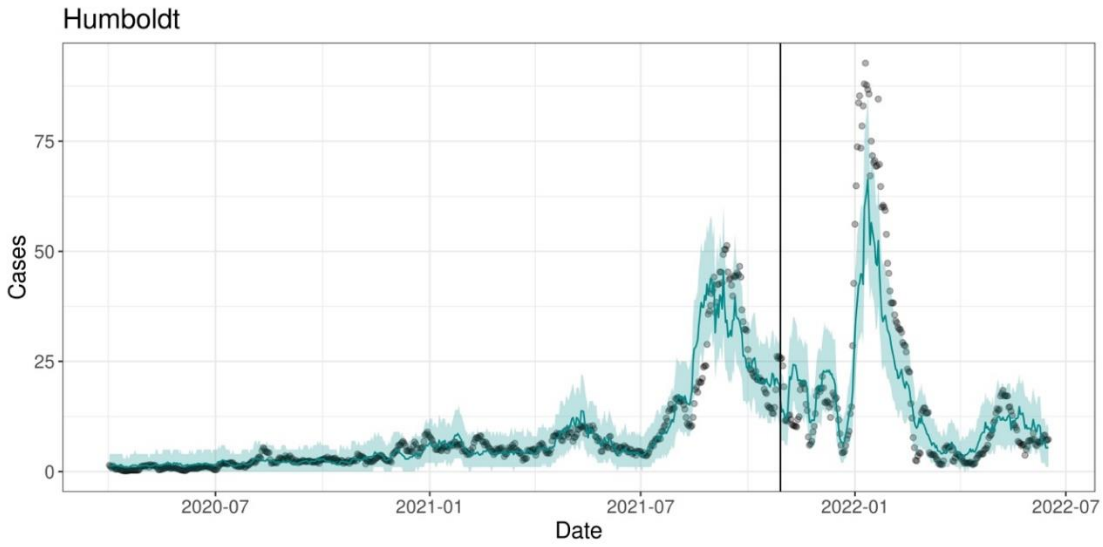

Model predictions for COVID-19 cases among children aged 5-11 years in Humboldt County.

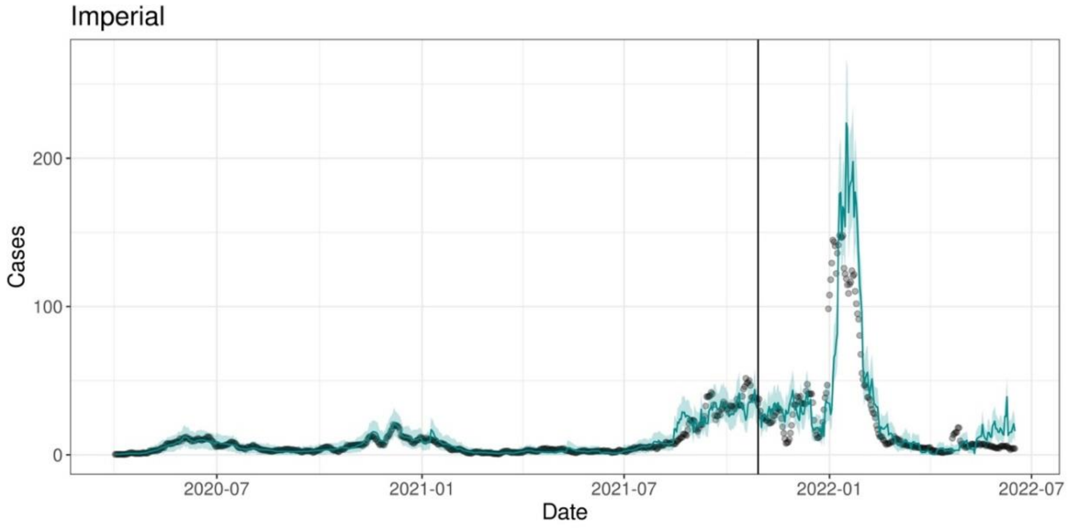

Model predictions for COVID-19 cases among children aged 5-11 years in Imperial County.

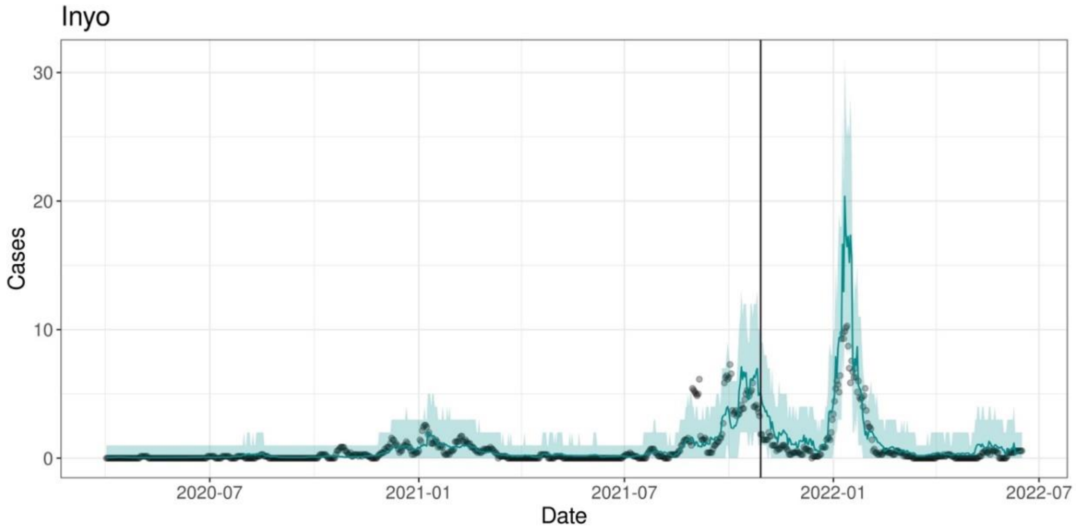

Model predictions for COVID-19 cases among children aged 5-11 years in Inyo County.

© 2024 Head JR et al. *JAMA Network Open.*

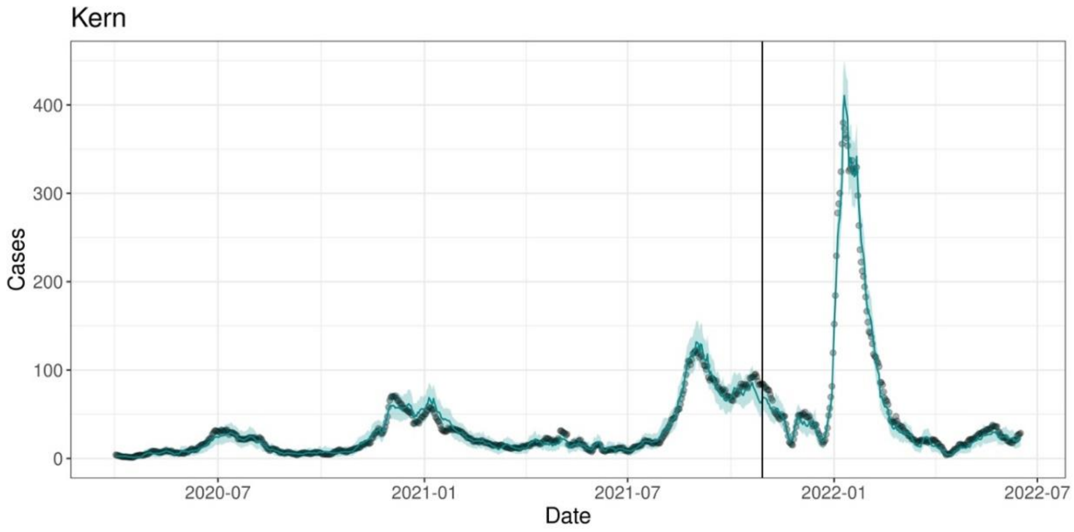

Model predictions for COVID-19 cases among children aged 5-11 years in Kern County.

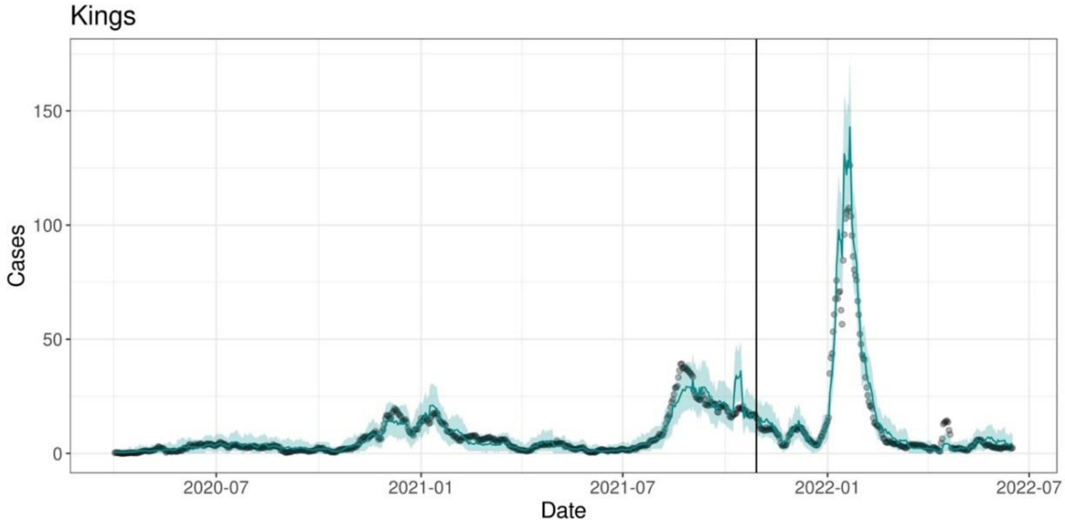

Model predictions for COVID-19 cases among children aged 5-11 years in Kings County.

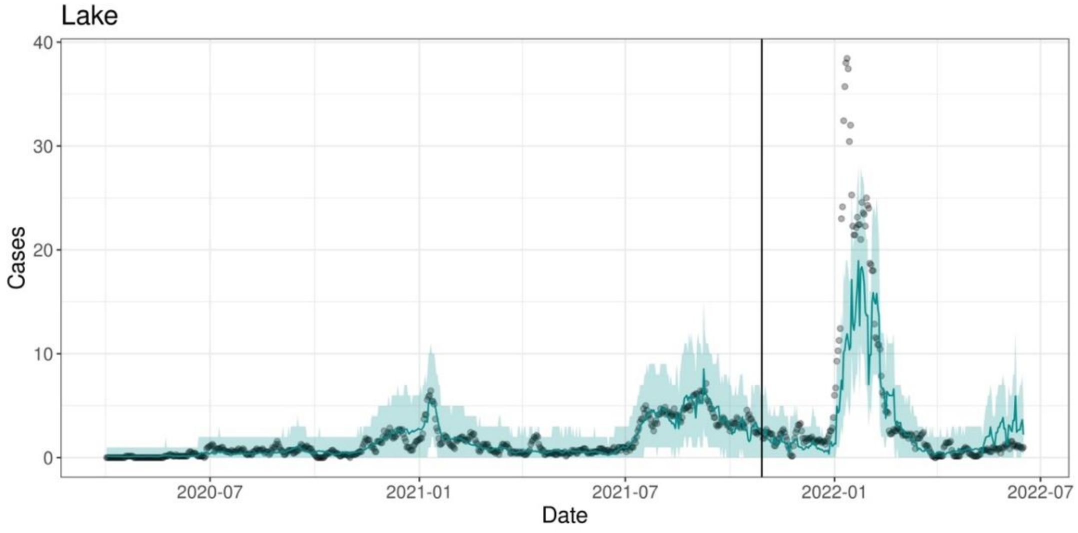

Model predictions for COVID-19 cases among children aged 5-11 years in Lake County.

© 2024 Head JR et al. *JAMA Network Open.*

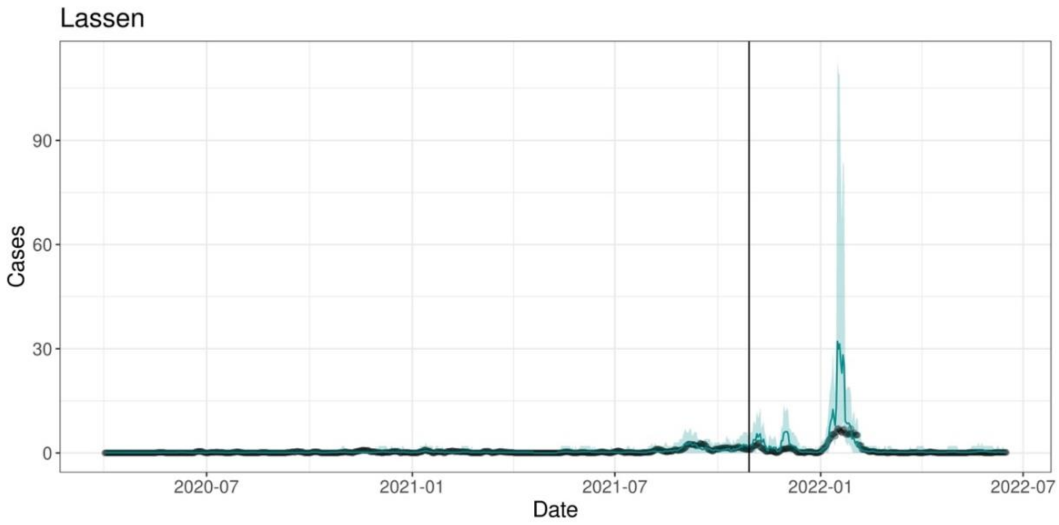

Model predictions for COVID-19 cases among children aged 5-11 years in Lassen County.

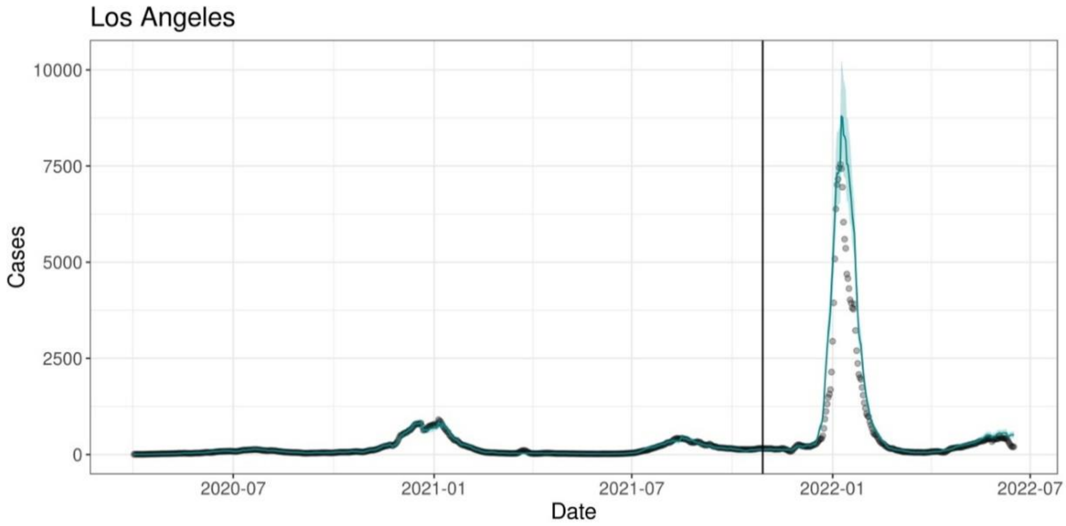

Model predictions for COVID-19 cases among children aged 5-11 years in Los Angeles County.

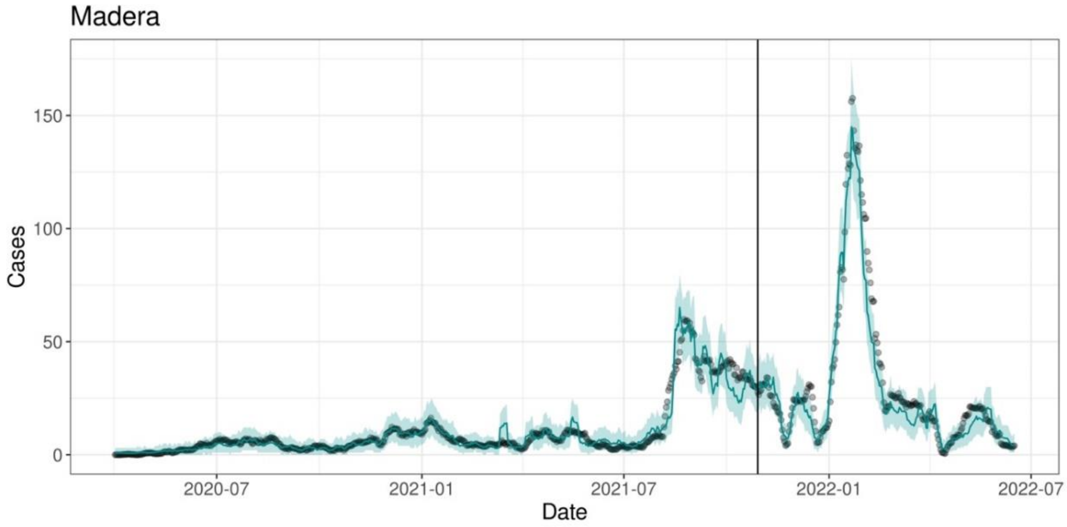

Model predictions for COVID-19 cases among children aged 5-11 years in Madera County.

© 2024 Head JR et al. *JAMA Network Open.*

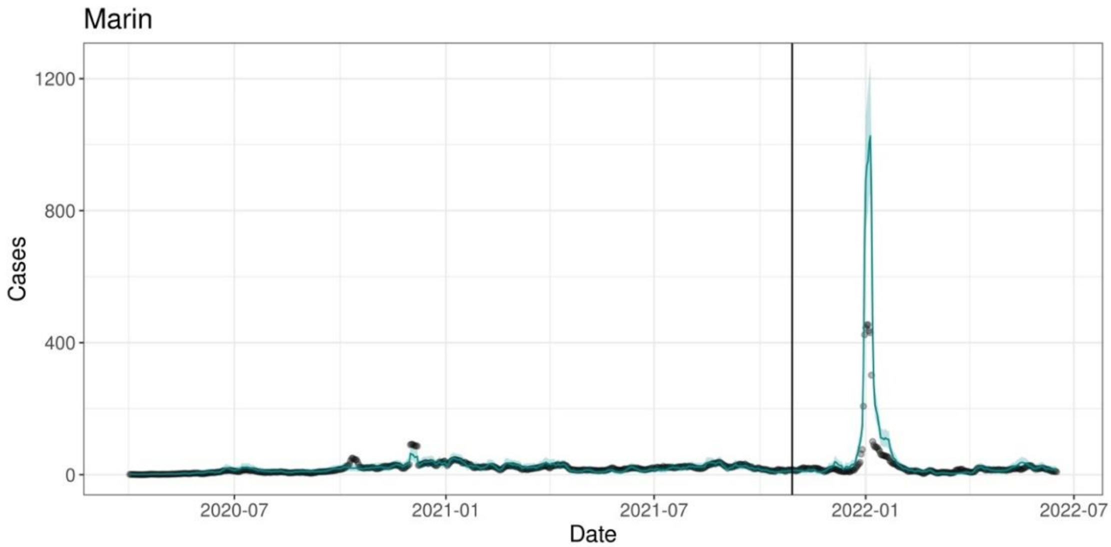

Model predictions for COVID-19 cases among children aged 5-11 years in Marin County.

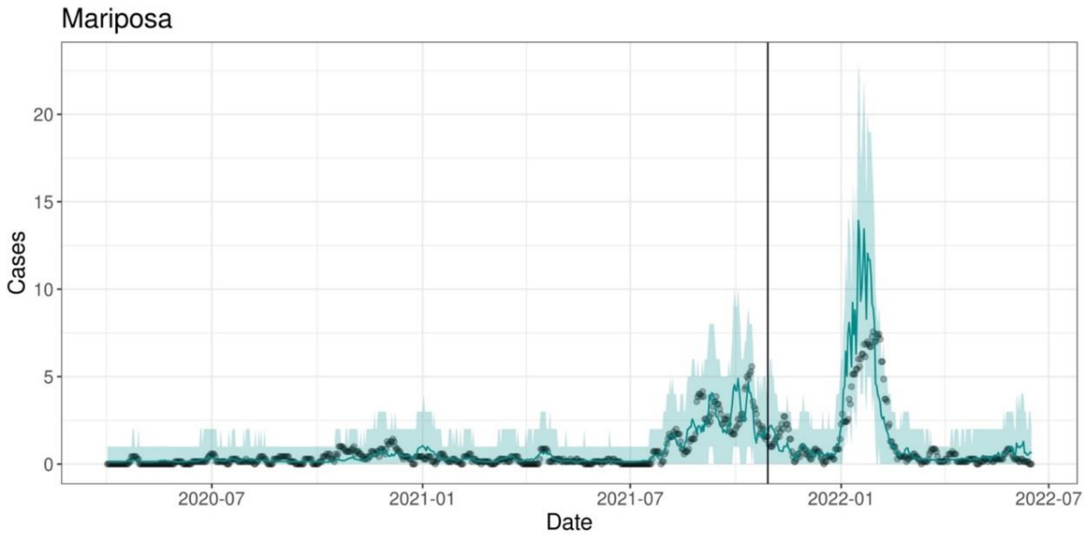

Model predictions for COVID-19 cases among children aged 5-11 years in Mariposa County.

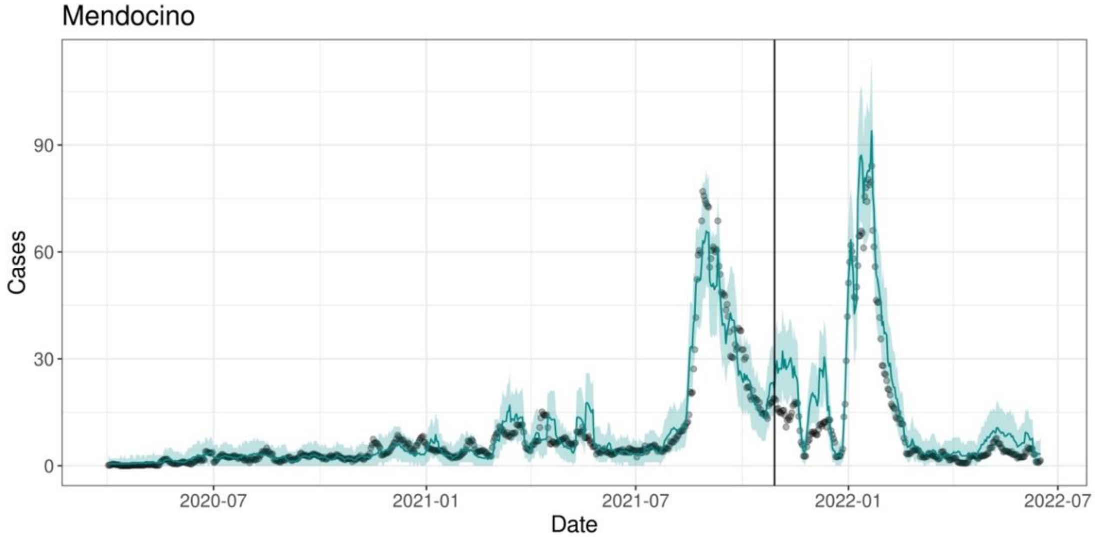

Model predictions for COVID-19 cases among children aged 5-11 years in Mendocino County.

© 2024 Head JR et al. *JAMA Network Open.*

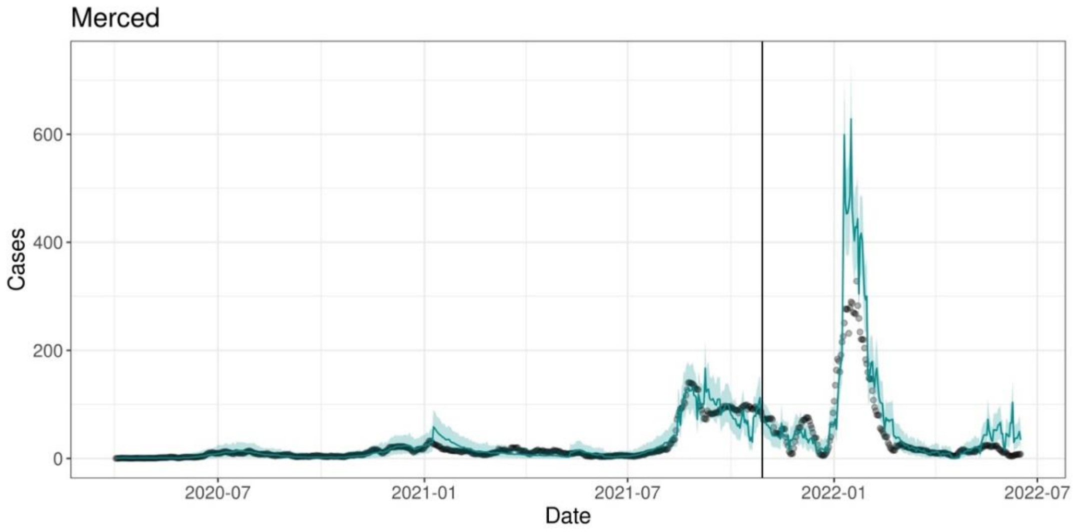

Model predictions for COVID-19 cases among children aged 5-11 years in Merced County.

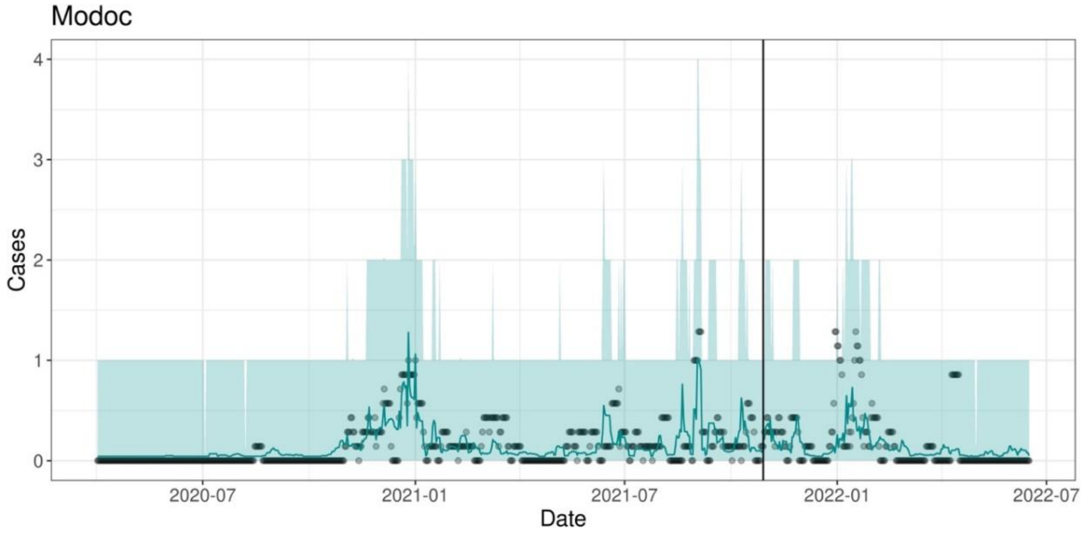

Model predictions for COVID-19 cases among children aged 5-11 years in Modoc County.

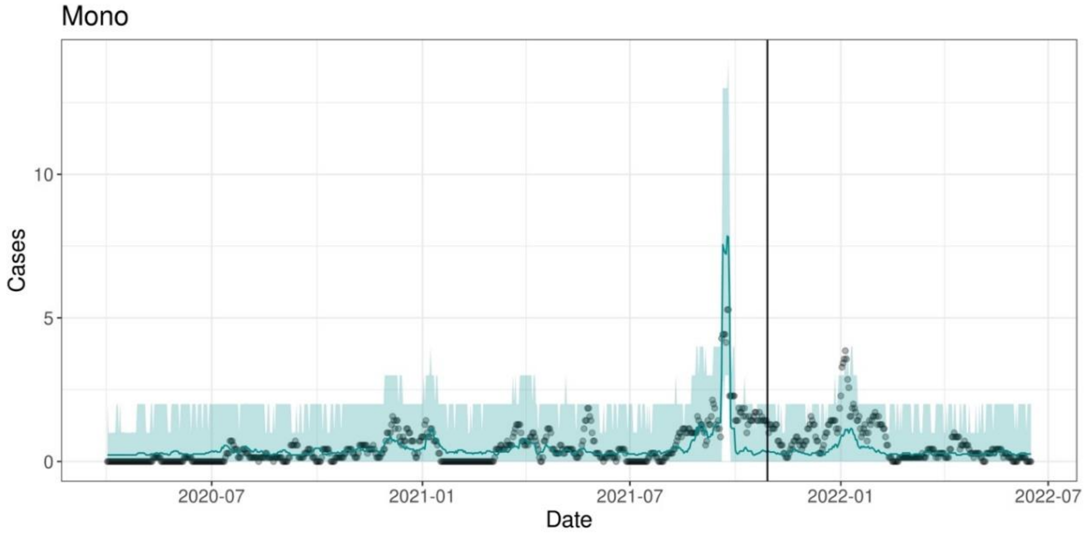

Model predictions for COVID-19 cases among children aged 5-11 years in Mono County.

© 2024 Head JR et al. *JAMA Network Open.*

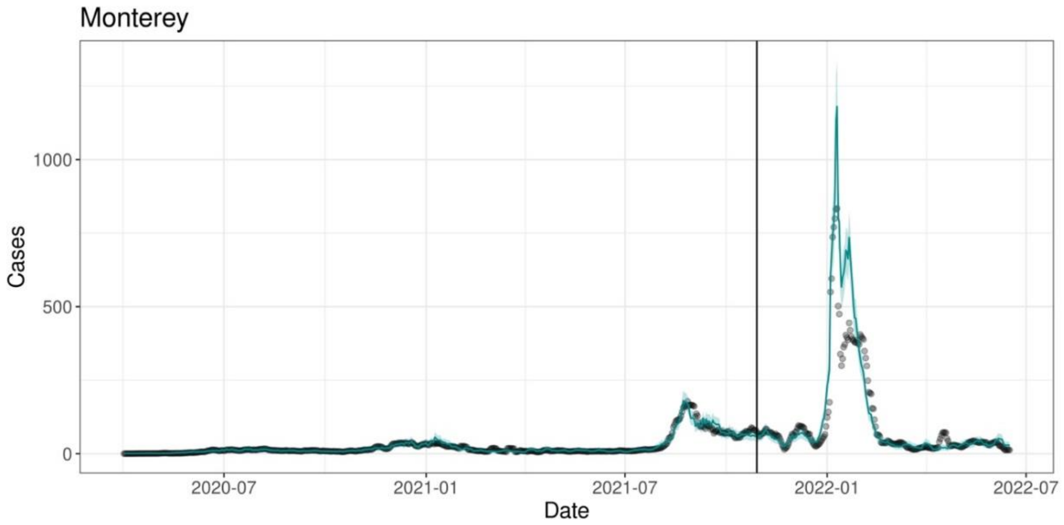

Model predictions for COVID-19 cases among children aged 5-11 years in Monterey County.

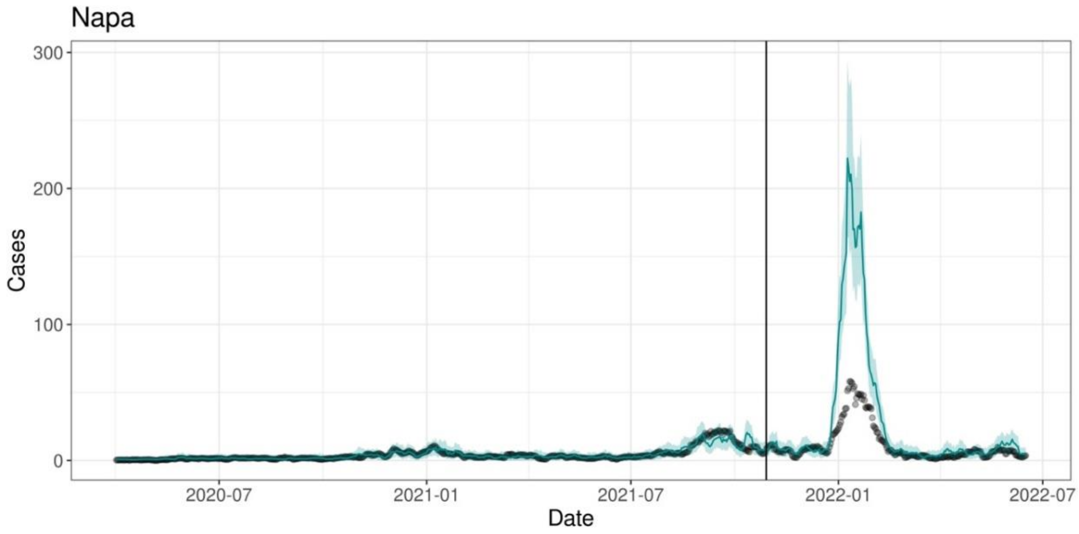

Model predictions for COVID-19 cases among children aged 5-11 years in Napa County.

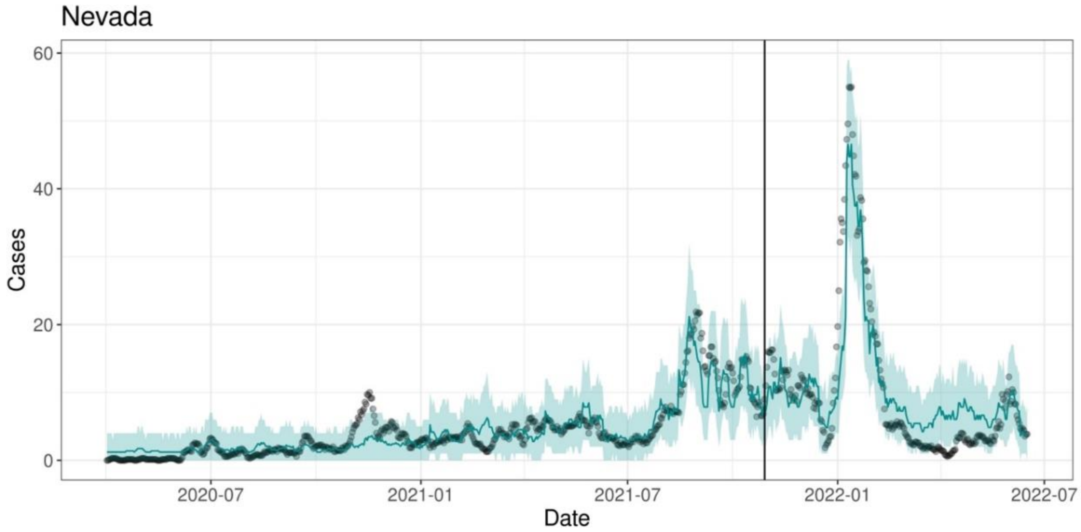

Model predictions for COVID-19 cases among children aged 5-11 years in Nevada County.

© 2024 Head JR et al. *JAMA Network Open.*

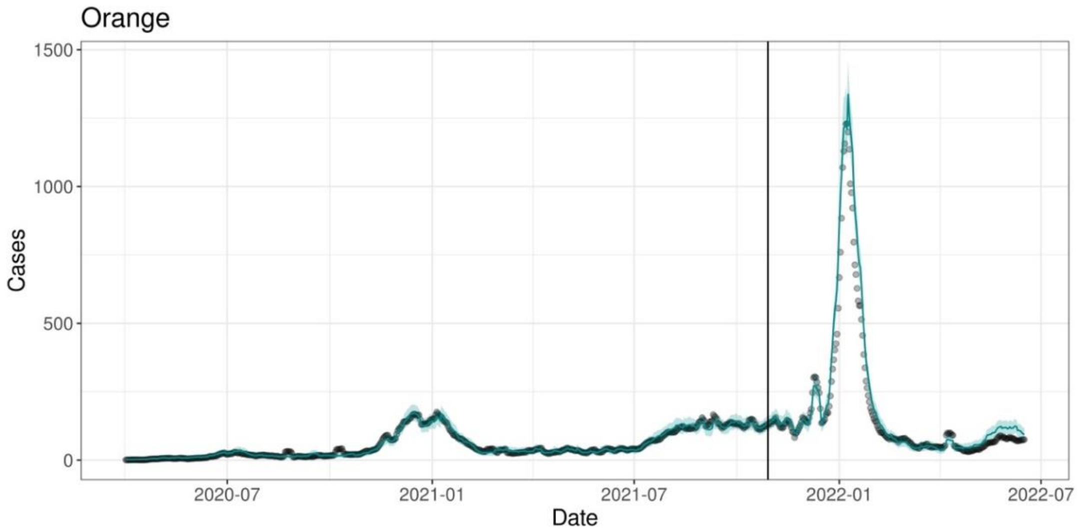

Model predictions for COVID-19 cases among children aged 5-11 years in Orange County.

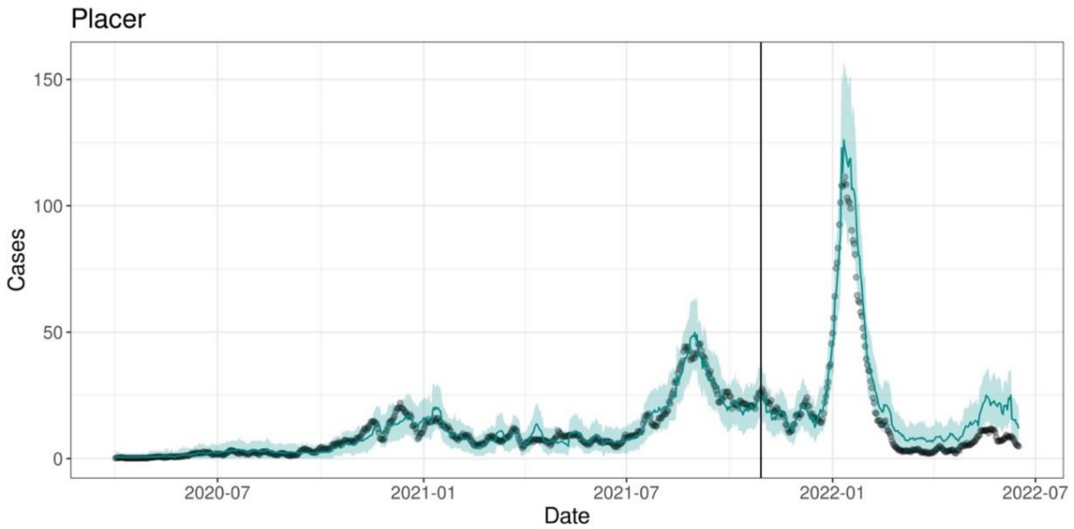

Model predictions for COVID-19 cases among children aged 5-11 years in Placer County.

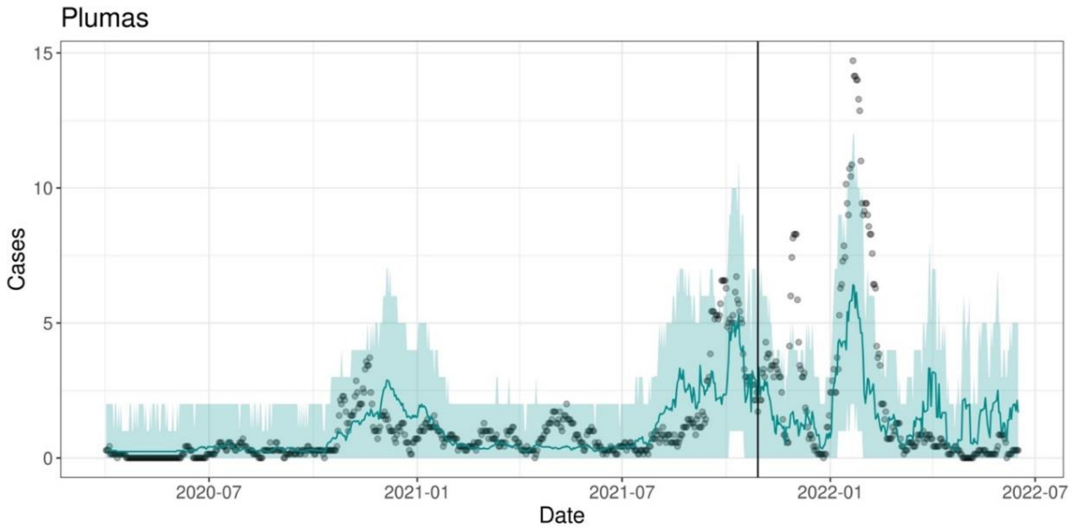

Model predictions for COVID-19 cases among children aged 5-11 years in Plumas County.

© 2024 Head JR et al. *JAMA Network Open.*

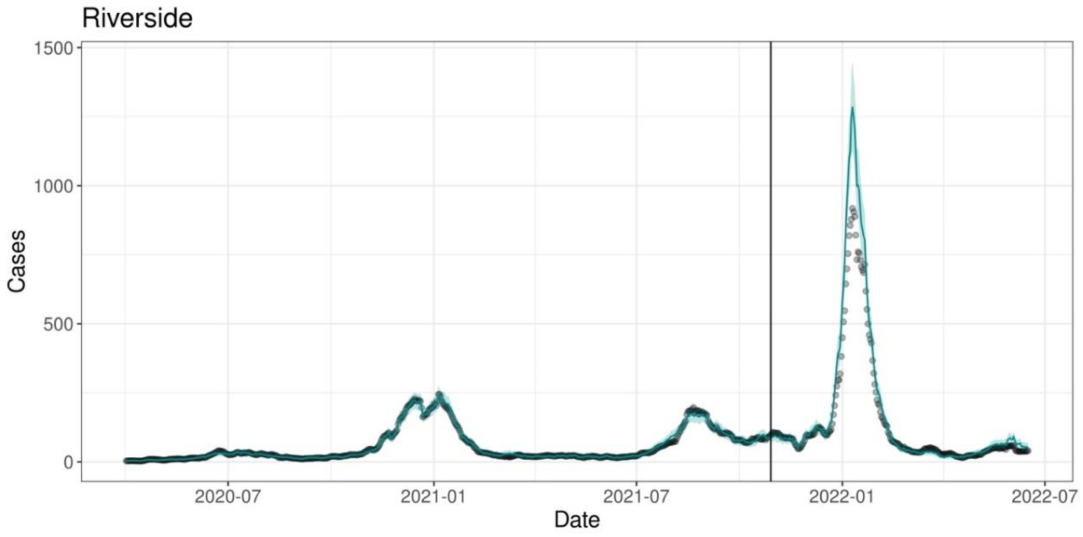

Model predictions for COVID-19 cases among children aged 5-11 years in Riverside County.

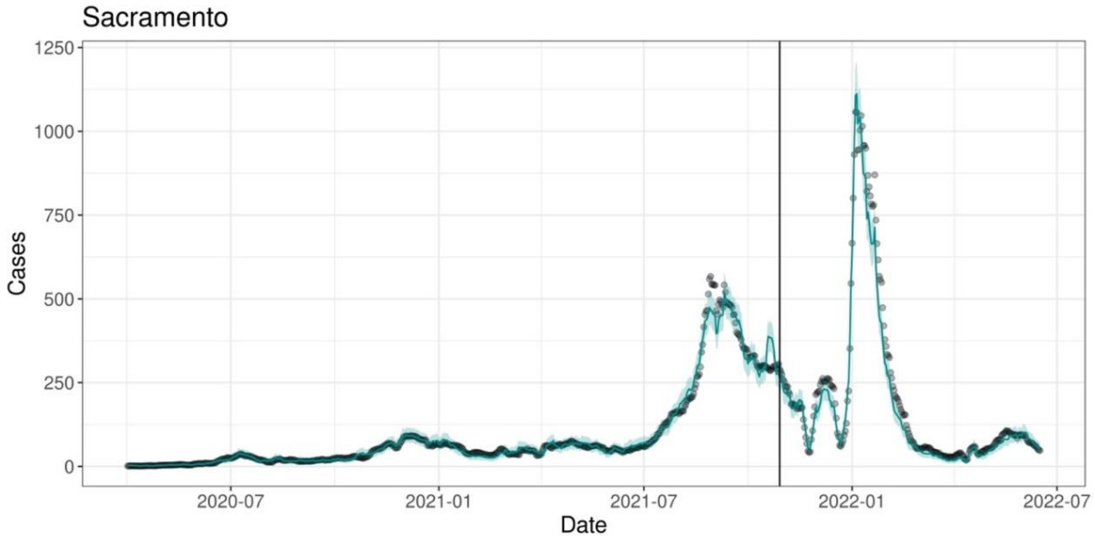

Model predictions for COVID-19 cases among children aged 5-11 years in Sacramento County.

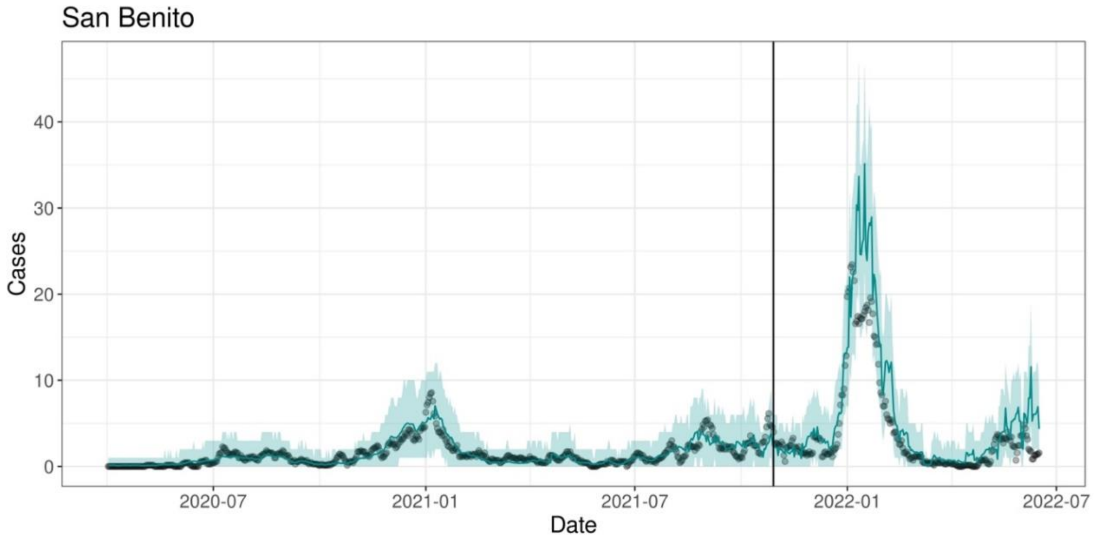

Model predictions for COVID-19 cases among children aged 5-11 years in San Benito County.

© 2024 Head JR et al. *JAMA Network Open.*

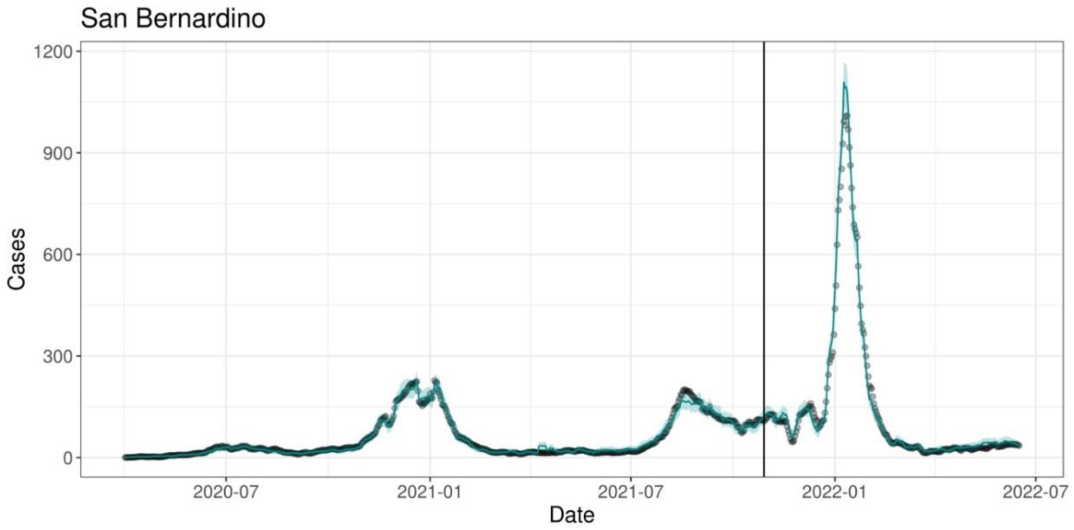

Model predictions for COVID-19 cases among children aged 5-11 years in San Bernardino County.

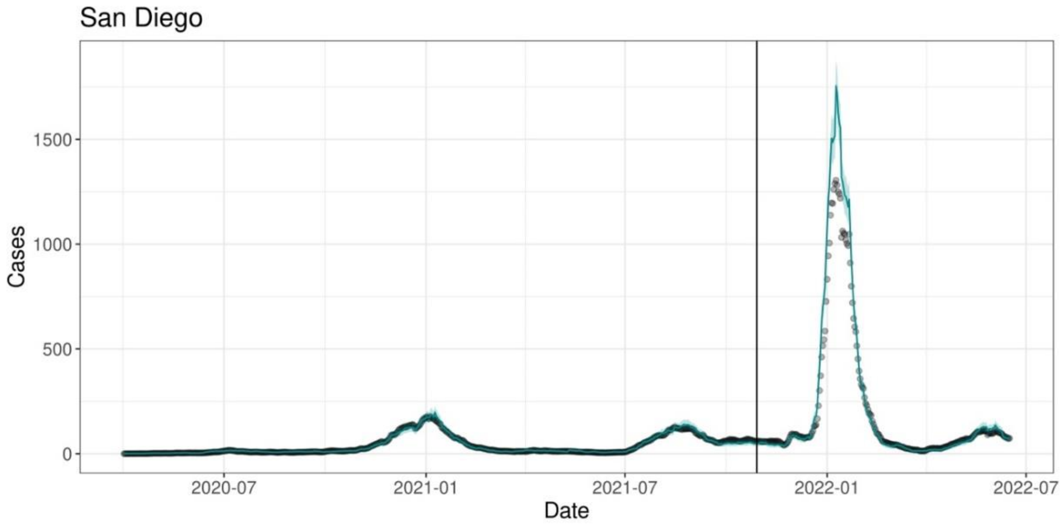

Model predictions for COVID-19 cases among children aged 5-11 years in San Diego County.

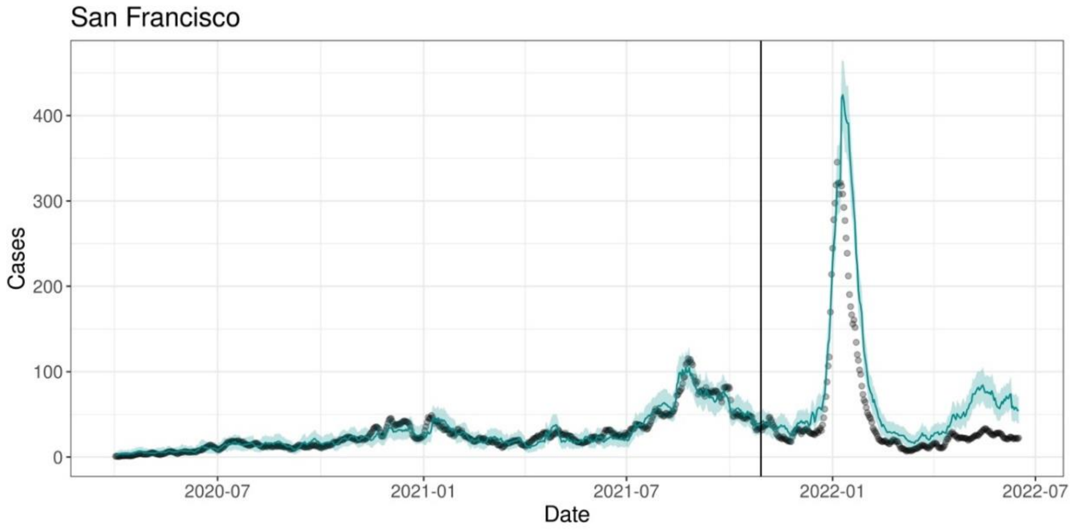

Model predictions for COVID-19 cases among children aged 5-11 years in San Francisco County.

© 2024 Head JR et al. *JAMA Network Open.*

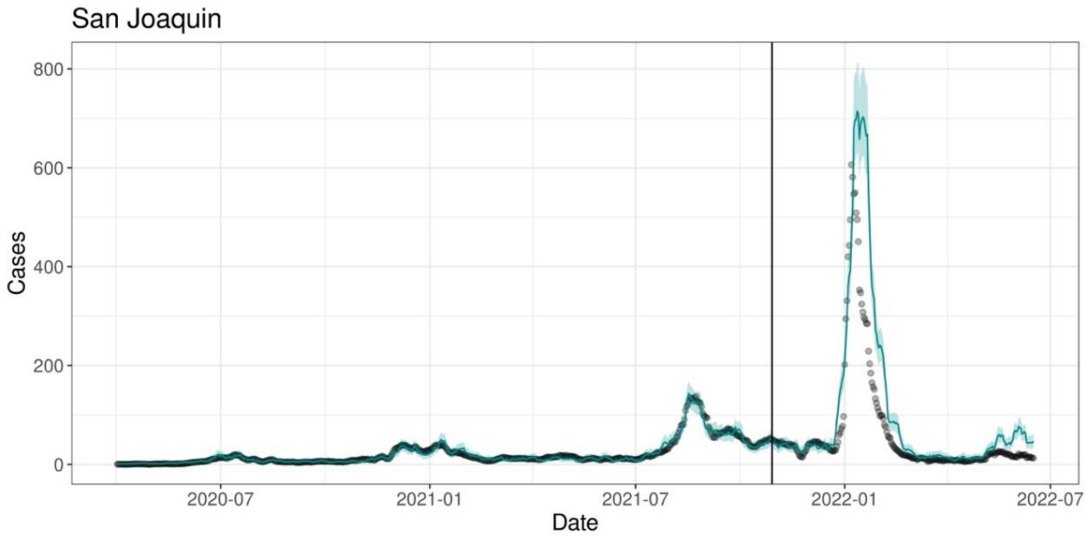

Model predictions for COVID-19 cases among children aged 5-11 years in San Joaquin County.

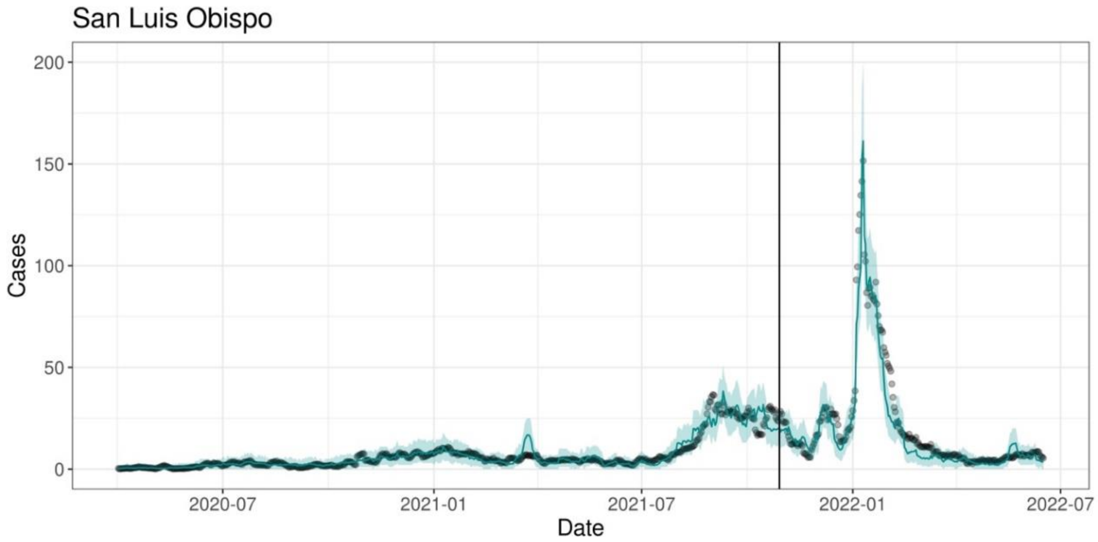

Model predictions for COVID-19 cases among children aged 5-11 years in San Luis Obispo County.

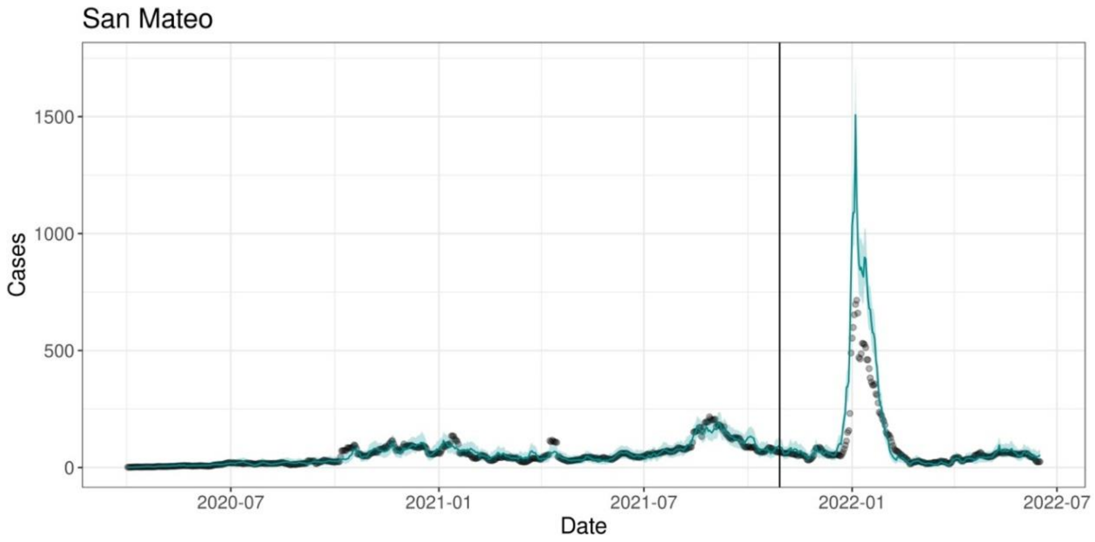

Model predictions for COVID-19 cases among children aged 5-11 years in San Mateo County.

© 2024 Head JR et al. *JAMA Network Open.*

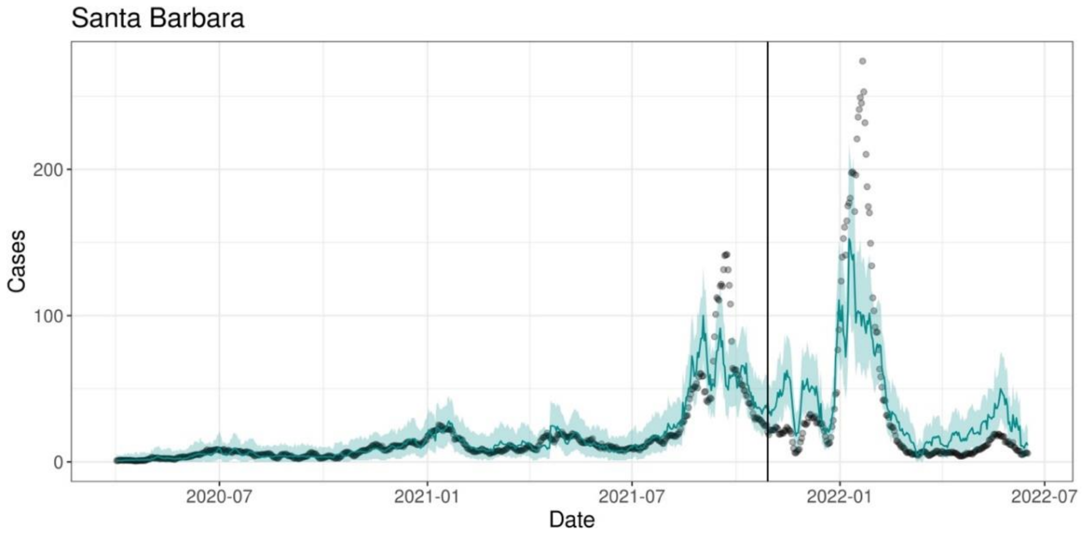

Model predictions for COVID-19 cases among children aged 5-11 years in Santa Barbara County.

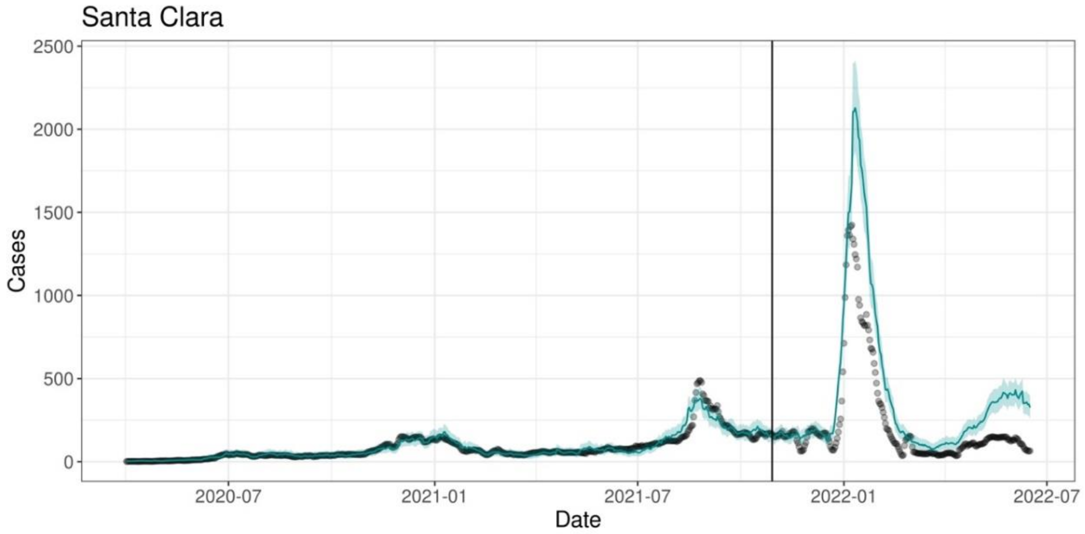

Model predictions for COVID-19 cases among children aged 5-11 years in Santa Clara County.

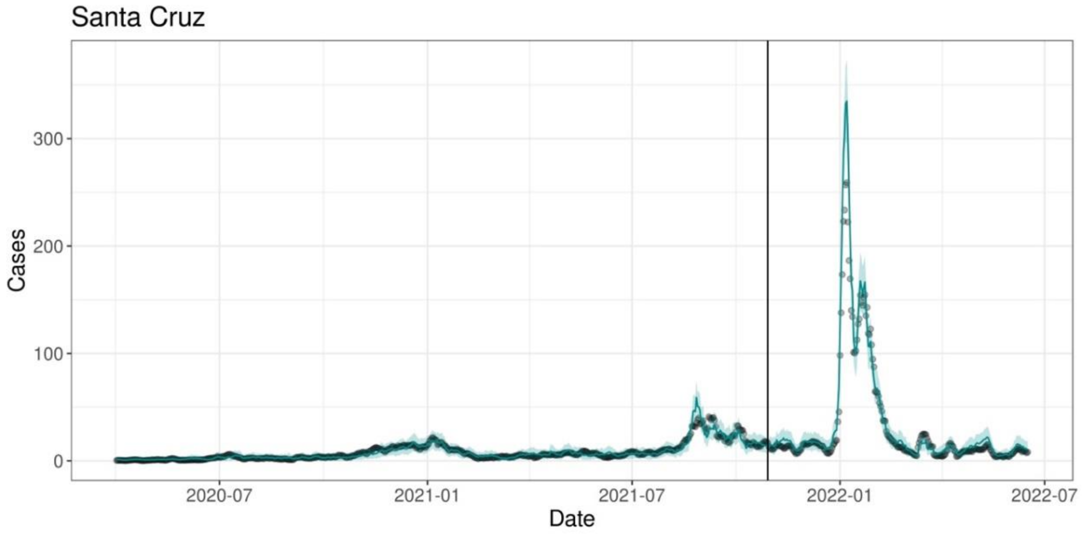

Model predictions for COVID-19 cases among children aged 5-11 years in Santa Cruz County.

© 2024 Head JR et al. *JAMA Network Open.*

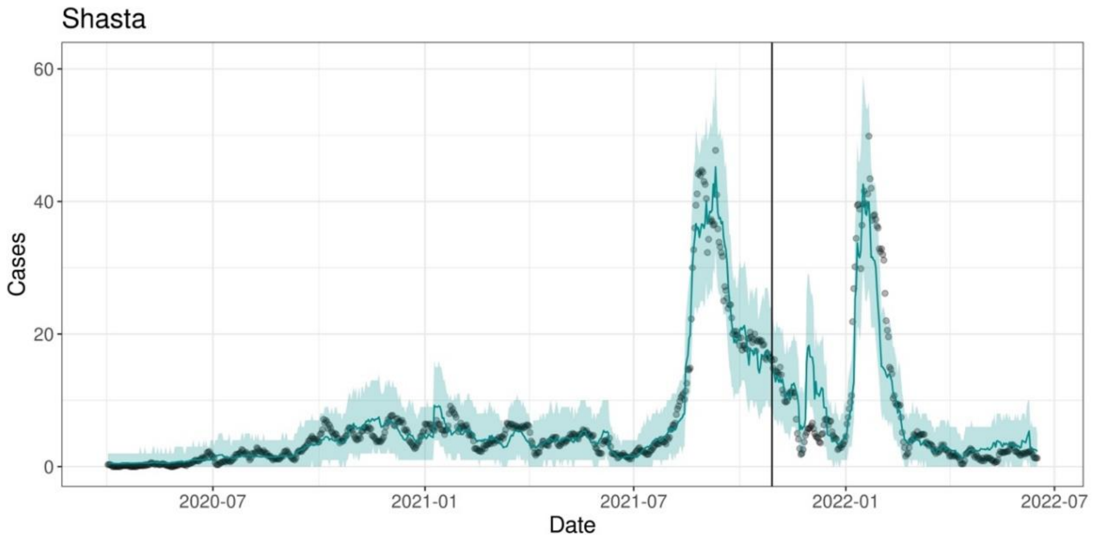

Model predictions for COVID-19 cases among children aged 5-11 years in Shasta County.

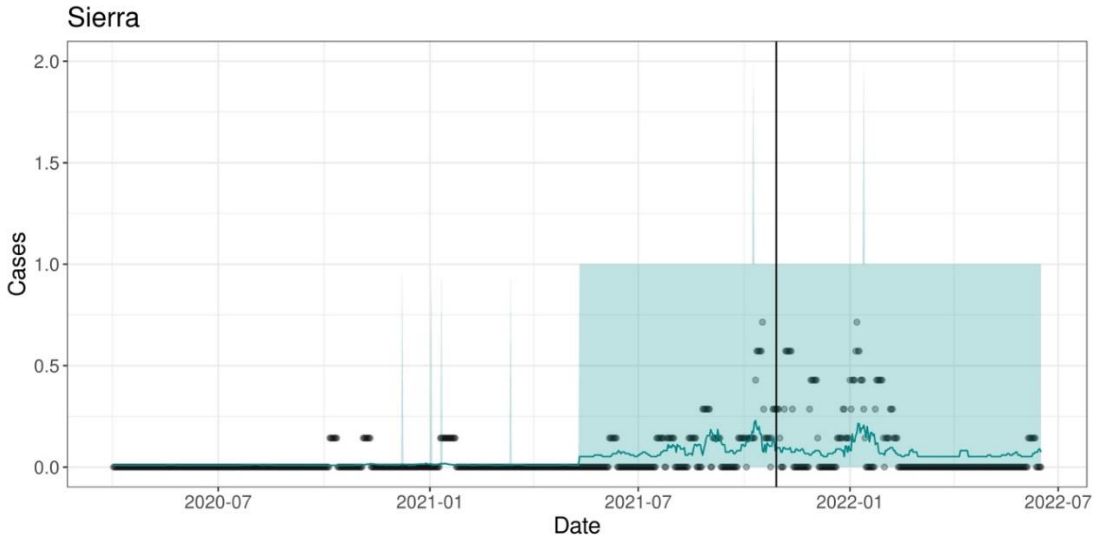

Model predictions for COVID-19 cases among children aged 5-11 years in Sierra County.

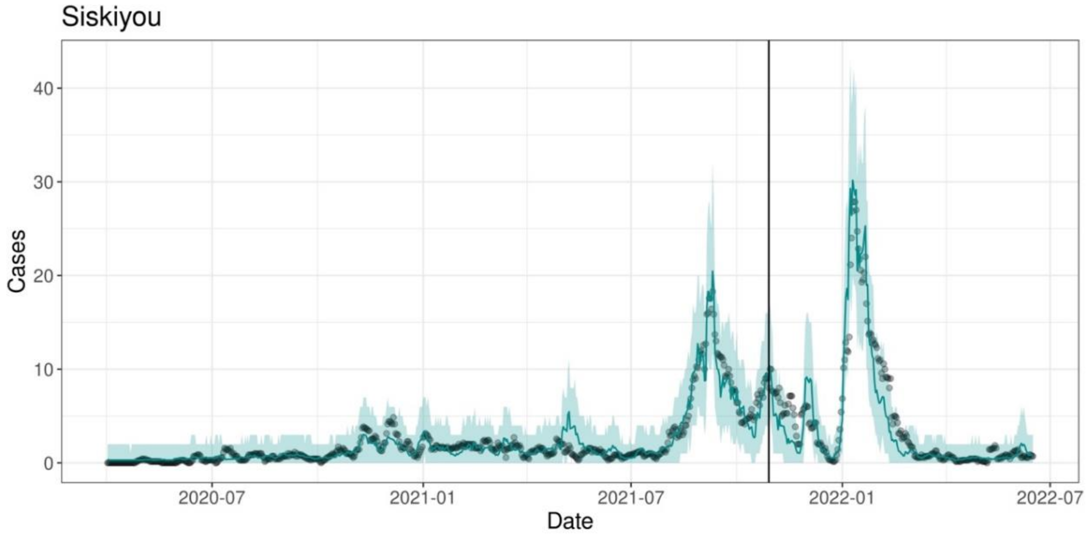

Model predictions for COVID-19 cases among children aged 5-11 years in Siskiyou County.

© 2024 Head JR et al. *JAMA Network Open.*

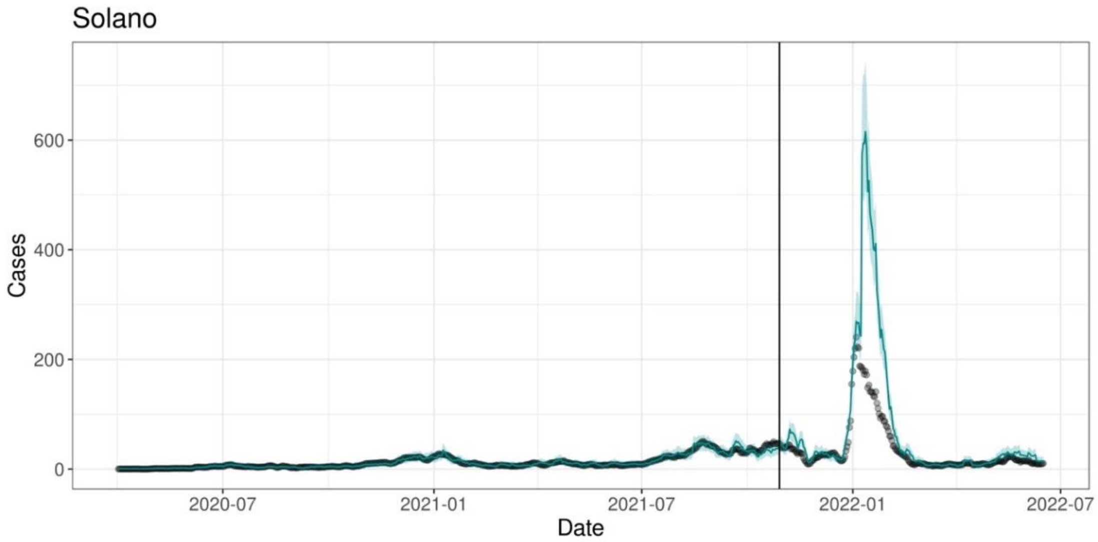

Model predictions for COVID-19 cases among children aged 5-11 years in Solano County.

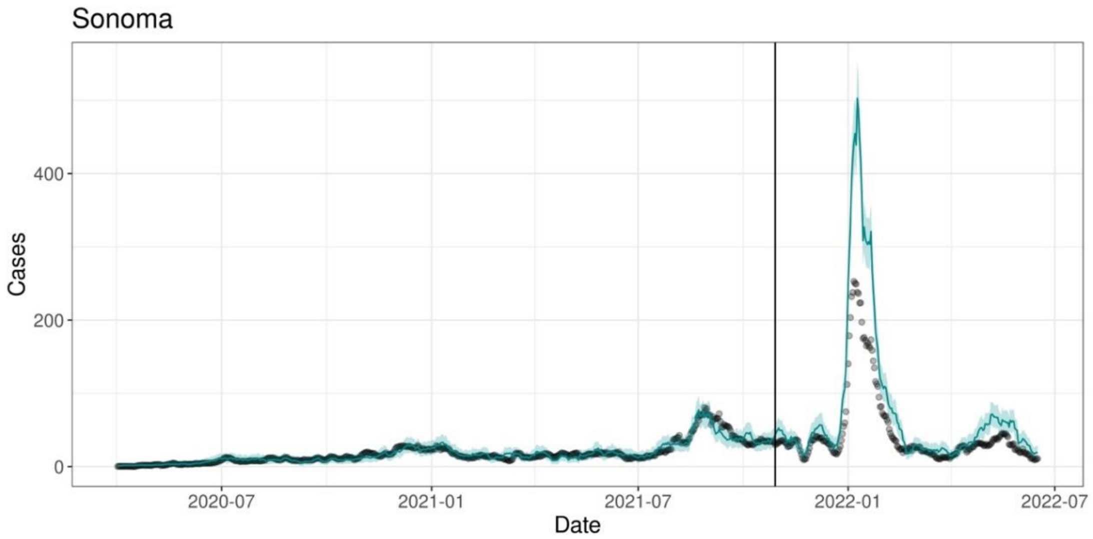

Model predictions for COVID-19 cases among children aged 5-11 years in Sonoma County.

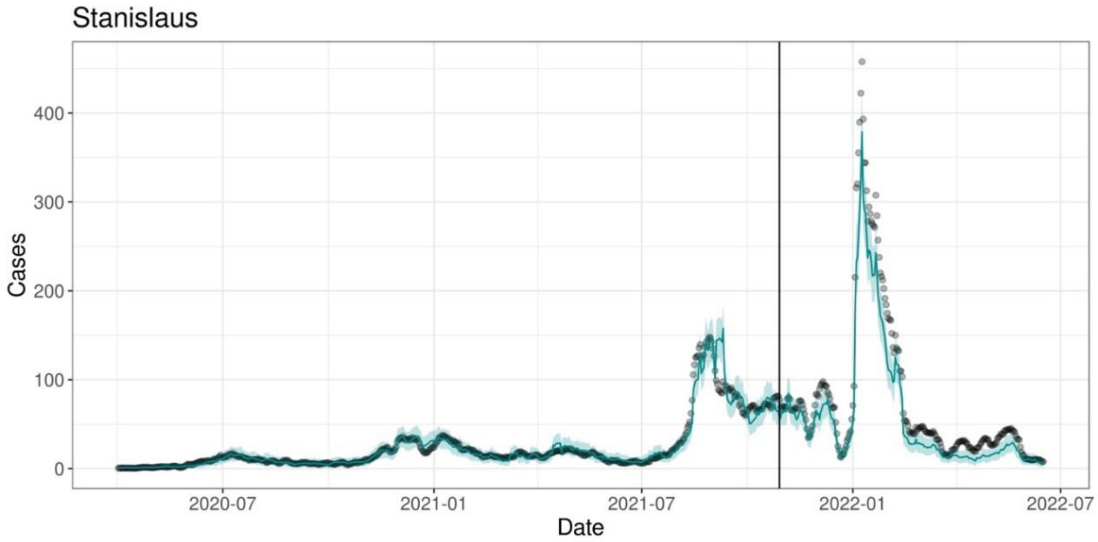

Model predictions for COVID-19 cases among children aged 5-11 years in Stanislaus County.

© 2024 Head JR et al. *JAMA Network Open.*

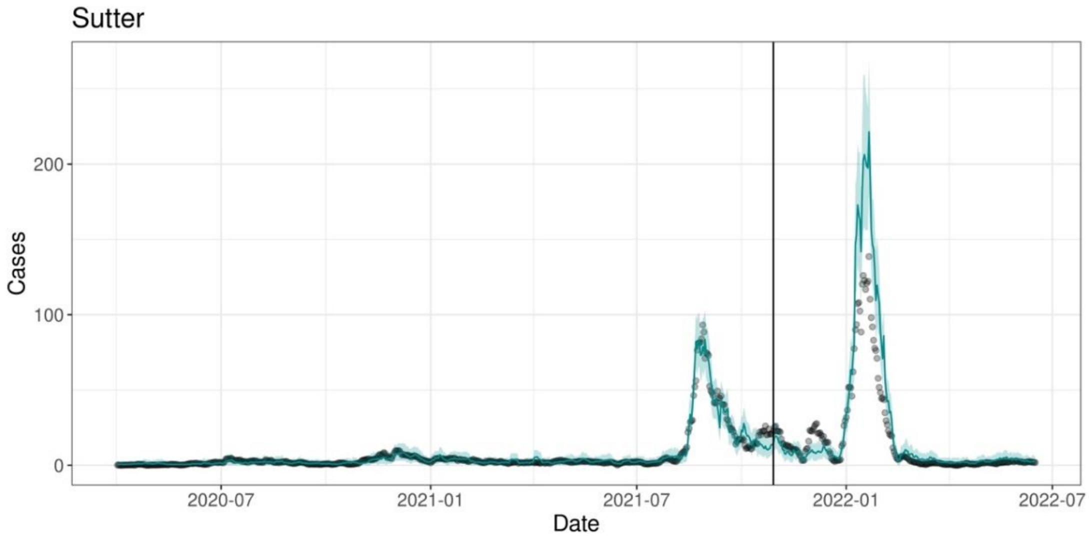

Model predictions for COVID-19 cases among children aged 5-11 years in Sutter County.

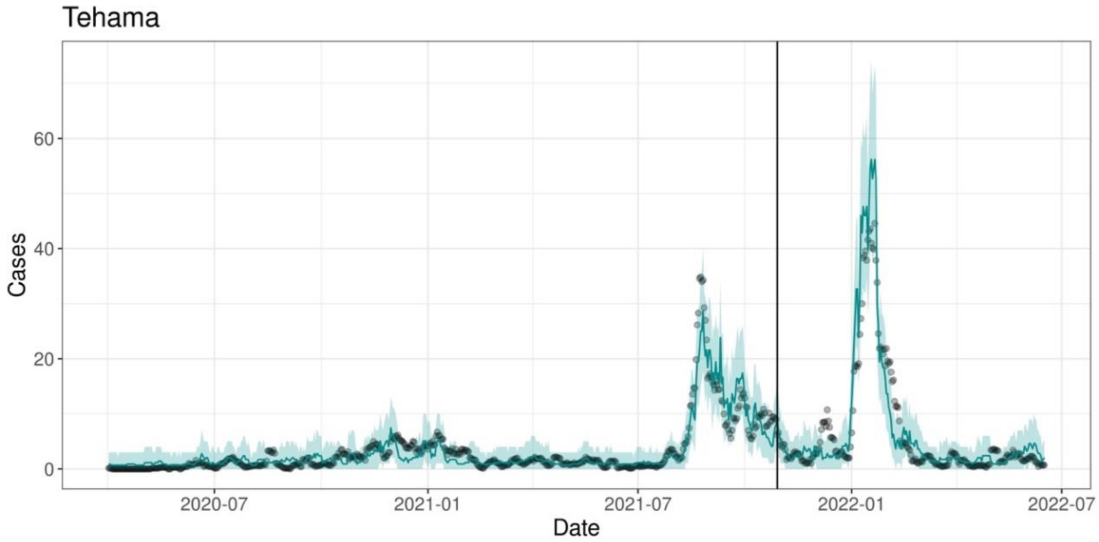

Model predictions for COVID-19 cases among children aged 5-11 years in Tehama County.

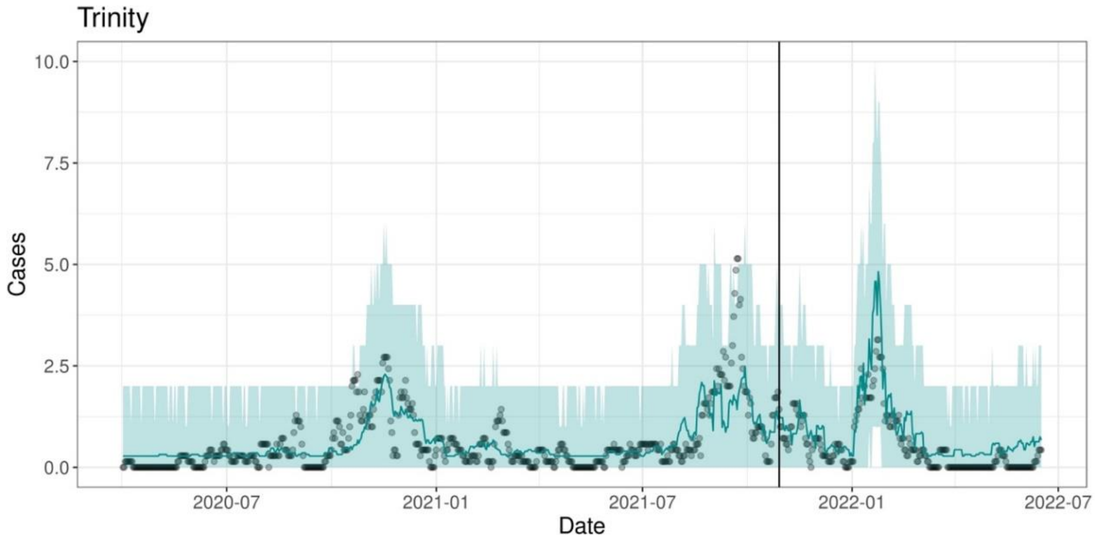

Model predictions for COVID-19 cases among children aged 5-11 years in Trinity County.

© 2024 Head JR et al. *JAMA Network Open.*

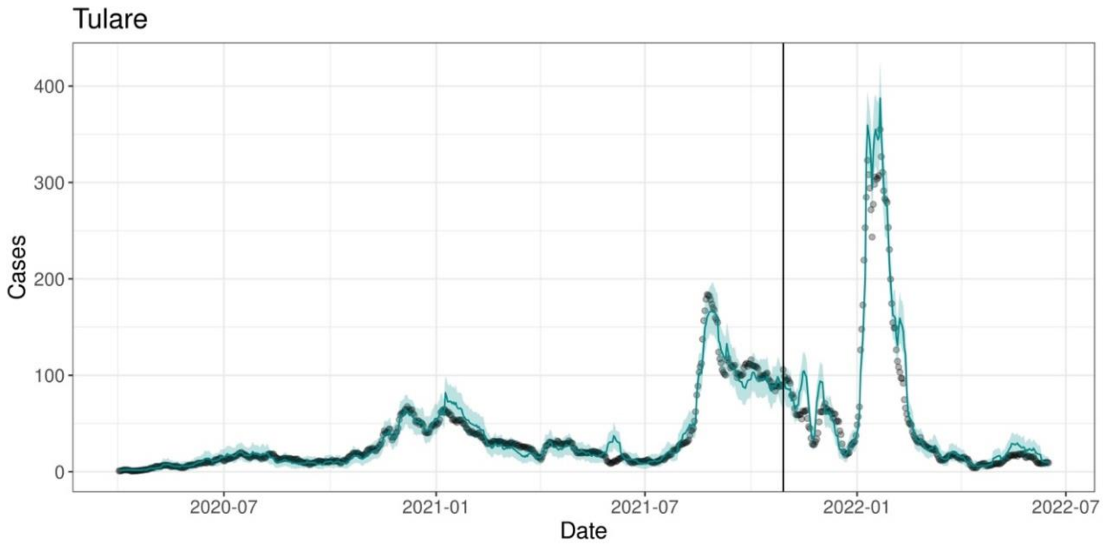

Model predictions for COVID-19 cases among children aged 5-11 years in Tulare County.

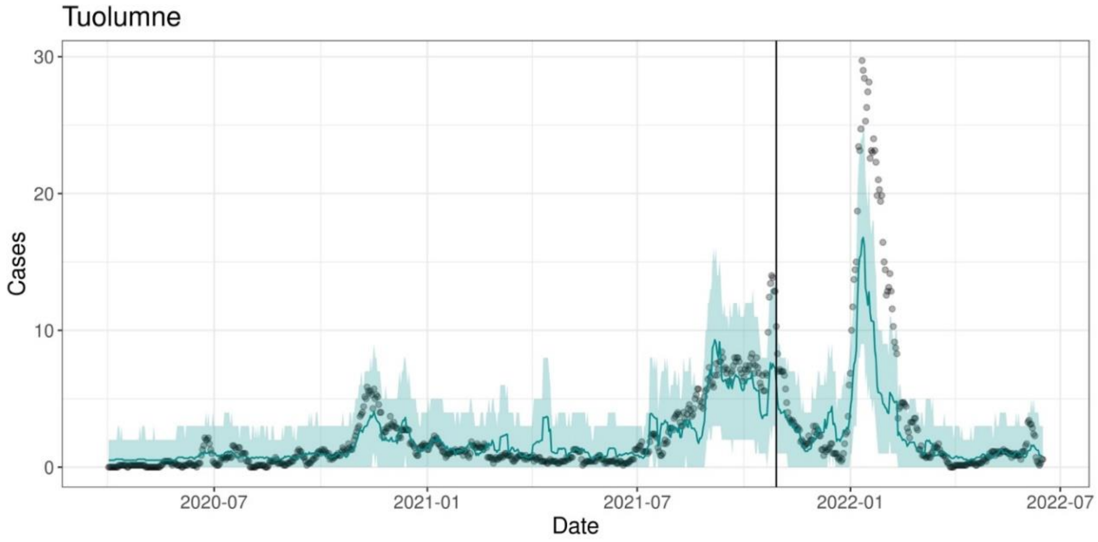

Model predictions for COVID-19 cases among children aged 5-11 years in Tuolumne County.

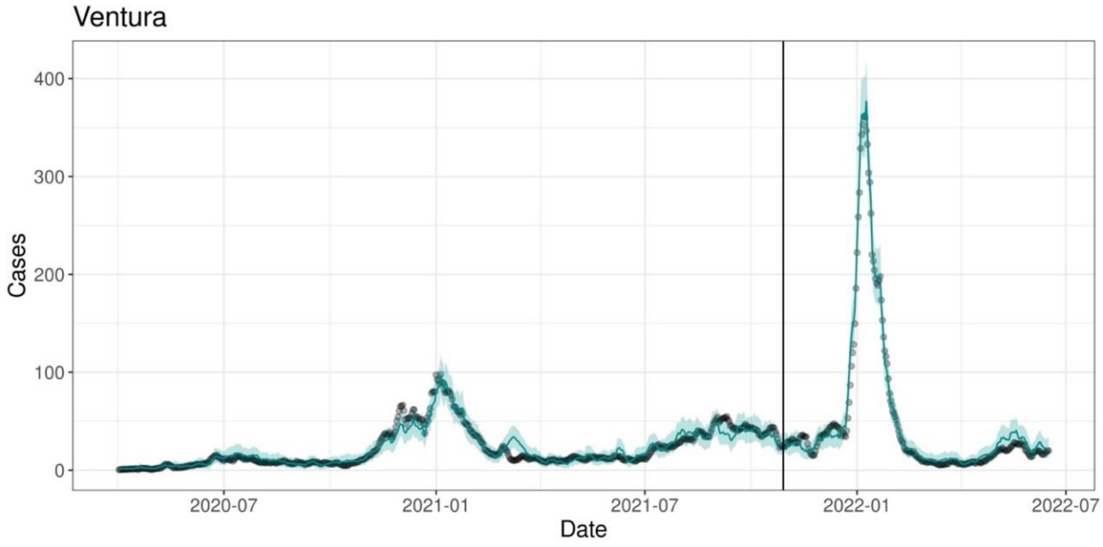

Model predictions for COVID-19 cases among children aged 5-11 years in Ventura County.

© 2024 Head JR et al. *JAMA Network Open.*

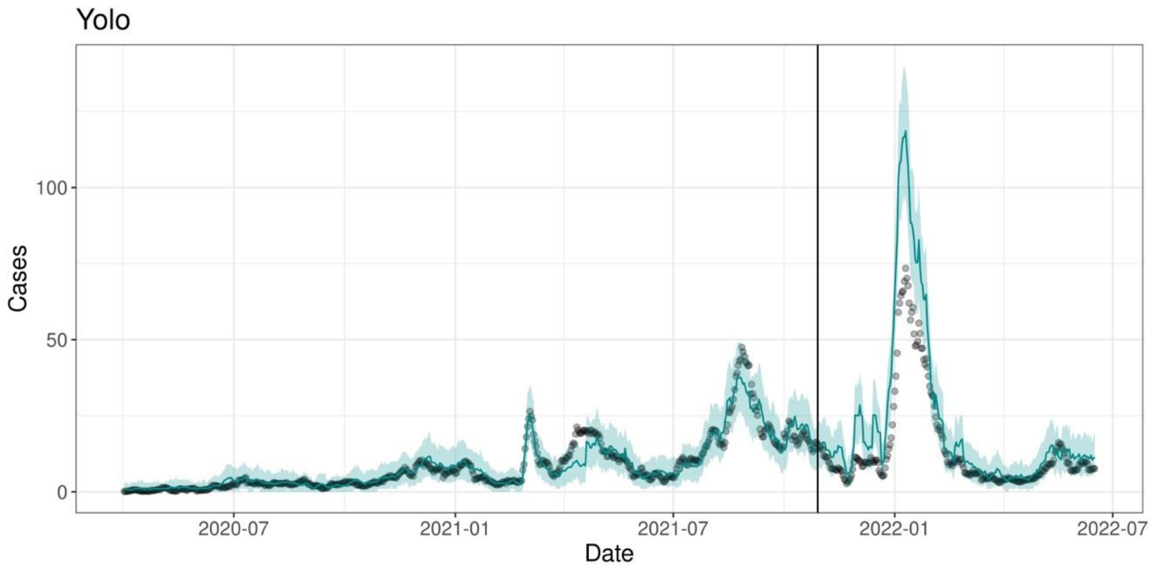

Model predictions for COVID-19 cases among children aged 5-11 years in Yolo County.

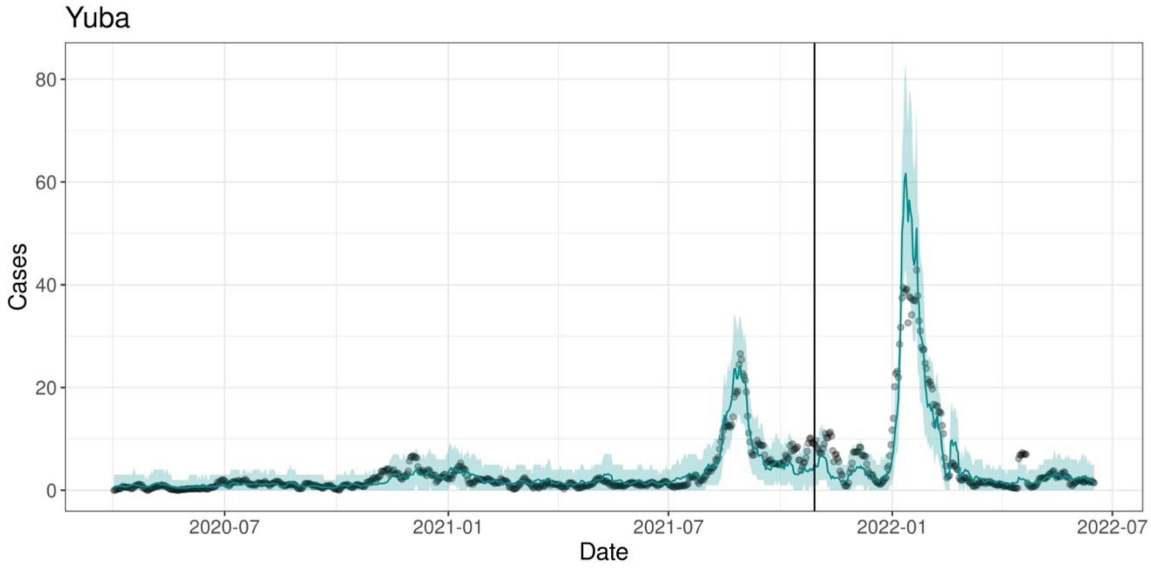

Model predictions for COVID-19 cases among children aged 5-11 years in Yuba County.

© 2024 Head JR et al. *JAMA Network Open.*

eFigures 11. Model predictions for COVID-19 hospitalizations among children aged 6-59 months. Black dots represent weekly hospitalization count, lines are model predictions, and shaded colored region is 95% prediction interval. Vertical lines are located at the time of vaccine eligibility.

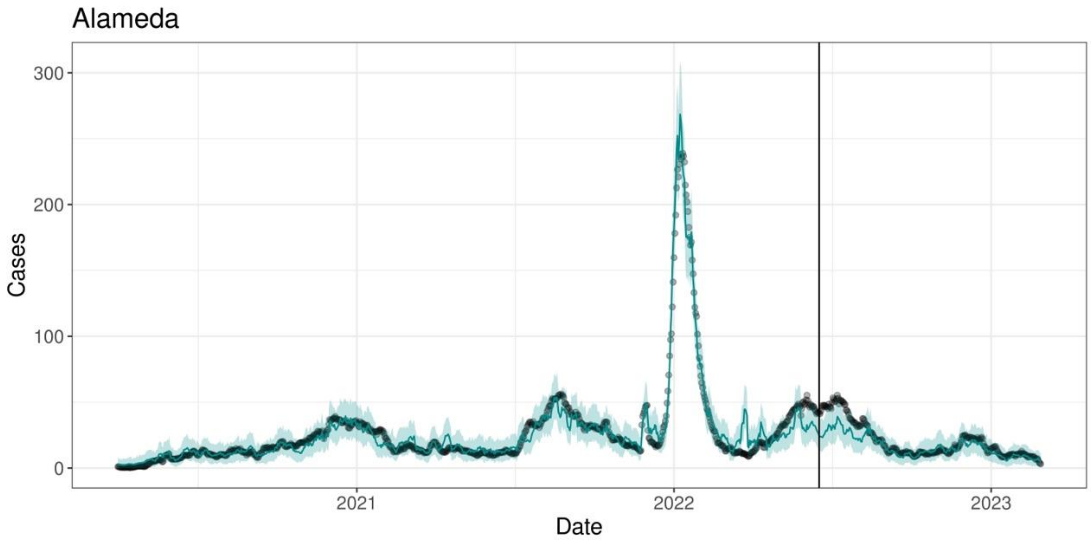

Model predictions for COVID-19 cases among children aged 6-59 months in Alameda County.

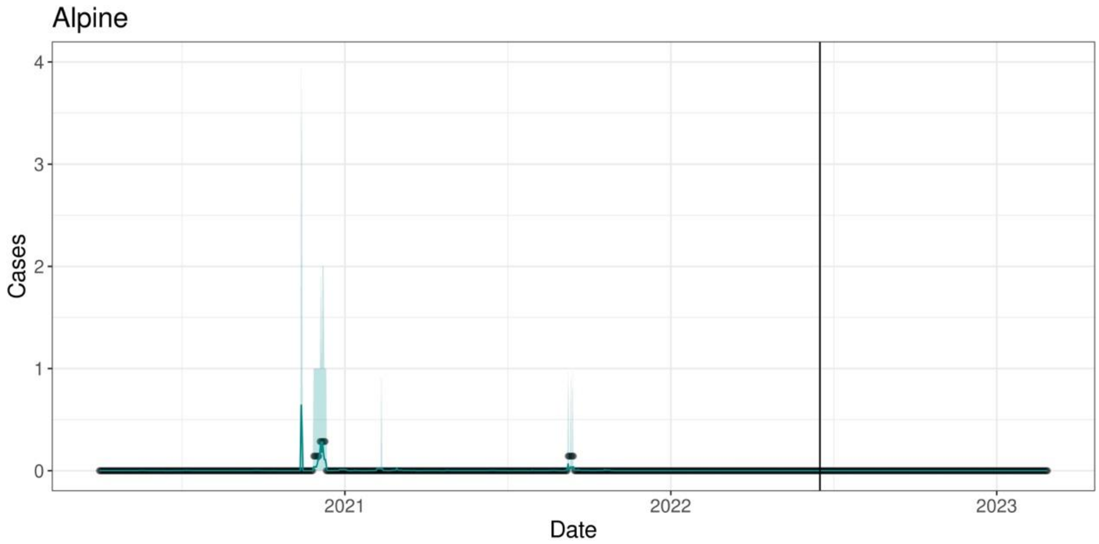

Model predictions for COVID-19 cases among children aged 6-59 months in Alpine County.

© 2024 Head JR et al. *JAMA Network Open.*

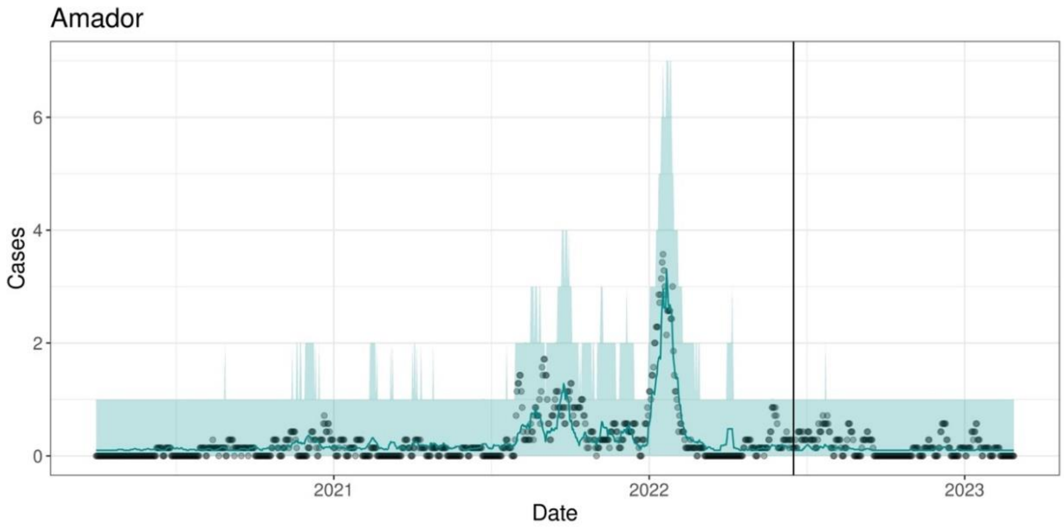

Model predictions for COVID-19 cases among children aged 6-59 months in Amador County.

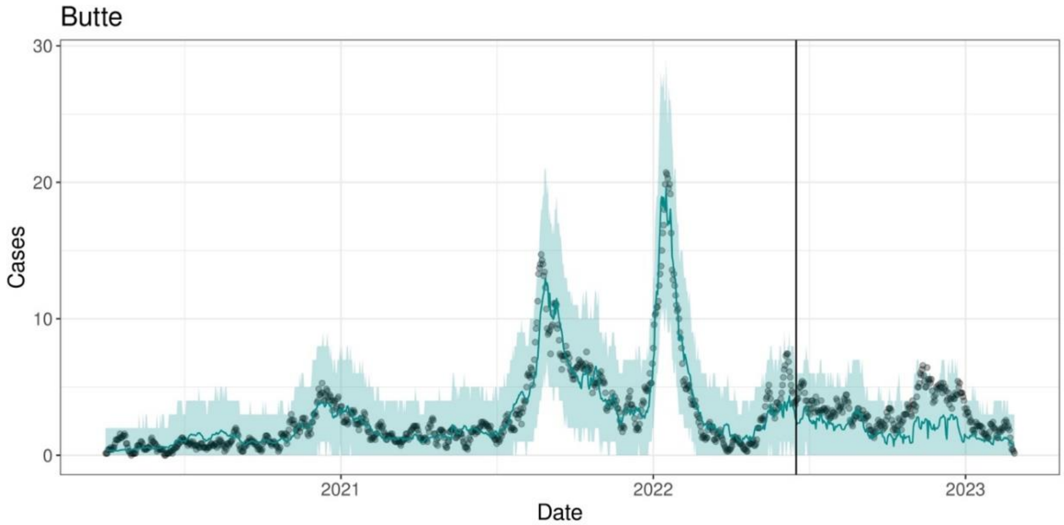

Model predictions for COVID-19 cases among children aged 6-59 months in Butte County.

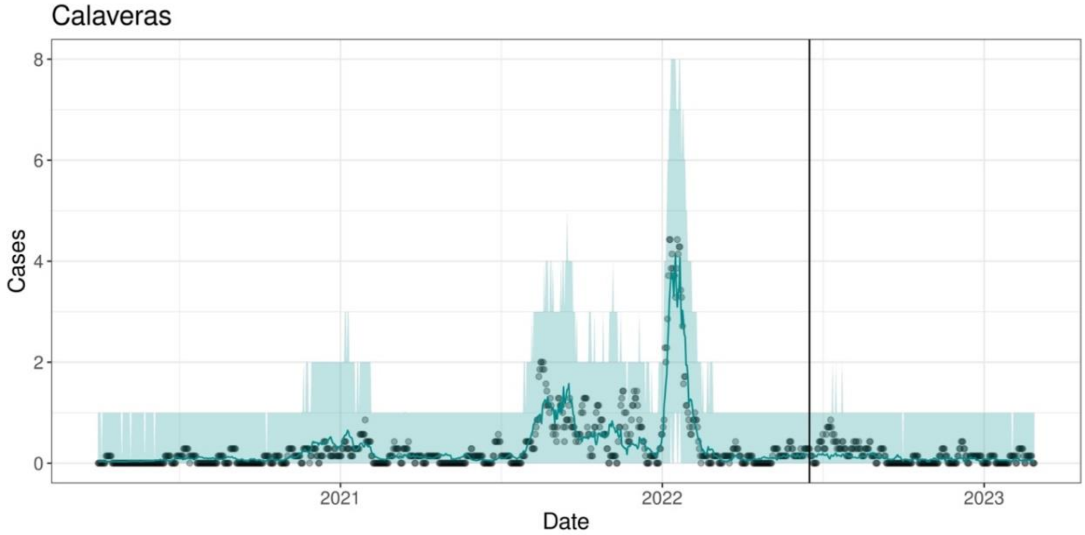

Model predictions for COVID-19 cases among children aged 6-59 months in Calaveras County.

© 2024 Head JR et al. *JAMA Network Open.*

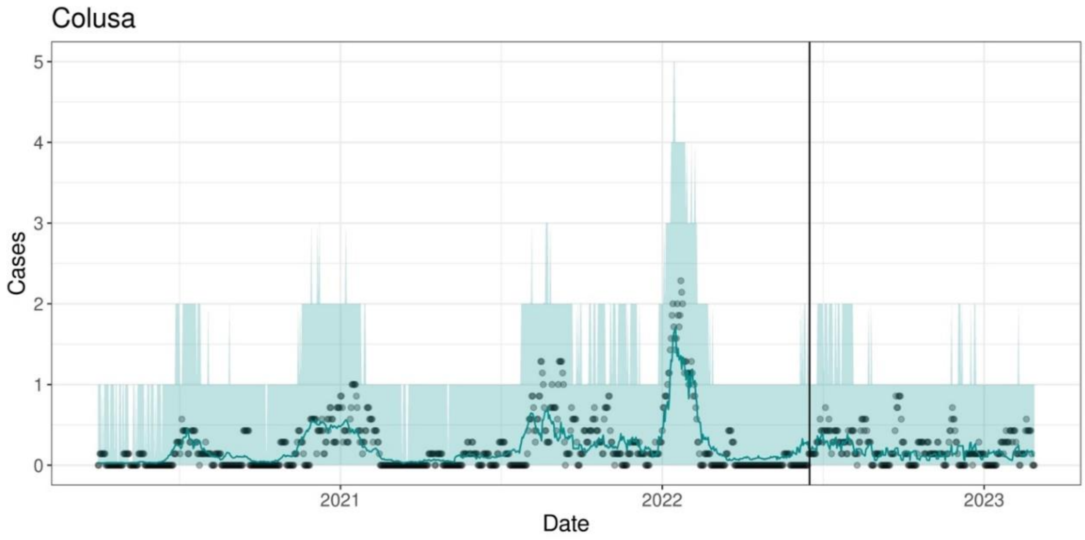

Model predictions for COVID-19 cases among children aged 6-59 months in Colusa County.

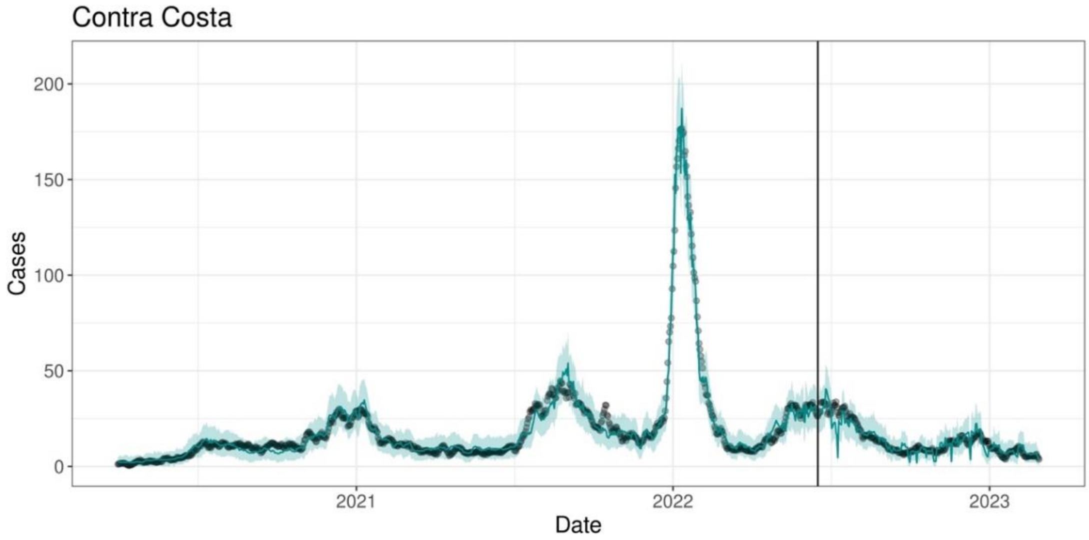

Model predictions for COVID-19 cases among children aged 6-59 months in Contra Costa County.

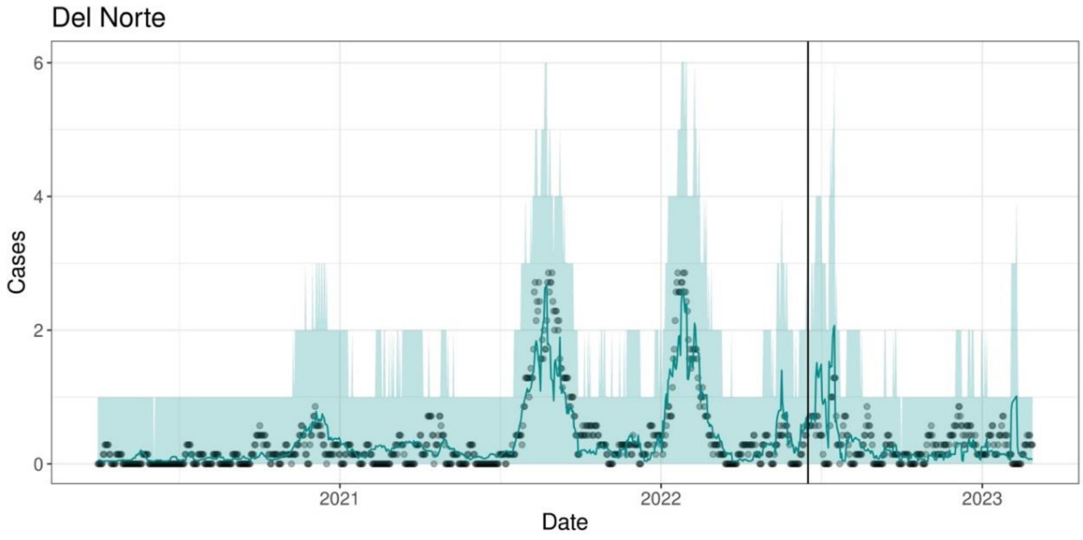

Model predictions for COVID-19 cases among children aged 6-59 months in Del Norte County.

© 2024 Head JR et al. *JAMA Network Open.*

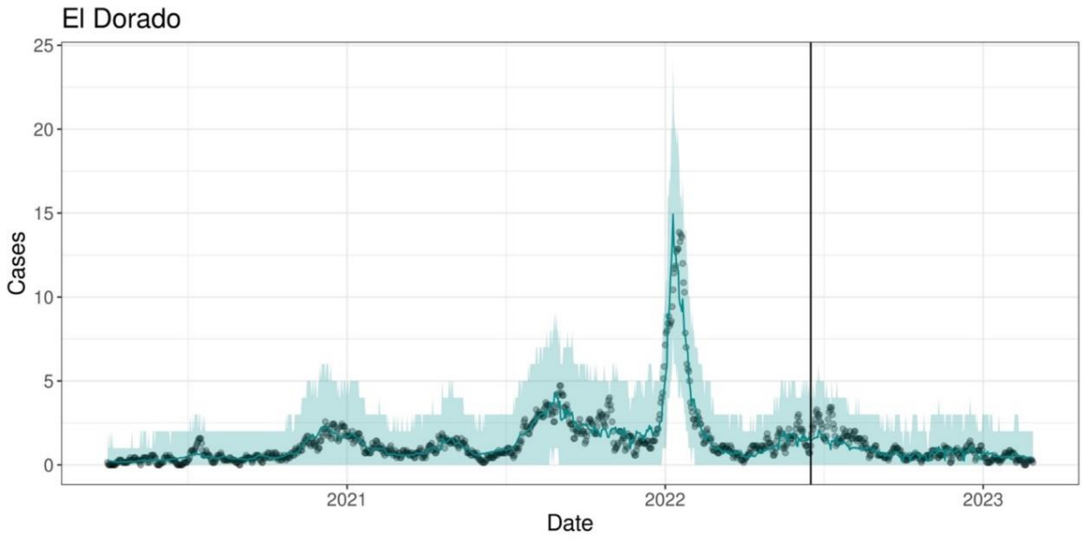

Model predictions for COVID-19 cases among children aged 6-59 months in El Dorado County.

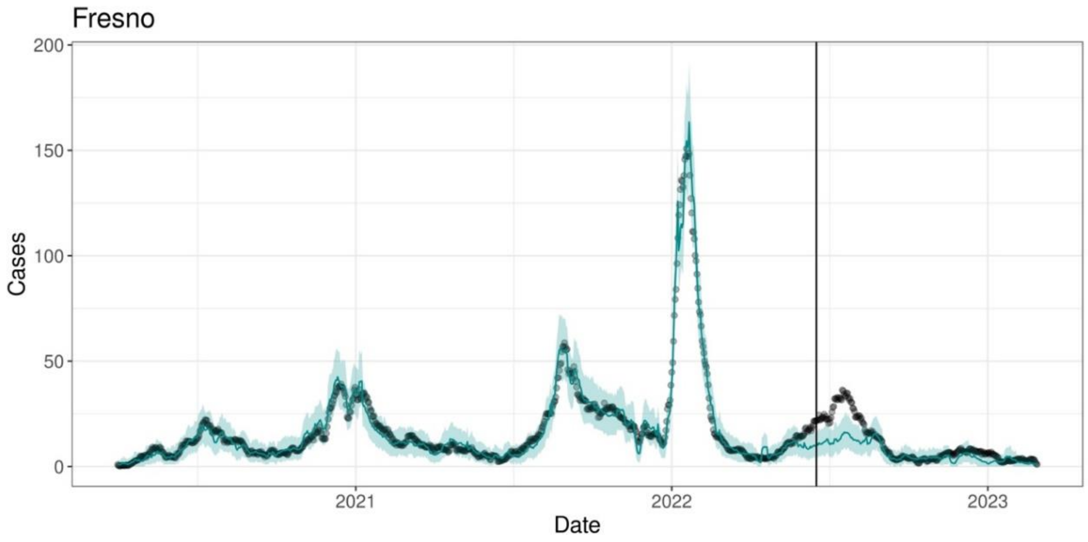

Model predictions for COVID-19 cases among children aged 6-59 months in Fresno County.

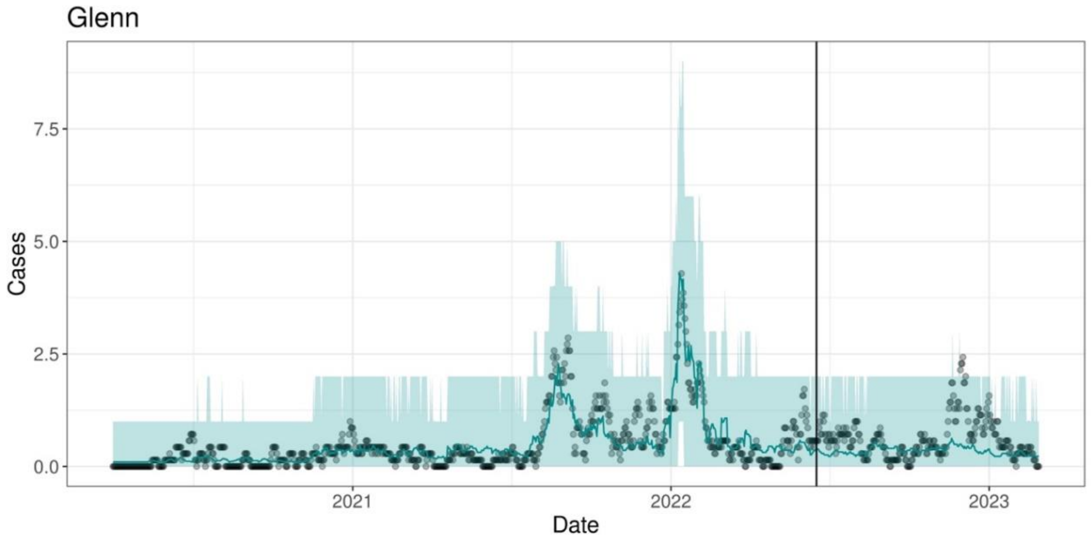

Model predictions for COVID-19 cases among children aged 6-59 months in Glenn County.

© 2024 Head JR et al. *JAMA Network Open.*

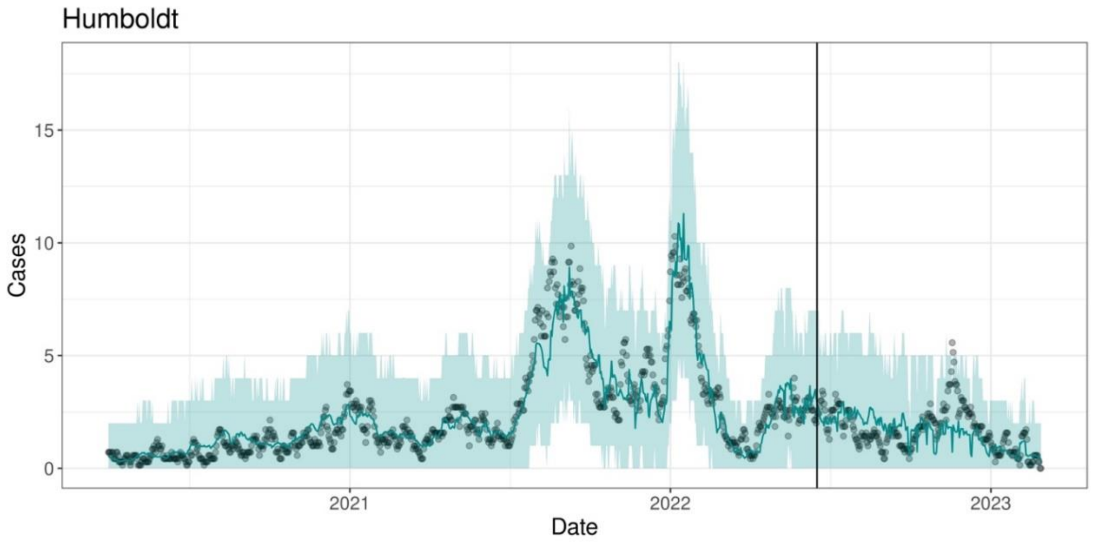

Model predictions for COVID-19 cases among children aged 6-59 months in Humboldt County.

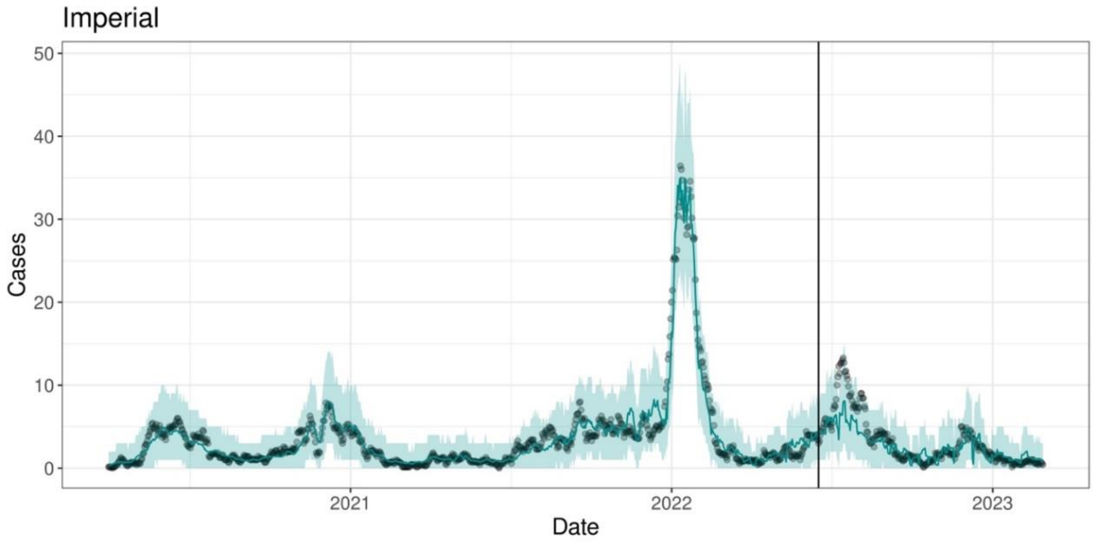

Model predictions for COVID-19 cases among children aged 6-59 months in Imperial County.

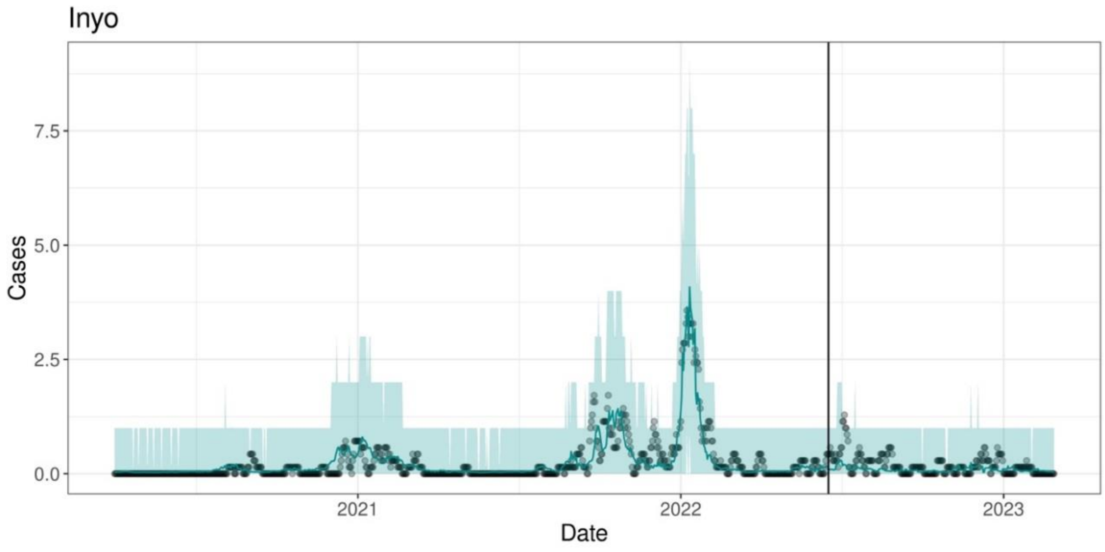

Model predictions for COVID-19 cases among children aged 6-59 months in Inyo County.

© 2024 Head JR et al. *JAMA Network Open.*

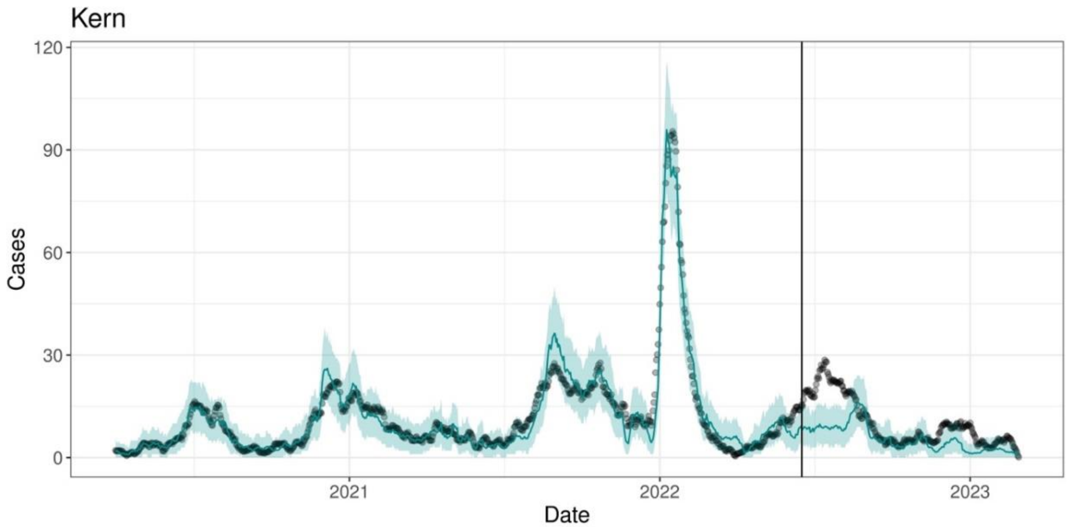

Model predictions for COVID-19 cases among children aged 6-59 months in Kern County.

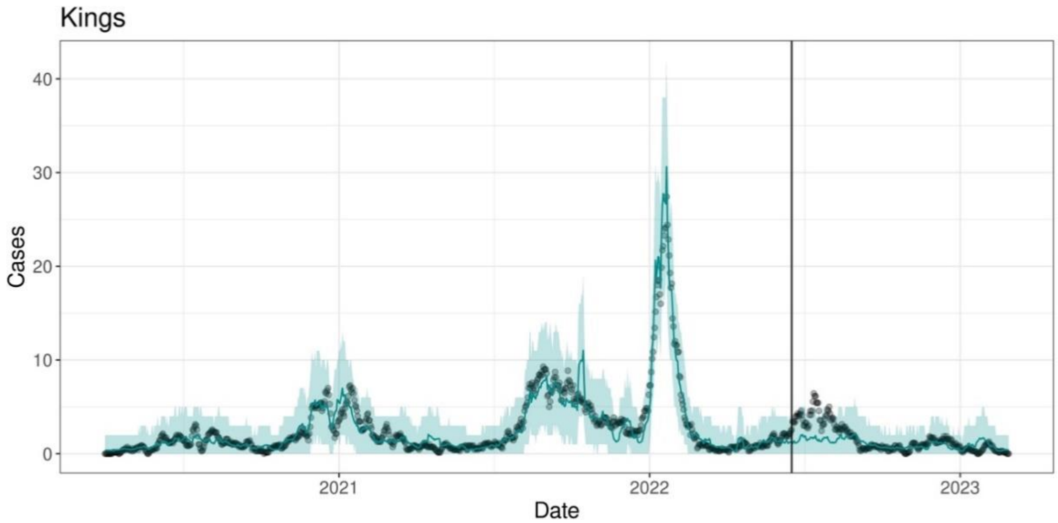

Model predictions for COVID-19 cases among children aged 6-59 months in Kings County.

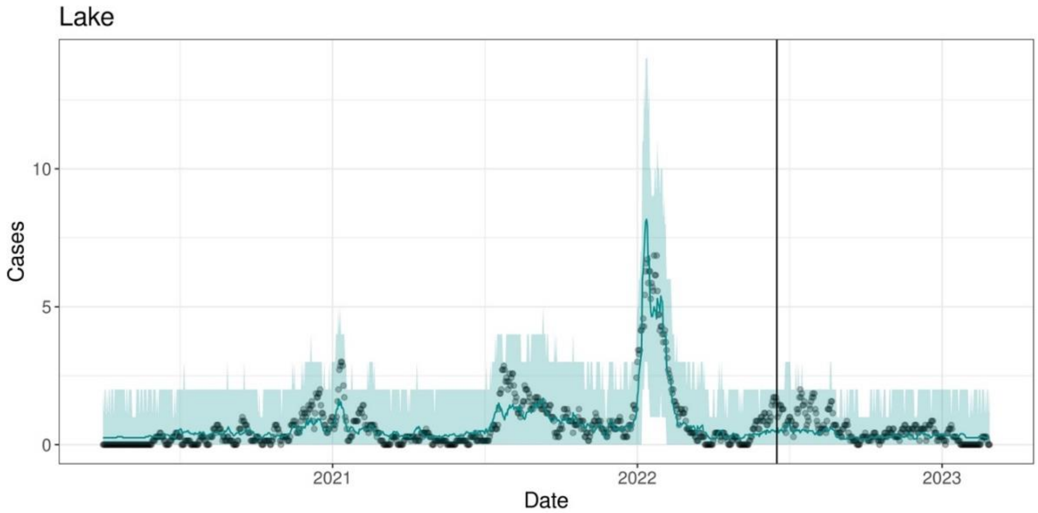

Model predictions for COVID-19 cases among children aged 6-59 months in Lake County.

© 2024 Head JR et al. *JAMA Network Open.*

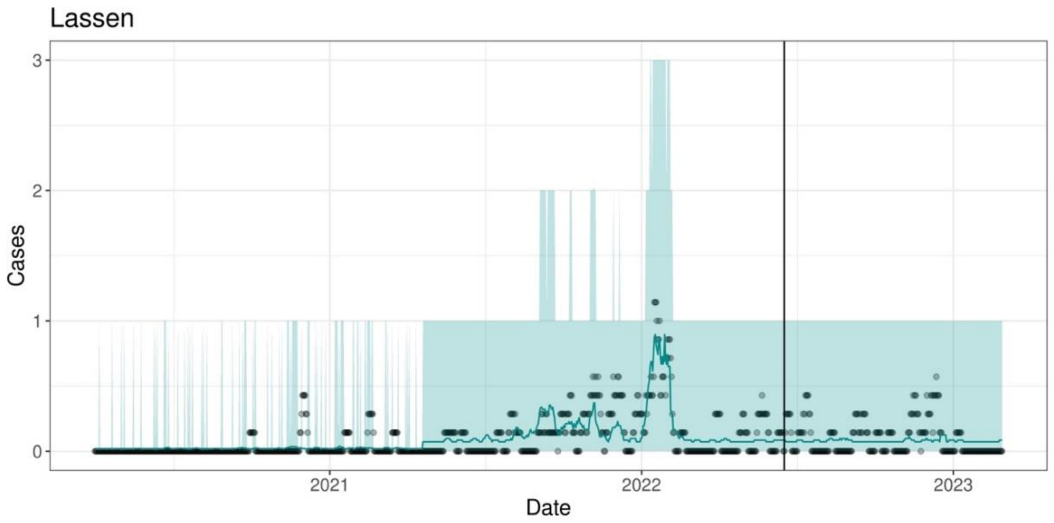

Model predictions for COVID-19 cases among children aged 6-59 months in Lassen County.

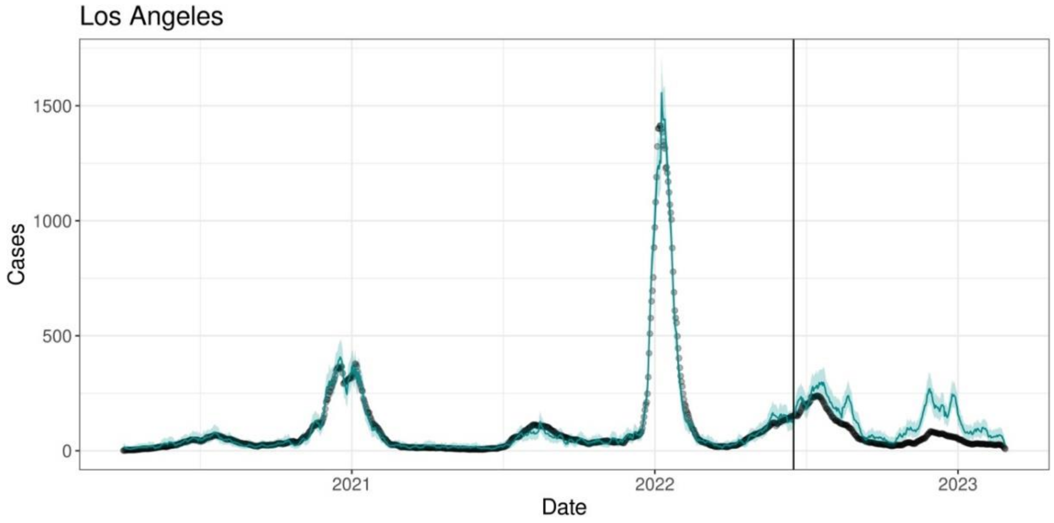

Model predictions for COVID-19 cases among children aged 6-59 months in Los Angeles County.

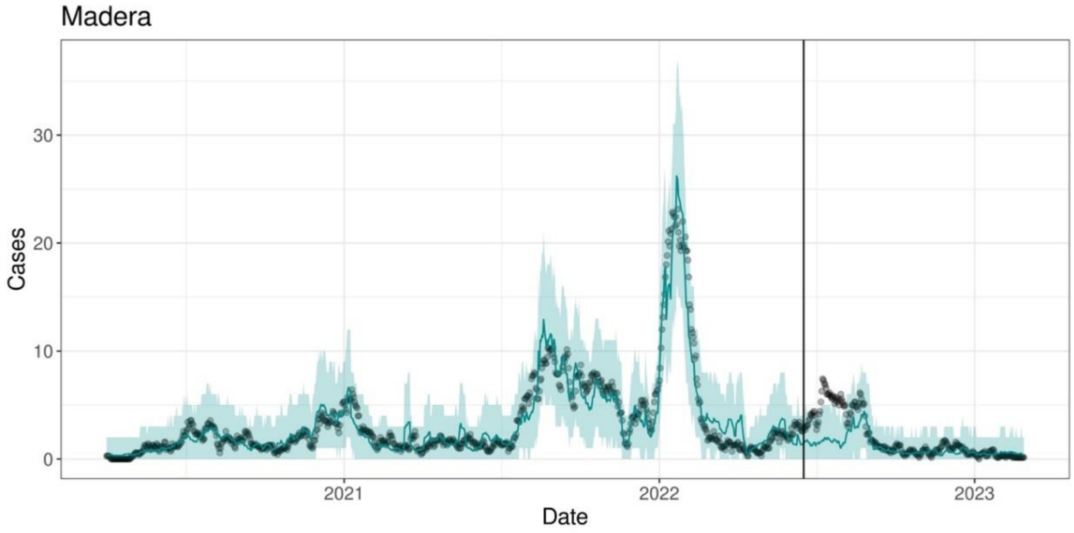

Model predictions for COVID-19 cases among children aged 6-59 months in Madera County.

© 2024 Head JR et al. *JAMA Network Open.*

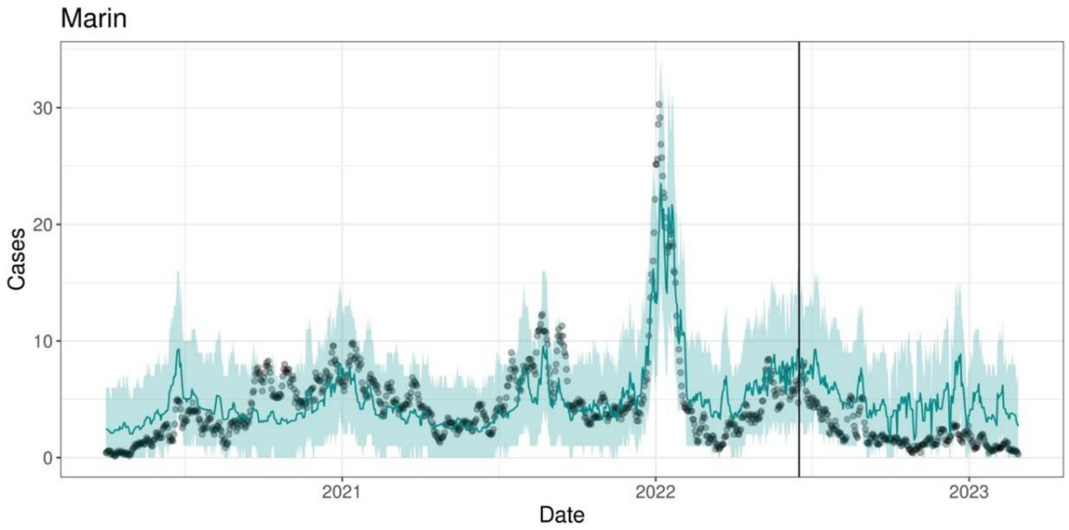

Model predictions for COVID-19 cases among children aged 6-59 months in Marin County.

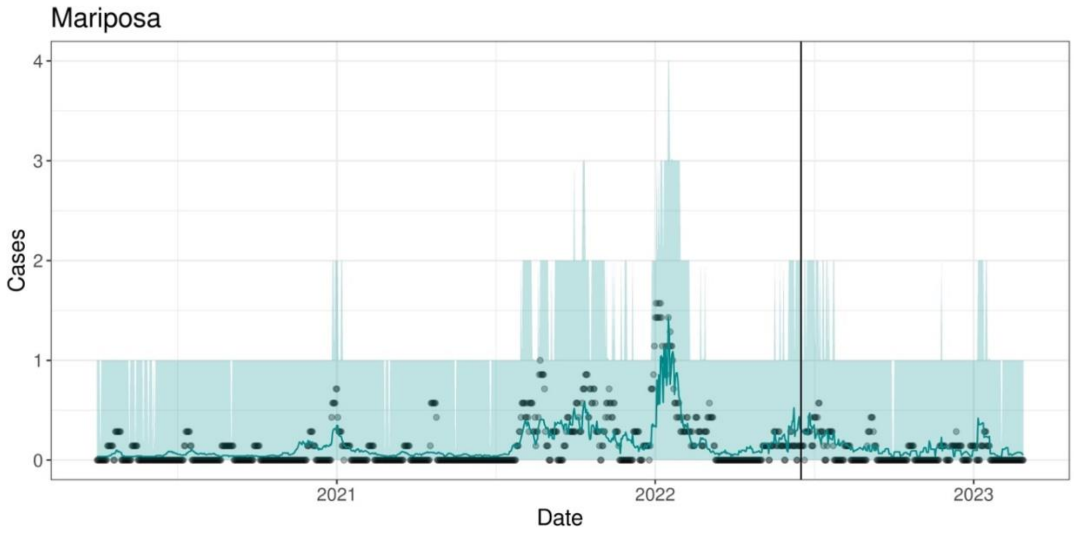

Model predictions for COVID-19 cases among children aged 6-59 months in Mariposa County.

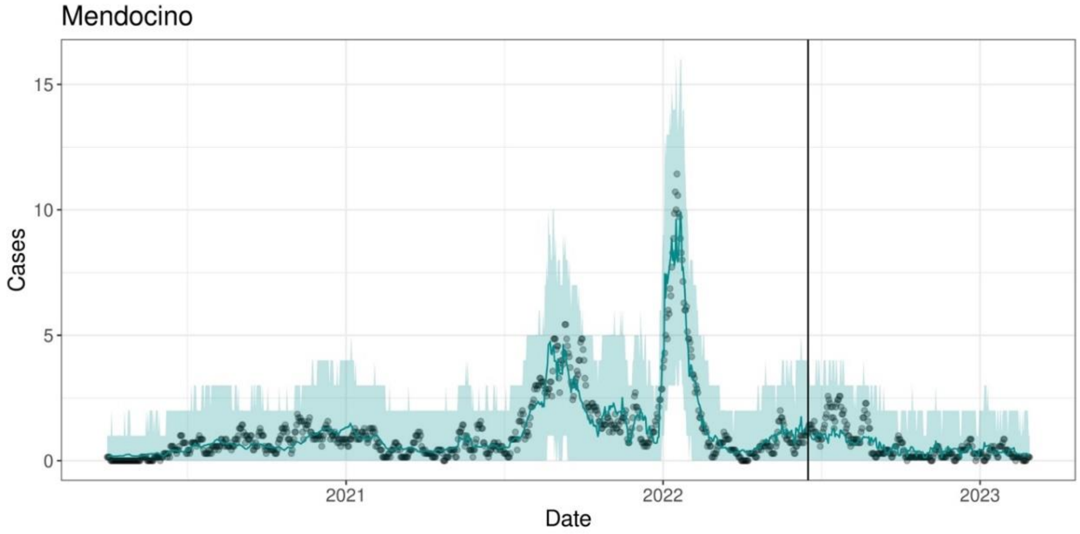

Model predictions for COVID-19 cases among children aged 6-59 months in Mendocino County.

© 2024 Head JR et al. *JAMA Network Open.*

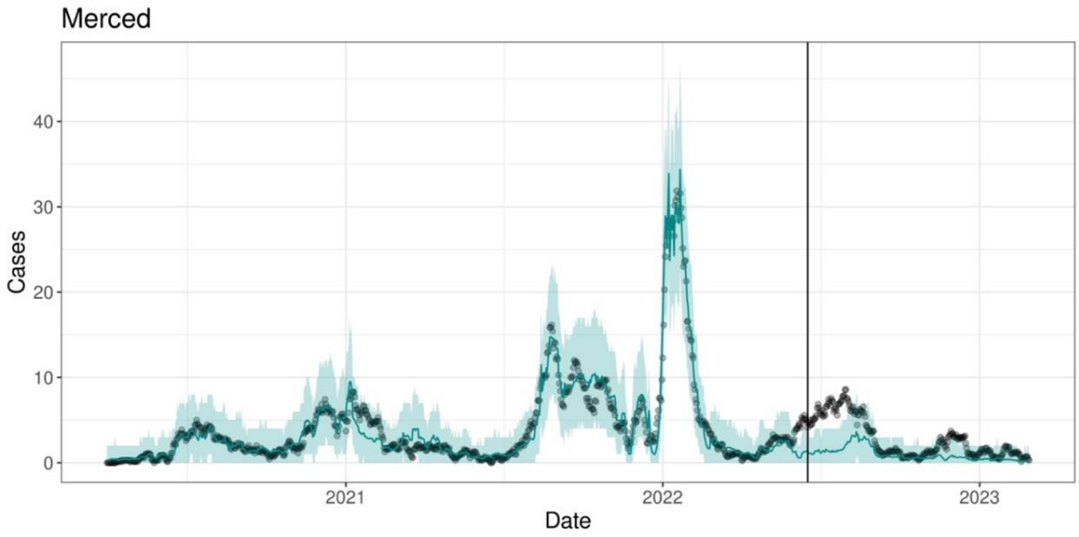

Model predictions for COVID-19 cases among children aged 6-59 months in Merced County.

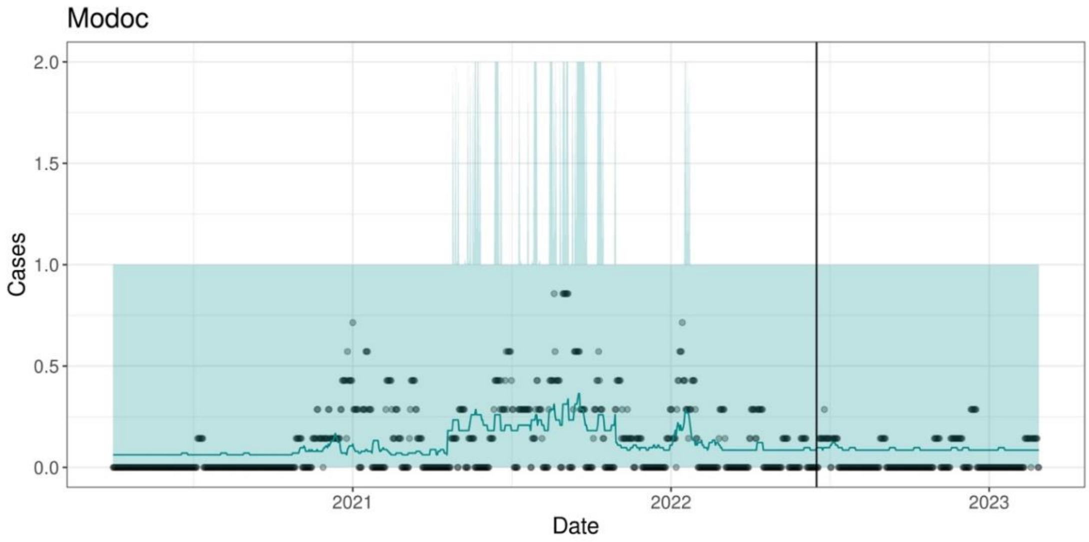

Model predictions for COVID-19 cases among children aged 6-59 months in Modoc County.

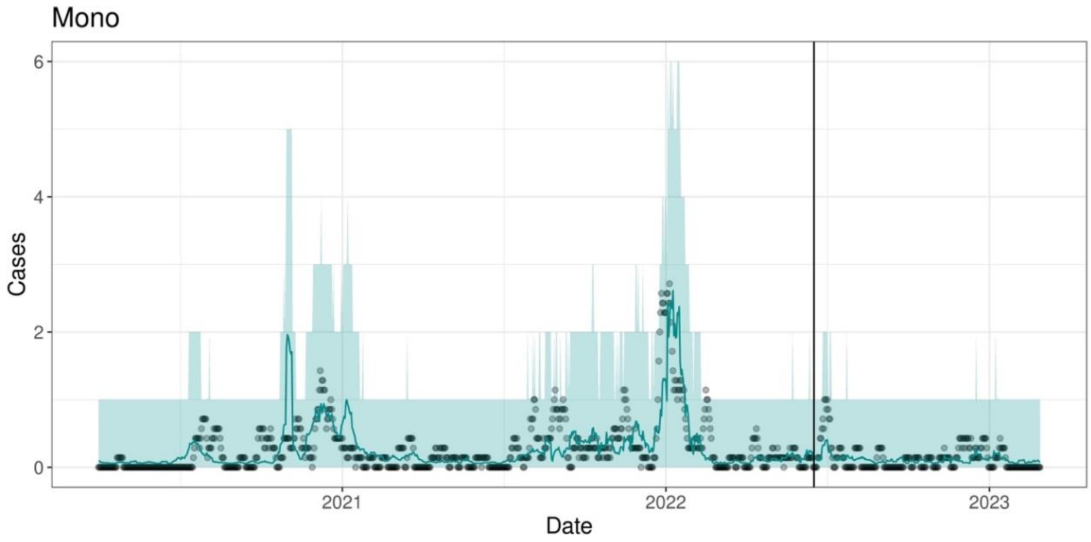

Model predictions for COVID-19 cases among children aged 6-59 months in Mono County.

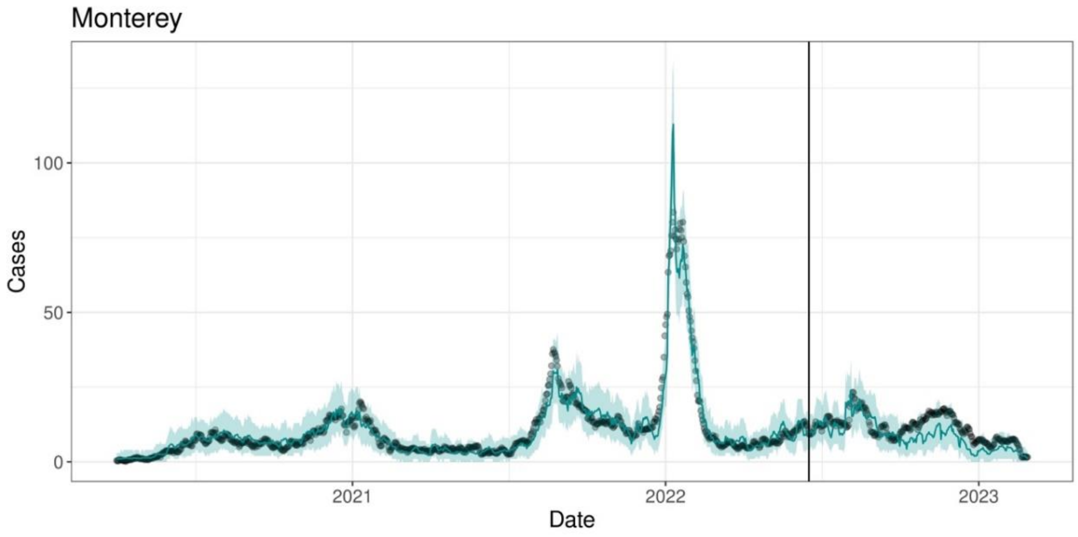

Model predictions for COVID-19 cases among children aged 6-59 months in Monterey County.

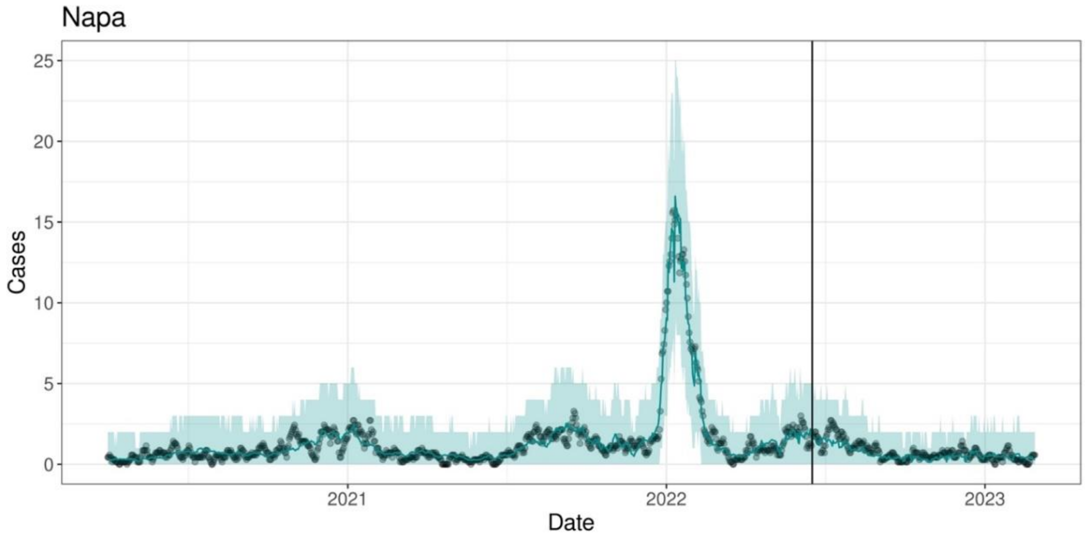

Model predictions for COVID-19 cases among children aged 6-59 months in Napa County.

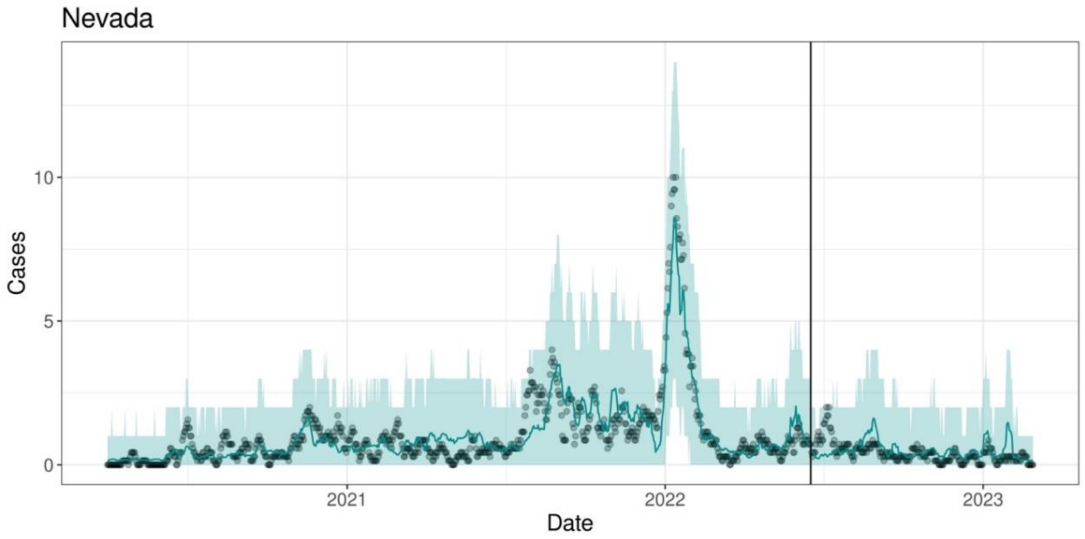

Model predictions for COVID-19 cases among children aged 6-59 months in Nevada County.

© 2024 Head JR et al. *JAMA Network Open.*

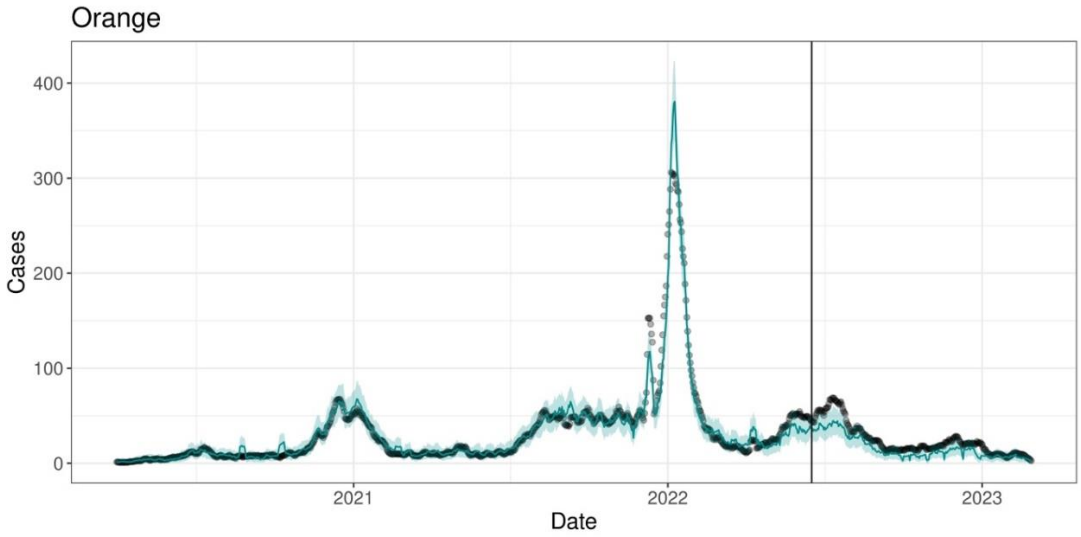

Model predictions for COVID-19 cases among children aged 6-59 months in Orange County.

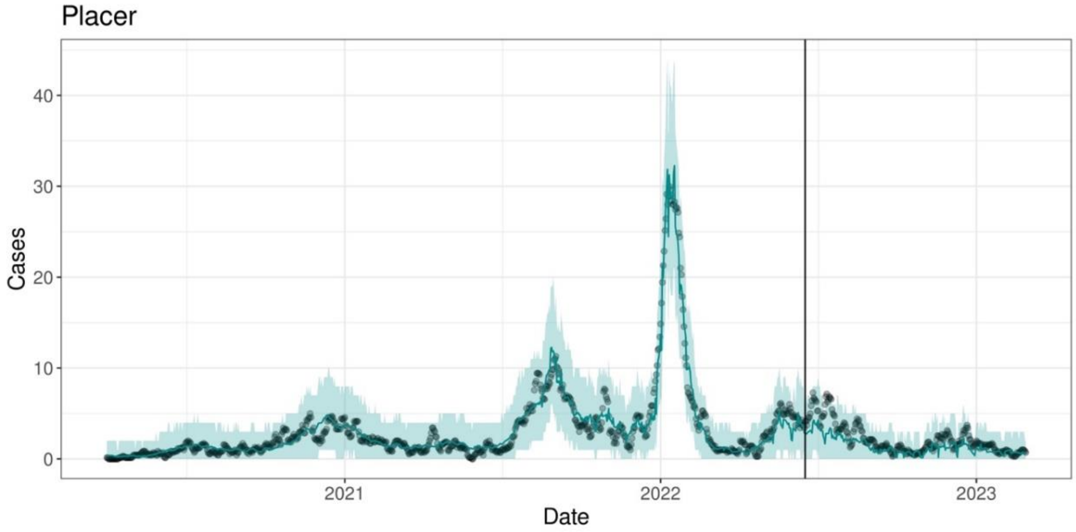

Model predictions for COVID-19 cases among children aged 6-59 months in Placer County.

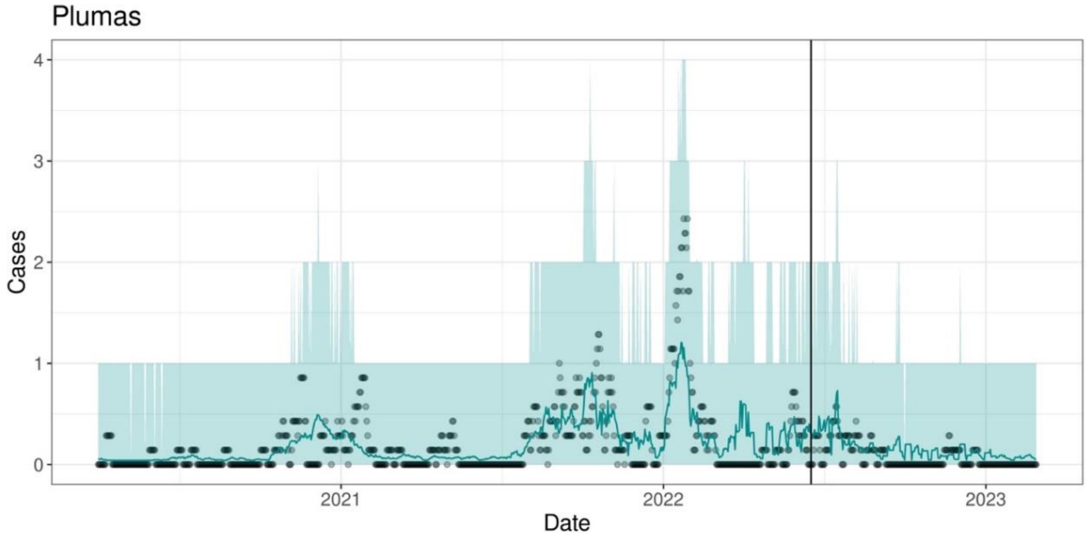

Model predictions for COVID-19 cases among children aged 6-59 months in Plumas County.

© 2024 Head JR et al. *JAMA Network Open.*

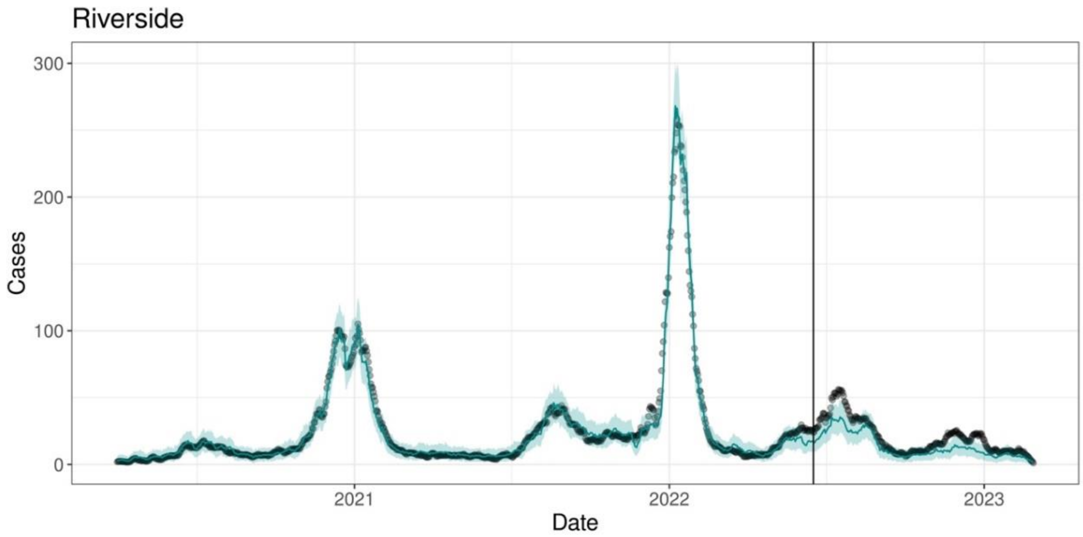

Model predictions for COVID-19 cases among children aged 6-59 months in Riverside County.

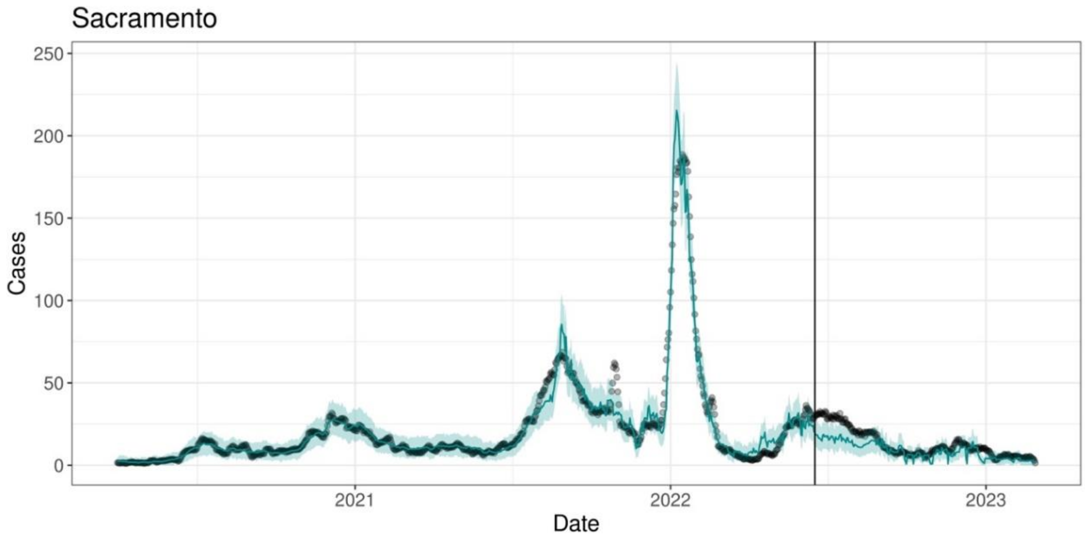

Model predictions for COVID-19 cases among children aged 6-59 months in Sacramento County.

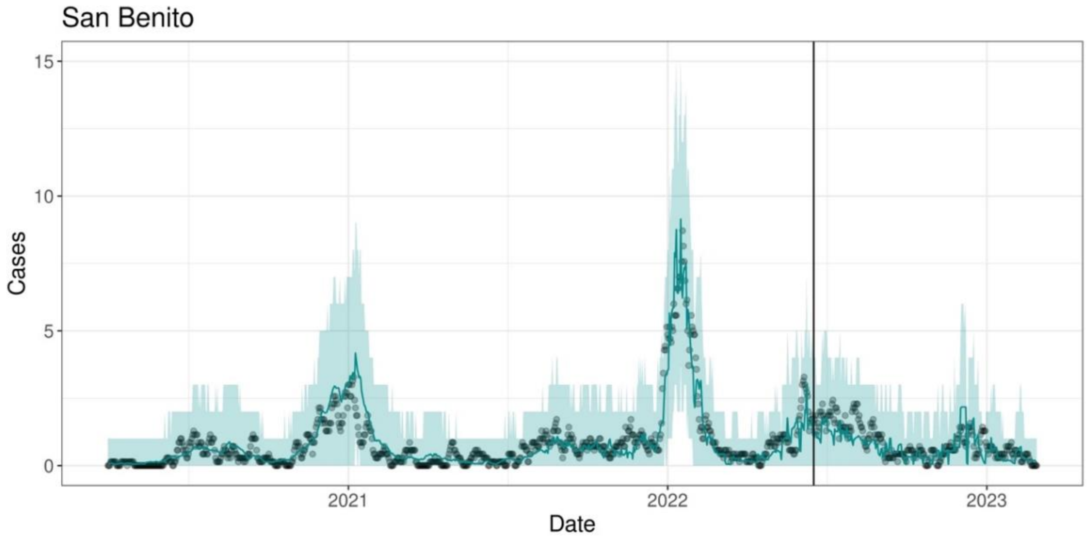

Model predictions for COVID-19 cases among children aged 6-59 months in San Benito County.

© 2024 Head JR et al. *JAMA Network Open.*

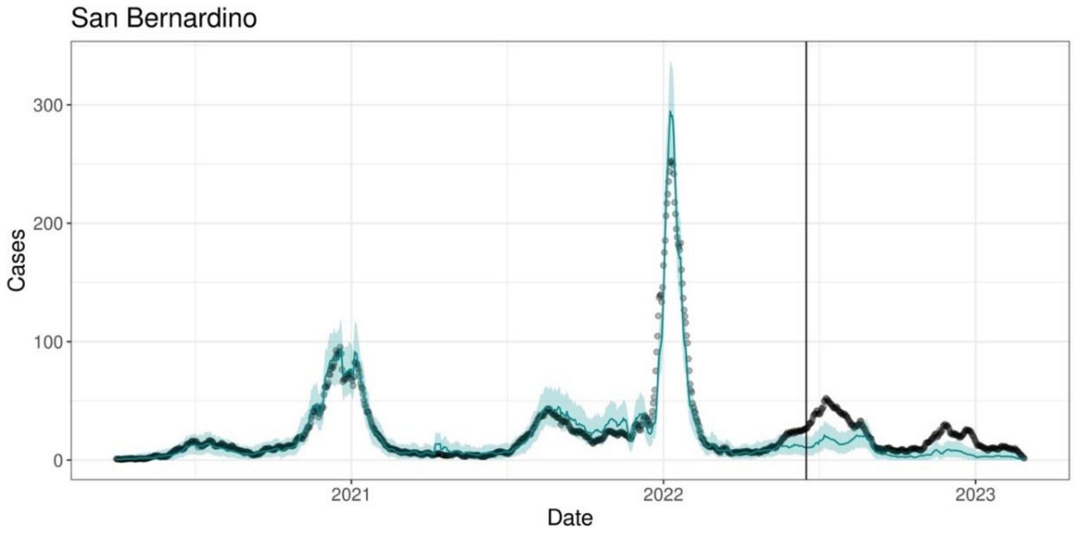

Model predictions for COVID-19 cases among children aged 6-59 months in San Bernardino County.

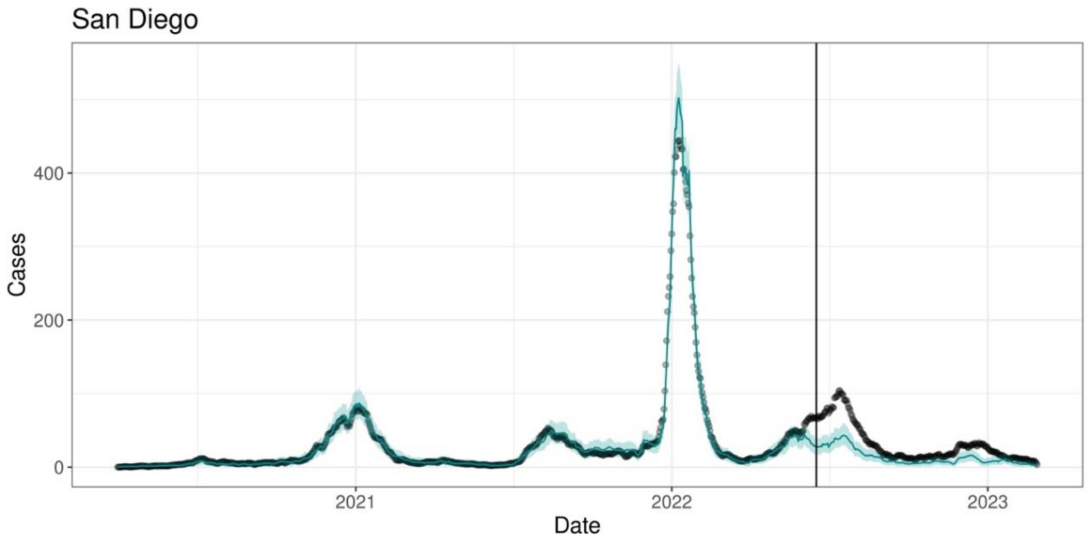

Model predictions for COVID-19 cases among children aged 6-59 months in San Diego County.

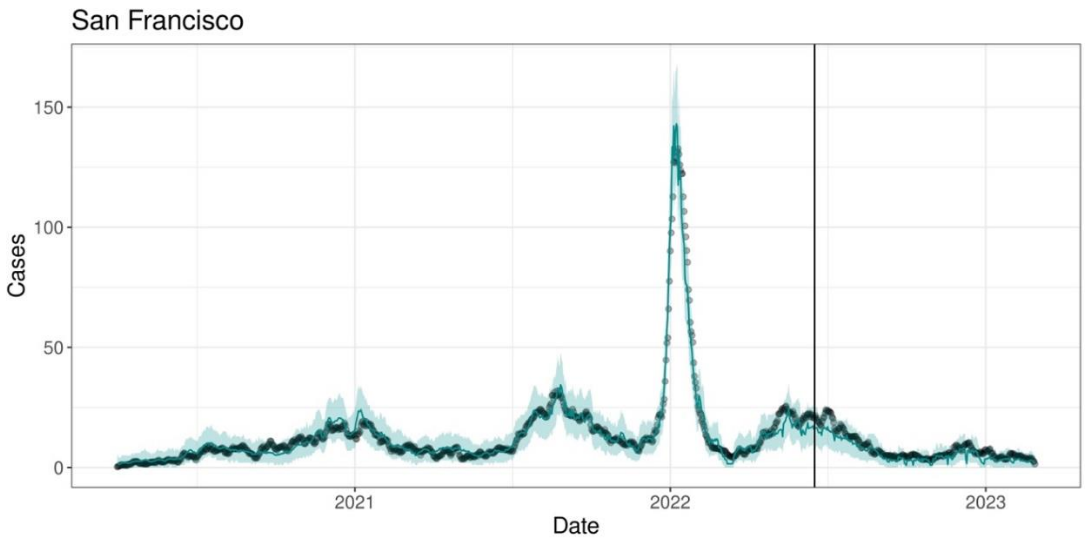

Model predictions for COVID-19 cases among children aged 6-59 months in San Francisco County.

© 2024 Head JR et al. *JAMA Network Open.*

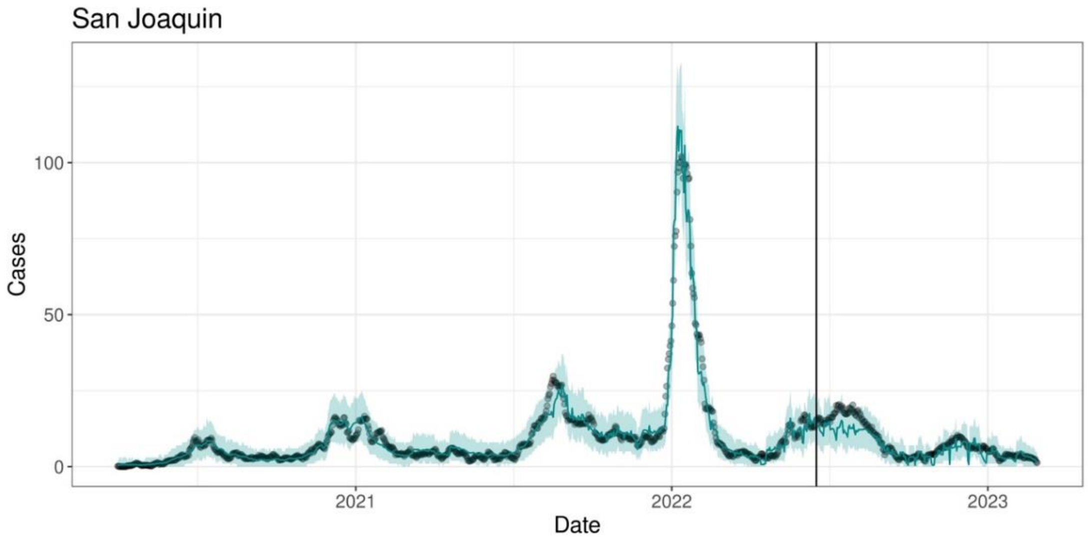

Model predictions for COVID-19 cases among children aged 6-59 months in San Joaquin County.

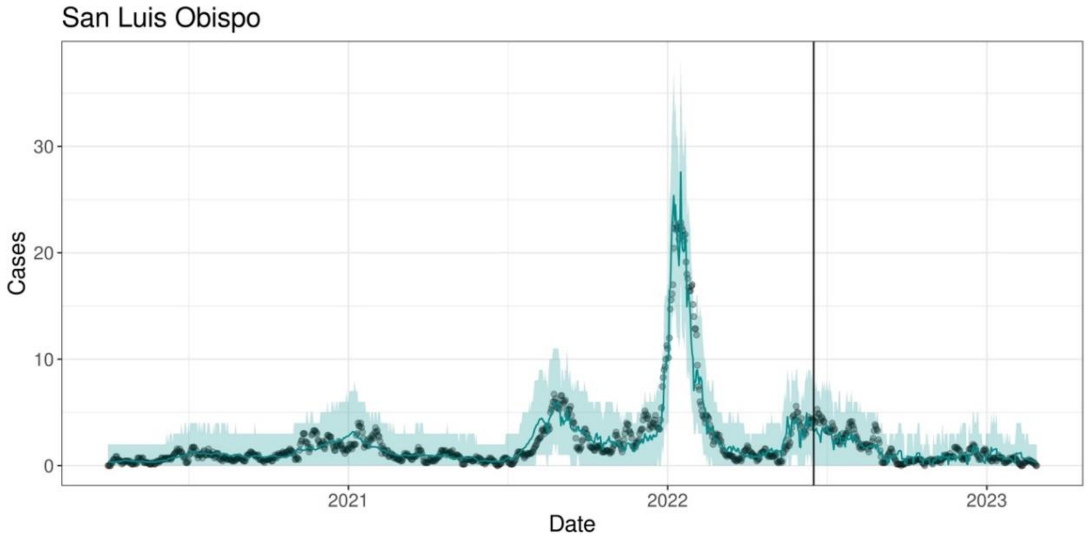

Model predictions for COVID-19 cases among children aged 6-59 months in San Luis Obispo County.

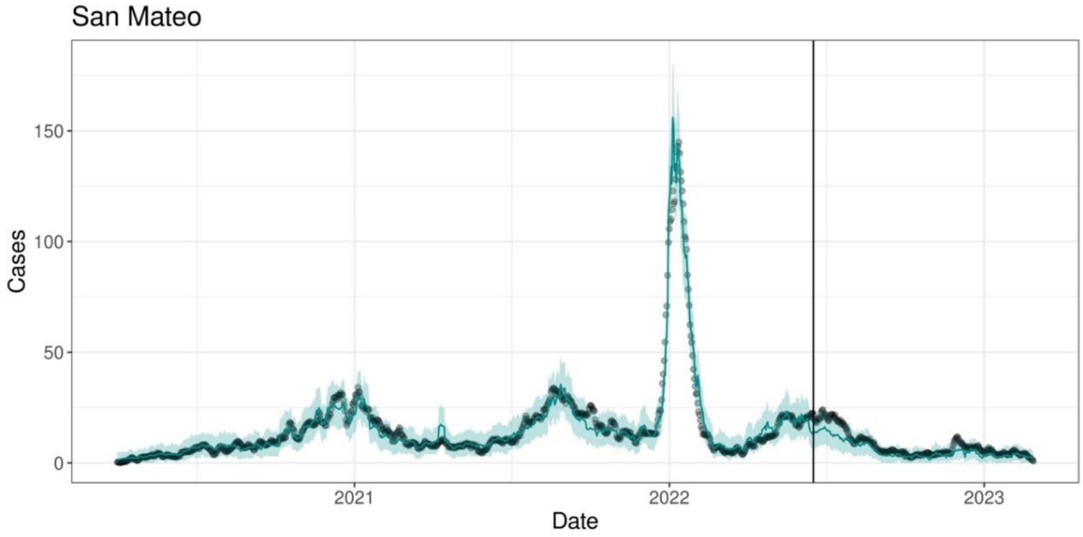

Model predictions for COVID-19 cases among children aged 6-59 months in San Mateo County.

© 2024 Head JR et al. *JAMA Network Open.*

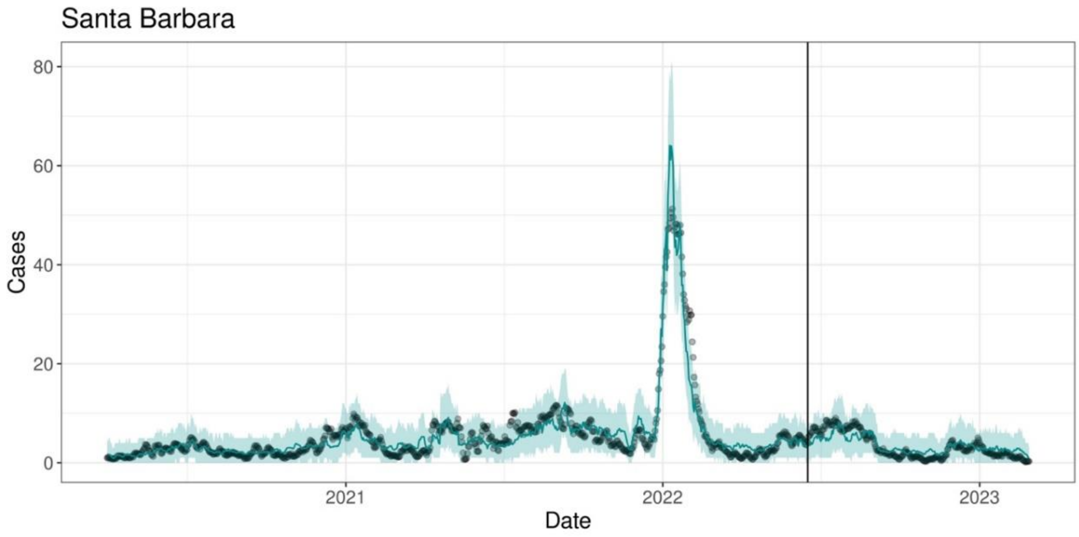

Model predictions for COVID-19 cases among children aged 6-59 months in Santa Barbara County.

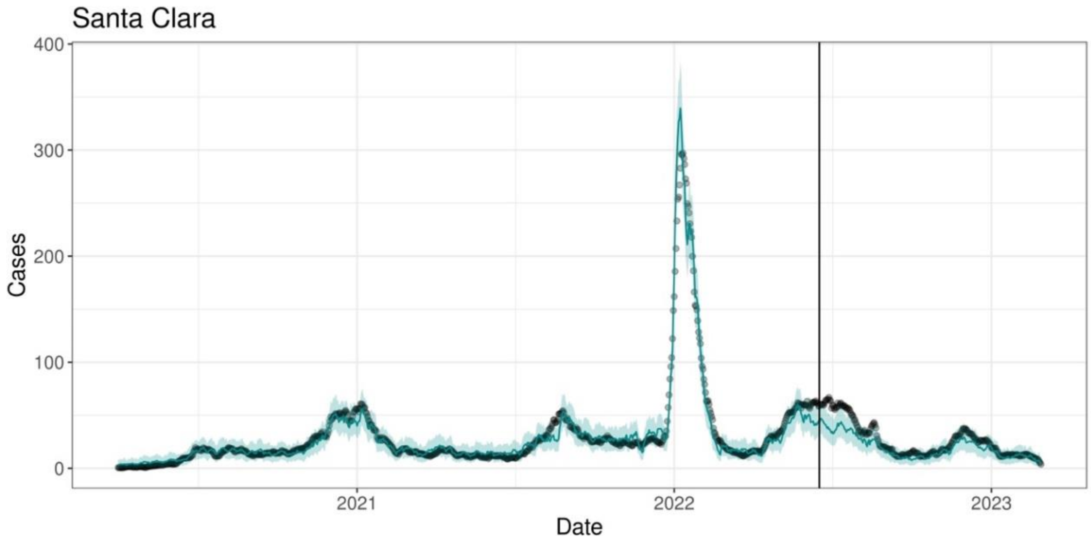

Model predictions for COVID-19 cases among children aged 6-59 months in Santa Clara County.

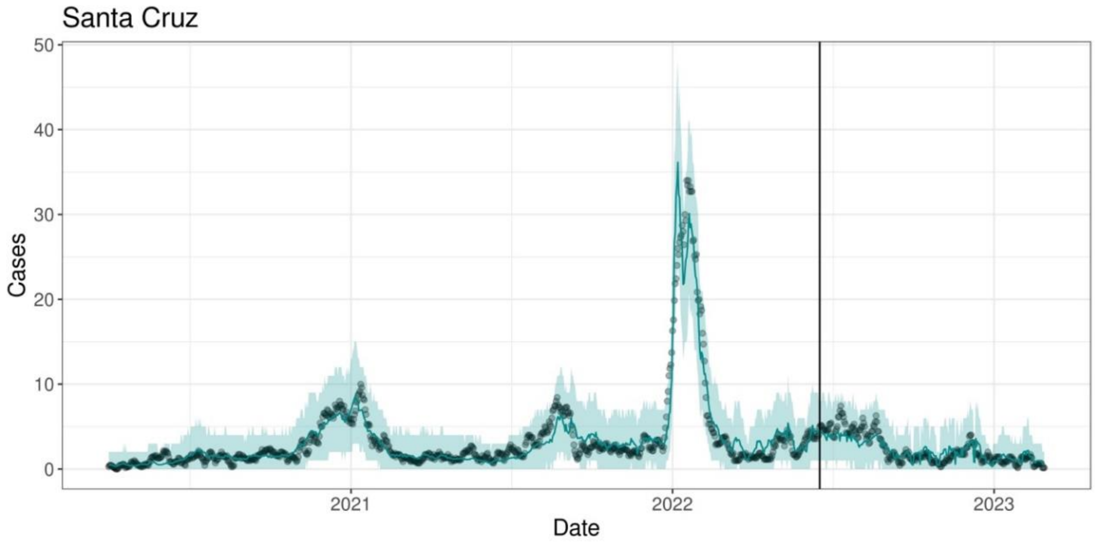

Model predictions for COVID-19 cases among children aged 6-59 months in Santa Cruz County.

© 2024 Head JR et al. *JAMA Network Open.*

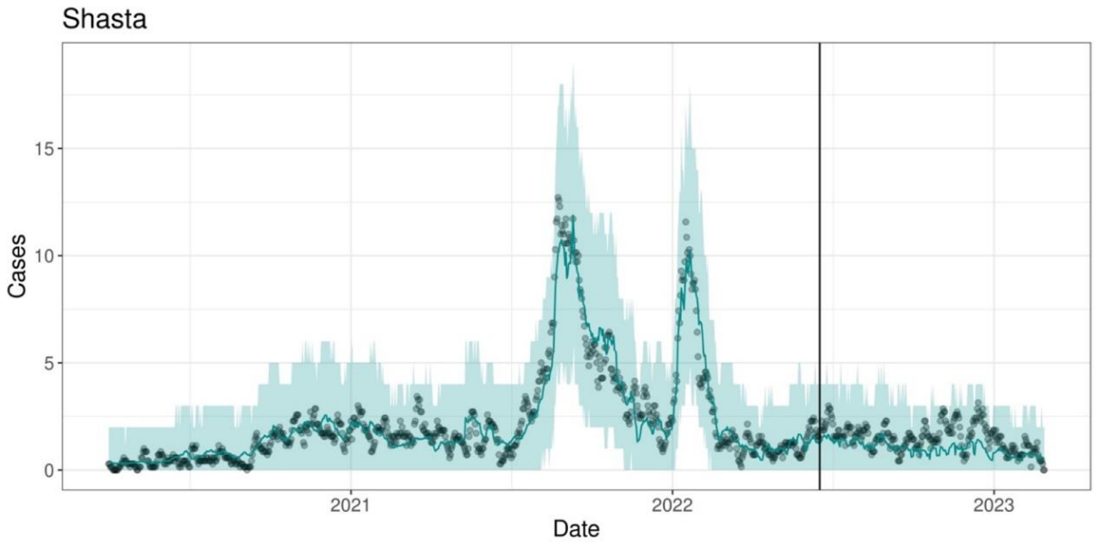

Model predictions for COVID-19 cases among children aged 6-59 months in Shasta County.

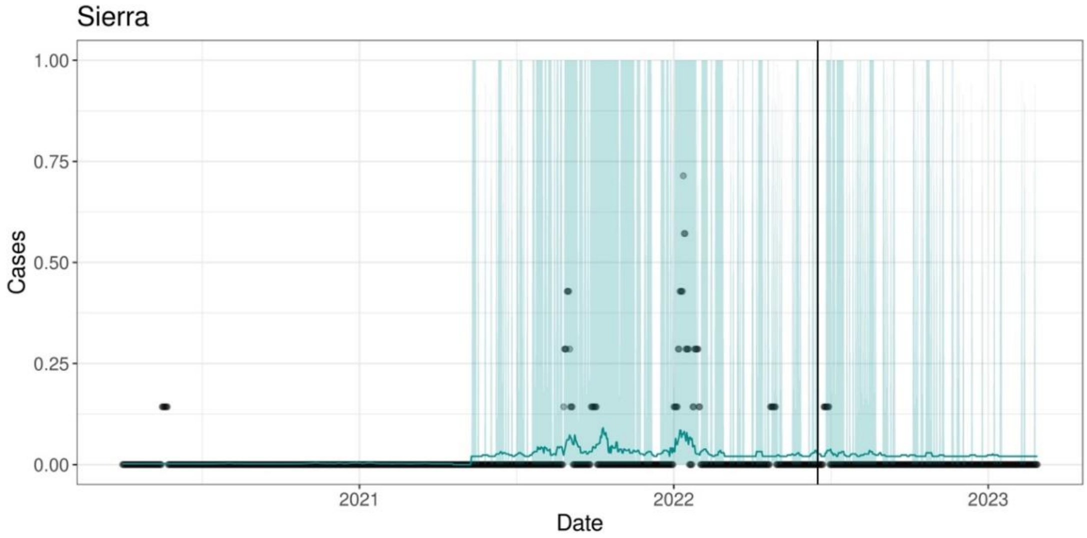

Model predictions for COVID-19 cases among children aged 6-59 months in Sierra County.

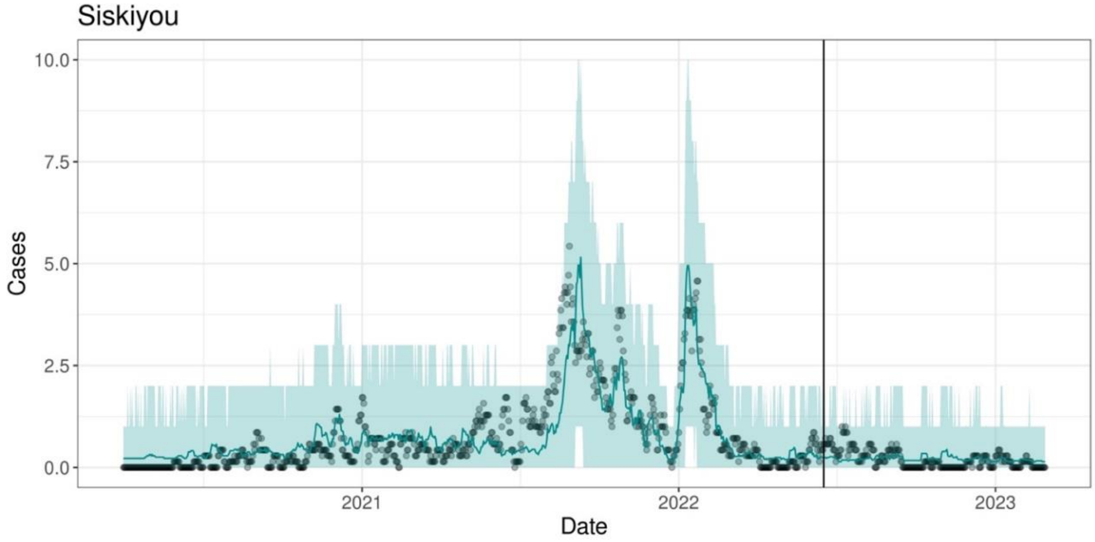

Model predictions for COVID-19 cases among children aged 6-59 months in Siskiyou County.

© 2024 Head JR et al. *JAMA Network Open.*

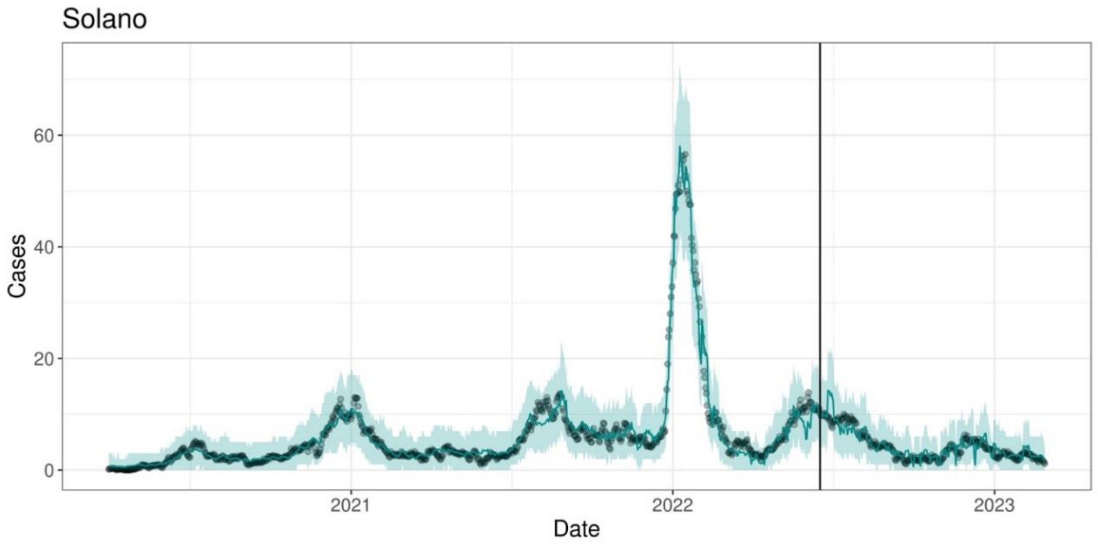

Model predictions for COVID-19 cases among children aged 6-59 months in Solano County.

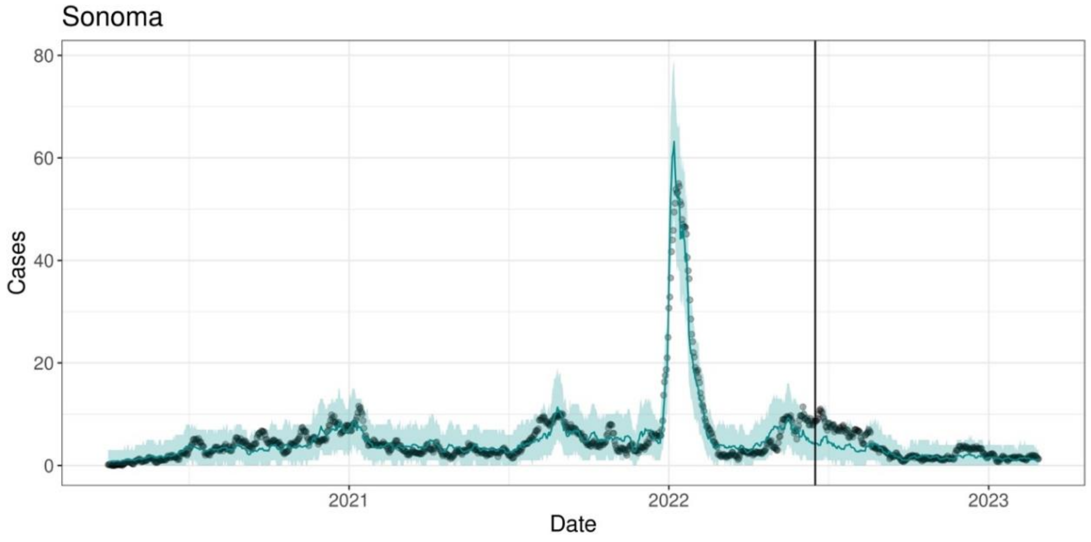

Model predictions for COVID-19 cases among children aged 6-59 months in Sonoma County.

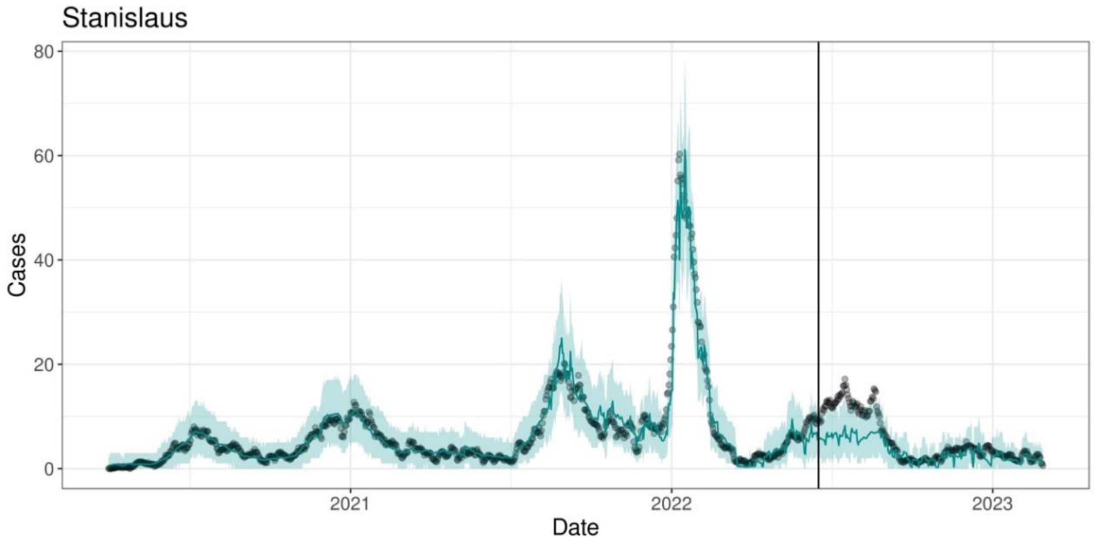

Model predictions for COVID-19 cases among children aged 6-59 months in Stanislaus County.

© 2024 Head JR et al. *JAMA Network Open.*

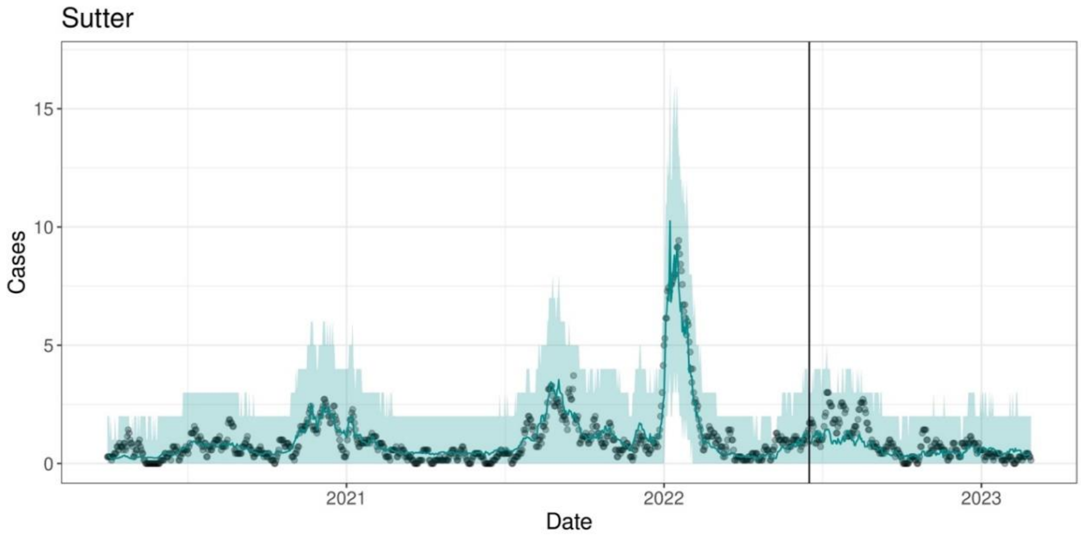

Model predictions for COVID-19 cases among children aged 6-59 months in Sutter County.

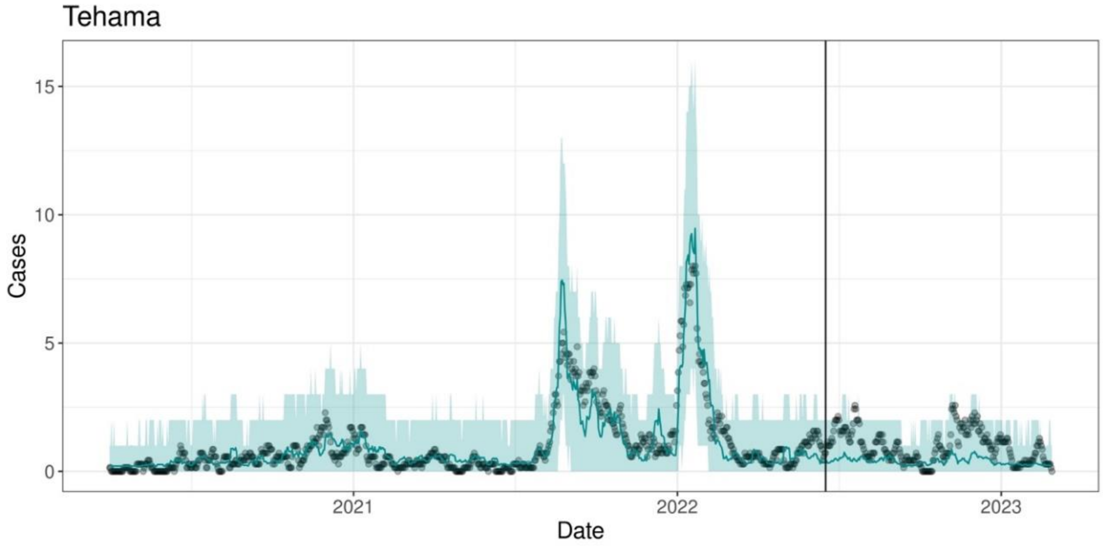

Model predictions for COVID-19 cases among children aged 6-59 months in Tehama County.

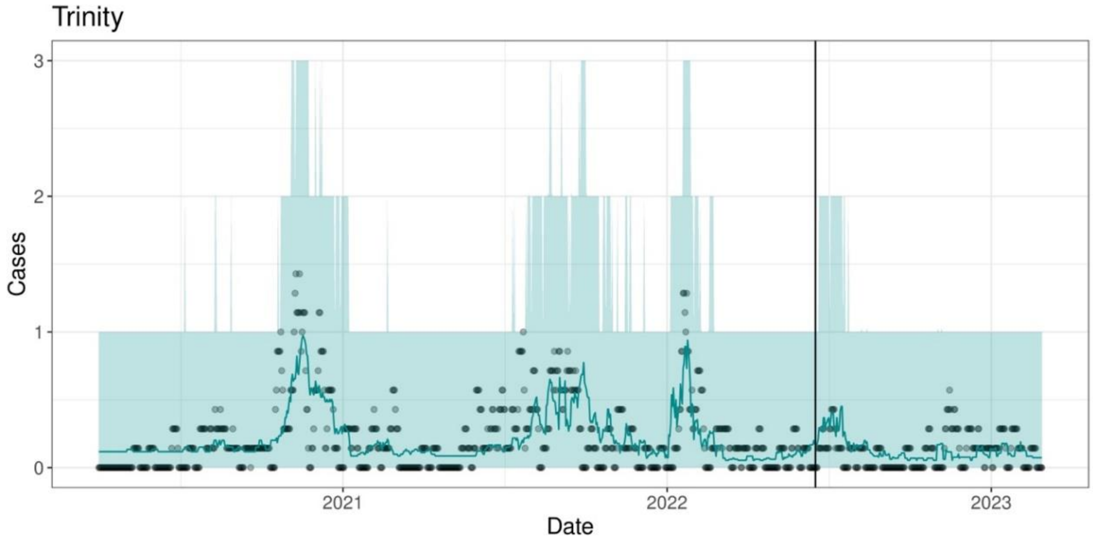

Model predictions for COVID-19 cases among children aged 6-59 months in Trinity County.

© 2024 Head JR et al. *JAMA Network Open.*

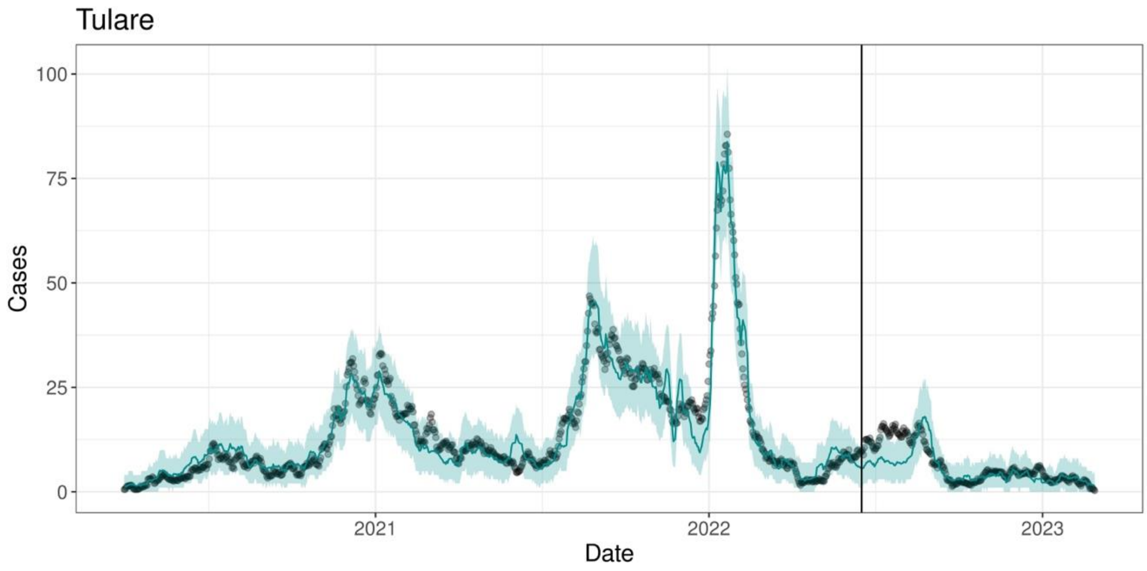

Model predictions for COVID-19 cases among children aged 6-59 months in Tulare County.

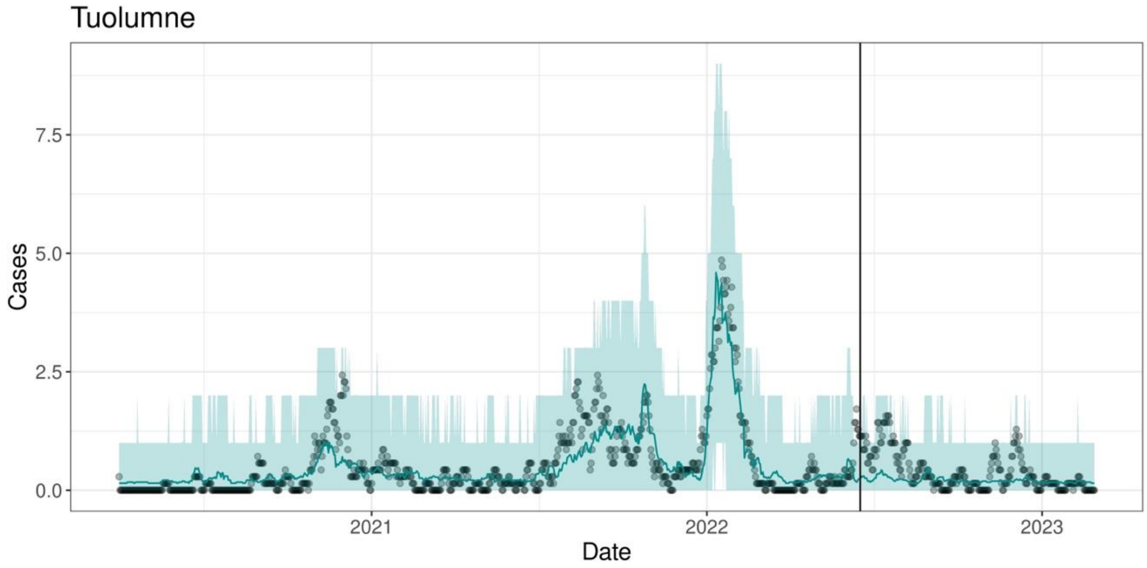

Model predictions for COVID-19 cases among children aged 6-59 months in Tuolumne County.

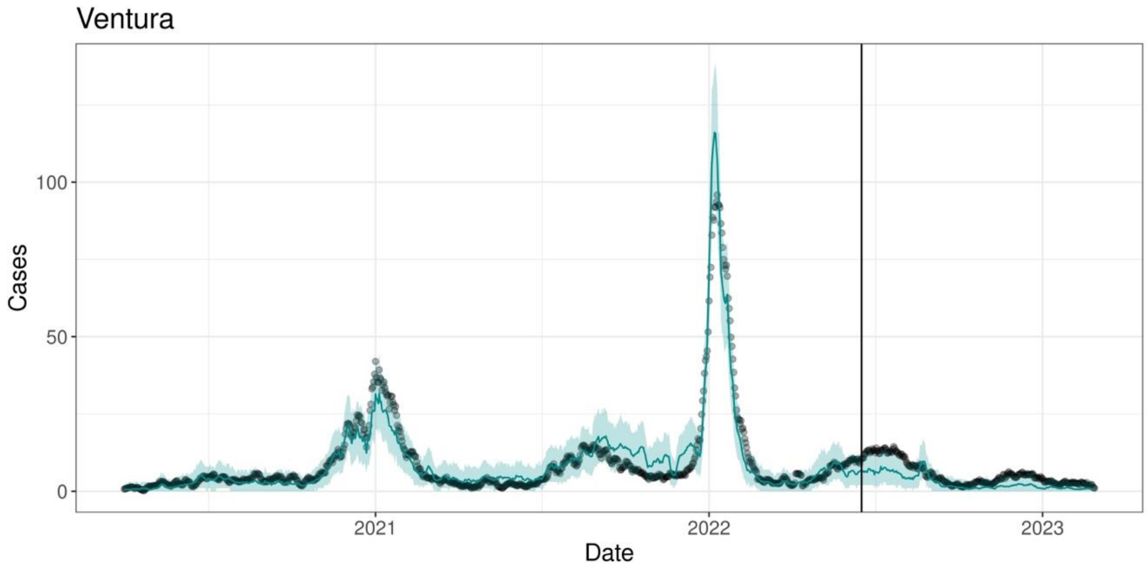

© 2024 Head JR et al. *JAMA Network Open.*

Model predictions for COVID-19 cases among children aged 6-59 months in Ventura County.

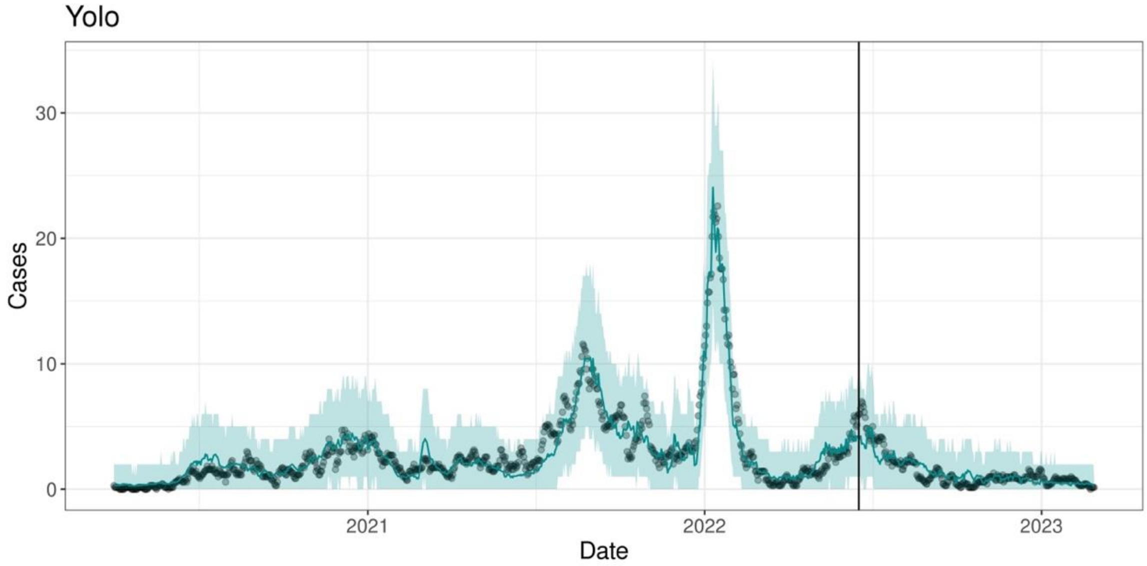

Model predictions for COVID-19 cases among children aged 6-59 months in Yolo County.

© 2024 Head JR et al. *JAMA Network Open.*

eFigure 12. Region-specific model predictions for COVID-19 hospitalizations among children aged 12-15 years. Black dots represent weekly hospitalization count, lines are model predictions, and shaded colored region is 95% prediction interval. Vertical lines are located at the time of vaccine eligibility. Shaded gray region indicates the post-vaccine evaluation period.

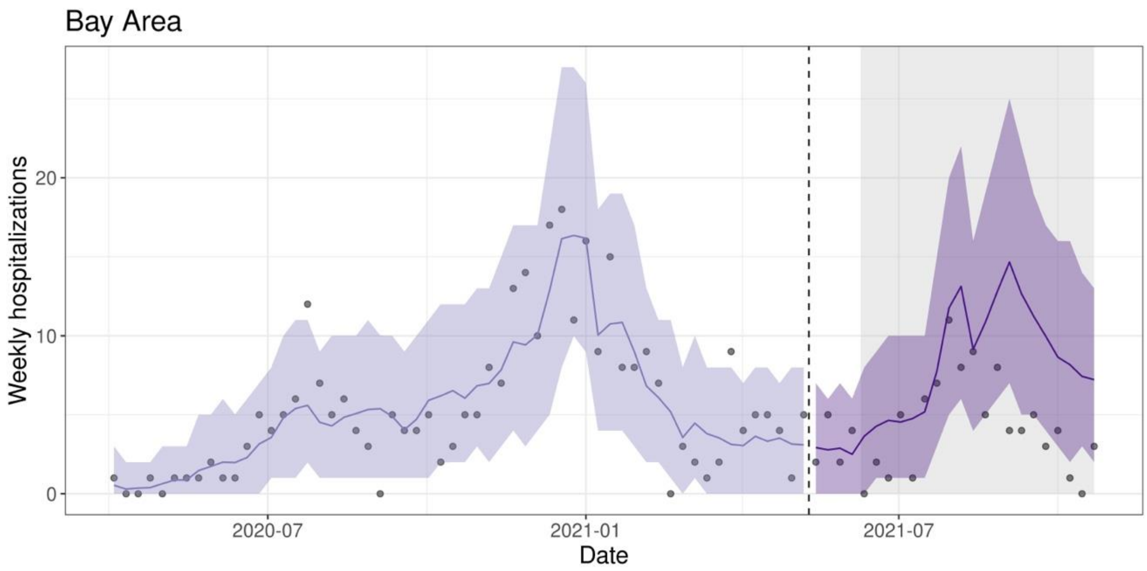

Model predictions for COVID-19 hospitalization among children aged 12-15 years in the Bay Area region of California.

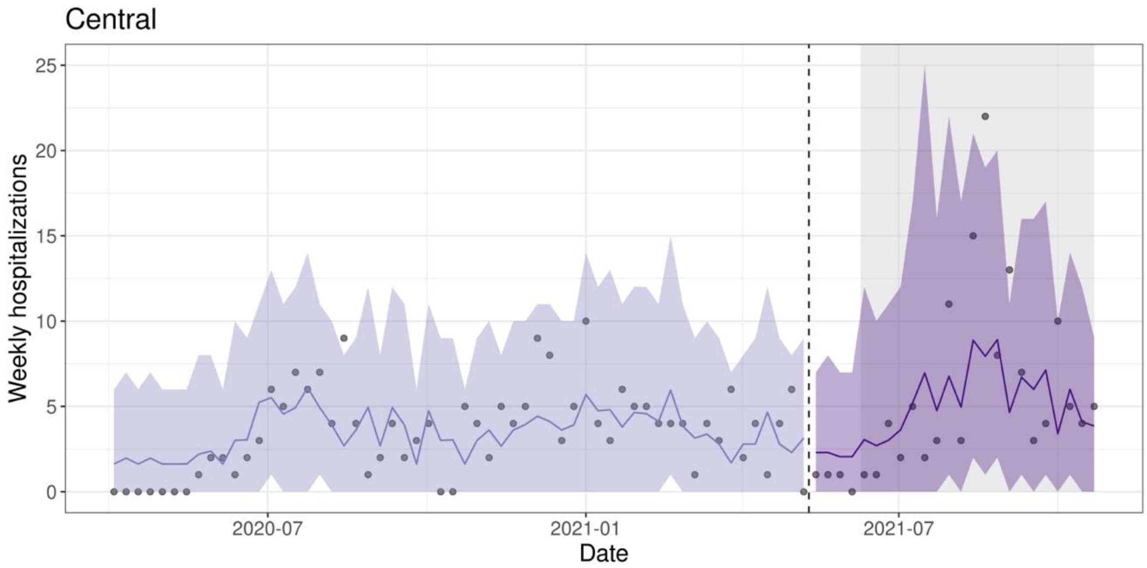

Model predictions for COVID-19 hospitalization among children aged 12-15 years in the Central region of California.

© 2024 Head JR et al. *JAMA Network Open.*

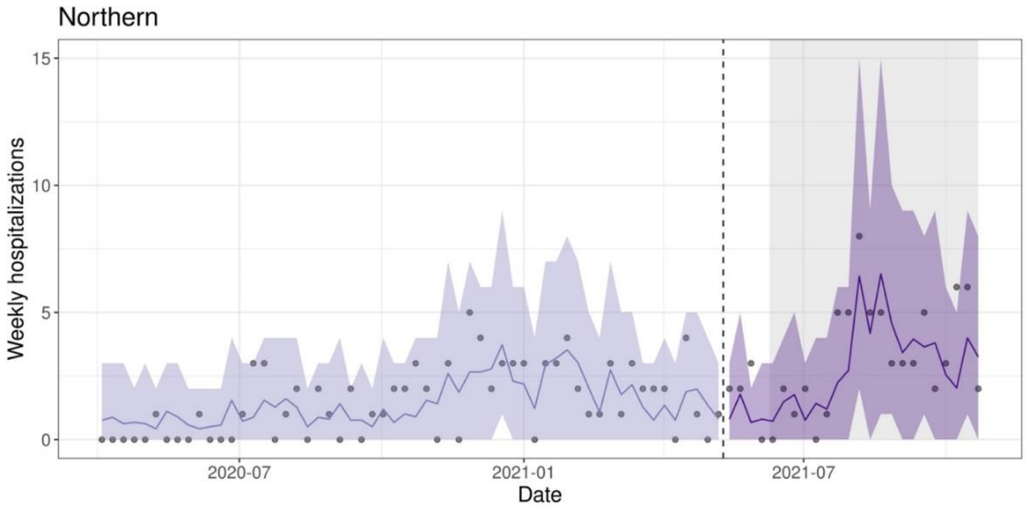

Model predictions for COVID-19 hospitalization among children aged 12-15 years in the Northern region of California.

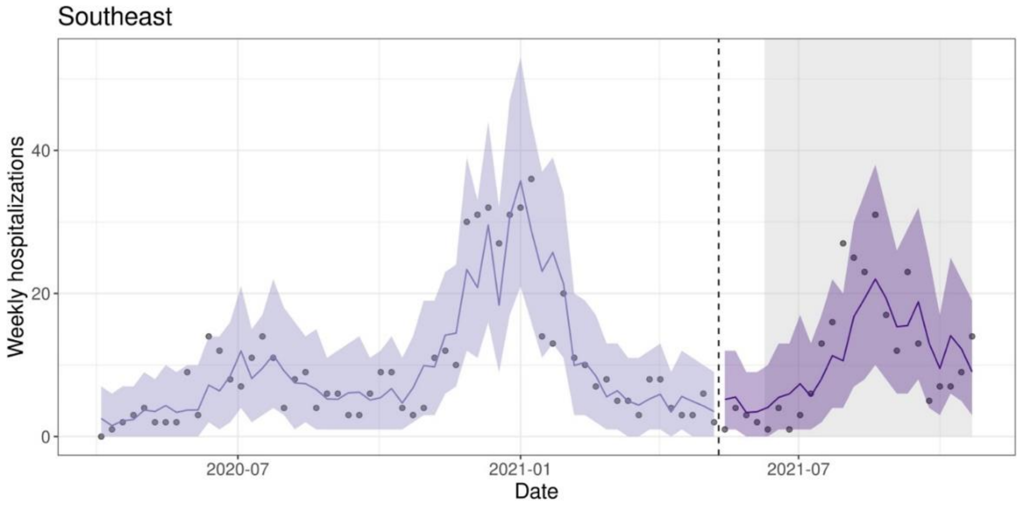

Model predictions for COVID-19 hospitalization among children aged 12-15 years in the Southeast region of California.

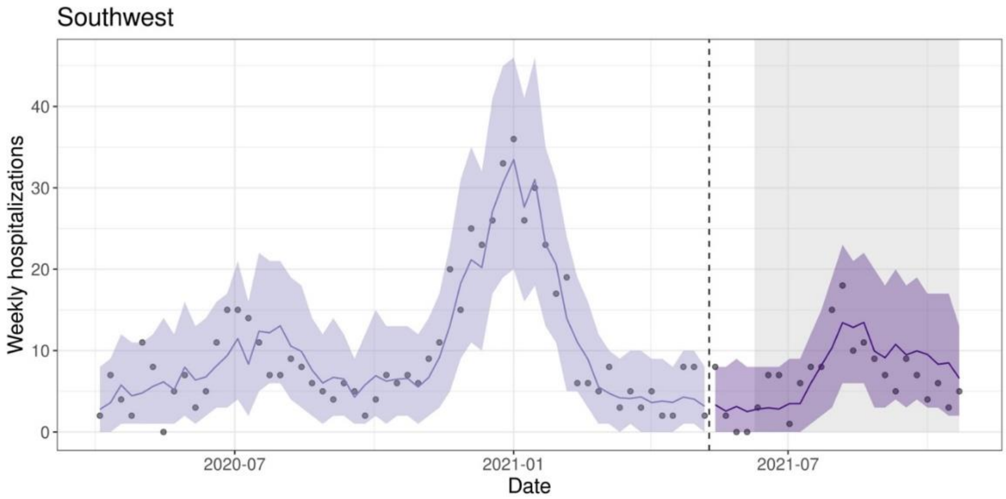

Model predictions for COVID-19 hospitalization among children aged 12-15 years in the Southwest region of California.

© 2024 Head JR et al. *JAMA Network Open.*

eFigure 13. Model predictions for COVID-19 hospitalizations among children aged 5-11 years. Black dots represent weekly hospitalization count, lines are model predictions, and shaded colored region is 95% prediction interval. Vertical lines are located at the time of vaccine eligibility. Shaded gray region indicates the post-vaccine evaluation period.

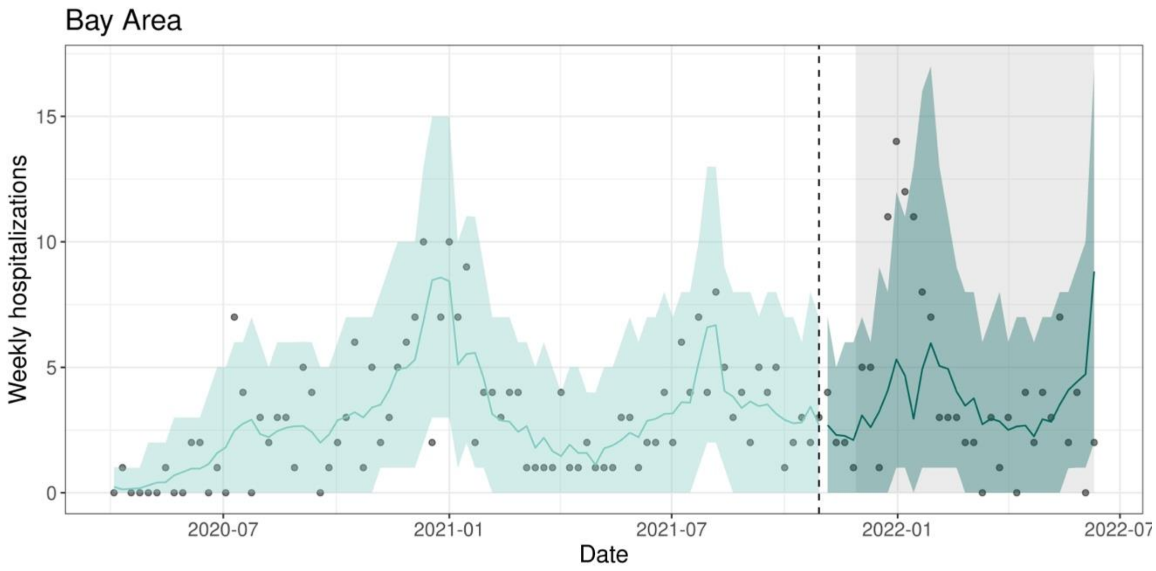

Model predictions for COVID-19 hospitalization among children aged 5-11 years in the Bay Area region of California.

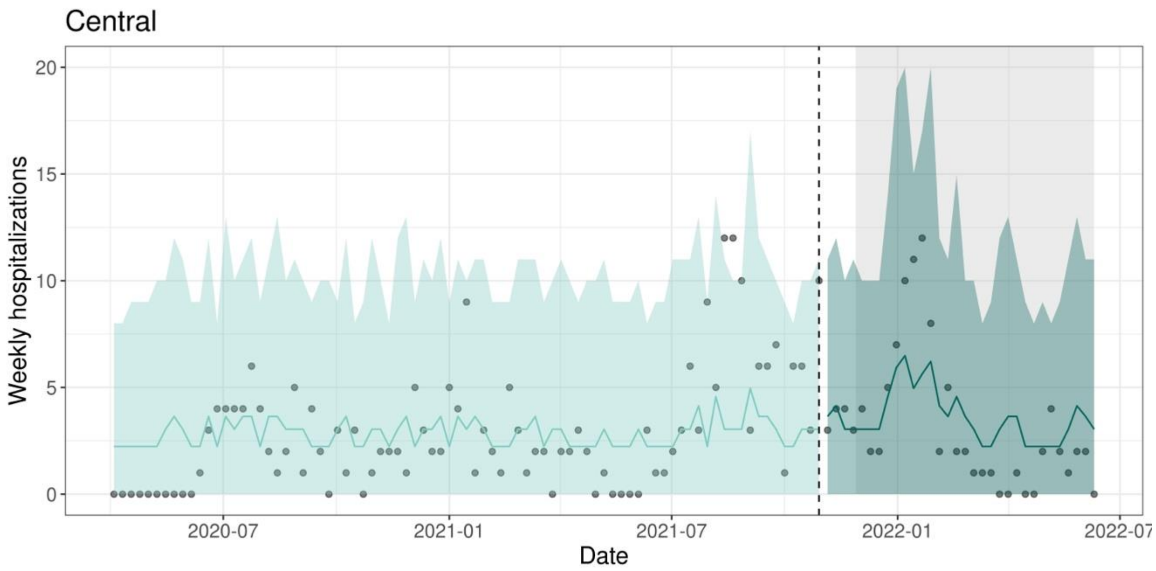

Model predictions for COVID-19 hospitalization among children aged 5-11 years in the Central region of California.

© 2024 Head JR et al. *JAMA Network Open.*

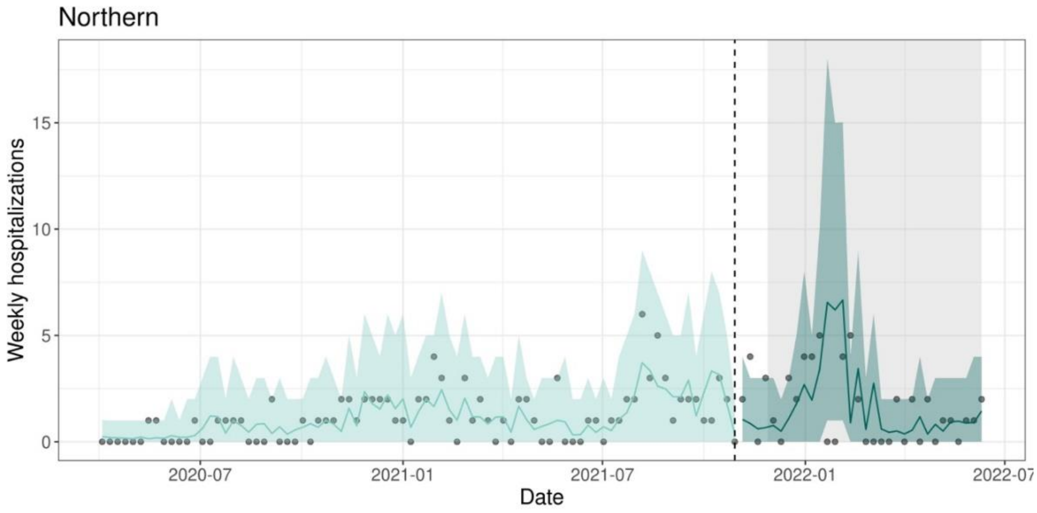

Model predictions for COVID-19 hospitalization among children aged 5-11 years in the Northern region of California.

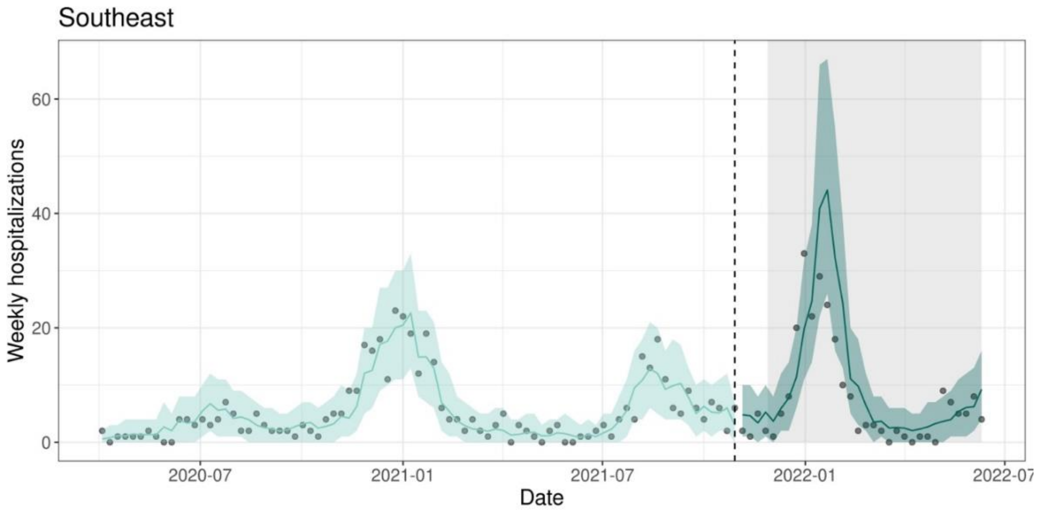

Model predictions for COVID-19 hospitalization among children aged 5-11 years in the Southeast region of California.

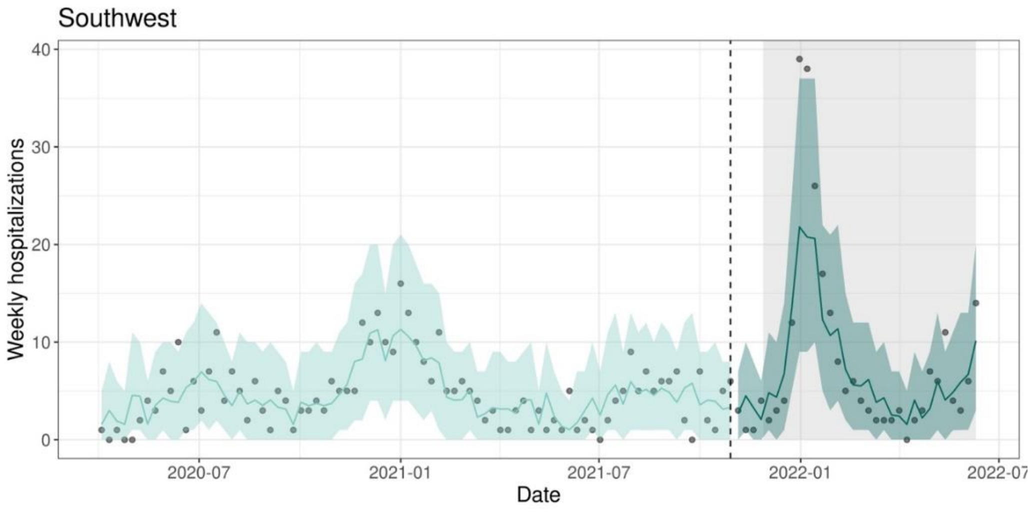

Model predictions for COVID-19 hospitalization among children aged 5-11 years in the Southwest region of California.

© 2024 Head JR et al. *JAMA Network Open.*

eFigure 14. Model predictions for COVID-19 hospitalizations among children aged 6-59 months. Black dots represent weekly hospitalization count, lines are model predictions, and shaded colored region is 95% prediction interval. Vertical lines are located at the time of vaccine eligibility. Shaded gray region indicates the post-vaccine evaluation period.

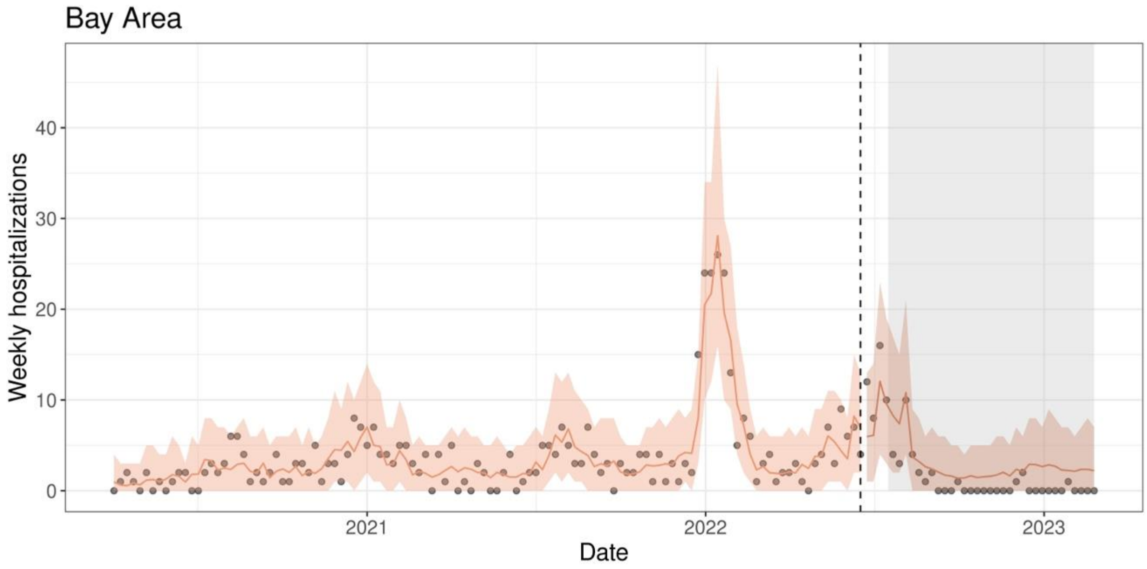

Model predictions for COVID-19 hospitalization among children aged 6-59 months in the Bay Area region of California.

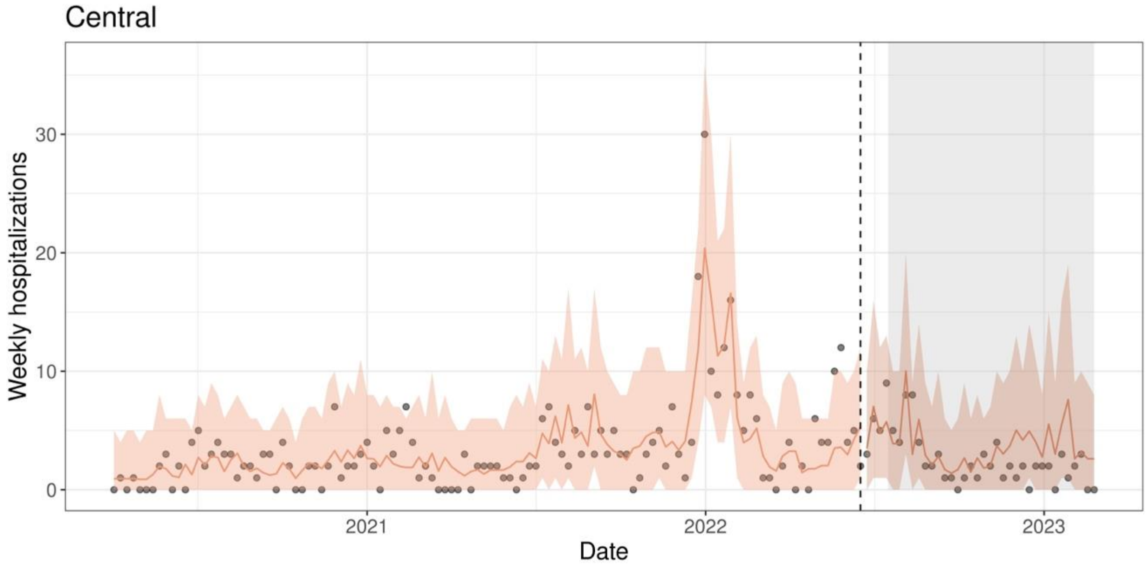

. Model predictions for COVID-19 hospitalization among children aged 6-59 months in the Central region of California.

© 2024 Head JR et al. *JAMA Network Open.*

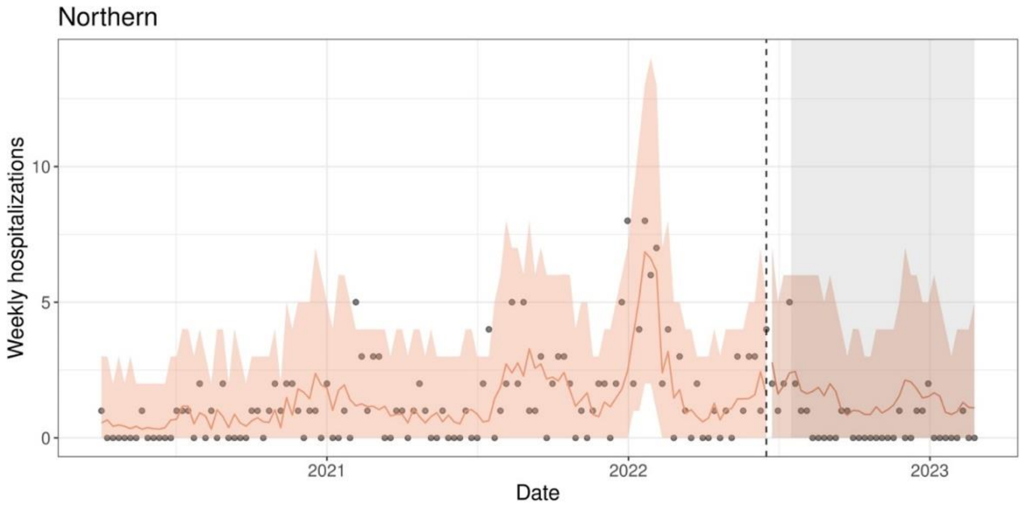

Model predictions for COVID-19 hospitalization among children aged 6-59 months in the Northern region of California.

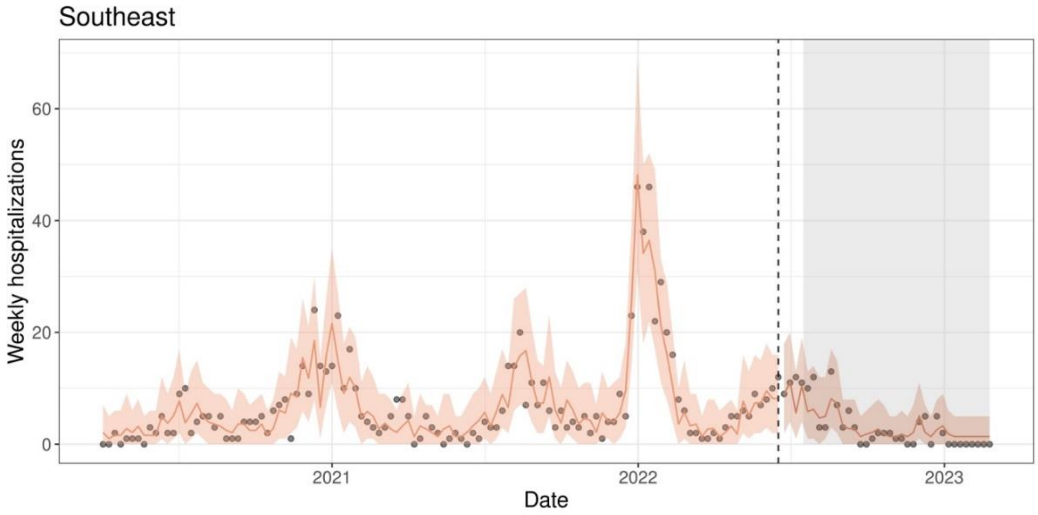

Model predictions for COVID-19 hospitalization among children aged 6-59 months in the Southeast region of California.

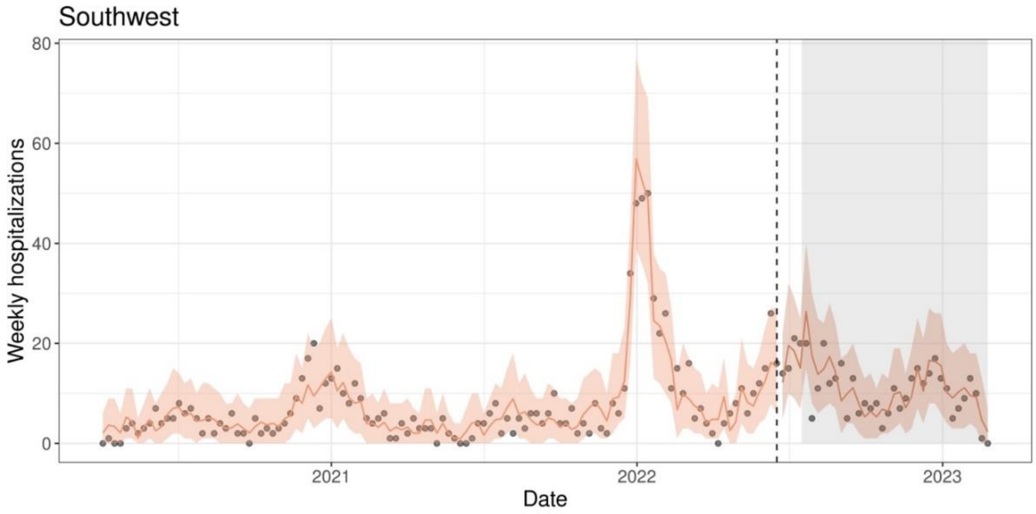

Model predictions for COVID-19 hospitalization among children aged 6-59 months in the Southwest region of California.

© 2024 Head JR et al. *JAMA Network Open.*
