# Supplementary material for: COVID-19 Vaccination and Incidence of Pediatric SARS-CoV-2 Infection and Hospitalization
Source: JAMA Netw Open. 2024 Apr 23;7(4):e247822. doi: 10.1001/jamanetworkopen.2024.7822 (PMC11040406; doi:10.1001/jamanetworkopen.2024.7822)
Supplement: Supplement 2. — Data Sharing Statement [file jamanetwopen-e247822-s002.pdf]

Data Sharing Statement

Head. COVID-19 Vaccination and Incidence of Pediatric SARS-CoV-2 Infection and Hospitalization. *JAMA Netw Open*. Published April 23, 2024.
doi:10.1001/jamanetworkopen.2024.7822

Data
Data available: No

Additional Information
Explanation for why data not available: Data was obtained via an arrangement with California Department of Public Health. Data may be made available upon request to California Department of Public Health.
